# Hydrostatic Pressure Controls Angiogenesis Through Endothelial YAP1 During Lung Regeneration

**DOI:** 10.3389/fbioe.2022.823642

**Published:** 2022-02-18

**Authors:** Tadanori Mammoto, Tendai Hunyenyiwa, Priscilla Kyi, Kathryn Hendee, Kienna Matus, Sridhar Rao, Sang H. Lee, Diana M. Tabima, Naomi C. Chesler, Akiko Mammoto

**Affiliations:** ^1^ Department of Pediatrics, Medical College of Wisconsin, Milwaukee, WI, United States; ^2^ Department of Pharmacology and Toxicology, Medical College of Wisconsin, Milwaukee, WI, United States; ^3^ Department of Cell Biology, Neurobiology and Anatomy, Medical College of Wisconsin, Milwaukee, WI, United States; ^4^ Blood Research Institute, Versiti, Milwaukee, WI, United States; ^5^ Biomedical Engineering, University of Wisconsin-Madison, Madison, WI, United States; ^6^ Edwards Lifesciences Foundation Cardiovascular Innovation and Research Center and Biomedical Engineering, University of California, Irvine, Irvine, CA, United States

**Keywords:** angiogenesis, pressure, lung, Yap1, TEAD, Tie2

## Abstract

Pulmonary artery (PA) pressure increases during lung growth after unilateral pneumonectomy (PNX). Mechanosensitive transcriptional co-activator, yes-associated protein (YAP1), in endothelial cells (ECs) is necessary for angiogenesis during post-PNX lung growth. We investigate whether increases in PA pressure following PNX control-angiogenesis through YAP1. When hydrostatic pressure is applied to human pulmonary arterial ECs (HPAECs), the expression of YAP1, transcription factor TEAD1, and angiogenic factor receptor Tie2 increases, while these effects are inhibited when HPAECs are treated with YAP1 siRNA or YAP1S94A mutant that fails to bind to TEAD1. Hydrostatic pressure also stimulates DNA synthesis, cell migration, and EC sprouting in HPAECs, while YAP1 knockdown or YAP1S94A mutant inhibits the effects. Gene enrichment analysis reveals that the levels of genes involved in extracellular matrix (ECM), cell adhesion, regeneration, or angiogenesis are altered in post-PNX mouse lung ECs, which interact with YAP1. Exosomes are known to promote tissue regeneration. Proteomics analysis reveals that exosomes isolated from conditioned media of post-PNX mouse lung ECs contain the higher levels of ECM and cell-adhesion proteins compared to those from sham-operated mouse lung ECs. Recruitment of host lung ECs and blood vessel formation are stimulated in the fibrin gel containing exosomes isolated from post-PNX mouse lung ECs or pressurized ECs, while YAP1 knockdown inhibits the effects. These results suggest that increases in PA pressure stimulate angiogenesis through YAP1 during regenerative lung growth.

## Introduction

Compensatory lung growth is induced in the remaining lung tissues after unilateral PNX in humans and other species (e.g., mice, dogs) (Hsia et al., 2001; Sakurai et al., 2007; Ding et al., 2011; Butler et al., 2012; Thane et al., 2014; Mammoto et al., 2016a; Liu et al., 2016; Mammoto T. et al., 2019; Hendee et al., 2021); the remaining alveolar units undergo epithelial and EC proliferation to compensate for the initial loss. The mechanical environment is dramatically changed after PNX (Hsia et al., 2001; Dane et al., 2013; Filipovic et al., 2013; Thane et al., 2014; Dane et al., 2016; Liu et al., 2016). For example, increases in parenchymal strain due to the expansion of the remaining lobes contribute to post-PNX lung alveolar cell growth (Hsia et al., 2001; Dane et al., 2013; Filipovic et al., 2013; Liu et al., 2016). Increases in PA pressure due to redirection of the entire cardiac output through remaining lung lobes also play important roles in post-PNX lung growth (Dane et al., 2013; Dane et al., 2016).

Angiogenesis -growth of new capillaries-constitutes an essential part of the regenerative program (Sakurai et al., 2007; Ding et al., 2011; Mammoto et al., 2016a; Mammoto T. et al., 2019; Mammoto and Mammoto, 2019; Hendee et al., 2021). Inhibition of angiogenesis impairs compensatory lung growth after PNX in adult mice (Sakurai et al., 2007; Ding et al., 2011; Mammoto et al., 2016a; Mammoto T. et al., 2019; Hendee et al., 2021). Mechanical factors such as ECM stiffness, shear stress, and stretching forces control angiogenesis and vascular function (Mammoto et al., 2009; Thodeti et al., 2009; Mammoto A. et al., 2013; Mammoto T. et al., 2013; Mammoto and Mammoto, 2019). Hippo signaling transducer, YAP1, acts as a transcriptional co-activator and controls organ size, development, and regeneration (e.g., liver, heart, intestine, muscle, lung) (Barry et al., 2013; Piccolo et al., 2013; Lin and Pu, 2014; Mahoney et al., 2014; Yimlamai et al., 2014; Yu et al., 2015; Liu et al., 2016; Mammoto T. et al., 2019). In the lung, YAP1 activation in alveolar epithelial stem cells and ECs promotes post-PNX lung growth (Liu et al., 2016; Mammoto T. et al., 2019), while deregulation of YAP1 contributes to chronic obstructive pulmonary disease (Makita et al., 2008) and pulmonary fibrosis (Liu et al., 2015; Panciera et al., 2017). YAP1 stimulates angiogenesis in the retina and other organs (Choi et al., 2015; Nakajima et al., 2017; Mammoto A. et al., 2018; Boopathy and Hong, 2019). YAP1 is a mechanosensitive gene and its activity is controlled by mechanical stimuli (e.g., cell shape and size (Dupont et al., 2011; Panciera et al., 2017), cell density (Ota and Sasaki, 2008; Choi et al., 2015), rigidity and topology of ECM (Dupont et al., 2011; Bertero et al., 2016), shear stress (Dupont et al., 2011; Wang et al., 2016; Nakajima et al., 2017; Panciera et al., 2017), mechanical tension (Liu et al., 2016)). In our previous study, we demonstrated that YAP1 in ECs is necessary for angiogenesis during post-PNX lung growth (Mammoto T. et al., 2019). Given that 1) mechanical environment changes after PNX, which drives post-PNX lung growth (Hsia et al., 2001; Dane et al., 2013; Filipovic et al., 2013; Liu et al., 2016), 2) YAP1 senses various mechanical stimuli (Ota and Sasaki, 2008; Dupont et al., 2011; Choi et al., 2015; Bertero et al., 2016; Liu et al., 2016; Wang et al., 2016; Nakajima et al., 2017; Panciera et al., 2017), and 3) mechanical forces control angiogenesis (Mammoto et al., 2009; Thodeti et al., 2009; Mammoto A. et al., 2013; Mammoto T. et al., 2013; Mammoto and Mammoto, 2019), in this study we have investigated whether post-PNX changes in the mechanical forces control angiogenesis through endothelial YAP1. It has been reported that increased mechanical stretch after PNX induces post-PNX lung growth through epithelial YAP1 signaling (Liu et al., 2016). However, the effects of PA pressure altering during post-PNX lung growth on angiogenesis have not been explored. Here we examined whether increases in PA pressure after PNX stimulate angiogenesis through YAP1.

Exosomes are one of the types of extracellular vesicles produced by almost all cell types, including ECs (Thery et al., 2002; Pant et al., 2012; Kourembanas, 2015; Davidson et al., 2018). Exosomes contain numerous proteins, lipids, and various types of nucleic acids (DNA, mRNA, miRNA, noncoding RNA) (Thery et al., 2002; Kourembanas, 2015). Exosomes are released from cells to serve as a messenger of signals for cell-cell communications as well as to remove unused or harmful RNA and proteins, maintaining tissue homeostasis and function in normal physiology and in diseases (e.g., aging (Xie et al., 2018), cancer (Jakhar and Crasta, 2019), atherosclerosis (Chang et al., 2019), pulmonary hypertension (Lee et al., 2012; Klinger et al., 2020; Mohan et al., 2020; Sindi et al., 2020; Otsuki et al., 2021)) (Thery et al., 2002; Thery et al., 2009; Pant et al., 2012; Kourembanas, 2015; Jakhar and Crasta, 2019; Otsuki et al., 2021). Exosomes promote angiogenesis and stimulate cardiac regeneration (Ibrahim et al., 2014; Shanmuganathan et al., 2018) and recovery from ischemia (Sahoo et al., 2011; Bang et al., 2014; Shanmuganathan et al., 2018). It has been reported that human mesenchymal stem cell (MSC)-derived exosomes ameliorate various lung diseases in animal models (e.g., pulmonary hypertension, bronchopulmonary dysplasia (BPD), airway inflammation, pulmonary fibrosis) (Lee et al., 2012; Cruz et al., 2015; Willis et al., 2018; Genschmer et al., 2019; Mansouri et al., 2019; Dinh et al., 2020; Klinger et al., 2020; Mohan et al., 2020; Sindi et al., 2020). However, the role of exosomes from ECs in lung regeneration has not been studied before.

Here we demonstrate that increases in PA pressure after PNX stimulate angiogenesis through endothelial YAP1 signaling. Exosomes isolated from post-PNX mouse lung ECs or pressured ECs stimulate angiogenesis in the mouse lungs. Modulation of mechanical environment or endothelial YAP1 signaling could improve the strategy for regenerative lung growth.

## Materials and Methods

### Materials

Anti-CD31 and -GM130 antibodies were from BD Biosciences (Franklin Lakes, NJ). Anti-YAP1- and -flotillin-1 antibodies were from Cell Signaling (Danvers, MA). Anti-β-actin antibody was from Sigma (St. Louis, MO). Anti-Tie2 monoclonal antibody was from Upstate (Lake Placid, NY). Anti-YAP1 and -CD63 antibodies were from Santa Cruz Biotechnology (Dallas, TX). Anti-ki67 antibody was from eBioscience (San Diego, CA). Anti-SPB and -ERG antibodies were from Abcam (Waltham, MA). Human pulmonary arterial endothelial cells (HPAECs, Lonza) were cultured in EBM2 medium containing 5% FBS and growth factors (VEGF, bFGF and PDGF).

### Gene Knockdown and Overexpression

Gene knockdown was performed using the RNA interference technique. siRNA for human YAP1 (5′-GAC​AUC​UUC​UGG​UCA​GAG​A-3′ and 5′- UCU​CUG​ACC​AGA​AGA​UGU​C-3′) was purchased from Sigma Genosys (St. Louis, MO) (Mammoto A. et al., 2018; Mammoto T. et al., 2019). HPAECs were transfected using siLentFect (BioRad, Hercules, CA) (Mammoto et al., 2009; Mammoto T. et al., 2019). As a control, siRNA duplex with an irrelevant sequence (QIAGEN, Germantown, MD) was used. The retroviral pQCXIH-myc-YAP (human) construct was a gift from Kunliang Guan. The lentiviral pLX304-YAP1(S94A) (human) construct was a gift from William Hahn. Generation of viral vectors was accomplished as reported (Mammoto et al., 2009; Mammoto et al., 2016a; Mammoto T. et al., 2019). HPAECs were incubated with viral stocks in the presence of 5 μg/ml polybrene (Sigma) and 90–100% infection was achieved 3 days later (Mammoto et al., 2009; Mammoto et al., 2016a; Mammoto T. et al., 2019).

### Molecular Biological and Biochemical Methods

Quantitative reverse transcription (qRT)-PCR was performed with the iScript reverse transcription and iTaq SYBR Green qPCR kit (BioRad) using the BioRad real time PCR system. β2 microglobulin controlled for overall cDNA content. The primers for human YAP1, TEAD1, Tie2, and β2 microglobulin were previously described (Mammoto et al., 2009; Mammoto A. et al., 2018; Mammoto T. et al., 2019). The primers for human CYR61 forward; CATTCCTCTGTGTCCCCAAGAA and reverse; TAC​TAT​CCT​CGT​CAC​AGA​CCC​A, human ANKRD1 forward; TGA​TTA​TGT​ATG​GCG​CGG​ATC​T and reverse; GCG​AGA​GGT​CTT​GTA​GGA​GTT​C, and human KLF2 forward; GCC​CUA​CCA​CUG​CAA​CUG​GUU and reverse; CCA​GUU​GCA​GUG​GUA​GGG​CUU.

### *In vitro* Pressure Experiment

To analyze the effects of hydrostatic pressure, HPAECs were pressurized in a pressure chamber that fits into a cell culture incubator (Prystopiuk et al., 2018) (Strex Cell, SanDiego, CA). Cells were exposed to graded pressures ranging from 0 to 50 mmHg (6.6 kPa). Since human PA pressure is 15–20 mmHg and PA pressure increases by 1.8–2.0 times in an *in vivo* mouse PNX model, we pressurized HPAECs with the range of 0–40 mmHg (0, 15, 30, 40 mmHg). As a control, cells were cultured in a standard incubator.

DNA synthesis of HPAECs pressurized (0, 40 mmHg) for 16 h was analyzed using an EdU assay. EdU-positive cells were analyzed using a Nikon A1 confocal imaging system (Mammoto A. et al., 2018; Mammoto T. et al., 2018; Mammoto T. et al., 2019). EC migration was measured using a modified transwell migration assay. Since we focus on YAP1-Tie2 signaling in this study, we used a Tie2 ligand angiopoietin1 (Ang1) as a chemoattractant in the migration assay. The cells that migrated towards Ang1 (10 ng/ml) in 0.5% serum EBM-2 through the membrane were stained with Giemsa, counted and averaged in three or more independent experiments.

*In vitro* fibrin gel angiogenesis assay was performed as previously described (Mammoto et al., 2016a; Mammoto et al., 2016b; Mammoto T. et al., 2019). Briefly, 1 × 10^5^ HPAECs were incubated with 3,000 Cytodex 3 microcarrier beads (GE Healthcare Life Sciences, Pittsburgh, PA) in 1 ml 5% FBS/EGM2 in a glass tube for 4 h with gentle agitation. The beads coated with the cells were transferred to 25 cm^2^ tissue culture flask and incubated with YAP1 siRNA or virus treatment. As a control, cells were treated with control siRNA with irrelevant sequence or control virus (full length YAP1). After 16 h incubation, 250 beads coated with HPAECs were suspended in 500 µl of 2.5 mg/ml fibrinogen solution (Sigma) and mixed with 500 µl of thrombin solution (0.5 U) in a 24-well plate. After fibrin gels were solidified, 1 ml of 1% FBS/EGM2 containing 2 × 10^4^ human lung fibroblasts was seeded on top of each fibrin gel in a 24-well plate. Ang1 (20 ng/ml total protein) was added to the medium at day 1 and the medium was changed every other day. After incubation of beads in the fibrin gels for 5 days, the area of the sprout from the beads was quantified using ImageJ software.

### Mouse Lung Endothelial Cells Isolation and Culture

Mouse lung ECs were isolated using anti-CD31 conjugated magnetic beads (Mammoto T. et al., 2018; Mammoto T. et al., 2019; Mammoto et al., 2020). We cut mouse lung tissue into small pieces using small scissors and treated the tissue with 5 ml collagenase A (1 mg/ml) for 30 min at 37°C. The tissue suspension was filtered through a 40 μm cell strainer (Falcon) to remove the undigested tissue clumps and separate single cells. Cells were centrifuged (180 g, 5 min) at room temperature (RT) and the pellet was resuspended into 0.5 ml RBC Lysis Buffer (Sigma, 1 min, RT). The lysis reaction was stopped by adding 10 ml 10% FBS/DMEM, being centrifuged (180 g, 5 min, RT), and the pellet was resuspended into 0.5 ml 4% FBS/PBS with APC anti-mouse CD31 (Biolegend, 1/100), incubated (20 min, on ice) and washed three times with 4% FBS/PBS. Cells were centrifuged (180 g, 5 min, RT) and resuspended into 0.1 ml 4% FBS/PBS with anti-APC conjugated microbeads (Miltenyl Biotec, Somerville, MA), incubated (10 min, on ice) and washed three times with 4% FBS/PBS. The cells were then resuspended in 0.5 ml 4% FBS/PBS and CD31-positive ECs were magnetically separated using MACS column (Miltenyl Biotec) according to the manufacturer’s instruction. To increase the purity of the magnetically separated fraction, the eluted fraction was enriched over a second new MACS column. Isolated ECs were cultured on tissue culture dishes under 5% FBS/EGM2 for the subsequent experiments.

### Unilateral Pneumonectomy

The *in vivo* animal study was carried out in strict accordance with the recommendations in the Guide for the Care and Use of Laboratory Animals of the National Institutes of Health. The protocol was reviewed and approved by the Animal Care and Use Committee of Medical College of Wisconsin. Unilateral PNX was performed as described (Mammoto et al., 2016a; Mammoto T. et al., 2019; Hendee et al., 2021). Briefly, mice (CD1, C57BL6J, 8–12 week old, approximately 25 g, both male and female mice were used except for RNAseq samples where only male mice were used) were anesthetized with Ketamine (100 mg/kg)/Xylazine (10 mg/kg, intraperitoneal injection), intubated and mechanically ventilated using a rodent ventilator (MiniVent, Harvard Apparatus, Holliston, MA). After ensuring adequate anesthesia, thoracotomy was performed and the left lung was lifted through the incision and a 5–0 silk suture was passed around the hilum and tied. The hilum was then transected distal to the tie. The remaining portions of the hilum and tie were returned to the thoracic cavity. Sham-operated mice underwent thoracotomy without PNX. Meloxicam (5 mg/kg, subcutaneous injection, 3 days) was used as a postoperative analgesic. The proximal PA pressure was measured by PA cannulation under thoracotomy (Mammoto T. et al., 2018). DNA synthesis and proliferation of ECs (CD31^+^, VE-Cadherin^+^, CD45^−^) and epithelial cells (EpCAM^+^) in the mouse lungs were analyzed by measuring the number of bromodeoxyuridine (BrdU)^+^ cells using FACS (BD Biosciences BrdU flow kit) (Mammoto A. et al., 2019) and ki67 staining. Since there is not a significant difference between male and female in the effects of PNX on lung weight and PA pressure (not shown), we pooled the data from male and female in this study.

### RNA Sequencing and Analysis

ECs were isolated from mouse lungs 7 days after PNX and sham-operated C57BL6J mouse lungs (8 week old, n = 2 per group, each *n* was pooled from 2 male mice. Jackson Laboratory, stock # 664) as described above and isolated ECs were validated by FACS for EC markers (CD31^+^, VE-Cadherin^+^, CD45^−^). We used only male mice for RNAseq analysis to avoid hormonal effects. RNA was extracted using RNeasy mini kit (QIAGEN). Total RNA samples were submitted to the Institute for Systems Biology Molecular and Cell Core (Seattle, WA) for RNA sequencing. Library preparation was employed using the Illumina TruSeq Stranded mRNA kit. Sequencing was performed using the Illumina NextSeq500. Paired end sequencing was performed on a high output 150 cycle kit v2.5. The RNA sequencing reads were aligned to the mm10 reference genome. Differential gene expression analysis and Fragments Per Kilobase Million (FPKM) calculation were performed by Basepair Tech (www.basepairtech.com) using the DEseq2 pipeline (Supplementary Table S3). 831 upregulated and 180 downregulated significantly differentially expressed genes were defined as having a log2 fold change > 1 or < -1 and a p-adjusted value <0.01 with the FDR cutoff of 0.01 calculated by the Benjamini–Hochberg adjustment. Biological Processes Gene ontology (BP GO) analysis of significant targets was done via The Database for Annotation, Visualization and Integrated Discovery (DAVID) v 6.8 using the Functional Annotation Chart tool. Three different GO Terms charts were generated. The first examined all 1,011 significantly differentially expressed genes and produced 345 BP GO Term categories (Supplementary Table S4). For the second, a focus on mechanosensitive-related genes led to 522 significantly differentially expressed genes—460 upregulated and 62 downregulated—being detected as appearing on a master list comprised of Gene Card and BP GO Term categories relating to extracellular matrix (ECM), cell-cell junctions, the Hippo pathway, and cellular responses to mechanical forces including shear stress, tension, pressure, and stiffness/elasticity. 416 BP GO categories were returned from the resulting DAVID analysis (Supplementary Table S5). Network generation was performed on the 522 mechanosensitive significantly differentially expressed genes with Ingenuity Pathway Analysis (IPA) software (QIAGEN). The network was constructed by starting with the shortest interactions identified between the mechanosensitive genes and Yap1, then determining the shortest connections between those connected to Yap1 and all others, and finally adding the shortest connections between all genes connected to Yap1 and only the remaining unconnected genes. Many genes were trimmed if they connected to less than 4 other genes. The resulting network was comprised of 233 genes -220 upregulated and 13 downregulated-that underwent BP GO analysis in DAVID to generate the third BP GO Term table with 371 categories. The BP GO Terms in this third table were color-coded into groups relating to: development, regeneration, and angiogenesis; ECM and cell adhesion; cell growth, proliferation, and migration; cell cycle, repair, metabolism, senescence, and apoptosis; cellular signaling and protein processing; and inflammatory and immune responses (Supplementary Table S6, Figure 2B). These groups were also used to color the network genes accordingly. Heatmaps of the top 20 upregulated and 13 downregulated network genes as well as the 46 network genes comprising the top 25 BP GO Term categories were generated by Basepair (Supplementary Figure S3). RNAseq results are available in NCBI Geo (GSE154110).

### Exosome Isolation and Purification

ECs isolated from C57BL6J mouse lungs were plated at 1 × 10^6^ cells per 6 cm tissue culture dish with EGM2 medium containing 5% exosome depleted FBS (Thermo Fisher Scientific) and pre-filtered (0.2 μm) conditioned media was collected after 24 h. Exosomes were isolated from conditioned media and mouse serum using Total Exosome Isolation Reagent from Cell Culture Media and serum (Thermo Fisher Scientific, Waltham, MA), respectively, according to the manufacturer’s protocol (Gartz et al., 2018; Doyle and Wang, 2019; Gartz et al., 2020). The exosome pellet was resuspended in 25 μl of filtered (0.2 μm) PBS. Isolated exosomes were confirmed with exosome marker proteins (CD63, flotillin-1) using immunoblotting (IB).

For transmission electron microscopy (TEM) to analyze the ultrastructure of the exosome, resuspended exosomes were adsorbed onto freshly ionized, 400 mesh formvar/carbon grids, washed once with distilled water, and negatively stained with 2% aqueous Uranyl acetate. Exosome preparations were viewed in a Hitachi H600 transmission electron microscope and images were recorded with a Hamamatsu CCD camera using AMT image capture software.

Size and concentration distributions of exosomes were determined using nanoparticle tracking analysis (NTA; NanoSight LM10 system, Malvern instruments, Malvern, United Kingdom) (Gartz et al., 2018).

### Proteomics Analysis

Proteomics analysis of exosomes was performed by the Northwestern University Proteomics Core Facility. Isolated exosomes were briefly tip sonicated (∼10 s) to break the exosome membrane and purified proteins by acetone/TCA precipitation. Then, the proteins were reduced, alkylated, and digested with trypsin according to the optimized protocol. Digested peptides were desalted on C18 columns then subjected to mass spectrometry analysis. Data was searched against a Mus musculus database. Proteomics data analysis on two control (sham) and three PNX (7 days after PNX) mouse exosome replicates was performed using Scaffold 5.1.0 software. 228 proteins were identified in at least one of both the control and PNX sample replicates. A cutoff threshold of greater than or equal to eight Total Spectrum Counts in at least one overall replicate was used to further narrow the list to 152 proteins of interest (Supplementary Table S2). The differentially expressed genes associated with the 152 proteins underwent BPGO analysis via the Functional Annotation Chart feature of the DAVID v6.8 software (Figure 5D).

### Fibrin Gel Implantation on the Mouse Lung *in vivo*

Fibrin gel was fabricated as described (Mammoto et al., 2016b; Mammoto T. et al., 2018; Mammoto T. et al., 2019). Briefly, we added thrombin (2.5 U/ml) and isolated exosomes (4 μg) to the fibrinogen solution (12.5 mg/ml), mixed well, and incubated drops of the mixture (total 30 μl) at 37°C for 30 min until they solidified (Mammoto et al., 2016b; Mammoto T. et al., 2018; Mammoto T. et al., 2019). For gel implantation on the mouse lungs (Mammoto et al., 2016b; Mammoto T. et al., 2018; Mammoto T. et al., 2019), C57BL6J mice were mechanically ventilated and thoracotomy was performed in the fifth left intercostal space. After thoracotomy, a small area of the left visceral pleura (0.5 mm^2^) was scraped using forceps and the fabricated fibrin gel was implanted on the mouse lung surface using a fibrin glue. We implanted the gel on the mouse lungs for 7 days. To examine the effects of pressure and YAP1, we isolated ECs from *Yap1* ^*fl/fl*^ *-Cdh5*(*PAC*)*-Cre* ^*ERT2*^ or *Yap1* ^*fl/fl*^ mouse lungs after tamoxifen induction (Mammoto T. et al., 2019), exposed ECs to pressure (40 mmHg), collected exosomes, mixed exosomes into the fibrin gel, and implanted the gel to the mouse lungs (Mammoto et al., 2016b; Mammoto T. et al., 2018; Mammoto T. et al., 2019). *Yap1* ^*fl/fl*^ mice were obtained from Dr. Fernando Camargo (Harvard Medical School) (Schlegelmilch et al., 2011) and crossed with *Cdh5(PAC)-Cre* ^*ERT2*^ mice (obtained from Dr. Ralf Adams, Max Planck Institute) (Wang et al., 2010), an inducible cre deleter under the control of VE-cadherin promoter, to create VEcadherin-specific Yap1 conditional knockout (*Yap1* ^*fl/fl*^ *-Cdh5*(*PAC*)*-Cre* ^*ERT2*^) mice, in which cre recombination is induced in ECs by administration of tamoxifen (125 μg/mouse, 5 days) (Mammoto T. et al., 2019). Angiogenesis is evaluated by density of blood vessels that are stained positive for EC marker (CD31) from five different areas of the gel (Mammoto et al., 2016b; Mammoto T. et al., 2018; Mammoto T. et al., 2019). To evaluate the connection of newly formed blood vessels in the gel to host vasculature, rhodamine-labeled concanavalinA (conA) (Vector Laboratories, Burlingame, CA) was intravenously injected to the host mouse (Mammoto et al., 2009). Barrier function of newly formed blood vessels in the gel was examined by measuring leakage of low MW rhodamine-labeled dextran (MW 4000, Sigma) intravenously injected to the host mouse (Mammoto A. et al., 2013). To analyze the lumen formation of the newly formed blood vessels in the gel, a z-stack of optical sections was taken using a confocal microscopy. Intravascular red blood cells carried in the gel implanted on the mouse lung were analyzed in the hematoxylin and eosin (H and E) stained histological sections (Mammoto T. et al., 2019). To track the exosomes in the gel, exosomes were labeled with ExoGlow-Protein EV labeling kit (System Biosciences, Palo Alto, CA) according to the manufacturer’s instruction and mixed in the gel. Fluorescent images were taken on a Nikon A1 confocal imaging system and morphometric analysis was performed using ImageJ software as we reported (Mammoto et al., 2012; Mammoto et al., 2016a; Mammoto et al., 2016b; Mammoto A. et al., 2018; Mammoto T. et al., 2018; Mammoto T. et al., 2019).

### Statistical Analysis

All phenotypic analysis was performed by masked observers unaware of the identity of experimental groups. Error bars (SEM) and *p* values were determined from the results of three or more independent experiments. Student’s t-test was used for statistical significance for two groups. For more than two groups, one-way ANOVA with a post-hoc analysis using the Bonferroni test was conducted.

## Results

### Pulmonary Artery Pressure Increases After Pneumonectomy

**FIGURE 1:**
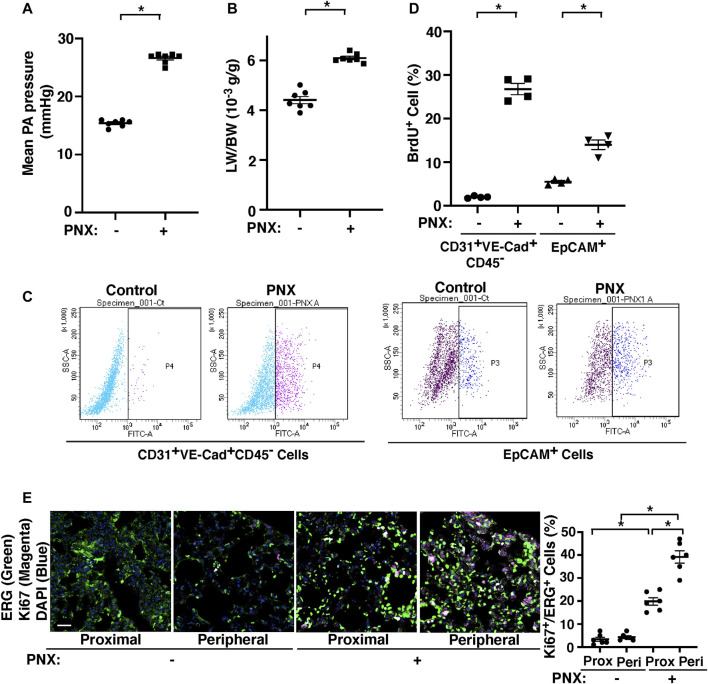
PA pressure increases after left PNX. **(A)** Graph showing the mean PA pressure 7 days after PNX in CD1 mice (n = 7, mean ± s.e.m., *p < 0.05). **(B)** Graph showing the ratio of the weight of right lung lobes (LW) to mouse BW 7 days after left PNX in CD1 mice (n = 7, mean ± s.e.m., *p < 0.05). **(C)** Representative FACS plots showing BrdU-positive CD31^+^VE-cadherin^+^CD45^−^ cells (fraction P4) and EpCAM^+^ cells (fraction P3) 7 days after left PNX in CD1 mice. **(D)** Graph showing BrdU-positive CD31^+^VE-cadherin^+^CD45^−^ cells and EpCAM^+^ cells analyzed using FACS 7 days after left PNX in CD1 mice (n = 4, mean ± s.e.m., *p < 0.05). **(E)** Immunofluorescence (IF) micrographs showing staining of ki67 (magenta), ERG (green) and DAPI (blue) in the proximal or peripheral region of post-PNX or sham-operated mouse lungs. Scale bar, 50 μm. Graph showing the percentage of ki67-positive ERG^+^ cells in the post-PNX or sham-operated mouse lungs (n = 6, mean ± s.e.m., *p < 0.05).

Consistent with others’ report using dogs (Dane et al., 2013; Dane et al., 2016), when we measured the proximal PA pressure by PA cannulation under thoracotomy in mice (Mammoto T. et al., 2018), right PA pressure increased by 1.8-fold following left PNX (Figure 1A), in which lung size (not shown) (Mammoto et al., 2016a) and the ratio of the weight of right lobes to mouse body weight (BW) increased by 1.3-fold (Figure 1B) (Mammoto et al., 2016a; Mammoto T. et al., 2019). The levels of BrdU-positive CD31^+^ VE-cadherin^+^ CD45^−^ ECs and EpCAM^+^ epithelial cells measured by FACS were also 16.6- and 2.3- times higher in the post-PNX lungs compared to those in the sham-operated mice (Figures 1C,D). We also conducted immunohistochemical analysis of ki67^+^ ERG^+^ ECs and ki67^+^SPB^+^ alveolar epithelial type 2 (AT2) cells, which differentiate into AT1 cells and contribute to neoalveolarization (Liu et al., 2016; Lechner et al., 2017; Kobayashi et al., 2020), at the proximal and peripheral regions of the lungs and examined where ECs and epithelial cells proliferate in the lungs after PNX. The number of ki67^+^ proliferative ECs and AT2 cells were significantly higher in the post-PNX mouse lungs (Figure 1E, Supplementary Figure S1A,B). Regarding the region of proliferation, ki67^+^ proliferative ECs and AT2 cells significantly increased in the peripheral region of the lungs compared to proximal region after PNX (Figure 1E, Supplementary Figure S1A,B).

**FIGURE 2:**
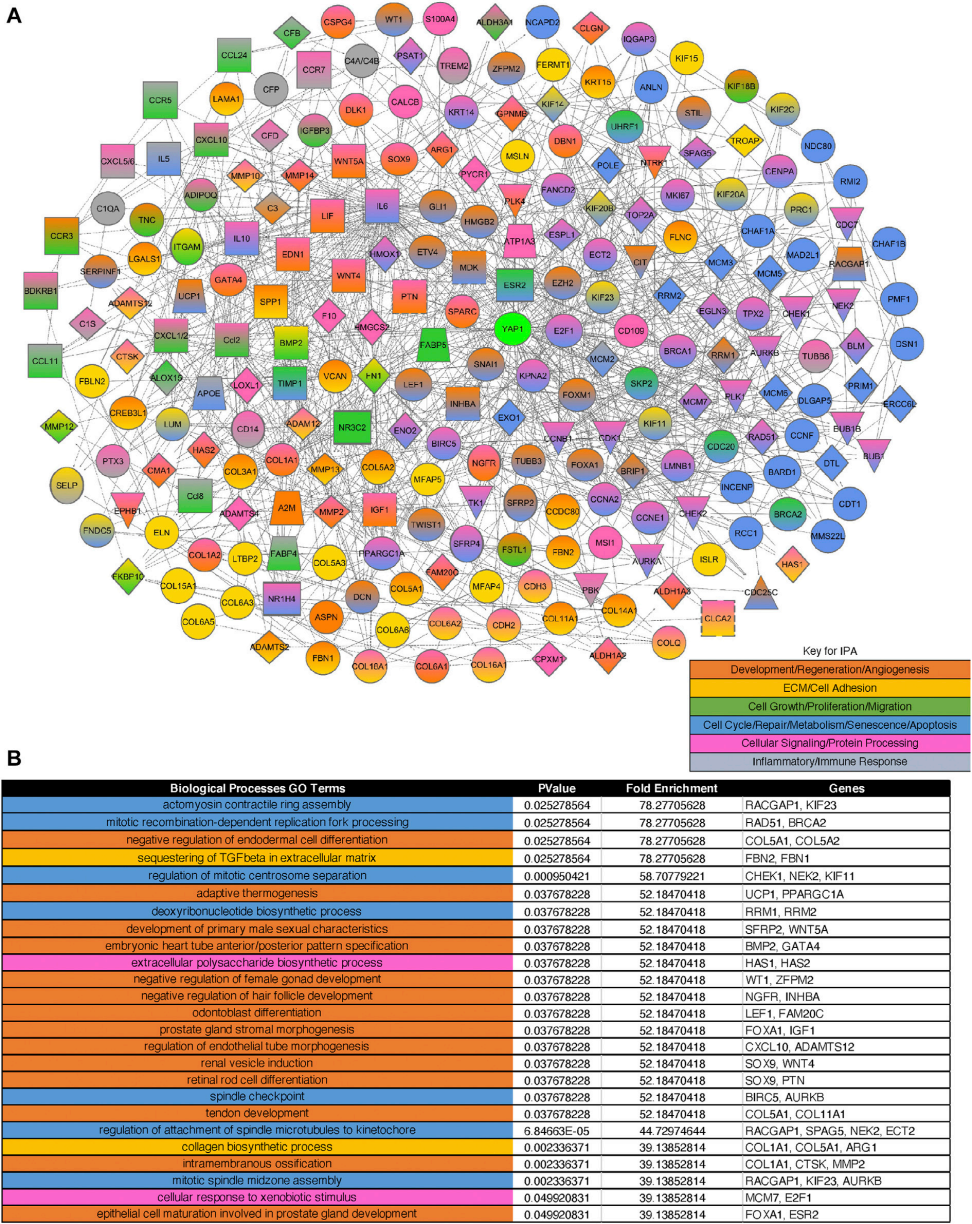
Gene expression profiles and networks in ECs isolated from post-PNX mouse lungs. **(A)** IPA network analysis of three rounds of interactions between the 233 mechanosensitive significantly differentially expressed genes and Yap1. Orange: Development, regeneration, and angiogenesis. Gold: ECM and cell adhesion. Dark green: Cell growth, proliferation, and migration. Blue: Cell cycle, repair, metabolism, senescence, and apoptosis. Pink: Cellular signaling and protein processing. Grey: Inflammatory and immune responses. **(B)** Top 25 BP GO Terms table derived from the 233 significantly differentially expressed mechanosensitive genes appearing in the IPA network. The color-coding corresponds to the network color key.

To identify critical changes in the mechanobiology-related gene expression after PNX, we conducted unbiased RNA sequencing analysis. RNAseq analysis revealed that a total of 17,328 genes were determined to alter between ECs isolated from the post-PNX mouse lungs (7 days) and those from control sham-operated mouse lungs. Differential gene expression analysis revealed that 831 upregulated and 180 downregulated significantly differentially expressed genes met the criteria of a log2 fold change >1 or < -1 and p-adjusted value <0.01 (Supplementary Table S3, Supplementary Figure S2) and generated 345 BP GO Terms categories (Supplementary Table S4). Of these genes, 522 significantly differentially expressed genes (460 upregulated and 62 downregulated) were identified as mechanosensitive genes appeared on a master list comprised of Gene Card and BP GO Term categories relating to ECM, cell-cell junctions, the Hippo pathway, and cellular responses to mechanical forces including shear stress, tension, pressure, and stiffness/elasticity; these mechanosensitive genes appeared in 416 BP GO categories (Supplementary Table S5). IPA network analysis demonstrated that among these 522 mechanosensitive significantly differentially expressed genes, 233 genes (220 upregulated, 13 downregulated; heatmaps of top 20 upregulated and 13 downregulated genes in Supplementary Figure S3A), which produced 371 BP Go Term categories color-coded into groups encompassing development, regeneration, and angiogenesis; ECM/cell adhesion; cell growth, proliferation, and migration; cell cycle, repair, metabolism, senescence, and apoptosis; cellular signaling and protein processing; and inflammatory and immune responses (Supplementary Table S6, the top 25 of BP GO Terms in Figure 2B, the heatmap of the genes in top 25 of BP GO Terms in Supplementary Figure S3B), interact closely with Yap1 (Figure 2A).

### Pressurization of Endothelial Cells Stimulates YAP1 and Tie2 Expression and Angiogenic Activity *in vitro*

**FIGURE 3:**
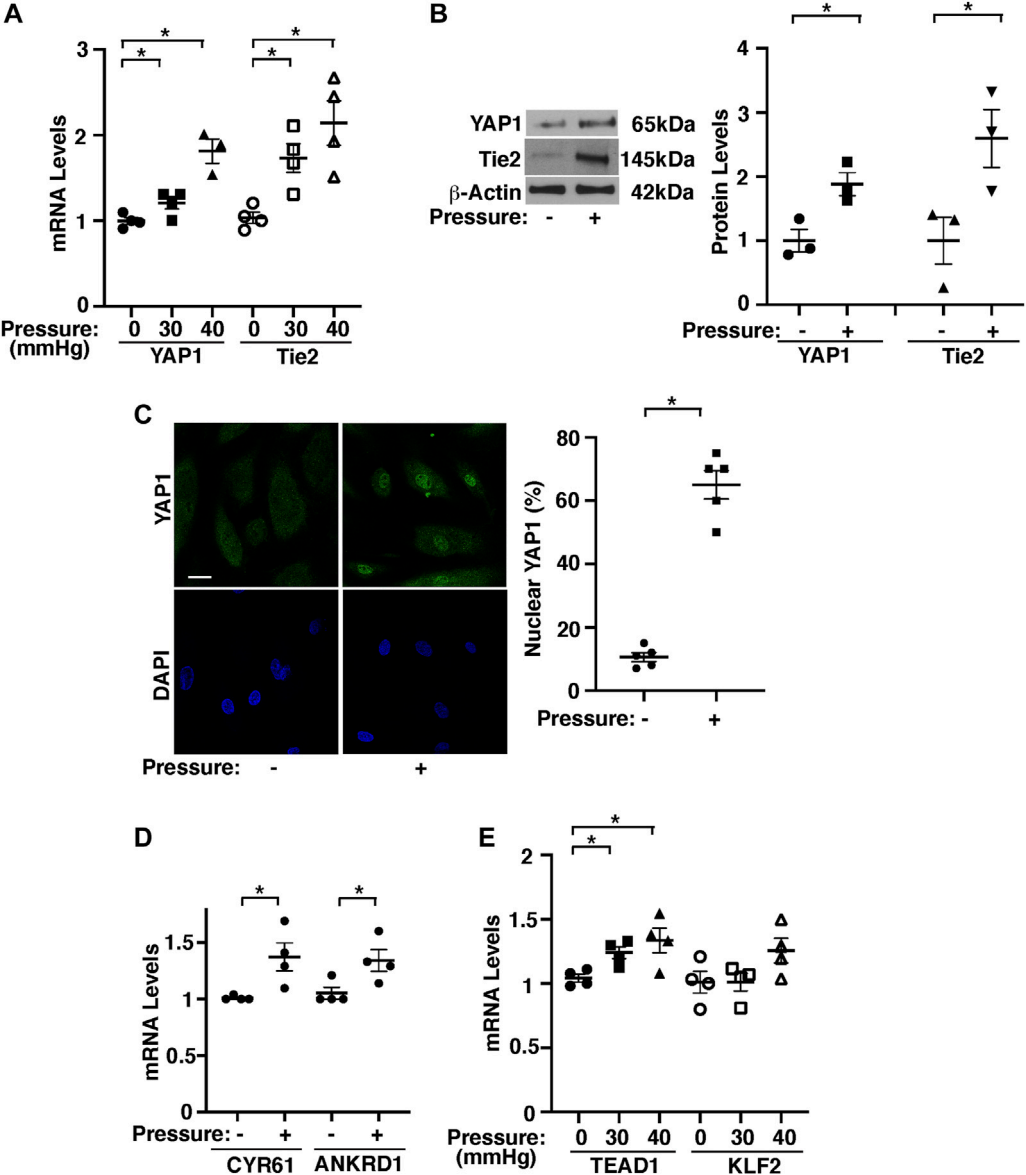
Hydrostatic pressure controls YAP1-TEAD1 signaling in HPAECs. **(A)** Graph showing YAP1 and Tie2 mRNA levels in HPAECs exposed to pressure (30, 40 mmHg, n = 3-4, mean ± s.e.m., *p < 0.05). **(B)** Representative immunoblots showing YAP1, Tie2, and β-actin protein levels in HPAECs exposed to hydrostatic pressure (*left*, 40 mmHg). Graph showing the quantification of immunoblots (*right*, n = 3, mean ± s.e.m., *p < 0.05). **(C)** IF micrographs showing nuclear localization of YAP1 in pressurized HPAECs (*left*, 40 mmHg). Scale bar, 10 μm. Graph showing nuclear localization of YAP1 (*right*, 40 mmHg, n = 5, mean ± s.e.m., *p < 0.05). **(D)** Graph showing the mRNA levels of CYR61 and ANKRD1 in HPAECs exposed to pressure (40 mmHg, n = 4, mean ± s.e.m., *p < 0.05). **(E)** Graph showing TEAD1 and KLF2 mRNA levels in HPAECs exposed to pressure (30, 40 mmHg, n = 4, mean ± s.e.m., *p < 0.05).

To study the effects of pressurization of ECs on angiogenesis, we exposed HPAECs to elevated hydrostatic pressure (30–40 mmHg) using a pressure chamber for 16 h. Since human PA pressure is 15–20 mmHg and PA pressure increases by 1.8–2.0 times in an *in vivo* mouse PNX model, we pressurized HPAECs with the range of 0–40 mmHg. There was no significant difference in the expression of YAP1 and Tie2 in ECs exposed to 15 mmHg compared to that in nonpressurized ECs (Supplementary Figure S1C), and therefore we used the nonpressurized condition as a control. The mRNA and protein levels of YAP1 and Tie2 were 1.8- and 2.1- times and 1.8- and 2.4-times higher, respectively, in HPAECs under pressure (40 mmHg) compared to those in the nonpressurized condition (Figure 3A,B). Similar trends were observed in HPAECs under 30 mmHg pressure (Figure 3A). YAP1 was also localized in the nucleus 5.6- times more in pressurized HPAECs when analyzed using immunocytochemistry (40 mmHg, Figure 3C). The mRNA expression of YAP1 target genes, CYR61 and ANKRD1 was also 1.4- and 1.3-times higher, respectively, in pressurized HPAECs (Figure 3D). Pressurization also significantly increased the levels of TEAD1, which binds to YAP1 and controls Tie2 expression (Mammoto A. et al., 2018; Mammoto T. et al., 2019) (Figure 3E).

**FIGURE 4:**
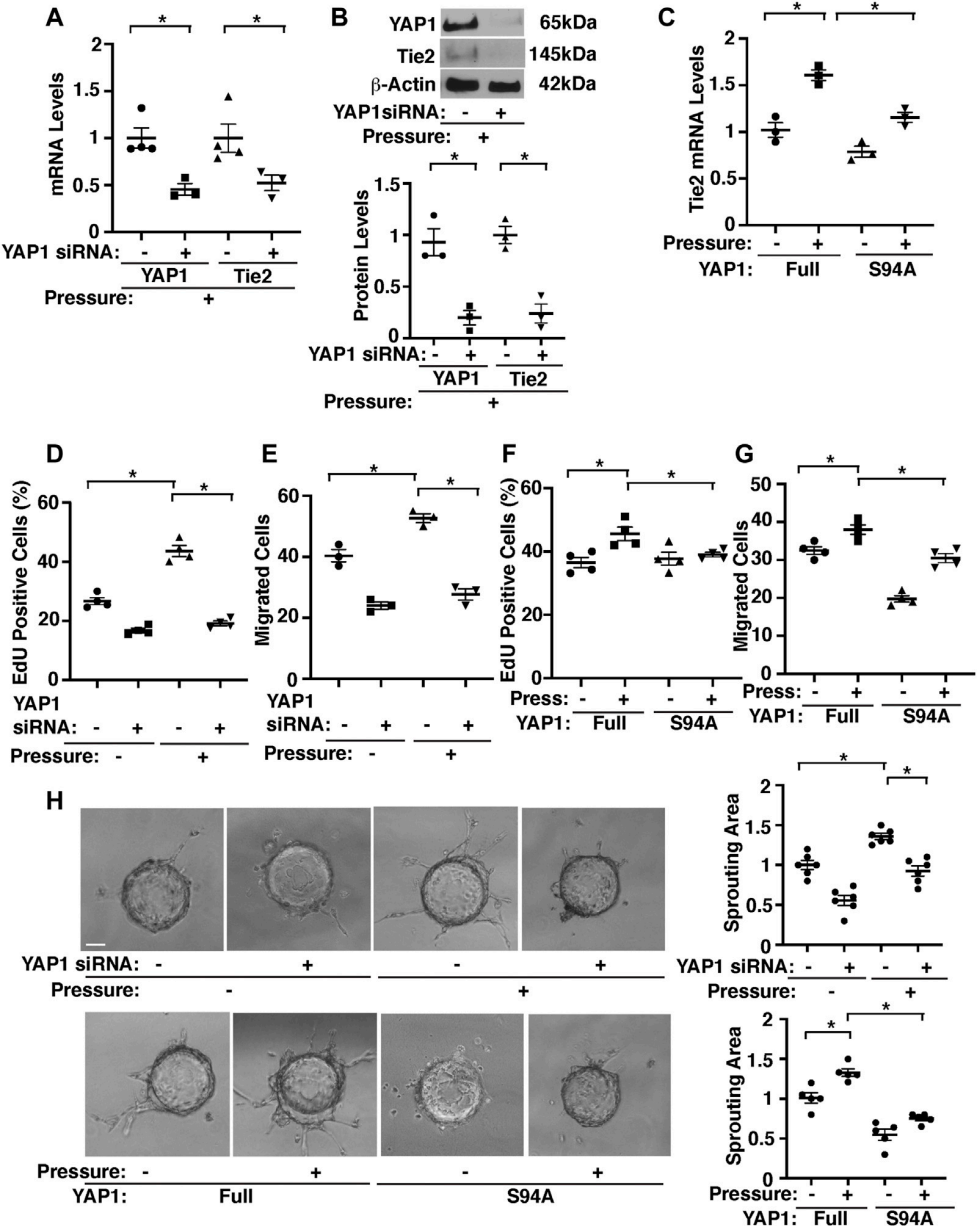
Hydrostatic pressure controls angiogenic activity through YAP1-TEAD1 signaling in HPAECs. **(A)** Graph showing the mRNA levels of YAP1 and Tie2 in HPAECs treated with YAP1 siRNA or control siRNA and exposed to pressure (40 mmHg, n = 3-4, mean ± s.e.m., *p < 0.05). **(B)** Representative immunoblots showing YAP1, Tie2, and β-actin protein levels in HPAECs treated with YAP1 siRNA or control siRNA with irrelevant sequences and exposed to hydrostatic pressure (*top*, 40 mmHg). Graph showing the quantification of immunoblots (*bottom*, n = 3, mean ± s.e.m., *p < 0.05). **(C)** Graph showing the mRNA levels of Tie2 in HPAECs treated with YAP1 full length (Full) or YAP1S94A mutant construct and exposed to pressure (40 mmHg, n = 3, mean ± s.e.m., *p < 0.05). **(D)** Graph showing the percentage of EdU-positive HPAECs treated with YAP1 siRNA or control siRNA and exposed to pressure (40 mmHg, n = 4, mean ± s.e.m., *p < 0.05). **(E)** Graph showing the migration of HPAECs treated with YAP1 siRNA or control siRNA and exposed to pressure (40 mmHg, n = 3, mean ± s.e.m., *p < 0.05). **F)** Graph showing the percentage of EdU-positive HPAECs treated with YAP1 Full or YAP1S94A mutant construct and exposed to pressure (40 mmHg, n = 4, mean ± s.e.m., *p < 0.05). **(G)** Graph showing the migration of HPAECs treated with YAP1 Full or YAP1S94A mutant construct and exposed to pressure (40 mmHg, n = 4, mean ± s.e.m., *p < 0.05). **(H)** Phase contrast images showing EC sprouting from each bead in HPAECs treated with Ang1 or in combination with YAP1 siRNA, lentivirus (YAP1 Full or YAP1S94A mutant construct), or control siRNA with irrelevant sequences and exposed to pressure (40 mmHg). Scale bar, 20 μm. Graphs showing changes in sprout area in HPAECs treated with Ang1 or in combination with YAP1 siRNA, lentivirus (YAP1 Full or YAP1S94A mutant construct), or control siRNA and exposed to pressure (40 mmHg) (n = 5-6, mean ± s.e.m., *p < 0.05).

Knockdown of YAP1 using siRNA transfection decreased Tie2 mRNA and protein expression by 49 and 82%, respectively in HPAECs under pressure (40 mmHg) compared to those treated with control siRNA that does not inhibit YAP1 expression (Figures 4A,B). Consistently, YAP1S94A mutant construct, which inhibits YAP1 and TEAD1 interaction (Mammoto A. et al., 2018), inhibited the Tie2 expression in HPAECs under pressure (40 mmHg, Figure 4C), suggesting that YAP1-TEAD1 interaction is necessary for Tie2 expression in HPAECs under pressure.

Pressurization of HPAECs treated with control siRNA (40 mmHg, 16 h) also significantly stimulated DNA synthesis and migration analyzed by an EdU assay and a transwell migration assay, respectively; EdU-positive ECs and EC migration towards a Tie2 ligand, Ang1 increased by 1.7- times and 1.3-times, respectively, while siRNA-based knockdown of YAP1 inhibited the effects (Figures 4D,E). YAP1S94A mutant construct also inhibited the DNA synthesis and migration under pressure (Figures 4F,G).

To examine whether pressurization of HPAECs controls blood vessel formation through YAP1 signaling *in vitro*, we performed a three-dimensional (3D) EC sprouting assay, in which microbeads coated with HPAECs were cultured in the fibrin gel for 5 days and sprouting from the beads was quantified (Mammoto et al., 2016a; Mammoto et al., 2016b; Mammoto T. et al., 2019). Consistent with DNA synthesis and migration, pressurization of HPAECs treated with control siRNA (40 mmHg, 16 h) stimulated EC sprouting; sprouting area increased by 1.3-times, while siRNA-based knockdown of YAP1 inhibited the effects (Figure 4H). YAP1S94A mutant construct also inhibited EC sprouting under pressure (Figure 4H). These results suggest that YAP1-TEAD1 signaling mediates the effects of EC pressurization on angiogenic activity.

### Exosomes From Pressurized Endothelial Cells Stimulate Angiogenesis in the Fibrin Gel Implanted on the Mouse Lungs

**FIGURE 5:**
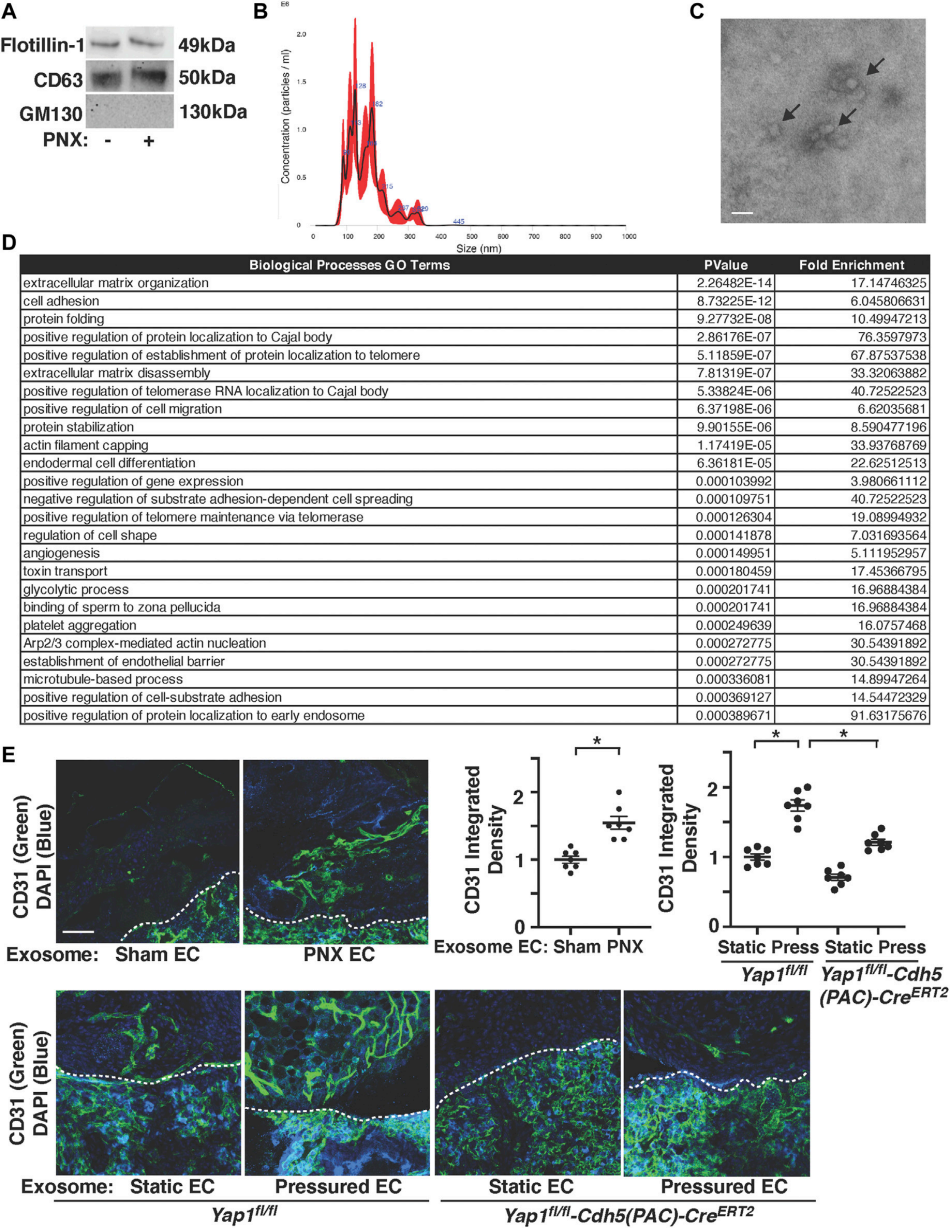
Post-PNX mouse lung EC exosomes stimulate vascular formation in the gel implanted on the mouse lungs. **(A)** IB analysis of Flotillin-1, CD63, and GM130 in exosomes collected from conditioned media of ECs isolated from post-PNX mouse lungs or sham-operated mouse lungs. **(B)** Size distribution and particle concentration of isolated exosomes analyzed using NTA. **(C)** TEM image of exosome morphology. Scale bar: 150 nm. Arrows indicate exosomes. **(D)** Top 25 Biological Processes GO Term categories by *p*-value derived from the 152 differentially expressed genes identified in PNX vs sham-operated mouse EC exosome proteomics analysis. **(E)** Vascular formation in the implanted fibrin gel supplemented with exosomes isolated from conditioned media of post-PNX or sham-operated mouse lung ECs (*top*). Vascular formation in the implanted fibrin gel supplemented with exosomes isolated from conditioned media of unpressured or pressured *Yap1* ^*fl/fl*^ *-Cdh5*(*PAC*)*-Cre* ^*ERT2*^ or *Yap1* ^*fl/fl*^ mouse lung ECs (*bottom*). Scale bar: 50 μm. Dashed lines indicate the interface between implanted fibrin gel and host lung. Graphs showing quantification of vessel density (n = 7, mean ± s.e.m., *p < 0.05).

Exosomes promote angiogenesis and stimulate tissue regeneration (Ibrahim et al., 2014; Shanmuganathan et al., 2018). When exosomes were isolated from pre-filtered (0.2 μm) conditioned media of ECs (1 × 10^6^ cells) isolated from post-PNX or sham-operated C57BL6J mouse lungs using total exosome isolation reagent (Gartz et al., 2018; Doyle and Wang, 2019; Gartz et al., 2020), the isolated exosome population was positive for established exosome markers (CD63, Flotillin-1) and negative for the cellular marker GM130 when analyzed using IB (Figure 5A). Nanoparticle tracking analysis (NTA) revealed that isolated EC exosomes were heterogenous in diameter with 90–180 nm (Figure 5B). Transmission electron microscopy (TEM) exhibited the round vesicular looking morphology with approximately 80–120 nm in size (Figure 5C) (Hung and Leonard, 2015; Ludwig et al., 2017; Gartz et al., 2018; Lee et al., 2018; Zhang et al., 2018).

Proteomics analysis of exosomes isolated from conditioned media of two sham-operated vs three post-PNX (7 days) mouse lung EC replicates identified 228 proteins present in at least one of both the control and PNX sample replicates. A cutoff threshold of greater than or equal to eight Total Spectrum Counts in at least one overall replicate further narrowed the list to 152 proteins of interest (Supplementary Table S2). Consistent with RNAseq results, the top 25 Biological Processes GO Term categories derived from the differentially expressed genes associated with the 152 proteins ranged from extracellular matrix organization, cell adhesion, actin cytoskeleton remodeling to protein localization, stabilization, and folding among other functions (Figure 5D).

To examine the effects of exosomes isolated from post-PNX mouse lung ECs on blood vessel formation, we implanted the fibrin gel containing exosomes on the mouse lungs. First, we tracked exosomes in the gel and examined how exosomes are incorporated into the fibrin gel. When we labeled exosome cargos, mixed labeled exosomes into the gel, and implanted on the mouse lungs, the labeled exosomes were in the gel just after implantation (day 0). Exosomes remained in the gel 3 days after implantation, in which most of the exosomes attached to the surface of the recruited cells and/or were internalized into the cells. The number of exosomes in the gel decreased 7 days after implantation (Supplementary Figure S4A). Recruitment of host mouse ECs and vascular formation were significantly stimulated in the gel containing exosomes isolated from conditioned media of post-PNX (7 days) mouse lung ECs compared to that supplemented with exosomes from sham-operated mouse lung ECs (Figure 5E). To examine the effects of pressure and YAP1, we isolated ECs from *Yap1* ^*fl/fl*^ *-Cdh5*(*PAC*)*-Cre* ^*ERT2*^ or *Yap1* ^*fl/fl*^ mouse lungs after tamoxifen induction (Mammoto T. et al., 2019), exposed ECs to pressure (40 mmHg), collected exosomes, mixed exosomes into the fibrin gel, and implanted the gel on the mouse lungs (Mammoto et al., 2016b; Mammoto A. et al., 2018; Mammoto T. et al., 2019). Exosomes isolated from conditioned media of pressurized *Yap1* ^*fl/fl*^ mouse lung ECs promoted recruitment of host mouse ECs and vascular formation in the gel compared to those supplemented with exosomes from unpressurized ECs (Figure 5E). Exosomes from *Yap1* ^*fl/fl*^ *-Cdh5*(*PAC*)*-Cre* ^*ERT2*^ mouse lung ECs inhibited the effects (Figure 5E), suggesting that endothelial YAP1 is necessary for isolated exosomes to induce vascular formation in the gel. It is important to note that the newly formed blood vessels in the gel containing exosomes from pressurized ECs 1) connected to host vasculature; intravenous injection of rhodamine labeled concanavalinA (conA) to the host mouse labeled the newly formed blood vessels in the gel containing exosomes from pressured ECs (Supplementary Figure S4B), 2) had lumens; 3D reconstruction and z-stack of confocal microscopy images reveal that there are lumen structures in the newly formed vasculatures (Supplementary Figure S4C), 3) had sufficient barrier function; low MW rhodamine labeled dextran (MW 4000) intravenously injected to the mouse did not leak out of newly formed blood vessels in the gel (Supplementary Figure S4D), and 4) carried red blood cells; H&E-stained image shows that red blood cells were carried in the gel containing exosomes implanted on the mouse lungs (Supplementary Figure S4E), suggesting that EC-derived exosomes induce functional vascular formation in the gel.

## Discussion

In this report, we demonstrate that hydrostatic pressure, which significantly increases after unilateral PNX, stimulates angiogenic activities through endothelial YAP1 signaling. We also found that exosomes isolated from conditioned media of post-PNX lung ECs or pressurized ECs stimulate blood vessel formation in the fibrin gel implanted on the mouse lungs (Supplementary Figure S5). Gene enrichment analysis confirms that mechanosensitive genes altered in post-PNX mouse lung ECs interact with YAP1. Modulation of either the mechanical environment or endothelial YAP1 signaling can pave the way for the development of more efficient strategies for lung regeneration.

Our RNAseq and proteomics results demonstrate that the levels of the genes related to ECM, cell adhesion, organ development and regeneration are significantly altered after PNX (Figure 2B, Figure 5D, Supplementary Table S6). YAP1 is a mechanosensitive gene and mediates post-PNX lung growth (Mammoto T. et al., 2019). In fact, the genes listed in the BP GO terms of mechanosensitive cellular responses interact with YAP1 (Figure 2A). Other Hippo molecules such as AMOT, which mediates shear-induced YAP1 activation in a zebrafish model (Nakajima et al., 2017), WW domain containing transcription regulator 1 (WWTR1, TAZ) and LATS, the activity of which is controlled by actin cytoskeleton (Zhao et al., 2010; Piccolo et al., 2013; Panciera et al., 2017), mammalian STE20-like (MST) 1/2, Merlin and Expanded, which are responsible for membrane and actin cytoskeleton association, are known to act upstream of the Hippo pathway (Hamaratoglu et al., 2006) and may also mediate the mechanical force-dependent lung growth after PNX. It has been reported that YAP1 activity is controlled by Wnt signaling and vice versa (Azzolin et al., 2014) and that Wnt co-receptor, LDL receptor related protein5 (LRP5) controls Tie2 expression in ECs and modulates lung development and regeneration (Mammoto et al., 2012; Mammoto T. et al., 2013; Mammoto et al., 2016a). Given that ECM-stiffness controls postnatal lung development through LRP5-Tie2 signaling (Mammoto T. et al., 2013), changes in the mechanical forces after PNX may control YAP1 activity through LRP5 as well. YAP1 also regulates expression of another mechanosensitive transcription factor, Twist1 (Farge, 2003; Zhang et al., 2014), which also controls post-PNX lung growth (Hendee et al., 2021) and may be involved in the mechanism. In fact, Twist1 interacts with YAP1 in the network derived from significantly differentially expressed genes after PNX (Figure 2A).

While endothelial YAP1 mediates post-PNX lung growth and PA-pressure-induced EC proliferation and migration, YAP1 is not significantly differentially expressed in post-PNX lung ECs in our bulk RNAseq data. This may be because; 1) YAP1 is phosphorylated at multiple sites, which controls nuclear translocation of YAP1 and its co-transcriptional activity (Szulzewsky et al., 2021). For example, YAP1S127 phosphorylation sequesters YAP1 to the cytoplasm and suppresses YAP1 gene transcriptional activity (Ota and Sasaki, 2008; Piccolo et al., 2014). In fact, there was significant increase in YAP1 nuclear expression co-stained with ERG in the post-PNX mouse lungs, suggesting that YAP1 activity is significantly increased in ECs after PNX (Supplementary Figure S1D). This is consistent with the *in vitro* results using cells under pressure (Figure 3C). In addition, 2) it has been reported that there is a vast heterogeneity of EC populations in the lung (Gillich et al., 2020). 3) The levels of mechanical forces altered following PNX may also be different at different portions of the lungs; while PA pressure is significantly increased after PNX, the increase will be less at the capillary level and negligible in the veins. On the contrary, changes in other mechanical forces such as stretching forces following PNX are more prominent at the peripheral region of the lungs, where ECs and AT2 cells are proliferating more compared to proximal region (Figure 1E, Supplementary Figure S1A,B). In our immunohistochemical analysis, there were YAP1-negative ERG-positive ECs in the post-PNX lungs, especially in the proximal region (Supplementary Figure S1D). This spatial and EC subpopulation-dependent difference in YAP1 expression and the post-translational modification may explain why Yap1 is not detected as a significantly differentially expressed gene in our RNAseq results, while it mediates pressure-dependent angiogenic factor expression and EC behaviors *in vitro*. Different EC subpopulations (e.g., PA, capillary, vein) in the lungs may sense different types of mechanical forces in a spatiotemporal manner during post-PNX lung growth. These spatial differences in the different types of mechanical forces altered after PNX may differentially alter YAP1 expression depending on EC subpopulations and regions of the lungs, which cannot be accurately profiled by bulk RNAseq. Future investigation using single cell RNAseq or bulk RNAseq with specific EC subpopulations enriching for pulmonary artery EC markers (Gja5+ Bmx+) (Manning et al., 2021) would further elucidate the mechanism of pressure-induced angiogenesis during post-PNX lung growth.

Increases in flow change shear stress in the lung after PNX. However, although hydrostatic pressure increased TEAD1 and Tie2 expression, it did not change the levels of a major shear stress-induced transcription factor, KLF2 (Figure 3E). The distinct signaling pathways may mediate the mechanism in a cooperative way. Spatiotemporal controls of multiple gene expression lead to the fine vascular and alveolar morphogenesis during lung regeneration. It remains unknown whether TEAD1 directly regulates Tie2 transcription and how the mechanical environment altered after PNX controls the mechanism. Given that Tie2 promoter contains M-CAT-like TEAD1 binding motif, TEAD1 may directly control Tie2 transcription. Further investigation including ChIPseq will uncover the transcriptional mechanism.

Increases of PA pressure after PNX may only be transient and restored at the later stage; however, these transient changes in PA pressure may trigger the mechanosensitive gene expression and activity. Pressure-independent effects, such as indirect paracrine effects, neurohormonal effects or changes in oxygen concentration may change YAP1 activity and also be involved in the mechanism. It has been demonstrated that various types of EC progenitor cells are involved in lung development and regeneration (Ren et al., 2019; Gillich et al., 2020; Niethamer et al., 2020). The response to pressure may be different among the EC populations and the portions of the lungs where specific EC progenitors are localized, as well as during the time course of post-PNX lung growth. Further study using EC lineage tracing will be necessary to elucidate the entire mechanism.

We focus on the effects of PA pressure on endothelial YAP1 expression/activity and angiogenic activities. Other lung cells (e.g., epithelial cells, smooth muscle cells, immune cells) (Stevens et al., 2008) may interact with ECs and contribute to post-PNX lung growth. In fact, both CD31^+^ VE-Cad^+^ CD45^−^ ECs and EpCAM^+^ epithelial cells proliferate after PNX (Figures 1C,D) and YAP1 expresses in alveolar epithelial cells and contributes to lung regeneration (Liu et al., 2016). These other cells may also sense changes in mechanical environment after PNX and secrete angiogenic and other chemical factors, and indirectly control vascular formation after PNX in a spatiotemporal manner. It has been reported that after PNX, 1) AT2 cells, one of the major alveolar epithelial progenitor cells, proliferate (5–7 days after PNX) when analyzed using a BrdU incorporation assay (Liu et al., 2016), which is consistent with the IHC analysis of ki67 staining (Supplementary Figure S1A,B); 2) these AT2 cells differentiate into AT1 cells that are specialized for gas exchange when analyzed using a lineage trancing assay (3–4 weeks after PNX) (Liu et al., 2016; Lechner et al., 2017; Kobayashi et al., 2020); and 3) alveolar number increases in the post PNX mouse lungs (Liu et al., 2016; Mammoto T. et al., 2019; Hendee et al., 2021). These results suggest that neoalveolarization takes place in the post-PNX mouse lungs. Further investigation of the effects of PA pressure on post-PNX alveolar formation would delineate the mechanism of post-PNX regenerative lung growth.

We demonstrate that exosomes collected from post-PNX mouse lung ECs or pressured ECs stimulate vascular formation in the gel implanted on the mouse lungs (Figure 5E). It has been reported that exosomes are also in the mouse circulation (Cavallari et al., 2020; Chen et al., 2021). Consistently, when we collected exosomes from sham vs post-PNX mouse serum and examined the effects on angiogenic activity, the post-PNX mouse serum-derived exosomes stimulated EC sprouting *in vitro* (Supplementary Figure S4F). Thus, serum-derived exosomes may also contribute to angiogenesis in the post-PNX mouse lungs. Blood vessels induced by EC-derived exosomes seem to be physiological and functional because blood vessels stimulated by pressured EC-derived exosomes have organized lumen structures and sufficient barrier functions with carrying red blood cells in the implanted gel (Supplementary Figure S4B,C,D,E). Thus, exosomes may have potential to be a better strategy for lung regeneration or repair from injury, in which angiogenesis and mechanical forces are involved. Although we focus on the effects of exosomes on angiogenesis in this study, exosomes may also target other cells (e.g., epithelial cells, immune cells, fibroblasts) in the mouse lungs to control post-PNX lung growth. Our proteomics analysis of exosomes reveals that ECM or actin cytoskeleton remodeling proteins are altered after PNX, which may remodel the fibrin gel structures or mechanics, and control blood vessel formation in the gel. Further analysis of exosomes isolated from different time points, different portions of the lungs, or specific EC populations and their effects not only on ECs but also on other types of cells will elucidate the mechanism.

It is known that exosomes have different fates in the cell; exosomes secreted into the extracellular spaces 1) interact with the surface receptors of recipient cells, 2) fuse with the plasma membrane to release their contents into cytosol, or 3) are internalized into the recipient cells. Internalized exosomes are sorted into late endosomes and the exosome contents are released into the nucleus, endoplasmic reticulum or cytosol. Exosomes are also degraded in the lysosomes or exocytosed again to the extracellular space through the recycling endosomes (Gurung et al., 2021). These exosome fates are determined depending on the contents of exosomes and are different among cell populations/conditions, which will change the number/concentration of exosomes in the gel over time. Thus, even if we mixed the gel with the same protein amount of exosomes, the concentration would be differentially changed, which leads to the changes in vascular morphogenesis in the gel. Further time course investigation of exosome fates, trafficking, and components in different cell types recruited into the gel would uncover the mechanism.

In summary, we have demonstrated that changes in the mechanical environment after PNX such as increased PA pressure control endothelial YAP1 expression and angiogenic activities. Modulation of mechanical environment or endothelial YAP1 signaling may improve the strategies for lung regeneration.

## Data Availability

The datasets presented in this study can be found in online repositories. The names of the repository/repositories and accession number(s) can be found in NCBI GEO GSE154110.

## Supplementary Material

Supplementary Figure 1 Cellular proliferation and YAP1 expression in the post-PNX mouse lungs. **(A)** IF micrographs showing staining of ki67 (magenta), SPB (green) and DAPI (blue) in the proximal or peripheral region of post-PNX or sham-operated mouse lungs. Scale bar, 50 μm. **(B)** Graph showing the percentage of ki67-positive SPB+ cells in the post-PNX or sham-operated mouse lungs (n = 6, mean ± s.e.m., **p* < 0.05). **(C)** Graph showing YAP1 and Tie2 mRNA levels in HPAECs exposed to pressure (15 mmHg, n = 4, mean ± s.e.m.). **(D)** IF micrographs showing staining of YAP1 (magenta), ERG (green) and DAPI (blue) in the proximal or peripheral region of post-PNX or sham-operated mouse lungs (top). Scale bar, 50 μm. Graph showing the percentage of YAP1-positive ERG+ cells in the post-PNX or sham-operated mouse lungs (n = 6, mean ± s.e.m., **p* < 0.05).

Supplementary Figure 2 Top 50 genes significantly differentially expressed in post-PNX mouse lung ECs. The list of top 50 genes significantly differentially expressed in ECs isolated from the post-PNX mouse lungs (7 days) compared to those from control sham-operated mouse lungs analyzed by RNAseq. RNAseq results are filtered for genes with a >2-fold change and an adjusted *p*-value <0.01.

Supplementary Figure 3 Gene expression heatmaps of ECs isolated from post-PNX mouse lungs. **(A)** Heatmaps of the top 20 upregulated and 13 downregulated genes of the 233 significantly differentially expressed mechanosensitive-related genes comprising the IPA network. **(B)** Heatmap of the 46 IPA network genes comprising the top 25 BP GO Term categories listed in Fig. 2B. Genes are color-coded as IPA network. Orange: Development, regeneration, and angiogenesis. Gold: ECM and cell adhesion. Blue: Cell cycle, repair, metabolism, senescence, and apoptosis. Pink: Cellular signaling and protein processing.

Supplementary Figure 4 Pressurized EC exosomes stimulate vascular formation in the gel implanted on the mouse lungs. **(A)** IF micrographs showing the implanted gel containing green fluorescence-labeled exosomes from pressured ECs 0, 3 and 7 days after implantation. Scale bar: 50 μm. **(B)** IF micrograph showing CD31^+^ blood vessels (green) labeled with intravenously injected rhodamine-labeled conA (magenta) in the gel containing exosomes from pressured ECs. Scale bar: 50 μm. Dashed line indicates the interface between implanted fibrin gel and host lung. **(C)** 3D reconstructed IF image of vascular formation in the implanted fibrin gel supplemented with exosomes from pressured ECs (top). Z-stack image of vascular lumen formation in the implanted fibrin gel supplemented with exosomes from pressured ECs (bottom). Scale bar: 20 μm. **(D)** IF micrograph showing CD31^+^ blood vessels (green) and intravenously injected low MW rhodamine-labeled dextran leakage (magenta) in the gel containing exosomes from pressured ECs. Scale bar: 50 μm. Dashed line indicates the interface between implanted fibrin gel and host lung. **(E)** H&E-stained micrographs showing the gel containing exosomes from pressured ECs implanted on the mouse lung that contains red blood cells. Scale bar: 100 μm. Dashed line indicates the interface between implanted fibrin gel and host lung. Arrows in the higher magnification image (right) indicate red blood cells. **(F)** Phase contrast images showing EC sprouting from bead covered with HPAECs treated with exosomes from post-PNX or sham-operated mouse serum. Scale bar, 20 μm. Graph showing changes in sprout area in HPAECs treated with exosomes from post-PNX or sham-operated mouse serum (n = 6, mean ± s.e.m., **p* < 0.05).

Supplementary Figure 5 Schematic illustration of the effects of post-PNX increases in PA pressure on angiogenesis in the lungs. PA pressure increases after PNX. Hydrostatic pressure upregulates the expression of YAP1 and Tie2, and induces EC DNA synthesis, migration, and sprouting *in vitro*. Exosomes from post-PNX mouse lung ECs or pressurized ECs stimulate angiogenesis through YAP1 signaling.
