# Supplementary figures and images for: Hydrostatic Pressure Controls Angiogenesis Through Endothelial YAP1 During Lung Regeneration
Source: Front Bioeng Biotechnol. 2022 Feb 18;10:823642. doi: 10.3389/fbioe.2022.823642 (PMC8896883; doi:10.3389/fbioe.2022.823642)

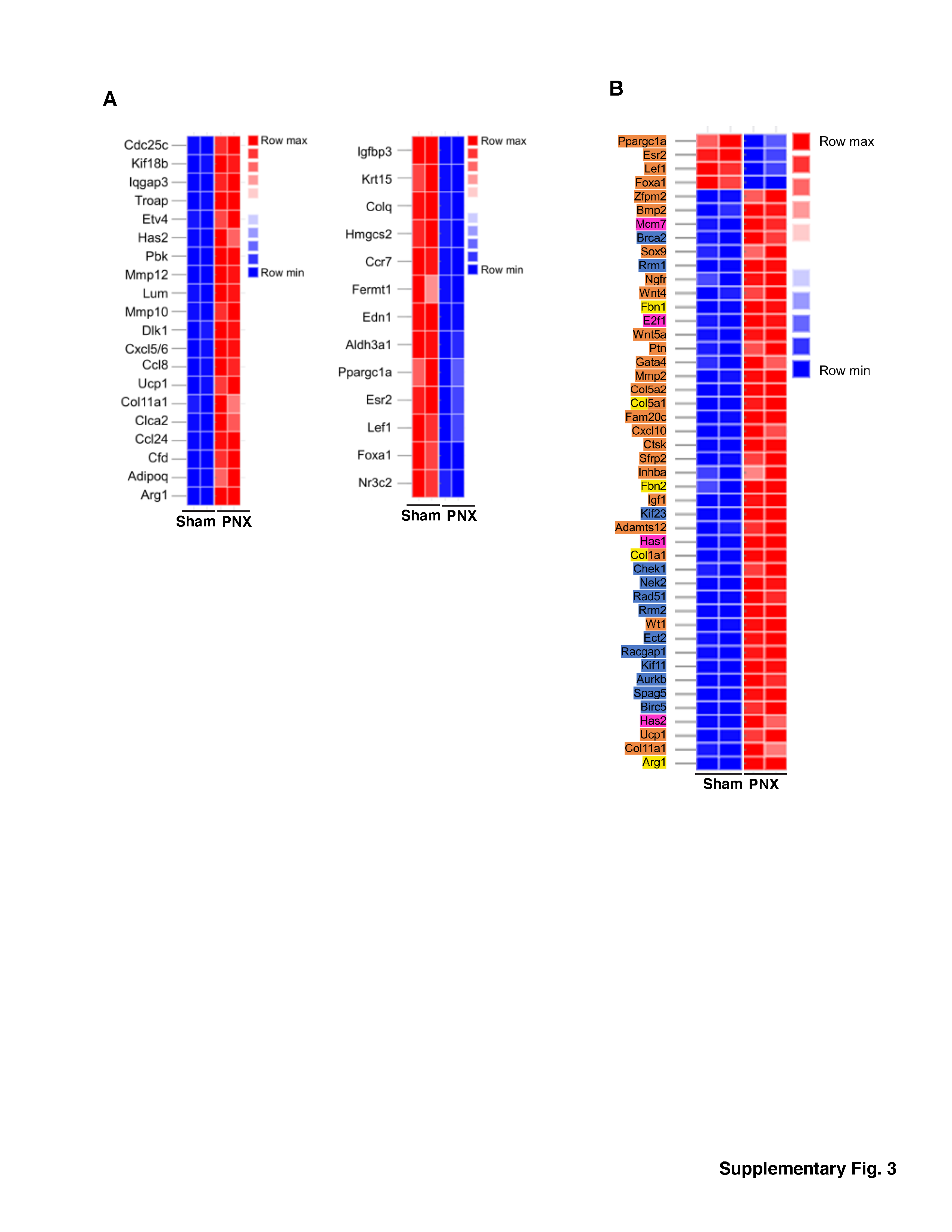

Supplement: Supplementary file 1 [file Image3.TIFF]

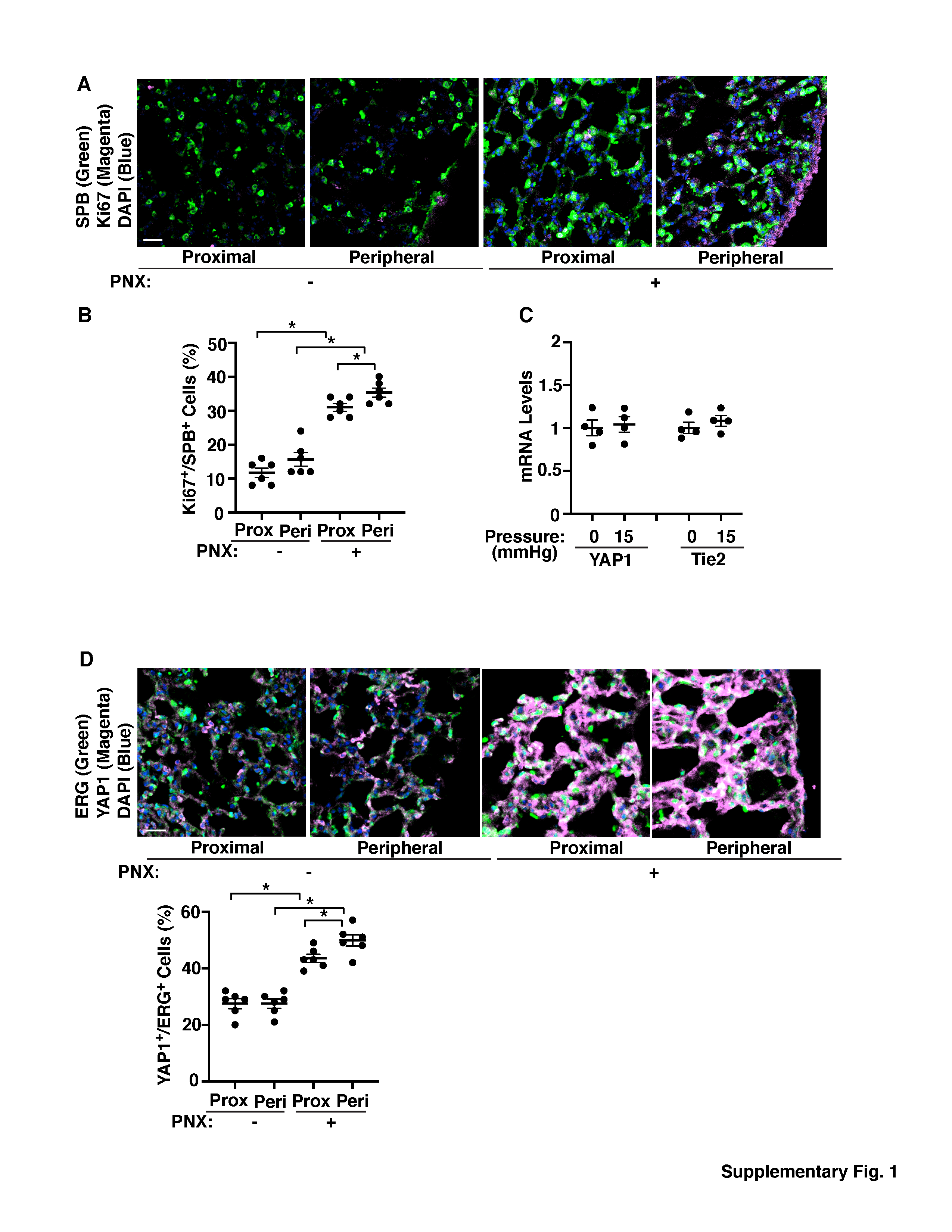

Supplement: Supplementary file 3 [file Image1.TIFF]

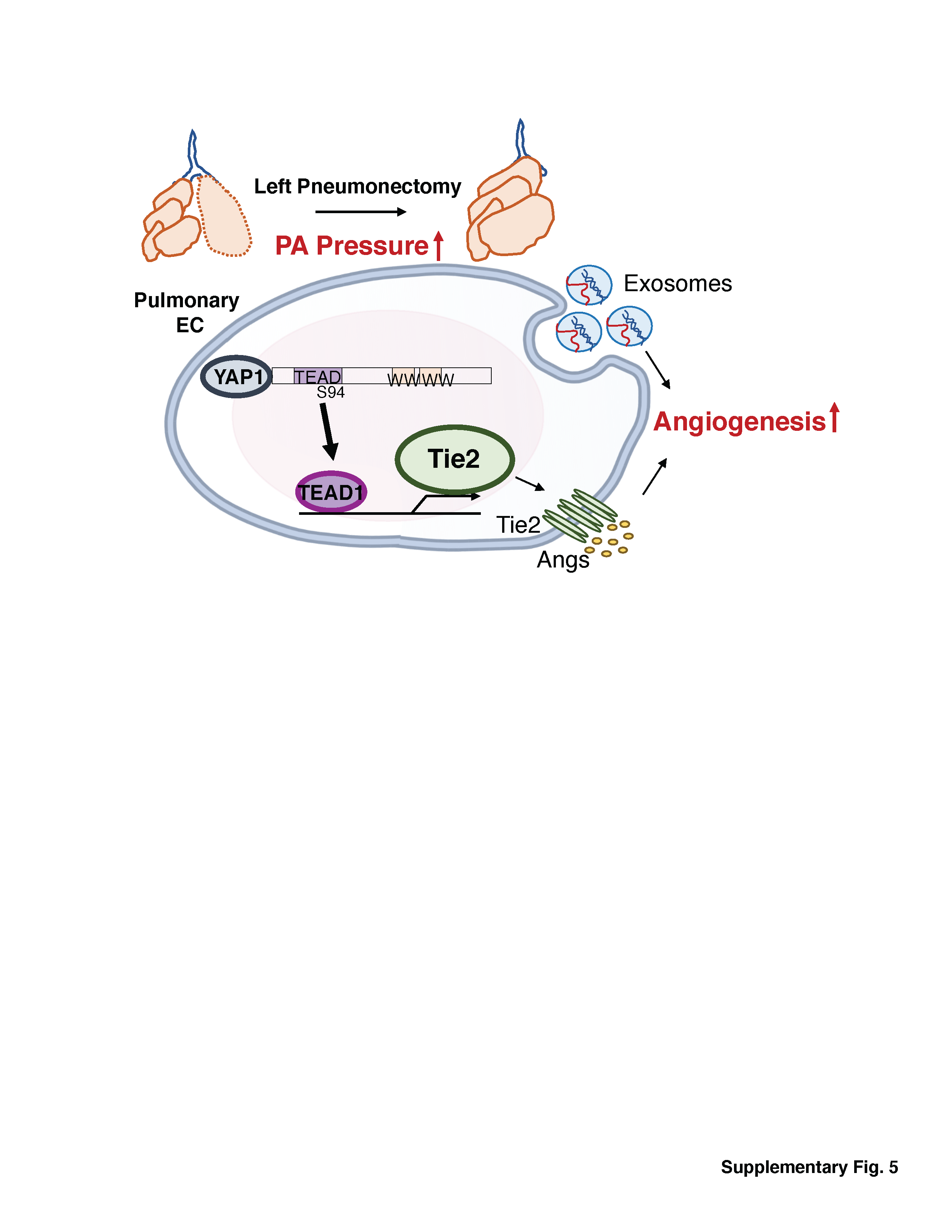

Supplement: Supplementary file 6 [file Image5.TIFF]

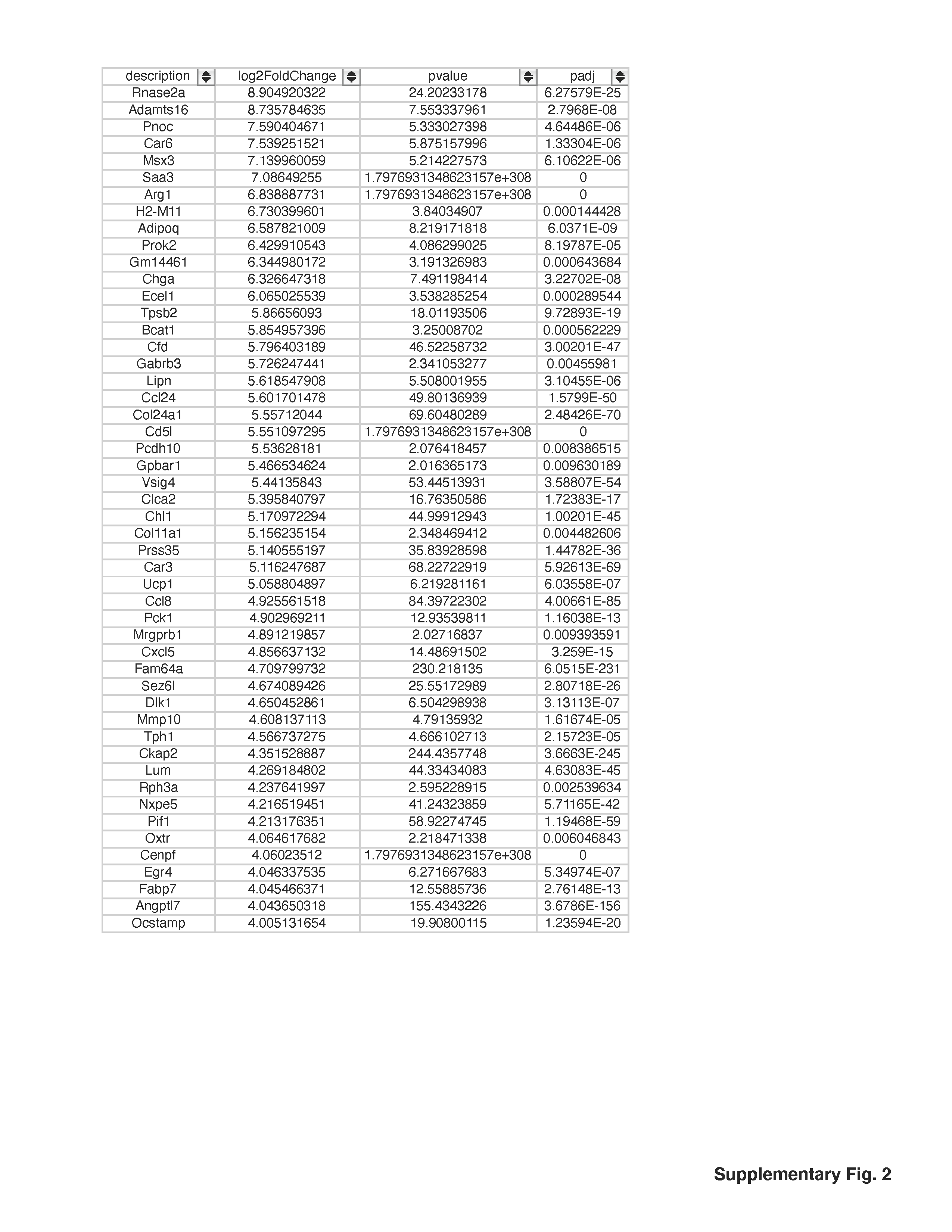

Supplement: Supplementary file 10 [file Image2.TIFF]

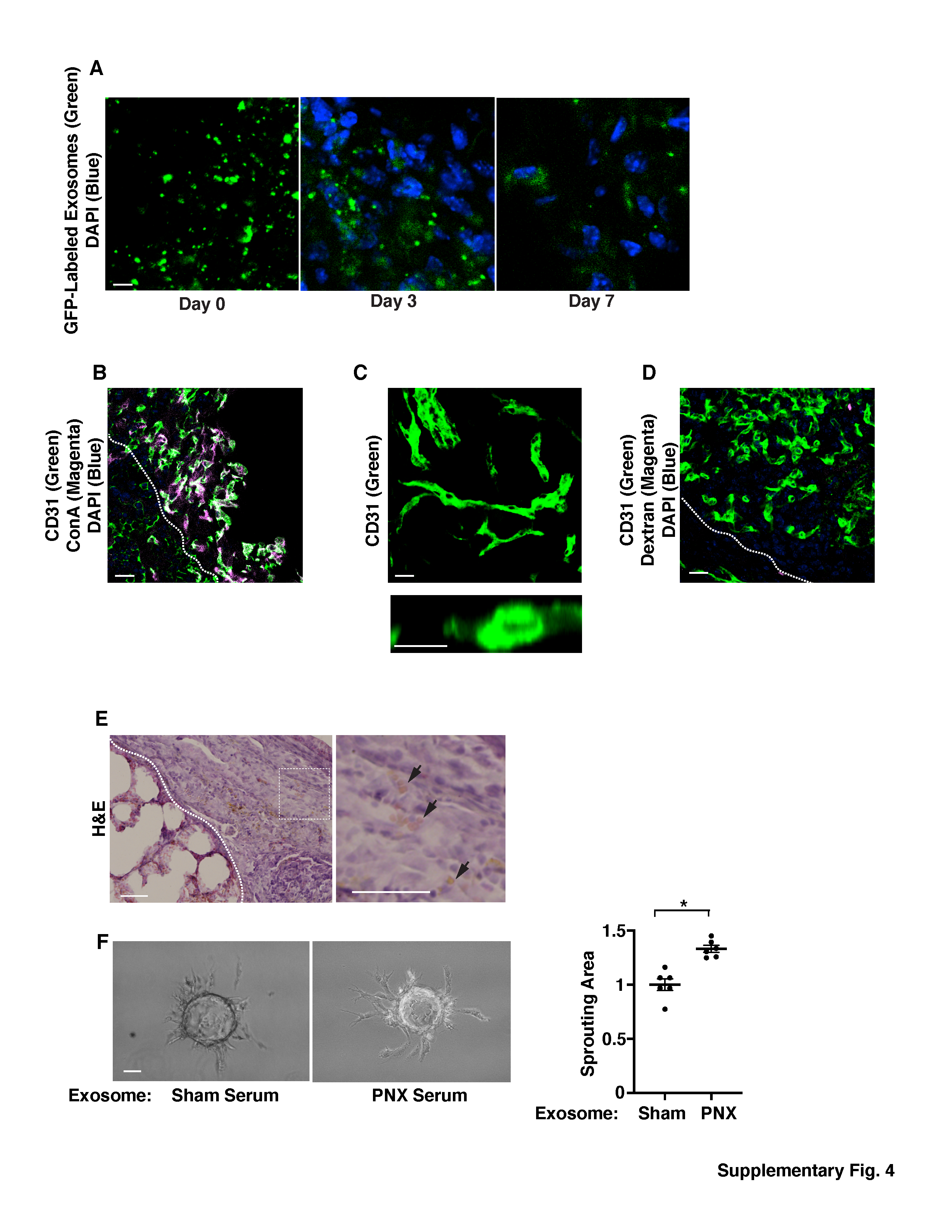

Supplement: Supplementary file 11 [file Image4.TIFF]
