# Supplementary material for: Hydrostatic Pressure Controls Angiogenesis Through Endothelial YAP1 During Lung Regeneration
Source: Front Bioeng Biotechnol. 2022 Feb 18;10:823642. doi: 10.3389/fbioe.2022.823642 (PMC8896883; doi:10.3389/fbioe.2022.823642)
Supplement: Supplementary file 2 [file DataSheet2.PDF]

Proteins	Sham1 (Spectrum Count)	Sham2 (Spectrum Count)	PNX1 (Spectrum Count)	PNX2 (SpectrumCount)	PNX3 (Spectrum Count)
Tln1	67	68	107	90	50
Thbs1	67	56	81	53	50
Lamb2	38	54	33	26	25
Lama5	53	50	46	33	24
Lamc1	63	49	46	47	41
Sptan1	51	45	65	31	45
Hist1h2bm	49	33	67	27	39
Fasn	29	33	65	47	20
Flna	34	32	89	77	26
Sptbn1	39	32	46	23	33
Actn4	25	31	39	14	18
Actg1	84	30	86	46	25
Dpysl2	43	30	42	27	22
Tuba1b	25	30	29	29	27
Myh9	17	29	57	32	26
Tuba1a	24	28	0	27	28
Vcp	39	27	92	38	30
C3	34	25	81	56	34
Cltc	28	25	58	26	27
Tubb4b	38	25	47	22	18
Actn1	18	25	42	17	11
Cbr2	58	23	37	22	9
Tubb5	33	23	44	22	17
Tuba4a	19	23	23	22	18
Msn	47	22	43	14	12
Hist1h2ab	20	22	32	13	18
Lamb1	27	21	28	21	18
Aldh1a1	35	21	23	10	6
Lama3	22	21	8	6	2
Hspg2	38	20	38	22	12
Hist2h2ac	23	20	28	13	16
Ager	36	20	15	14	11
Hspa8	20	20	25	14	20
Tnc	115	19	236	123	35
Nid1	37	19	32	21	13
Hist1h4a	16	19	33	10	26
Gsn	23	18	17	14	10
Flnb	10	18	30	9	18
Ehd2	12	18	6	4	8
Hist1h3b	43	17	95	28	16
Anxa2	25	17	36	11	15
Itgb1	15	16	17	10	17
Cavin1	15	16	10	3	12
Col4a1	20	15	24	9	14
Aldh2	18	15	11	8	5
Cap1	18	14	34	29	10
Hba	23	14	11	5	16
Hist1h1e	13	14	4	2	14
Eef2	17	12	51	27	14
Col4a3	10	12	9	5	8

Itih2	9	12	5	8	11
Hist1h1c	11	12	4	0	13
Bcam	12	12	5	2	3
Myl6	3	12	3	1	8
H2afx	14	11	21	7	9
Vcl	24	11	27	14	7
Xdh	20	11	19	5	2
Vim	6	11	16	10	10
Anxa1	1	11	8	3	14
Ehd4	13	11	7	1	4
H2afy	17	10	17	10	6
Ezr	11	10	15	4	9
Anxa5	15	10	12	1	4
Serpinc1	9	10	3	3	13
Itga3	5	10	6	3	2
Actc1	24	9	34	17	12
C4b	10	9	38	30	15
Dync1h1	13	9	38	17	5
Selenbp1	19	9	8	4	3
Gdi2	6	9	14	5	3
Gnb1	9	9	8	1	4
Npnt	4	9	2	2	4
Fn1	20	8	121	67	19
Lgals3bp	21	8	44	33	9
Hsp90ab1	22	8	29	11	9
Pkm	13	8	22	16	12
Ppia	20	8	18	15	8
Hmgb1	12	8	21	7	7
Agrn	17	8	14	7	0
Eno1	11	8	15	5	4
Ace	18	8	11	8	2
Ap2b1	6	8	17	4	4
Fbln1	6	8	7	5	12
Cct6a	5	8	11	4	8
Apob	6	8	7	3	7
Lamc2	5	8	3	1	6
Gstm1	7	8	5	0	4
Hbb-b1	30	7	13	4	7
Vat1	8	7	10	6	1
Krt5	1	7	1	2	8
Actr3	17	6	38	21	5
Tgm2	13	6	34	8	5
Eef1a1	8	6	20	11	4
H2afz	8	6	6	2	7
Gapdh	6	6	14	8	10
Tcp1	4	6	16	7	9
Cd36	9	6	6	4	5
Clic5	11	6	3	2	3
Arpc3	2	6	8	0	1
Tkt	10	5	23	13	2
Col4a2	11	5	11	5	5

Ppp2r1a	9	5	15	11	3
Rdx	15	5	13	2	0
Pgk1	7	5	12	7	3
Col4a4	10	5	9	3	1
Cct5	2	5	10	3	5
Pfn1	12	4	24	14	2
Ehd1	14	4	13	3	2
Lama4	13	4	14	8	1
Psma2	5	4	14	6	6
Serpinh1	10	4	13	5	4
Anxa6	7	4	16	0	5
Arhgdia	9	4	9	3	5
Rap1b	6	4	8	4	5
Myo1c	9	4	4	3	4
Lamb3	9	4	2	1	0
Inmt	35	3	26	11	5
Iqgap1	20	3	31	13	4
Uba1	14	3	28	12	4
Ctsb	11	3	18	16	2
Hsp90aa1	14	3	16	5	2
Ncl	5	3	14	8	3
Serpinf1	9	3	10	5	4
Lmna	8	3	8	4	3
Gpi	9	3	9	4	2
Coro1c	3	3	9	4	2
Fbn1	17	2	25	12	13
Clic4	14	2	18	3	0
Ywhaz	16	2	13	1	6
Rack1	8	2	16	10	1
Arpc1b	2	2	17	6	1
Ywhaq	14	2	8	2	2
Clu	8	2	11	4	3
Postn	5	2	11	5	4
Aldoa	7	2	8	3	1
Adk	6	2	11	3	1
Sod1	11	2	3	3	0
Psma6	3	2	11	4	0
Wars	4	2	8	2	0
Hnrnpa2b1	2	2	2	1	8
Acly	9	1	17	7	0
Cct2	3	1	15	6	0
Arpc2	6	1	16	2	1
Vwf	6	1	14	4	0
Cfl1	4	1	11	6	0
Cct7	1	1	8	5	2
Atp6v1a	2	1	8	4	1
Ap2a2	3	1	10	3	1
Itih3	2	1	1	1	9
Psmb3	3	1	10	3	1
Psma4	2	1	14	1	1
Atp6v1b2	1	1	8	2	0
