# Supplementary material for: Hydrostatic Pressure Controls Angiogenesis Through Endothelial YAP1 During Lung Regeneration
Source: Front Bioeng Biotechnol. 2022 Feb 18;10:823642. doi: 10.3389/fbioe.2022.823642 (PMC8896883; doi:10.3389/fbioe.2022.823642)
Supplement: Supplementary file 4 [file DataSheet4.PDF]

Biological Processes GO Terms	PValue	Fold Enrichment	Genes
cell cycle	3.53103E-45	4.336982677	PRC1, DBF4, KNTC1, AURKA, AURKB, BRINP1, CDCA8, OIP5, CDKN2C, INCENP, CCSAP, CDCA2, H2AFX, CCNA1, CCNA2, CDCA5, ASPM, CDCA3, LIG1, SGOL1, ESCO2, WEE1, NCAPD2, UHRF1, MAD2L1, SPAG5, MAPK4, CNTROB, DSCC1, TICRR, NEK2, ANLN, CHEK1, CHEK2, MYBL2, RCC1, SPC24, SPC25, NCAPG2, FBXO5, SKA3, SKA2, SKA1, DDIAS, HELLS, ERCC6L, CKAP2, DIXDC1, MKI67, NUF2, BRCA2, SPDL1, CDC20, NDC80, GAS1, PMF1, BRCA1, FAM64A, PLK1, CHAF1A, CHAF1B, E2F1, KIF23, CLSPN, E2F7, BORA, E2F8, CDT1, CCNE2, FAM83D, CCNE1, KIF2C, MCM8, CDC45, MCM7, MASTL, CDC7, CDC6, CDK1, KIF11, DSN1, CCNF, TPX2, NUSAP1, MCM2, UBE2C, MCM3, ECT2, MCM5, NEK11, MCM6, APITD1, FANCD2, NSL1, BUB1B, UBE2S, MELK, CKS1B, FOXM1, CEP55, NCAPH, BUB1, ZWILCH, DLGAP5, GMNN, PSRC1, KIF18B, CENPE, BIRC5, KNSTRN, CDKN3, RACGAP1, CDC25C, SMC2, GSG2, CCNB1, CCNB2, KIF20B, CKS2, CENPW, CHTF18, MIS18BP1, CIT, SMPD3
cell division	2.56732E-36	4.995535204	KIF23, KIFC1, PRC1, BORA, KNTC1, AURKA, AURKB, FAM83D, CCNE2, CCNE1, KIF2C, CDC45, CDCA8, OIP5, CCSAP, INCENP, CDCA2, MASTL, CCNA1, CCNA2, CDCA5, ASPM, CDCA3, CDC7, KIF14, CDC6, CDK1, KIF11, DSN1, LIG1, SGOL1, CCNF, TPX2, NUSAP1, UBE2C, ECT2, MCM5, WEE1, NCAPD2, MAD2L1, APITD1, SPAG5, NSL1, CNTROB, BUB1B, UBE2S, CKS1B, NEK2, ANLN, CHEK2, CEP55, RCC1, SPC24, SPC25, NCAPH, NCAPG2, BUB1, SKA3, FBXO5, SKA2, SKA1, ZWILCH, HELLS, ERCC6L, KIFC5B, PSRC1, NUF2, KIF18B, CENPE, SPDL1, CDC20, NDC80, BIRC5, PMF1, RACGAP1, KNSTRN, CDC25C, SMC2, CCNB1, FAM64A, CCNB2, PLK1, KIF20B, CKS2, CENPW, MIS18BP1, CIT
mitotic nuclear division	5.27394E-36	5.814546764	KIF23, BORA, KNTC1, AURKA, AURKB, FAM83D, KIF2C, CDCA8, OIP5, CCSAP, INCENP, CDCA2, MASTL, CCNA1, CCNA2, CDCA5, ASPM, TUBB3, CDCA3, CDC6, CDK1, KIF11, DSN1, CCNF, SGOL1, TPX2, NUSAP1, ESPL1, UBE2C, WEE1, NCAPD2, MAD2L1, APITD1, SPAG5, NSL1, BUB1B, NEK2, ANLN, CHEK2, CEP55, RCC1, SPC24, SPC25, NCAPH, NCAPG2, BUB1, SKA3, FBXO5, SKA2, SKA1, ZWILCH, HELLS, ERCC6L, CENPN, PSRC1, NUF2, KIF18B, CENPE, SPDL1, BIRC5, NDC80, CDC20, PMF1, KNSTRN, CDC25C, SMC2, CENPH, CCNB1, FAM64A, CCNB2, PLK1, KIF20B, CENPW, MIS18BP1, CIT
DNA replication	1.31349E-18	6.285383234	BLM, TICRR, DBF4, MCM10, CDT1, PRIM1, MCM8, CDC45, MCM7, POLE2, ADRA2A, POLQ, ORC1, FEN1, GINS1, GINS2, CDC6, DTL, LIG1, PIF1, POLE, RMI2, MCM2, MCM3, 2810417H13RIK, BRCA1, MCM5, MCM6, RFC5, RFC4, RRM2, RRM1, CHTF18, CHAF1A, CHAF1B, DSCC1
chromosome segregation	2.03425E-16	6.997491259	NEK2, RCC1, KIF2C, SPC25, OIP5, INCENP, BUB1, CDCA2, SKA3, SKA2, SKA1, TOP2A, CENPN, KIF11, DSN1, SGOL1, CENPF, ESPL1, NDC80, BIRC5, CENPE, PMF1, KNSTRN, BRCA1, ESCO2, CENPH, SPAG5, NSL1, CENPW
cell adhesion	4.18401E-13	2.789543796	CADM3, CADM4, MYBPC2, FERMT1, NRCAM, WISP1, TGFBI, SPON2, CLCA2, CDHR1, SDK1, CDHR3, PTPRU, CERCAM, AMIGO2, TNFAIP6, HAS1, VCAN, HAS2, ADAM12, MFAP4, CHL1, AOC3, CYP1B1, TNC, COL28A1, CDH2, CDH3, ITGAM, EPHB1, APLP1, ISLR, COL6A6, COL6A5, COL6A4, MSLN, COL6A2, COL6A1, CD4, GPNMB, CD6, THBS2, FN1, SPP1, THBS4, COL18A1, FLRT2, SELP, HAPLN1, PCDH10, NFASC, COL15A1, EFS, NID2, EMILIN2, COL16A1, COL5A1, LAMA1, LYVE1, COL14A1, TENM3, TROAP, ITGAD
mitotic sister chromatid segregation	3.23071E-12	13.07177528	NEK2, DSN1, NUSAP1, KIF18B, ESPL1, NDC80, KNSTRN, CDCA8, MAD2L1, PLK1, SPAG5, CENPA, NSL1, CIT
inflammatory response	3.14431E-11	2.996519914	CXCL1, C3AR1, HMGB2, CCL2, CXCL5, C3, FFAR2, CXCL3, CCL9, CSPG4, CCL8, BDKRB1, PF4, CCL7, IL10, CXCL10, CALCA, CCL24, CHIL4, SLC11A1, MEFV, CHIL3, CYP26B1, PYCARD, REG3G, TPSB2, ADAM8, NR1H4, SPP1, SELP, IL6, BMP2, IL5, C4B, ACKR1, CD5L, CELA1, CD163, CCL11, CCL12, TNFRSF9, CCR7, CCR5, CXCL13, CCR3, PLA2G7, NGFR, CD14
cellular response to tumor necrosis factor	5.7757E-09	4.490239689	IL6, CCL2, EDN1, CCL9, CCL8, PPARGC1A, CCL7, BRCA1, DCSTAMP, PCK1, CCL24, ADAMTS7, CCL11, CALCA, CCL12, OCSTAMP, SFRP1, PYCARD, FABP4, HAS2, COL1A1, ADAMTS12, DBN1
cellular response to DNA damage stimulus	8.52794E-09	2.505423595	RAD51C, CLSPN, RAD51B, E2F7, MCM10, MCM8, MCM7, H2AFX, MASTL, TOP2A, MMS22L, DTL, GEN1, NEIL3, LIG1, POLE, RAD51, UHRF1, APITD1, FANCD2, SPATA18, PIDD1, UBE2T, BLM, TICRR, FOXM1, UNG, EGLN3, CHEK1, CHEK2, POLQ, DDIAS, FEN1, EXO1, RAD51AP1, PIF1, BRIP1, BRCA2, ATAD5, CDKN3, RAD54L, 2810417H13RIK, BRCA1, PARPBP, RAD54B, ZRANB3, CHAF1A, CHAF1B, BARD1
immune response	1.52497E-08	2.921239695	CXCL1, CCL2, CXCL5, VPREB3, CXCL3, ENPP3, CCL9, CCL8, TNFSF14, PF4, CCL7, IL10, CXCL10, LIF, CCL24, CLEC4E, CXCR5, CD4, NFIL3, CD28, IL6, IL5, PRG4, H2-Q1, MCPT4, CCR9, CCL11, CCL12, TNFRSF9, CCR7, FCGR2B, H2-EB2, CCR5, CXCL13, SLPI, CMA1, NGFR
DNA replication initiation	4.86083E-08	9.84273555	CCNE2, CDC7, CDC6, CCNE1, CDC45, MCM7, MCM2, MCM3, MCM10, MCM5, MCM6
microtubule-based movement	8.2298E-08	4.955782934	KIF14, KIF23, KIFC5B, KIFC1, KIF22, DNAH9, KIF11, KIF27, KIF15, DNAH7A, KIF18A, KIF18B, CENPE, DNAH5, KIF2C, KIF4, KIF20B, KIF20A
cellular response to interleukin-1	1.22008E-07	4.831888361	IL6, CCL2, EDN1, CCL9, SAA3, CCL8, SOX9, CCL7, PCK1, CCL24, ADAMTS7, CCL11, CCL12, SFRP1, PYCARD, HAS2, ADAMTS12, FN1
chemokine-mediated signaling pathway	1.46097E-07	5.856834377	CXCL1, CCL2, CXCL5, CXCL3, ACKR1, CCL9, CCL8, PF4, CCL7, CXCL10, CCR9, CCL24, CCL11, CCL12, CXCL13
mitotic metaphase plate congression	2.20801E-07	7.579432723	KIFC5B, CCNB1, KIF14, KIF22, KIF2C, KIFC1, CDCA8, PSRC1, KIF18A, SPDL1, CENPE, CDCA5
DNA repair	2.80709E-07	2.566202065	KIF22, RAD51C, CLSPN, RAD51B, BLM, TICRR, UNG, FOXM1, CHEK1, CHEK2, ANKLE1, MCM8, H2AFX, POLQ, FEN1, EXO1, MMS22L, RAD51AP1, GEN1, NEIL3, PIF1, LIG1, POLE, BRCA2, BRIP1, RAD54L, 2810417H13RIK, BRCA1, RAD51, UHRF1, APITD1, FANCD2, PARPBP, ZRANB3, CHAF1A, CHAF1B, UBE2T, BARD1
xenobiotic glucuronidation	7.40596E-07	16.70282396	UGT1A10, UGT1A9, UGT1A7C, UGT1A2, UGT1A6B, UGT1A5, UGT1A1
collagen fibril organization	1.04481E-06	6.607710579	COL14A1, CYP1B1, SFRP2, LUM, COL3A1, COL1A2, COL1A1, LOXL2, COL11A1, ADAMTS2, COL5A2, COL5A1
chemotaxis	2.09626E-06	3.639840573	C3AR1, HMGB2, CCL2, CXCL5, CXCL3, CCL9, CCL8, PF4, CCL7, CXCL10, CCR9, CCL24, CCL11, CCL12, PROK2, CCR7, CXCR5, CCR5, CXCL13, CCR3
male gonad development	3.97561E-06	3.643090431	WNT5A, LRRC6, TESC, HMGB2, BRIP1, SOX9, INSL6, MMP14, WT1, WNT2B, INHBA, WNT4, SFRP1, BOK, SFRP2, RRM1, GATA4, ASPM, TEX11
response to lipopolysaccharide	5.12969E-06	2.834271797	CXCL1, HMGB2, CXCL5, CXCL3, EDN1, PF4, BDKRB1, DCN, SCGB1A1, CXCL10, SLC11A1, WFDC21, FMO1, SPON2, CD6, LOXL1, PTGER3, CYP1A1, SPARC, UGT1A1, PCK1, TNFRSF9, CCR7, CXCL13, SLPI, NGFR
mitotic cytokinesis	5.77285E-06	7.158353127	CKAP2, KIF23, PLK1, CENPA, KIF4, NUSAP1, ANLN, CEP55, RACGAP1, KIF20A
metaphase plate congression	7.21961E-06	12.52711797	FAM83D, KIF22, KIF2C, CENPQ, CENPF, NDC80, CENPE
collagen catabolic process	8.00911E-06	8.053147268	MMP10, CTSK, PRTN3, MMP8, MMP19, MMP14, MMP13, ADAMTS2, MMP2
double-strand break repair via homologous recombination	1.26802E-05	4.421335755	RAD51C, RAD51B, MMS22L, RAD51AP1, BLM, GEN1, TONSL, BRCA2, RAD54L, BRCA1, RAD51, MCM8, H2AFX, RAD54B
osteoblast differentiation	1.80259E-05	3.420805919	BMP2, FIGNL1, TNC, IGF1, FHL2, LEF1, SNAI1, GLI1, SFRP1, CREB3L1, COL6A1, VCAN, COL1A1, GPNMB, IGFBP3, CHRD, SPP1, TWIST1
response to drug	5.2643E-05	2.153840764	HMGB2, OXTR, CDH3, MDK, CBX7, MMP2, SCGB1A1, IL10, ALDH3A1, ARG1, TYMS, MCM7, GATA4, PTN, COL18A1, CDK1, IL6, CYP1A1, LGALS1, ATP1A3, PPARGC1A, RAD54L, UGT1A1, RAD51, CCNB1, INHBA, SFRP1, ABCB1B, SFRP2, NTRK1, ABAT, RAD54B, COL1A1, NNMT
positive regulation of cytokinesis	0.00010418	5.856834377	KIF23, KIF14, CDC6, CXCR5, KIF20B, AURKB, CIT, RACGAP1, ECT2
G2/M transition of mitotic cell cycle	0.00010418	5.856834377	PLK1, FOXM1, SKP2, CHEK1, BIRC5, MASTL, CHEK2, CIT, CDC25C
activation of protein kinase activity	0.00010418	5.856834377	CALCA, KIF14, SLC11A1, CLSPN, CKS1B, TPX2, CKS2, ADRA2A, ECT2
wound healing	0.000108611	3.426871178	WNT5A, IL6, TNC, COL3A1, SPARC, DCN, CDH3, MMP12, TIMP1, SLC11A1, ALOX15, BNC1, SERPINB2, COL1A1, FN1
negative regulation of angiogenesis	0.000110962	3.877441277	CCL2, PF4, DCN, SPARC, CXCL10, LIF, SERPINF1, MEG3, PTN, HHIP, NGFR, THBS2, THBS4
G1/S transition of mitotic cell cycle	0.000119192	4.156463106	CCNE2, CCNE1, INHBA, CDKN2C, POLE, SKP2, IQGAP3, TCF19, CDKN3, RCC1, RHOU, CDCA5
regulation of attachment of spindle microtubules to kinetochore	0.000145282	15.33932813	NEK2, SPAG5, KNSTRN, RACGAP1, ECT2
regulation of cell proliferation	0.000155086	2.365094646	CXCL5, FOXM1, CXCL3, TNC, EZH2, EGLN3, NR3C2, CHEK1, PF4, SOX9, LGR5, CXCL10, CDCA7, MATK, IL6, PRG4, IGF1, BRCA2, ESR2, SPARC, CELA1, BRCA1, TNFRSF9, SFRP2, CXCL13
protein heterotrimerization	0.000160123	7.911863983	C1QTNF6, COL6A4, COL1A2, COL6A2, COL6A1, COL1A1, ADIPOQ
mitotic spindle assembly	0.000162616	5.522158127	KIFC5B, KIFC1, KIF11, NEK2, TPX2, BIRC5, CDC20, CHEK2, MYBL2
cellular response to retinoic acid	0.000184879	3.964626348	ALDH1A2, BRINP1, TESC, CCL2, OSR1, SERPINF1, TNC, CYP26B1, CYP26A1, COL1A1, SOX9, PCK1
positive regulation of cell migration	0.000201954	2.433134807	COL18A1, C3AR1, BMP2, ARHGEF39, FAM110C, EDN1, IGF1, LEF1, MMP14, LRRC15, MMP2, SNAI1, CXCL10, CCL24, CCL11, TNFAIP6, CEMIP, ADRA2A, ROR2, HAS2, COL1A1, GPNMB, FN1
mitotic chromosome condensation	0.000202484	9.911565869	NCAPH, NCAPG, NUSAP1, CDCA5, SMC2, NCAPD2
negative regulation of neuron death	0.000228332	4.218315236	CHGA, IL6, GABRB3, SERPINF1, APOE, NTRK1, IGF1, ESR2, GPNMB, PPARGC1A, MDK
positive regulation of endothelial cell proliferation	0.000243917	3.846279292	CCL24, CCL11, WNT5A, ARG1, BMP2, HMGB2, CCL2, LRG1, CCR3, VASH2, IL10, THBS4
cytokinesis	0.000245656	5.223663093	KIF23, PRC1, PLK1, INCENP, BRCA2, BIRC5, CIT, ECT2, KIF20A
flavonoid glucuronidation	0.000295668	7.158353127	UGT1A10, UGT1A9, UGT1A7C, UGT1A2, UGT1A6B, UGT1A5, UGT1A1
flavonoid biosynthetic process	0.000295668	7.158353127	UGT1A10, UGT1A9, UGT1A7C, UGT1A2, UGT1A6B, UGT1A5, UGT1A1
cartilage development	0.000391163	3.404582585	WNT5A, TYMS, BMP2, ITGB8, SFRP2, LUM, EDN1, MGP, PRRX2, SOX9, MMP13, COL11A1, TIMP1
retinoic acid metabolic process	0.000437086	8.590023753	ALDH1A2, LRAT, ADH1, ALDH1A3, CYP26B1, CYP26A1
positive regulation of transcription, DNA-templated	0.000477384	1.715022103	WNT5A, E2F1, CKS1B, HMGB2, BLM, GDF6, FOXM1, ARNT2, CHEK2, SOX9, MDK, WT1, IL10, GLI1, WNT4, LHX2, GATA4, MEG3, CCNA2, ETV4, IL6, TESC, BMP2, IL5, PSRC1, MLXIPL, BRCA2, FGF23, ATAD2, LEF1, IGF1, ESR2, KLF15, PPARGC1A, SNAI1, BRCA1, INHBA, SFRP1, EBF3, EBF2, CKS2, MYRF, ROR2, ZFPM2, COL1A1, NFE2L3
DNA recombination	0.000546967	3.284420847	EXO1, RAD51C, HMGB2, RAD51B, RAD51AP1, BLM, PIF1, LIG1, RAG1, BRCA2, H2AFX, BRCA1, RAD51
heterophilic cell-cell adhesion via plasma membrane cell adhesion molecules	0.000592867	4.129819112	AMIGO2, SELP, CADM3, CADM4, LGALS1, TENM3, GM609, LGALS7, CDH2, CD6
proteolysis	0.000593427	1.697341463	ADAMTS19, ECEL1, PRTN3, LGMN, ADAMTS16, MMP8, UCHL1, MMP23, MMP2, CPZ, PAPPA, ADAMTS12, CFI, TPSB2, ADAM8, CFD, PRSS35, ADAM28, CAPN6, CLCA2, F10, CFB, TRABD2B, MMP19, MCPT4, ESPL1, MMP14, CELA1, MMP13, MMP12, ADAMTS7, MMP10, CTSK, ADAMTS9, CAPN11, CPXM1, MYRF, CMA1, PAPPA2, ADAM12, C1S1, PHEX, TPSAB1, ADAMTS2, ADAMTS4, USP26
positive regulation of inflammatory response	0.000611226	3.749613543	CCL24, CCL11, WNT5A, CCL12, CCL2, CCR5, CCL9, CCL8, FABP4, ADAM8, CCL7
spindle organization	0.00061154	8.053147268	KIF11, SPAG5, AURKA, AURKB, KNSTRN, ASPM
chromosome condensation	0.00061154	8.053147268	CDK1, NCAPH, NCAPG2, SMC2, TOP2A, NCAPD2
lymphocyte chemotaxis	0.000678725	5.206075002	CCL24, CCL11, CCL12, CCL2, CCL9, CCL8, ADAM8, CCL7
protein localization to kinetochore	0.00077894	10.73752969	CDK1, BUB1B, TTK, SPDL1, AURKB
eosinophil chemotaxis	0.00077894	10.73752969	CCL24, CCL11, CCL2, CCR3, CCL7
DNA unwinding involved in DNA replication	0.00077894	10.73752969	MCM7, MCM2, TOP2A, RAD51, MCM6
extracellular matrix organization	0.000826959	2.825665708	COL18A1, ADAMTSL2, OLFML2B, ELN, CCDC80, NID2, VIT, SOX9, COL5A3, APLP1, LAMA1, COL6A4, TGFBI, VWA1, FN1
positive regulation of cell proliferation	0.000857751	1.703740874	CAV3, WNT5A, RAD51B, PRC1, CD248, FOXM1, TNC, ARNT2, EDN1, ESM1, SOX9, IL31RA, GLI1, CXCL10, ALDH3A1, TIMP1, LIF, ALDH1A2, FOLR2, PTN, FGFBP1, SHC4, FN1, CDC7, KIF14, COL18A1, IL6, IL5, ACER2, MLXIPL, CENPF, LEF1, IGF1, CDC20, GAS1, CLEC11A, PROK2, SFRP1, SFRP2, KIF20B, FABP4, SP6, HAS2
cell chemotaxis	0.00092861	3.30385529	CXCL1, CCL12, HMGB2, CCL2, CXCL5, CXCL13, CCL9, SAA3, CCL8, LEF1, EPHB1, CXCL10
mitotic cell cycle phase transition	0.000934273	17.18004751	CDK1, CKS1B, CKS2, NEK11
cilium movement	0.000985344	4.908585002	LRRC6, CCDC40, RSPH4A, CCDC151, DNAH7A, CFAP221, DNAH5, DNAH6
positive regulation of canonical Wnt signaling pathway	0.001003448	3.525756018	DKK2, DIXDC1, WNT4, SFRP1, SFRP2, SFRP4, ROR2, COL1A1, LGR5, ASPM, WNT2B
trophoblast giant cell differentiation	0.001110056	7.158353127	LIF, PLK4, E2F7, E2F8, GJB5, SNAI1
cellular response to interferon-gamma	0.001128361	3.473906665	CCL24, CCL11, WNT5A, CCL12, CCL2, EDN1, CCL9, GBP10, CCL8, AQP4, CCL7
extracellular fibril organization	0.001179144	9.761390628	LTBP2, COL3A1, MFAP4, MFAP5, COL5A1
aging	0.001190849	2.358532533	IL6, CCL2, CYP1A1, COL3A1, DCN, AURKB, PPARGC1A, IL10, TIMP1, ALDH3A1, PCK1, CALCA, ARG1, TYMS, SERPINF1, APOE, NTRK1, KRT14, ABAT
neutrophil chemotaxis	0.001265687	3.423560191	CCL24, CCL11, CXCL1, CCL12, CCL2, CXCL3, CCL9, CCL8, CCL7, ITGAM, SPP1
endodermal cell differentiation	0.001270455	5.567607988	INHBA, MMP8, COL6A1, MMP14, COL11A1, MMP2, FN1
skeletal system development	0.001383639	2.809820854	GDF3, HAPLN1, COL3A1, FAM20C, EDN1, FBN1, NPR3, SOX9, COL5A2, COL1A2, ROR2, VCAN, COL1A1, CHRD
cell differentiation	0.00139193	1.541799135	CSPG4, ILDR2, DLK1, GLI1, WNT4, OCSTAMP, OSR1, SRRM4, DMKN, CCNA1, MATK, TWIST1, BATF3, MDGA1, MMP19, FGF23, MGP, PTPRU, ECT2, PTPRQ, SPATA18, MYRF, ROR2, EFNA5, NGFR, DBN1, TBX18, CHL1, TSNAXIP1, WNT5A, MYBL1, NHS, MYBL2, SOX9, MDK, DAPL1, SPEG, MEG3, DCLK1, TRIP13, TESC, BMP2, CDC20, INSC, FRZB, RACGAP1, DCSTAMP, CLGN, SFRP1, SFRP2, NTRK1, TENM3, SFRP4, MYCBPAP, CIT, AGR3
cell proliferation	0.001471214	2.147505938	CDK1, CKS1B, MKI67, CYP1A1, CFB, E2F8, UCHL1, CSPG4, BRCA2, IGF1, AURKB, TACC3, MCM10, FAM83D, PROK2, UHRF1, MCM7, BOK, TGFBI, CKS2, BUB1, MELK
positive regulation of angiogenesis	0.001485258	2.662197444	WNT5A, C3AR1, CYP1B1, C3, CELA1, VASH2, BRCA1, CCL24, CCL11, LRG1, SFRP2, CCR3, HMOX1, GATA4, CMA1
mitotic spindle organization	0.001554106	5.368764846	CCNB1, STIL, SPC25, NDC80, AURKA, AURKB, RCC1
axon guidance	0.001570897	2.45017456	WNT5A, FLRT2, NFASC, GAS1, EPHB1, EPHB2, NRCAM, LAMA1, CRMP1, LHX2, NTRK1, MEG3, EFNA5, NGFR, EFNA4, CHL1, TUBB3
prostate gland epithelium morphogenesis	0.001703936	8.947941409	TNC, FOXA1, IGF1, ESR2, MMP2
response to activity	0.001754103	3.944398662	CDK1, CCL2, NTRK1, EDN1, PTN, PPARGC1A, ADIPOQ, IL10, PCK1
mitotic spindle midzone assembly	0.001803895	14.31670625	KIF23, KIF4, AURKB, RACGAP1
collagen biosynthetic process	0.001803895	14.31670625	ARG1, COL1A1, ADAMTS3, COL5A1
regulation of mitotic nuclear division	0.001859161	6.442517815	MKI67, BORA, KIF20B, FBXO5, CENPE, RCC1
immune system process	0.002022846	1.794260836	HMGB2, HMGB3, CD8A, C3, FFAR2, FCNA, C1QC, IL31RA, MARCO, CFP, MEFV, CLEC4E, PYCARD, CD4, CLEC4A2, CLEC4D, CFI, SPON2, CFD, NR1H4, EXO1, BPIFA1, CFB, THEMIS, CD5L, PADI4, DCSTAMP, C1QA, C1QB, SLPI, C1S1, CD14
monocyte chemotaxis	0.002232926	4.295011876	CCL24, CCL11, CALCA, CCL12, CCL2, CCL9, CCL8, CCL7
negative regulation of inflammatory response	0.00229136	2.962077156	TNFAIP6, MEFV, SERPINF1, APOE, MIR147, AOAH, CD276, PBK, ADIPOQ, NR1H4, IL10, IL22RA2
G2 DNA damage checkpoint	0.002347753	6.135731252	CLSPN, PLK1, DTL, CHEK1, BRCA1, NEK11
cellular response to fatty acid	0.002347753	6.135731252	CCNB1, E2F1, FFAR2, EDN1, UCP1, NR1H4
epithelial to mesenchymal transition	0.002697389	4.849206957	FAM83D, BMP2, WNT4, LEF1, LOXL2, SOX9, SNAI1
negative regulation of canonical Wnt signaling pathway	0.002738519	2.737017372	DKK2, WNT5A, BMP2, WNT4, SFRP1, SFRP2, SFRP4, ROR2, LEF1, CDH2, FRZB, SOX9, GLI1
positive regulation of ERK1 and ERK2 cascade	0.003003228	2.170351746	BMP2, IL6, CCL2, C3, CCL9, CCL8, FGF23, ESR2, CCL7, CCL24, CCL11, CCL12, ALOX15, NDRG4, NTRK1, GATA4, PYCARD, TREM2, GPNMB
negative regulation of hormone secretion	0.003198568	7.669664065	LIF, IL6, EDN1, FGF23, ADIPOQ
cell activation	0.003198568	7.669664065	LYPD1, SLURP1, IGF1, FN1, TIMP1
tissue remodeling	0.003198568	7.669664065	CSPG4, CELA1, MMP14, MMP2, THBS4
cellular response to platelet-derived growth factor stimulus	0.003594336	5.602189404	CCL2, HAS1, PTN, HAS2, CCNA2, ERRFI1
cellular response to estradiol stimulus	0.003749722	4.555315627	IL6, SFRP1, PPARGC1A, UGT1A1, CCNA2, MMP2, IL10
negative regulation of cell proliferation	0.003991727	1.733663648	PODN, CYP1B1, SLURP1, E2F7, SOX9, IL10, WT1, H19, LIF, ALDH1A2, CDKN2C, HMOX1, MEG3, DFNA5, IL6, TESC, BMP2, BRIP1, IGF1, RIAN, ESR2, PTPRU, FRZB, INHBA, TNFRSF9, SFRP1, SFRP2, NTRK1, SFRP4, ROR2, IGFBP3
organ regeneration	0.004153875	3.451348829	LIF, CDK1, CCL2, MKI67, CXCL5, PTPRU, UGT1A1, CCNA2, NNMT
retinol metabolic process	0.004368957	5.368764846	ALDH1A2, DHRS3, CYP1B1, LRAT, ADH1, ALDH1A3
replication fork processing	0.004368957	5.368764846	MMS22L, BLM, APITD1, TONSL, ZRANB3, RAD51
positive regulation of epithelial to mesenchymal transition	0.004377348	4.421335755	BMP2, CRB2, EZH2, LEF1, COL1A1, SNAI1, TWIST1
response to ionizing radiation	0.004639647	3.39079885	RAD51C, RAD51B, TICRR, KRT14, RRM1, RAD54B, RAD54L, BRCA1, RAD51
maternal process involved in parturition	0.004708656	10.73752969	CCL2, CYP1A1, EDN1, OXTR
regulation of mitotic spindle organization	0.004708656	10.73752969	PSRC1, BORA, TPX2, TACC3
positive regulation of DNA-directed DNA polymerase activity	0.004708656	10.73752969	RFC5, RFC4, CHTF18, DSCC1
positive regulation of synapse assembly	0.004986028	3.067865626	FLRT2, AMIGO2, NTRK1, LRRN1, OXTR, EFNA5, TPBG, THBS2, EPHB1, EPHB2

bone mineralization	0.005079142	4.295011876	ASPN, BMP2, ALOX15, PTN, GPNMB, PHEX, MMP13
lung development	0.005103288	2.424603479	LIF, WNT5A, ARG1, ALDH1A2, CCDC40, FOXA1, MGP, IGF1, PTN, ZFPM2, SPARC, MMP14, ADAMTS2, GLI1
response to wounding	0.005167835	3.332336801	IL6, CCL2, CYP1A1, GRIN2C, AURKA, NGFR, MDK, FABP5, GLI1
axon regeneration	0.005399811	6.710956057	NREP, DHFR, APOE, FOLR1, CHL1
palate development	0.005992488	2.779125332	FRAS1, WNT5A, INHBA, DHRS3, OSR1, GABRB3, LEF1, GAS1, SNAI1, EPHB2, TWIST1
ERK1 and ERK2 cascade	0.00626198	4.955782934	CCL11, CCR3, IQGAP3, OXTR, IGF1, SOX9
spindle checkpoint	0.006281738	21.47505938	SPDL1, BIRC5, AURKB
development of primary male sexual characteristics	0.006281738	21.47505938	WNT5A, SFRP1, SFRP2
cellular response to interleukin-6	0.006804867	6.316193936	SELP, CCL2, FGF23, ENTPD2, PPARGC1A
bone trabecula formation	0.006820388	9.544470837	SFRP1, COL1A1, FBN2, MMP2
attachment of spindle microtubules to kinetochore	0.006820388	9.544470837	SGOL1, NUF2, NDC80, CENPE
kinetochore assembly	0.006820388	9.544470837	APITD1, CENPA, CENPW, CENPH
protein K6-linked ubiquitination	0.006820388	9.544470837	UBE2S, BRCA1, UBE2T, BARD1
regulation of mitotic metaphase/anaphase transition	0.006820388	9.544470837	CDC6, PLK1, CENPE, UBE2C
cellular response to transforming growth factor beta stimulus	0.007030209	3.168451384	WNT5A, ARG1, WNT4, SFRP1, EDN1, COL1A1, SOX9, PPARGC1A, DBN1
negative regulation of smooth muscle cell proliferation	0.007678612	3.955931991	NDRG4, HMOX1, ESR2, NPR3, IGFBP3, PPARGC1A, ADIPOQ
chromosome organization	0.007678612	3.955931991	CDCA8, BLM, BNC1, BRCA2, CENPW, RAD54L, SMC2
negative regulation of neuron apoptotic process	0.007754363	2.147505938	KIF14, CCL2, GABRB3, LGMN, BIRC5, PPARGC1A, MDK, CCL12, BOK, APOE, NTRK1, HMOX1, NGFR, ADAM8, FAIM2, CHL1
response to glucocorticoid	0.007845338	2.863341251	TYMS, IL6, PAPPA, PTPRU, SPARC, MDK, ADIPOQ, SCGB1A1, IL10, ALDH3A1
positive regulation of GTPase activity	0.008181886	2.206341717	WNT5A, CCL2, EZH2, CCL9, CCL8, RGS16, ECT2, CCL7, CCL24, CCL11, CCL12, WNT4, CCR7, NTRK1, ELMOD1
canonical Wnt signaling pathway	0.008254842	2.654220823	WNT5A, DIXDC1, WNT4, SFRP1, GATA4, SFRP4, LEF1, PTPRU, CDH3, WNT2B, GLI1
cellular response to hypoxia	0.008369584	2.501948666	CCNB1, E2F1, SFRP1, FNDC1, HMOX1, EDN1, PTN, ADAM8, PPARGC1A, CCNA2, PCK1, TWIST1
cell-cell signaling	0.008369584	2.501948666	WNT5A, WNT4, WISP1, CCR5, CXCL13, GATA4, EDN1, FJX1, ADRA2A, ADRA1B, FGFBP1, WNT2B
drug metabolic process	0.008431955	5.965294273	CYP1A1, FMO1, FMO3, DCK, CYP2B10
mitotic spindle assembly checkpoint	0.008431955	5.965294273	MAD2L1, PLK1, BUB1, BUB1B, CENPE
positive regulation of macrophage activation	0.008431955	5.965294273	WNT5A, ULBP1, IL10, RAET1B, IFI204
response to mechanical stimulus	0.008526966	3.067865626	CCNB1, CCL2, TNC, COL3A1, GATA4, BDKRB1, DCN, MMP14, MMP2
double-strand break repair	0.008526966	3.067865626	LIG1, BRCA2, CHEK2, POLQ, RAD54L, CDCA5, BRCA1, ESCO2, TRIP13
positive regulation of epithelial cell proliferation	0.008542585	2.825665708	WNT5A, IL6, SFRP1, OSR1, BNC1, IGF1, GAS1, SOX9, TBX18, TWIST1
positive regulation of neutrophil chemotaxis	0.008666485	4.601798439	CXCL1, C3AR1, CCR7, CXCL3, EDN1, THBS4
reciprocal meiotic recombination	0.008666485	4.601798439	RAD51C, RAD51B, CHTF18, TEX11, RAD51, TRIP13
glucose homeostasis	0.009091001	2.260532567	CAV3, IL6, PMCH, FFAR2, FBN1, FOXA1, MLXIPL, ADIPOQ, PCK1, GPR39, ADRA1B, ADRA2A, NGFR, NR1H4
positive regulation of keratinocyte proliferation	0.00940958	8.590023753	TGM1, HAS2, REG3G, CDH3
complement activation, alternative pathway	0.00940958	8.590023753	CFP, C3, CFB, CFD
cellular response to vitamin D	0.00940958	8.590023753	SFRP1, TNC, PTN, FGF23
liver regeneration	0.010079618	4.443115734	TYMS, HMOX1, EZH2, AURKA, IL10, GLI1
response to vitamin A	0.010293992	5.651331416	ARG1, TYMS, ALDH1A2, CYP1A1, GATA4
estrous cycle	0.010293992	5.651331416	HAS1, OXTR, PTN, HAS2, ESR2
embryo development	0.010917761	2.718361947	WNT5A, CKS1B, BMP2, LRAT, MEG3, BUB1, CKS2, BIRC5, DLK1, RACGAP1
positive regulation of smoothened signaling pathway	0.011642942	4.295011876	SFRP1, FOXA1, DCDC2A, PRRX2, GAS1, GLI1
positive regulation of protein kinase C activity	0.01217634	16.10629454	WNT5A, CEMIP, ROR2
regulation of mitotic centrosome separation	0.01217634	16.10629454	KIF11, NEK2, CHEK1
positive regulation of cell-substrate adhesion	0.012477343	3.579176564	ALOX15, FBLN2, CCDC80, PTN, VIT, DBN1, SPP1
positive regulation of interleukin-8 secretion	0.012495993	7.809112503	WNT5A, FCNA, FFAR2, PYCARD
one-carbon metabolic process	0.013363241	4.156463106	MTHFD2, DHFR, CAR13, ALDH1L2, CAR6, CAR3
ossification	0.013725931	2.460683888	BMP2, ALOX15, PTN, MGP, SPARC, MMP14, SOX9, COL11A1, COL5A2, SPP1, TWIST1
regulation of cell cycle	0.015161832	2.30089922	CCNE2, E2F1, CCNE1, FIGNL1, DTL, PRR11, CCNF, FOXA1, SKP2, MASTL, TACC3, 2810417H13RIK
bone morphogenesis	0.015246923	4.026573634	DHRS3, SFRP2, CYP26B1, HAS2, PAPPA2, MMP13
positive regulation of leukocyte migration	0.017400836	4.880695314	CCL12, SELP, CCL2, BDKRB1, AOC3
skin development	0.017549566	2.962077156	FRAS1, COL3A1, GJB3, COL1A1, NGFR, ADAMTS2, COL5A2, COL5A1
cellular response to lipopolysaccharide	0.018227114	1.849526645	WNT5A, HMGB2, IL6, CCL2, CXCL3, PPARGC1A, IL10, RAET1B, RAET1A, CXCL10, ARG1, MIR147, PYCARD, GBP10, SPON2, NR1H4, CD14, TNIP3
inner ear morphogenesis	0.018336977	2.684382423	WNT5A, MAFB, ALDH1A3, ROR2, PRRX2, FRZB, COL11A1, PTPRQ, EPHB2
positive regulation of tumor necrosis factor production	0.019139213	2.911872459	CCR7, CCL2, CCR5, PYCARD, PF4, SPON2, CD14, TWIST1
protein phosphorylation	0.019173439	1.454040479	WNT5A, STK33, NEK2, PASK, FAM20C, TTK, AURKA, CHEK1, AURKB, CHEK2, EPHB1, EPHB2, CCNE1, SPEG, BUB1, MASTL, DCLK1, MATK, CDC7, CDK1, BMP2, CAMK1G, BIRC5, PBK, GSG2, NEK11, WEE1, CCNB1, LAMA1, PLK4, PLK1, MAPK4, NTRK1, MAP3K19, ROR2, BUB1B, CIT, IGFBP3, MELK
stem cell differentiation	0.019528037	3.789716362	LIF, A2M, PHF19, OSR1, ETV4, SHC4
response to gamma radiation	0.019528037	3.789716362	CCL2, FANCD2, BRCA2, CHEK2, CCL7, CXCL10
brown fat cell differentiation	0.019528037	3.789716362	MRAP, LRG1, EBF2, FABP4, UCP1, ADIPOQ
eye morphogenesis	0.019671052	12.88503563	GAS1, COL5A2, COL5A1
mast cell chemotaxis	0.019671052	12.88503563	CCL11, CHGA, CCR3
double-strand break repair via break-induced replication	0.019671052	12.88503563	CDC7, GINS2, CDC45
regulation of vascular endothelial growth factor production	0.019671052	12.88503563	IL6, CCL2, AQP4
metaphase/anaphase transition of mitotic cell cycle	0.019671052	12.88503563	BUB1B, CIT, TACC3
protein localization to chromosome, centromeric region	0.019671052	12.88503563	CENPA, BUB1B, GSG2
osteoclast fusion	0.019671052	12.88503563	CD109, ADAM8, DCSTAMP
regulation of cell-cell adhesion	0.020209974	6.607710579	WNT4, LEF1, EFNA5, ADAM8
leukocyte migration involved in inflammatory response	0.020209974	6.607710579	CCL2, ADAM8, ITGAM, AOC3
positive regulation of gene expression	0.02032758	1.560843915	E2F1, HMGB2, TNC, IQGAP3, PF4, CDH3, SOX9, LIF, ALDH1A2, SLC11A1, OSR1, ITGB8, GATA4, CYP26B1, NFIL3, ETV4, TWIST1, CD28, FN1, CDK1, IL6, TESC, BMP2, PTGER3, LEF1, BRCA1, INHBA, CCR5, SFRP4
innate immune response	0.020923049	1.556941805	HMGB2, HMGB3, C3, FCNA, C1QC, MARCO, CFP, MEFV, CLEC4E, PYCARD, CLEC4A2, CLEC4D, CFI, PTX3, SPON2, CFD, NR1H4, MATK, C4B, BPIFA1, CFB, PADI4, C1QA, C1QB, TRIM59, SLPI, TREM2, C1S1, CD14
acute-phase response	0.021936186	3.681438751	IL6, SAA3, REG3G, UGT1A1, FN1, CD163
negative regulation of apoptotic process	0.022976377	1.441788439	WNT5A, STIL, FIGNL1, UNG, ARNT2, FHL2, AURKA, SOX9, INSL6, WT1, IL10, TIMP1, OSR1, NAIP1, HHIP, NR1H4, HELLS, SPP1, FN1, TWIST1, KIF14, CDK1, IL6, CENPF, LEF1, IGF1, BIRC5, GAS1, AMIGO2, PROK2, MAD2L1, SFRP1, PLK1, SERPINB2, NGFR, PIDD1, BARD1, TEX11
positive regulation of vascular endothelial growth factor production	0.02349517	4.473970705	C3AR1, CYP1B1, C3, GATA4, BRCA1
positive regulation of urine volume	0.024849449	6.135731252	PTGER3, EDN1, HAS2, NPR3
cellular response to follicle-stimulating hormone stimulus	0.024849449	6.135731252	INHBA, GATA4, EFNA5, PPARGC1A
positive regulation of protein import into nucleus, translocation	0.024849449	6.135731252	CDK1, IL6, IGF1, BRCA1
microtubule depolymerization	0.024849449	6.135731252	KIF14, KIF2C, KIF18A, KIF18B
positive regulation of fat cell differentiation	0.025337432	3.067865626	BMP2, ZBTB7C, SFRP1, SFRP2, IGF1, FRZB, TPH1
negative regulation of cell migration	0.025733638	2.228543898	WNT4, SLURP1, PODN, CYP1B1, SFRP1, CCR5, SFRP2, PTN, PTPRU, CHRD, ADIPOQ
embryonic limb morphogenesis	0.026504694	2.726991668	FRAS1, WNT5A, ALDH1A2, CYP26B1, LEF1, PRRX2, FBN2, TWIST1
metabolic process	0.027003449	1.484237366	BCAT1, ALPPL2, UNG, ENPP3, ARSI, RAG1, ALDH1L2, ALDH3A1, MTHFD2, ALDH1A2, UGT1A9, UGT1A7C, UGT1A2, UGT1A6B, CHIL3, ALDH1A3, ACAA1B, DCTD, CAMK1G, ACOXL, NEIL3, FBP1, NPL, UGT1A1, UGT1A10, HMGCS2, DNPH1, RRM1, CEMIP, ZRANB3, GLB1L3, TPH1
positive regulation of mesenchymal cell proliferation	0.027311027	3.482442062	WNT5A, PRRX2, GAS1, SOX9, TBX18, CHRD
positive regulation of bone mineralization	0.027311027	3.482442062	BMP2, WNT4, OSR1, FAM20C, CD276, FBN2
chromosome organization involved in meiotic cell cycle	0.028604631	10.73752969	CCNE2, CCNE1, RAD51
intramembranous ossification	0.028604631	10.73752969	CTSK, COL1A1, MMP2
negative regulation of centrosome duplication	0.028604631	10.73752969	KIFC5B, KIFC1, CCNF
regulation of branching involved in mammary gland duct morphogenesis	0.028604631	10.73752969	CAV3, WNT5A, ETV4
negative regulation of gastric acid secretion	0.028604631	10.73752969	PTGER3, GPR39, OXTR
cellular glucuronidation	0.028604631	10.73752969	UGT1A2, UGT1A5, UGT1A1
complement activation	0.030011125	5.726682502	C4B, C3, CFB, C1S1
positive regulation of T cell chemotaxis	0.030011125	5.726682502	WNT5A, CCR7, CXCL13, TNFSF14
regulation of double-strand break repair via homologous recombination	0.030011125	5.726682502	RAD51AP1, FIGNL1, CHEK1, RAD51
hematopoietic stem cell proliferation	0.030011125	5.726682502	WNT5A, PRG4, SFRP2, WNT2B
positive regulation of leukocyte chemotaxis	0.030011125	5.726682502	CXCL5, CXCL13, PF4, CXCL10
mitotic cell cycle	0.030285636	3.39079885	RRM1, CENPF, KIF18B, CENPW, CENPE, AURKA
response to steroid hormone	0.030734383	4.129819112	HMGB2, ADH1, ADRA1B, OXTR, SPP1
positive regulation of JAK-STAT cascade	0.030734383	4.129819112	IL6, CYP1B1, IL5, CXCL5, IL10
positive regulation of fibroblast proliferation	0.03082618	2.643084232	CCNB1, E2F1, WNT5A, CDC6, IGF1, NGFR, CCNA2, FN1
negative regulation of cell growth	0.031351362	2.061605701	INHBA, SFRP1, CDKN2C, SFRP2, TRO, PSRC1, FBP1, BDKRB1, GAS1, FRZB, WT1, DCSTAMP
positive regulation of smooth muscle cell proliferation	0.032245816	2.415944181	C3AR1, IL6, WISP1, HMOX1, EDN1, SKP2, IGF1, ORC1, PPARGC1A
positive regulation of cyclin-dependent protein serine/threonine kinase activity	0.03479316	3.976862849	STIL, CDC6, CKS1B, PSRC1, CKS2
cellular response to interleukin-4	0.03479316	3.976862849	ARG1, LEF1, MCM2, NFIL3, DCSTAMP
microtubule bundle formation	0.03479316	3.976862849	CAPN6, PRC1, PLK1, PSRC1, KIF20A
cell proliferation in forebrain	0.035690659	5.368764846	KIF14, DIXDC1, RRM1, FABP7
negative regulation of smooth muscle cell migration	0.035690659	5.368764846	NDRG4, IGFBP3, PPARGC1A, ADIPOQ
DNA-dependent DNA replication	0.035690659	5.368764846	RFC5, RFC4, POLE2, POLQ
inner cell mass cell proliferation	0.035690659	5.368764846	GINS1, NCAPG2, BRCA2, CHEK1
positive regulation of osteoblast differentiation	0.038155426	2.526477574	BMP2, WNT4, SFRP2, FAM20C, CD276, IGF1, FBN2, IFI204
cerebral cortex development	0.038155426	2.526477574	KIF14, LHX2, COL3A1, H2AFX, CDH2, TACC3, MDK, ASPM
hematopoietic progenitor cell differentiation	0.038653994	2.19133259	STON2, INHBA, SFRP1, KCP, ANLN, COL24A1, TOP2A, SMPD3, ESCO2, PTPRQ
negative regulation of cytokinesis	0.038827341	9.203596878	E2F7, E2F8, AURKB
glomerular visceral epithelial cell differentiation	0.038827341	9.203596878	KLF15, BASP1, WT1
strand invasion	0.038827341	9.203596878	RAD51C, RAD51B, RAD51
protein localization to chromatin	0.038827341	9.203596878	PLK1, EZH2, ESCO2
positive regulation of male gonad development	0.038827341	9.203596878	ZFPM2, SOX9, WT1
positive regulation of ubiquitin protein ligase activity	0.038827341	9.203596878	PLK1, CDC20, UBE2S
copper ion import	0.038827341	9.203596878	STEAP4, STEAP1, ATP7B
positive regulation of mitotic cell cycle spindle assembly checkpoint	0.038827341	9.203596878	MAD2L1, GEN1, NDC80
spermatogenesis	0.041492221	1.477399663	E2F1, TSNAXIP1, RAD51C, HMGB2, INSL6, SOX9, WT1, GLI1, GATA4, RPL39L, CYP26B1, H2AFX, CCNA1, ASPM, TRIP13, BRCA2, BRIP1, C330027C09RIK, CDC25C, RACGAP1, CCNB1, PROK2, CLGN, SPATA18, MYCBPAP, CHTF18, CIT, ADAMTS2
complement activation, classical pathway	0.041675197	2.733189376	C1QA, C1QB, C4B, C3, CFI, C1S1, C1QC
neural crest cell development	0.041880472	5.052955149	ALDH1A2, EDN1, CYP26A1, SOX9

negative regulation of peptidyl-tyrosine phosphorylation	0.041880472	5.052955149	SFRP1, SFRP2, IGF1, ERRFI1
non-canonical Wnt signaling pathway	0.041880472	5.052955149	WNT5A, WNT4, SFRP4, FRZB
positive regulation of calcium ion-dependent exocytosis	0.041880472	5.052955149	CACNA1I, CACNA1G, DOC2B, SCAMP5
positive regulation of cytosolic calcium ion concentration	0.043493714	1.886322784	CXCL1, CAV3, MCHR1, C3AR1, PTGER3, CCR5, CXCL13, PMCH, CXCL3, EDN1, ADRA1B, CACNA1G, OXTR
sprouting angiogenesis	0.043798484	3.702596445	E2F7, E2F8, LEF1, ESM1, LOXL2
mitotic cell cycle checkpoint	0.043798484	3.702596445	MAD2L1, KNTC1, TTK, CHEK1, ZWILCH
somitogenesis	0.044917824	2.684382423	WNT5A, SFRP1, CRB2, SFRP2, ROR2, LEF1, TBX18
positive regulation of peptidyl-tyrosine phosphorylation	0.045469758	2.126243503	LIF, IL6, IL5, CSPG4, IGF1, CD4, EFNA5, TREM2, ADIPOQ, THBS4
negative regulation of peptidase activity	0.0460649	2.019022677	WFDC12, A2M, BC048546, CST9, CD109, SERPINB2, SLPI, COL28A1, BIRC5, NAIP1, TIMP1
response to toxic substance	0.046496397	2.247389935	CDK1, TYMS, CYP1B1, CYP2F2, CYP1A1, ARNT2, PON1, INMT, RAD51
cellular response to glucose stimulus	0.046542922	2.419725001	SERPINF1, LGALS1, GATA4, MLXIPL, AQP4, IGF1, CMA1, PPARGC1A
negative regulation of JAK-STAT cascade	0.048144752	2.996519914	ASPN, FLRT2, PODN, DCN, LRRC15, NYX
positive regulation of protein phosphorylation	0.048584125	1.750684189	WNT5A, BMP2, PTGER3, C3, IQGAP3, CHEK2, SOX9, ADIPOQ, NTRK1, FNDC1, GATA4, CLIP3, GPNMB, CD6, KNDC1
DNA damage checkpoint	0.04874549	3.579176564	E2F1, CLSPN, CHEK1, H2AFX, CHEK2
cell fate commitment	0.049580485	2.386117709	WNT5A, BMP2, WNT4, SOX12, ROR2, GAS1, SOX9, WNT2B
cerebellar cortex development	0.050200236	8.053147268	KIF14, DIXDC1, EZH2
tube morphogenesis	0.050200236	8.053147268	WNT4, GATA4, FOXA1
negative regulation of retinoic acid receptor signaling pathway	0.050200236	8.053147268	DHRS3, CYP26B1, EZH2
positive regulation of acute inflammatory response	0.050200236	8.053147268	IL6, ADAM8, AOC3
pyrimidine nucleotide metabolic process	0.050200236	8.053147268	DCTD, NME4, DCK
astrocyte cell migration	0.050200236	8.053147268	CCL12, CCL2, MMP14
positive regulation of glucose transport	0.050200236	8.053147268	C3, CLIP3, AOC3
attachment of mitotic spindle microtubules to kinetochore	0.050200236	8.053147268	KIF2C, NDC80, CENPE
maintenance of epithelial cell apical/basal polarity	0.050200236	8.053147268	CRB2, LHX2, LIN7A
lipopolysaccharide-mediated signaling pathway	0.053987305	3.463719255	CCL12, CCL2, TREM2, CD6, CD14
cellular response to ionizing radiation	0.053987305	3.463719255	RAD51AP1, BLM, FIGNL1, ECT2, RAD51
angiogenesis	0.054686328	1.617368489	COL18A1, CYP1B1, CCL2, MMP19, CSPG4, CCDC80, ESM1, MMP14, MMP2, EPHB1, EPHB2, NRCAM, PROK2, CCL12, HMOX1, TGFBI, ADAM8, FN1
retinoid metabolic process	0.055746959	4.521065133	ALDH1A2, DHRS3, ADH1, APOE
oocyte maturation	0.055746959	4.521065133	CCNB1, FBXO5, BRCA2, TRIP13
negative regulation of cAMP biosynthetic process	0.055746959	4.521065133	PTGER3, EDN1, ADRA2A, APLP1
seminiferous tubule development	0.055746959	4.521065133	GATA4, KIF18A, BRIP1, WT1
response to nicotine	0.056712251	2.863341251	LYPD1, DHFR, NTRK1, HMOX1, EDN1, ABAT
positive regulation of mitotic cell cycle	0.059521645	3.355478029	CCNB1, CDK1, FOXA1, BRCA2, BIRC5
response to axon injury	0.059521645	3.355478029	ARG1, CDK1, FOLR1, LGALS1, NTRK1
negative regulation of G1/S transition of mitotic cell cycle	0.059521645	3.355478029	E2F7, SLFN9, EZH2, GPNMB, GSG2
heart development	0.062432073	1.563318499	BMP2, COL3A1, FBN1, EDN1, OXTR, CENPF, SPARC, SOX9, MMP13, MMP2, DNAH5, WT1, ALDH1A2, OSR1, GATA4, CCDC151, PTN, VCAN, ZFPM2
amine metabolic process	0.062594478	7.158353127	CYP1A1, INMT, AOC3
positive regulation of exit from mitosis	0.062594478	7.158353127	BIRC5, UBE2C, CDCA5
toxin metabolic process	0.062594478	7.158353127	CYP1B1, CYP1A1, FMO1
lung vasculature development	0.062594478	7.158353127	LIF, IGF1, ERRFI1
positive regulation of cAMP metabolic process	0.062594478	7.158353127	CHGA, PF4, CXCL10
regulation of chromosome segregation	0.062594478	7.158353127	KIF2C, MKI67, BUB1
negative regulation of histone acetylation	0.062594478	7.158353127	MSX3, BRCA1, TWIST1
negative regulation of collagen biosynthetic process	0.062594478	7.158353127	IL6, CYGB, ERRFI1
mitotic recombination	0.062594478	7.158353127	RAD51B, APITD1, TOP2A
positive regulation of cell-cell adhesion	0.063395946	4.295011876	WNT5A, CCL2, CCR5, TBX18
DNA metabolic process	0.063395946	4.295011876	MKI67, TOP2A, TK1, RAD51
retinal ganglion cell axon guidance	0.063395946	4.295011876	NRCAM, EFNA5, EPHB1, EPHB2
response to hypoxia	0.064639857	1.677739014	BMP2, CCL2, CYP1A1, ARNT2, EDN1, EGLN3, MMP14, TACC3, ADIPOQ, MMP2, ALDH3A1, HMOX1, ABAT, CYGB, LOXL2
positive regulation of release of sequestered calcium ion into cytosol	0.065345321	3.253796876	F2RL3, GPR39, CEMIP, BDKRB1, CXCL10
regulation of neuron apoptotic process	0.065345321	3.253796876	KIF14, GABRB3, EGLN3, ESR2, FAIM2
positive regulation of apoptotic process	0.065961955	1.474407062	BMP2, IL6, CYP1B1, ESR2, FRZB, ECT2, MMP2, WT1, ALDH1A2, SFRP1, BOK, SFRP2, ALDH1A3, HMOX1, SFRP4, PYCARD, PTN, NGFR, CLIP3, IGFBP3, TOP2A, MELK, BARD1
regulation of blood pressure	0.06761544	2.424603479	CALCA, C3AR1, HMOX1, EDN1, COL1A2, NPR3, AOC3
cell-matrix adhesion	0.070406776	2.202570193	ITGB8, COL3A1, MSLN, NID2, ADAMTS12, ADAM8, COL5A3, FN1
response to hormone	0.071096772	2.684382423	MMP19, MSI1, ADRA1B, FHL2, MMP14, TIMP1
positive regulation of phagocytosis	0.071096772	2.684382423	SLC11A1, CCR7, FCGR2B, C3, PYCARD, PTX3
T cell activation	0.071454293	3.158096968	IL6, CD8A, CD276, CD4, CD28
negative regulation of ossification	0.071500509	4.090487501	CALCA, SFRP1, TPH1, SOX9
determination of adult lifespan	0.071500509	4.090487501	MEG3, RAD54B, RAD54L, ANKLE1
Wnt signaling pathway	0.072629655	1.613150001	WNT5A, DIXDC1, TRABD2B, LEF1, CELA1, FRZB, WNT2B, CPZ, DKK2, CCNE1, WNT4, WISP1, SFRP1, SFRP2, SFRP4, ROR2
negative regulation of protein kinase activity	0.074658516	2.03447931	ASPN, CAV3, FLRT2, IL6, PODN, FABP4, DCN, LRRC15, NYX
nucleotide biosynthetic process	0.075890703	6.442517815	DCTD, TYMS, DHFR
anaphase-promoting complex-dependent catabolic process	0.075890703	6.442517815	CDC20, UBE2C, UBE2S
artery smooth muscle contraction	0.075890703	6.442517815	EDN1, CACNA1G, SMPD3
regulation of microtubule polymerization or depolymerization	0.075890703	6.442517815	SKA3, SKA2, SKA1
dorsal/ventral pattern formation	0.076296697	2.629599108	LHX2, EDN1, GAS1, HHIP, CHRD, GLI1
positive regulation of T cell proliferation	0.076425569	2.34883462	CCR7, IL6, CD276, CD4, CD6, PDCD1LG2, CD28
regulation of transcription from RNA polymerase II promoter	0.076809457	1.406427063	HMGB2, EZH2, FHL2, MYBL1, MYBL2, SOX9, WT1, MSX3, GATA4, NR1H4, BATF3, MAFB, SOX12, FOXA1, MLXIPL, BRIP1, LEF1, UCP1, SNAI1, INHBA, BNC1, CKS2, SP6, NFE2L3, ZFP367, IFI204
negative regulation of blood pressure	0.077843726	3.067865626	CALCA, PMCH, ABAT, BDKRB1, ADIPOQ
extracellular matrix disassembly	0.080042563	3.904556251	LAMA1, MMP19, MMP13, MMP12
positive regulation of interleukin-1 beta secretion	0.080042563	3.904556251	WNT5A, CCR7, CCR5, PYCARD
response to cytokine	0.082508761	2.120993519	TYMS, ALDH1A2, COL3A1, SERPINA3I, OXTR, SPARC, SCGB1A1, TIMP1
growth	0.084508052	2.982647136	GDF3, INHBA, BMP2, CCNB2, GDF6
inner ear development	0.085853634	2.277657813	C1QB, BMP2, IGF1, SPARC, LGR5, IFI204, LIN7A
protein complex assembly	0.085853634	2.277657813	CCNB1, CDK1, CLGN, GMNN, MGP, PF4, SOX9
phosphorylation	0.088060531	1.298328754	STK33, NEK2, PASK, FAM20C, DCK, TTK, AURKA, CHEK1, AURKB, CHEK2, ITPKA, EPHB1, EPHB2, TK1, SPEG, BUB1, MASTL, DCLK1, MATK, CDC7, CDK1, CAMK1G, AK5, PBK, GSG2, NEK11, WEE1, NME4, PLK4, PLK1, MAPK4, NTRK1, MAP3K19, ROR2, BUB1B, CIT, MELK
cochlea morphogenesis	0.089002827	3.734792936	WNT5A, FRZB, SOX9, TBX18
positive regulation of cardiac muscle cell proliferation	0.089002827	3.734792936	CCNB1, CDK1, GATA4, ZFPM2
ventricular cardiac muscle cell development	0.089978417	5.856834377	CCNB1, CDK1, FHL2
negative regulation of immune response	0.089978417	5.856834377	FCGR2B, COL3A1, FCRLB
regulation of cellular protein localization	0.089978417	5.856834377	CCNE2, WNT5A, CCNE1
toll-like receptor 4 signaling pathway	0.089978417	5.856834377	NR1H4, CD14, TNIP3
negative regulation of tumor necrosis factor-mediated signaling pathway	0.089978417	5.856834377	TRAIP, ADIPOQ, NR1H4
positive regulation of insulin-like growth factor receptor signaling pathway	0.089978417	5.856834377	IGF1, CDH3, IGFBP3
skin morphogenesis	0.089978417	5.856834377	COL1A2, COL1A1, ERRFI1
hepatocyte differentiation	0.089978417	5.856834377	CYP1A1, E2F7, E2F8
negative regulation of interleukin-1 beta production	0.089978417	5.856834377	MEFV, AQP4, ERRFI1
telomere maintenance via recombination	0.089978417	5.856834377	RAD51C, BRCA2, RAD51
glial cell migration	0.089978417	5.856834377	CCL2, CSPG4, FN1
negative regulation of cysteine-type endopeptidase activity involved in apoptotic process	0.09079692	2.243662921	IL6, SFRP2, RAG1, IGF1, LEF1, BIRC5, NAIP1
negative regulation of interleukin-13 production	0.090859924	21.47505938	LEF1, SCGB1A1
actomyosin contractile ring assembly	0.090859924	21.47505938	KIF23, RACGAP1
positive regulation of antigen processing and presentation of peptide antigen via MHC class II	0.090859924	21.47505938	PYCARD, TREM2
positive regulation of DNA endoreduplication	0.090859924	21.47505938	E2F7, E2F8
ureter urothelium development	0.090859924	21.47505938	OSR1, SOX9
forebrain neuroblast division	0.090859924	21.47505938	LEF1, ASPM
dUMP biosynthetic process	0.090859924	21.47505938	DCTD, DUT
positive regulation of eosinophil migration	0.090859924	21.47505938	CCL24, ADAM8
spindle assembly involved in female meiosis I	0.090859924	21.47505938	FBXO5, AURKA
minus-end-directed vesicle transport along microtubule	0.090859924	21.47505938	KIFC5B, KIFC1
sequestering of TGFbeta in extracellular matrix	0.090859924	21.47505938	FBN1, FBN2
negative regulation of endodermal cell differentiation	0.090859924	21.47505938	COL5A2, COL5A1
regulation of midbrain dopaminergic neuron differentiation	0.090859924	21.47505938	SFRP1, SFRP2
flavone metabolic process	0.090859924	21.47505938	PPARGC1A, UGT1A1
centrosome separation	0.090859924	21.47505938	NEK2, CNTROB
posterior mesonephric tubule development	0.090859924	21.47505938	OSR1, WT1
lymphocyte chemotaxis across high endothelial venule	0.090859924	21.47505938	CCR7, CXCL13
mitotic recombination-dependent replication fork processing	0.090859924	21.47505938	BRCA2, RAD51
cellular response to dexamethasone stimulus	0.091441025	2.902035052	ARG1, IL6, CCL2, SERPINF1, ERRFI1
regulation of mitotic cell cycle	0.091441025	2.902035052	CKS1B, PLK1, CKS2, FBXO5, BIRC5
base-excision repair	0.091441025	2.902035052	NEIL3, LIG1, UNG, POLQ, FEN1
positive regulation of interferon-gamma production	0.093085247	2.477891467	WNT5A, SLC11A1, ULBP1, PYCARD, CD14, RAET1B
transmembrane receptor protein tyrosine kinase signaling pathway	0.093949549	1.932755344	DOK2, NTRK1, CSPG4, ROR2, SHC2, EPHB1, EPHB2, SHC4, MATK
response to ethanol	0.09502357	1.835475161	CDK1, TYMS, CCL2, NTRK1, TNC, ABAT, SPARC, ADIPOQ, CD14, CAR3
sperm motility	0.095891259	2.210667878	LRRC6, CCDC40, CACNA1I, ENO4, INSL6, WT1, DNAH5
middle ear morphogenesis	0.098361064	3.579176564	OSR1, EDN1, PRRX2, GAS1
epithelial tube branching involved in lung morphogenesis	0.098361064	3.579176564	LAMA1, FOXA1, HHIP, SOX9
xenobiotic metabolic process	0.098361064	3.579176564	CYP1B1, CYP26B1, CYP26A1, CYP2B10
cellular response to gamma radiation	0.098361064	3.579176564	H2AFX, CHEK2, DDIAS, RAD51

positive regulation of synaptic transmission, glutamatergic	0.098361064	3.579176564	NTRK1, OXTR, ROR2, NGFR
protein kinase B signaling	0.098635776	2.825665708	CCL12, LINGO1, CCL2, IGF1, SOX9
