# Supplementary material for: Hydrostatic Pressure Controls Angiogenesis Through Endothelial YAP1 During Lung Regeneration
Source: Front Bioeng Biotechnol. 2022 Feb 18;10:823642. doi: 10.3389/fbioe.2022.823642 (PMC8896883; doi:10.3389/fbioe.2022.823642)
Supplement: Supplementary file 5 [file DataSheet6.PDF]

Biological Processes GO Terms	PValue	Fold Enrichment	Genes
actomyosin contractile ring assembly	0.025278564	78.27705628	RACGAP1, KIF23
mitotic recombination-dependent replication fork processing	0.025278564	78.27705628	RAD51, BRCA2
negative regulation of endodermal cell differentiation	0.025278564	78.27705628	COL5A1, COL5A2
sequestering of TGFbeta in extracellular matrix	0.025278564	78.27705628	FBN2, FBN1
regulation of mitotic centrosome separation	0.000950421	58.70779221	CHEK1, NEK2, KIF11
adaptive thermogenesis	0.037678228	52.18470418	UCP1, PPARGC1A
deoxyribonucleotide biosynthetic process	0.037678228	52.18470418	RRM1, RRM2
development of primary male sexual characteristics	0.037678228	52.18470418	SFRP2, WNT5A
embryonic heart tube anterior/posterior pattern specification	0.037678228	52.18470418	BMP2, GATA4
extracellular polysaccharide biosynthetic process	0.037678228	52.18470418	HAS1, HAS2
negative regulation of female gonad development	0.037678228	52.18470418	WT1, ZFPM2
negative regulation of hair follicle development	0.037678228	52.18470418	NGFR, INHBA
odontoblast differentiation	0.037678228	52.18470418	LEF1, FAM20C
prostate gland stromal morphogenesis	0.037678228	52.18470418	FOXA1, IGF1
regulation of endothelial tube morphogenesis	0.037678228	52.18470418	CXCL10, ADAMTS12
renal vesicle induction	0.037678228	52.18470418	SOX9, WNT4
retinal rod cell differentiation	0.037678228	52.18470418	SOX9, PTN
spindle checkpoint	0.037678228	52.18470418	BIRC5, AURKB
tendon development	0.037678228	52.18470418	COL5A1, COL11A1
regulation of attachment of spindle microtubules to kinetochore	6.84663E-05	44.72974644	RACGAP1, SPAG5, NEK2, ECT2
collagen biosynthetic process	0.002336371	39.13852814	COL1A1, COL5A1, ARG1
intramembranous ossification	0.002336371	39.13852814	COL1A1, CTSK, MMP2
mitotic spindle midzone assembly	0.002336371	39.13852814	RACGAP1, KIF23, AURKB
cellular response to xenobiotic stimulus	0.049920831	39.13852814	MCM7, E2F1
epithelial cell maturation involved in prostate gland development	0.049920831	39.13852814	FOXA1, ESR2
mitotic centrosome separation	0.049920831	39.13852814	KIF11, AURKA
negative regulation of membrane potential	0.049920831	39.13852814	IL6, PTN
positive regulation of apoptotic cell clearance	0.049920831	39.13852814	C3, CCL2
regulation of postsynaptic density protein 95 clustering	0.049920831	39.13852814	CDH2, DBN1
replication-born double-strand break repair via sister chromatid exchange	0.049920831	39.13852814	BLM, RAD51
response to hydrostatic pressure	0.049920831	39.13852814	NTRK1, COL18A1
thyroid-stimulating hormone-secreting cell differentiation	0.049920831	39.13852814	BMP2, WNT4
tricuspid valve formation	0.049920831	39.13852814	GATA4, ZFPM2
ventricular cardiac muscle tissue development	0.049920831	39.13852814	COL14A1, GATA4
DNA unwinding involved in DNA replication	5.04353E-06	39.13852814	TOP2A, RAD51, MCM7, MCM6, MCM2
extracellular fibril organization	7.84646E-06	35.58048013	MFAP5, MFAP4, COL3A1, COL5A1, LTBP2
mitotic sister chromatid segregation	5.21718E-12	34.03350273	DSN1, KIF18B, ESPL1, SPAG5, PLK1, NEK2, CENPA, NDC80, CIT, MAD2L1
DNA damage induced protein phosphorylation	0.003243533	33.54730983	CHEK2, CHEK1
positive regulation of male gonad development	0.003243533	33.54730983	WT1, SOX9, ZFPM2
prostate gland epithelium morphogenesis	1.16523E-05	32.61544012	FOXA1, MMP2, TNC, IGF1, ESR2
complement activation, alternative pathway	0.000228192	31.31082251	CFD, C3, CFP, CFB
eosinophil chemotaxis	0.000228192	31.31082251	CCL24, CCL11, CCL2, CCR3
bone mineralization involved in bone maturation	0.062008352	31.31082251	BMP2, IGF1
cellular response to camptothecin	0.062008352	31.31082251	BLM, RAD51
cellular response to cisplatin	0.062008352	31.31082251	RAD51, HMOX1
chordate embryonic development	0.062008352	31.31082251	BRCA1, BRCA2
eye morphogenesis	0.062008352	31.31082251	COL5A1, COL5A2
homologous chromosome segregation	0.062008352	31.31082251	ESPL1, PLK1
mast cell chemotaxis	0.062008352	31.31082251	CCL11, CCR3
metaphase/anaphase transition of mitotic cell cycle	0.062008352	31.31082251	BUB1B, CIT
mitotic spindle checkpoint	0.062008352	31.31082251	STIL, BUB1
mitral valve formation	0.062008352	31.31082251	GATA4, ZFPM2
peripheral nervous system axon regeneration	0.062008352	31.31082251	MMP2, TNC
positive regulation of meiotic nuclear division	0.062008352	31.31082251	WNT5A, WNT4
positive regulation of odontogenesis	0.062008352	31.31082251	BMP2, EDN1
protein localization to chromosome, centromeric region	0.062008352	31.31082251	BUB1B, CENPA
regulation of vascular endothelial growth factor production	0.062008352	31.31082251	IL6, CCL2
skeletal muscle satellite cell maintenance involved in skeletal muscle regeneration	0.062008352	31.31082251	IGF1, EZH2
tube morphogenesis	0.004288539	29.3538961	FOXA1, GATA4, WNT4
bone trabecula formation	0.005467765	26.09235209	FBN2, COL1A1, MMP2
regulation of chromosome segregation	0.005467765	26.09235209	KIF2C, MKI67, BUB1
cellular response to insulin-like growth factor stimulus	0.073942748	26.09235209	CCNA2, IGF1
chromosome breakage	0.073942748	26.09235209	BRCA1, BRCA2
chromosome organization involved in meiotic cell cycle	0.073942748	26.09235209	RAD51, CCNE1
hyaluronan biosynthetic process	0.073942748	26.09235209	HAS1, HAS2
negative regulation of mammary gland epithelial cell proliferation	0.073942748	26.09235209	BRCA2, ETV4
negative regulation of mesenchymal cell proliferation	0.073942748	26.09235209	WNT5A, PTN
negative regulation of steroid biosynthetic process	0.073942748	26.09235209	BMP2, WNT4
negative regulation of stress-activated MAPK cascade	0.073942748	26.09235209	PBK, FOXM1
proteoglycan metabolic process	0.073942748	26.09235209	BMP2, COL11A1
regulation of branching involved in mammary gland duct morphogenesis	0.073942748	26.09235209	WNT5A, ETV4
regulation of metanephric nephron tubule epithelial cell differentiation	0.073942748	26.09235209	LIF
retinoic acid biosynthetic process	0.073942748	26.09235209	ALDH1A3, ALDH1A2
protein localization to kinetochore	0.006777658	23.48311688	CDK1, BUB1B, AURKB
DNA replication initiation	4.40938E-07	22.83080808	MCM7, CCNE1, MCM3, CDC7, MCM5, MCM6, MCM2
negative regulation of hormone secretion	0.000666602	22.36487322	IL6, EDN1, ADIPOQ, LIF
positive regulation of protein import into nucleus, translocation	0.000666602	22.36487322	IL6, CDK1, IGF1, BRCA1

cellular response to gonadotropin stimulus	0.085725951	22.36487322	WT1, GATA4
convergent extension involved in axis elongation	0.085725951	22.36487322	SFRP2, WNT5A
extracellular matrix assembly	0.085725951	22.36487322	HAS1, HAS2
integrin activation	0.085725951	22.36487322	COL16A1, FN1
meiotic spindle organization	0.085725951	22.36487322	ESPL1, AURKA
mesenchymal to epithelial transition	0.085725951	22.36487322	WT1, WNT4
negative regulation of membrane protein ectodomain proteolysis	0.085725951	22.36487322	IL10, TIMP1
negative regulation of vascular smooth muscle cell proliferation	0.085725951	22.36487322	IL10, HMOX1
positive regulation of cell proliferation in bone marrow	0.085725951	22.36487322	IL6, LEF1
positive regulation of mitotic cell cycle spindle assembly checkpoint	0.085725951	22.36487322	NDC80, MAD2L1
positive regulation of ubiquitin protein ligase activity	0.085725951	22.36487322	CDC20, PLK1
protein localization to chromatin	0.085725951	22.36487322	PLK1, EZH2
glial cell migration	0.008214733	21.34828808	FN1, CCL2, CSPG4
positive regulation of insulin-like growth factor receptor signaling pathway	0.008214733	21.34828808	CDH3, IGFBP3, IGF1
protein heterotrimerization	8.50671E-05	20.59922534	COL1A1, COL1A2, COL6A2, COL6A1, ADIPOQ
collagen fibril organization	1.13366E-09	20.07104007	COL1A1, ADAMTS2, COL3A1, COL1A2, SFRP2, COL5A1, COL14A1, LUM, COL11A1, COL5A2
collagen catabolic process	1.11233E-05	19.56926407	ADAMTS2, MMP14, MMP13, CTSK, MMP2, MMP10
DNA damage response, signal transduction by p53 class mediator resulting in transcription of p21 class mediator	0.009775571	19.56926407	CHEK2, FOXM1, BRCA2
lung epithelial cell differentiation	0.009775571	19.56926407	FOXA1, SOX9
spindle organization	0.001006449	19.56926407	SPAG5, KIF11, AURKB, AURKA
astrocyte cell migration	0.097359867	19.56926407	MMP14, CCL2
atrioventricular canal development	0.097359867	19.56926407	HAS2, GATA4
attachment of mitotic spindle microtubules to kinetochore	0.097359867	19.56926407	KIF2C, NDC80
cellular aldehyde metabolic process	0.097359867	19.56926407	ALDH3A1, ALDH1A3
cellular response to hydroxyurea	0.097359867	19.56926407	BLM, RAD51
cerebellar cortex development	0.097359867	19.56926407	KIF14, EZH2
DNA topological change	0.097359867	19.56926407	TOP2A, HMGB2
lung lobe morphogenesis	0.097359867	19.56926407	LIF, IGF1
maternal process involved in parturition	0.097359867	19.56926407	EDN1, CCL2
negative regulation of smooth muscle cell apoptotic process	0.097359867	19.56926407	EDN1, IGF1
paraxial mesoderm formation	0.097359867	19.56926407	WNT5A, LEF1
response to yeast	0.097359867	19.56926407	IL6, PTX3
Sertoli cell differentiation	0.097359867	19.56926407	GATA4, SOX9
cytokinesis	1.70474E-08	19.04036504	PRC1, INCENP, PLK1, BIRC5, KIF23, KIF20A, ECT2, BRCA2, CIT
G2/M transition of mitotic cell cycle	1.58531E-07	18.97625607	CHEK2, PLK1, CHEK1, BIRC5, CDC25C, SKP2, FOXM1, CIT
cellular response to fatty acid	0.000128755	18.63739435	CCNB1, EDN1, UCP1, E2F1, NR1H4
negative regulation of fibroblast growth factor receptor signaling pathway	0.011456819	18.06393606	NGFR, WNT5A, WNT4
notochord development	0.011456819	18.06393606	STIL, SOX9
response to molecule of bacterial origin	0.011456819	18.06393606	IL10, CD14, CXCL2
endodermal cell differentiation	2.04798E-05	17.39490139	MMP14, COL11A1, MMP2, COL6A1, FN1, INHBA
mitotic spindle assembly checkpoint	0.00143927	17.39490139	PLK1, BUB1B, BUB1, MAD2L1
cellular response to platelet-derived growth factor stimulus	0.000186731	17.01675136	CCNA2, HAS1, CCL2, HAS2, PTN
mitotic spindle organization	2.46748E-05	16.77365492	STIL, CCNB1, RCC1, AURKB, NDC80, AURKA
cell activation	0.013255189	16.77365492	FN1, IGF1, TIMP1
cellular response to follicle-stimulating hormone stimulus	0.013255189	16.77365492	GATA4, INHBA, PPARGC1A
microtubule depolymerization	0.013255189	16.77365492	KIF18B, KIF14, KIF2C
tissue remodeling	0.013255189	16.77365492	MMP14, MMP2, CSPG4
positive regulation of cytokinesis	3.29719E-06	16.60422406	RACGAP1, KIF14, KIF23, KIF20B, ECT2, AURKB, CIT
estrous cycle	0.001693184	16.47938027	HAS1, HAS2, PTN, ESR2
mitotic cytokinesis	3.50446E-05	15.65541126	ANLN, RACGAP1, PLK1, KIF23, KIF20A, CENPA
atrioventricular valve morphogenesis	0.015167455	15.65541126	BMP2, GATA4, ZFPM2
complement activation	0.015167455	15.65541126	C3, C4B, CFB
DNA metabolic process	0.001973402	15.65541126	TOP2A, RAD51, TK1, MKI67
ERK1 and ERK2 cascade	0.000305967	15.05328005	CCL11, IGF1, SOX9, IQGAP3, CCR3
G2 DNA damage checkpoint	0.002280844	14.90991548	PLK1, CHEK1, BRCA1, DTL
chromosome condensation	0.017190456	14.67694805	TOP2A, CDK1, NCAPD2
negative regulation of smooth muscle cell migration	0.017190456	14.67694805	IGFBP3, ADIPOQ, PPARGC1A
positive regulation of cartilage development	0.017190456	14.67694805	BMP2, WNT5A, SOX9
positive regulation of cellular protein metabolic process	0.017190456	14.67694805	UHRF1, ADIPOQ, INHBA
Sertoli cell development	0.017190456	14.67694805	NTRK1, WT1, SOX9
positive regulation of epithelial to mesenchymal transition	6.56517E-05	13.81359817	COL1A1, BMP2, LEF1, SNAI1, TWIST1, EZH2
response to gamma radiation	6.56517E-05	13.81359817	CXCL10, FANCD2, CHEK2, CCL2, BRCA2, CXCL2
cellular response to interleukin-6	0.01932109	13.81359817	SELP, CCL2, PPARGC1A
endocardial cushion morphogenesis	0.01932109	13.81359817	BMP2, TWIST1, SOX9
neural crest cell development	0.01932109	13.81359817	EDN1, ALDH1A2, SOX9
non-canonical Wnt signaling pathway	0.01932109	13.81359817	SFRP4, WNT5A, WNT4
positive regulation of cardiac muscle cell proliferation	0.002980823	13.61340109	CCNB1, CDK1, GATA4, ZFPM2
liver regeneration	0.000471776	13.49604419	IL10, HMOX1, GLI1, EZH2, AURKA
bone mineralization	7.58052E-05	13.41892393	BMP2, MMP13, GPNMB, ALOX15, PTN, ASPN
mitotic spindle assembly	7.58052E-05	13.41892393	CDC20, TPX2, CHEK2, BIRC5, NEK2, KIF11
chondrocyte development	0.021556318	13.04617605	SFRP2, COL11A1, SOX9
trophoblast giant cell differentiation	0.021556318	13.04617605	PLK4, SNAI1, LIF
response to activity	2.68154E-06	12.77992756	NTRK1, IL10, EDN1, ADIPOQ, CDK1, CCL2, PTN, PPARGC1A
epithelial to mesenchymal transition	0.000612685	12.62533166	BMP2, LEF1, SNAI1, SOX9, WNT4
negative regulation of neuron death	5.09516E-07	12.58024119	NTRK1, IL6, GPNMB, MDK, SERPINF1, IGF1, APOE, PPARGC1A, ESR2
negative regulation of extrinsic apoptotic signaling pathway via death domain receptors	0.003799527	12.524329	SFRP2, HMGB2, HMOX1, BRCA1
response to vitamin A	0.023893157	12.3595352	ARG1, ALDH1A2, GATA4

seminiferous tubule development	0.023893157	12.3595352	BRIP1, WT1, GATA4
chromosome segregation	9.72897E-11	12.31324481	TOP2A, SPAG5, KIF11, BRCA1, PMF1, NDC80, DSN1, ESPL1, INCENP, BIRC5, RCC1, KIF2C, NEK2, BUB1
positive regulation of mitotic cell cycle	0.000693282	12.23079004	FOXA1, CCNB1, CDK1, BIRC5, BRCA2
cellular response to estradiol stimulus	0.000781103	11.86016004	IL10, CCNA2, IL6, MMP2, PPARGC1A
face development	0.026328685	11.74155844	ALDH1A3, ALDH1A2, WNT5A
positive regulation of cell-cell adhesion	0.026328685	11.74155844	WNT5A, CCL2, CCR5
protein localization to centrosome	0.026328685	11.74155844	STIL, SPAG5, AURKA
regulation of mitotic nuclear division	0.026328685	11.74155844	RCC1, MKI67, KIF20B
positive regulation of cell-substrate adhesion	0.000184777	11.18243661	CCDC80, ALOX15, SPP1, PTN, FBLN2, DBN1
response to X-ray	0.028860037	11.18243661	BLM, RAD51, BRCA2
response to mechanical stimulus	1.28689E-06	11.18243661	COL3A1, MMP14, CCNB1, MMP2, TNC, CCL2, BDKRB1, GATA4, DCN
wound healing	2.56516E-09	10.82555034	SPARC, ALOX15, WNT5A, TNC, FN1, CXCL2, DCN, MMP12, COL1A1, IL6, COL3A1, CDH3, TIMP1
negative regulation of DNA binding	0.005815257	10.79683535	LEF1, E2F1, HMOX1, NEK2
cellular response to interleukin-1	6.89478E-08	10.76309524	CCL24, IL6, EDN1, CCL8, CCL11, FN1, CCL2, HAS2, SOX9, ADAMTS12, CXCL2
centrosome duplication	0.031484403	10.67414404	STIL, BRCA1, BRCA2
extracellular matrix disassembly	0.031484403	10.67414404	MMP12, MMP13, LAMA1
positive regulation of interleukin-1 beta secretion	0.031484403	10.67414404	WNT5A, CCR7, CCR5
positive regulation of leukocyte migration	0.031484403	10.67414404	SELP, CCL2, BDKRB1
positive regulation of endothelial cell proliferation	2.07465E-06	10.51482846	IL10, CCL24, BMP2, CCL11, ARG1, WNT5A, HMGB2, CCL2, CCR3
negative regulation of smooth muscle cell proliferation	0.001340777	10.29961267	IGFBP3, ADIPOQ, HMOX1, PPARGC1A, ESR2
cellular response to transforming growth factor beta stimulus	1.19657E-05	10.26584345	COL1A1, EDN1, ARG1, WNT5A, SOX9, PPARGC1A, DBN1, WNT4
positive regulation of protein localization to nucleus	0.03419903	10.21005082	NGFR, CDK1, LIF
DNA replication	4.86009E-11	10.1823813	CDT1, RMI2, RRM1, BLM, RRM2, MCM7, PRIM1, BRCA1, CHAF1B, CHAF1A, MCM3, MCM5, MCM6, DTL, POLE, MCM2
cellular response to gamma radiation	0.037001218	9.784632035	RAD51, CHEK2
epithelial tube branching involved in lung morphogenesis	0.037001218	9.784632035	FOXA1, LAMA1, SOX9
meiotic nuclear division	0.037001218	9.784632035	RAD51, MKI67, AURKA
positive regulation of vascular endothelial growth factor production	0.037001218	9.784632035	C3, GATA4, BRCA1
regulation of cytokinesis	0.037001218	9.784632035	BRCA2, AURKB, AURKA
replication fork processing	0.037001218	9.784632035	MMS22L, BLM, RAD51
response to axon injury	0.007676412	9.784632035	NTRK1, LGALS1, ARG1, CDK1
osteoblast differentiation	2.03025E-09	9.698042371	IGFBP3, LEF1, TNC, TWIST1, IGF1, GLI1, COL1A1, VCAN, BMP2, GPNMB, CREB3L1, COL6A1, SNAI1, SPP1
lymphocyte chemotaxis	0.008365943	9.488128034	CCL24, CCL8, CCL11, CCL2
response to wounding	9.2441E-05	9.44723093	NGFR, IL6, FABP5, MDK, CCL2, GLI1, AURKA
mitotic nuclear division	1.32245E-21	9.325425477	ERCC6L, CCNF, BUB1B, KIF11, PMF1, AURKB, AURKA, CDC20, CCNB1, DSN1, TUBB3, CHEK2, RCC1, NEK2, BUB1, SPAG5, PLK1, KIF23, CDC25C, NDC80, CIT, CCNA2, ANLN, TPX2, KIF18B, ESPL1, INCENP, CDK1, BIRC5, NCAPD2, KIF2C, KIF20B, MAD2L1
cellular response to tumor necrosis factor	1.57995E-08	9.250924833	CCL24, EDN1, CCL11, BRCA1, ADAMTS12, COL1A1, IL6, CCL8, FABP4, CCL2, HAS2, PPARGC1A, DBN1
negative regulation of tumor necrosis factor production	0.002133271	9.101983288	IL10, GPNMB, ADIPOQ, NR1H4, TWIST1
positive regulation of collagen biosynthetic process	0.042857747	9.031968032	CREB3L1, CCL2, WNT4
positive regulation of JAK-STAT cascade	0.042857747	9.031968032	IL10, IL6, IL5
positive regulation of inflammatory response	0.000147192	8.697450697	CCL24, CCL8, CCL11, FABP4, WNT5A, CCL2, CCR5
adrenal gland development	0.045906954	8.697450697	MDK, WT1, WNT4
cellular response to interleukin-4	0.045906954	8.697450697	ARG1, LEF1, MCM2
maternal process involved in female pregnancy	0.045906954	8.697450697	ARG1, LIF, CCL2
microtubule bundle formation	0.045906954	8.697450697	PRC1, PLK1, KIF20A
negative regulation of angiogenesis	3.58906E-05	8.697450697	NGFR, CXCL10, SPARC, SERPINF1, LIF, CCL2, PTN, DCN
response to amino acid	0.010647465	8.697450697	IL6, EDN1, ARG1, CCL2
cartilage development	9.62595E-06	8.591384226	BMP2, EDN1, MMP13, SFRP2, LUM, COL11A1, WNT5A, TIMP1, SOX9
chemokine-mediated signaling pathway	0.000660886	8.53931523	CXCL10, CCL24, CCL8, CCL11, CCL2, CXCL2
cellular response to dexamethasone stimulus	0.011480041	8.462384462	IL6, ARG1, SERPINF1, CCL2
positive regulation of bone mineralization	0.011480041	8.462384462	FBN2, BMP2, FAM20C, WNT4
cellular response to retinoic acid	0.000175196	8.42983683	COL1A1, ALDH1A2, SERPINF1, TNC, CCL2, SOX9
positive regulation of fibroblast proliferation	0.000175196	8.42983683	CCNA2, NGFR, CCNB1, WNT5A, E2F1, FN1, IGF1
positive regulation of neutrophil chemotaxis	0.049033452	8.386827458	EDN1, CCR7, CXCL2
mammary gland development	0.012349121	8.239690134	LEF1, IGF1, SOX9, BRCA2
ossification	3.70053E-06	8.153860029	MMP14, BMP2, SPARC, COL11A1, COL5A2, ALOX15, SPP1, TWIST1, SOX9, PTN
negative regulation of fat cell differentiation	0.003201275	8.153860029	WNT5A, ADIPOQ, E2F1, ZFPM2, DLK1
skin development	0.000844057	8.097626511	COL1A1, NGFR, ADAMTS2, COL3A1, COL5A1, COL5A2
biomineral tissue development	0.0522348	8.097626511	SPP1, FAM20C, ASPN
cardiac muscle tissue development	0.0522348	8.097626511	ALDH1A2, GATA4, ZFPM2
response to cadmium ion	0.0522348	8.097626511	SPARC, ARG1, CDK1
spindle assembly	0.0522348	8.097626511	TPX2, RCC1, KIF11
microtubule-based movement	6.0345E-05	8.028416028	KIF18B, KIF14, KIF23, KIF2C, KIF20A, KIF11, KIF20B, KIF15
cell division	1.66287E-22	7.953283793	ERCC6L, CCNF, KIF14, BUB1B, KIF11, PMF1, AURKB, AURKA, CDC20, CCNB1, DSN1, RACGAP1, CHEK2, RCC1, NEK2, ECT2, BUB1, SPAG5, PLK1, CDC7, KIF23, CDC25C, NDC80, CIT, CCNA2, ANLN, TPX2, KIF18B, CCNE1, PRC1, INCENP, CDK1, BIRC5, MCM5, NCAPD2, KIF2C, KIF20B, MAD2L1
neutrophil chemotaxis	0.000243754	7.941150637	CCL24, CCL8, CCL11, ITGAM, SPP1, CCL2, CXCL2
DNA damage checkpoint	0.055508606	7.827705628	CHEK2, CHEK1, E2F1
endochondral ossification	0.055508606	7.827705628	COL1A1, MMP14, MMP13
response to electrical stimulus	0.055508606	7.827705628	NTRK1, IL6, CD14
synapsis	0.055508606	7.827705628	BRIP1, FANCD2, CCNE1
monocyte chemotaxis	0.014197714	7.827705628	CCL24, CCL8, CCL11, CCL2
male gonad development	1.66241E-06	7.68792517	BRIP1, RRM1, MMP14, SFRP2, WT1, WNT5A, HMGB2, GATA4, INHBA, SOX9, WNT4
lung development	5.32233E-07	7.575198995	FOXA1, ADAMTS2, MMP14, SPARC, ARG1, ALDH1A2, WNT5A, LIF, IGF1, PTN, GLI1, ZFPM2
G1/S transition of mitotic cell cycle	0.001144021	7.575198995	CCNE1, RCC1, INHBA, SKP2, IQGAP3, POLE
cellular response to ionizing radiation	0.058852528	7.575198995	BLM, RAD51, ECT2
lipopolysaccharide-mediated signaling pathway	0.058852528	7.575198995	CCL2, TREM2, CD14
regulation of neurogenesis	0.058852528	7.575198995	HMGB2, EZH2
bone morphogenesis	0.062264269	7.338474026	MMP13, SFRP2, HAS2

response to glucocorticoid	0.000384338	7.305858586	IL10, ALDH3A1, IL6, SPARC, MDK, ADIPOQ, CXCL2
positive regulation of DNA replication	0.017249432	7.28158663	IL6, CDK1, IGF1, GLI1
intrinsic apoptotic signaling pathway in response to DNA damage	0.004904023	7.247875581	CHEK2, E2F1, HMOX1, BRCA1, BRCA2
activation of protein kinase activity	0.06574158	7.116096025	TPX2, KIF14, ECT2
regulation of neuron apoptotic process	0.06574158	7.116096025	EGLN3, KIF14, ESR2
cell chemotaxis	0.000475006	7.024864025	CXCL10, CCL8, LEF1, HMGB2, CCL2, CXCL2, EPHB1
organ regeneration	0.005584399	6.989022882	CCNA2, CDK1, LIF, CCL2, MKI67
negative regulation of canonical Wnt signaling pathway	4.76393E-05	6.906799083	SFRP4, BMP2, SFRP2, CDH2, WNT5A, LEF1, SOX9, GLI1, WNT4
cellular response to interferon-gamma	0.001732915	6.906799083	CCL24, EDN1, CCL8, CCL11, WNT5A, CCL2
positive regulation of osteoblast differentiation	0.001732915	6.906799083	FBN2, BMP2, SFRP2, FAM20C, IGF1, WNT4
brown fat cell differentiation	0.069282259	6.906799083	FABP4, ADIPOQ, UCP1
mitotic metaphase plate congression	0.069282259	6.906799083	CCNB1, KIF14, KIF2C
stem cell differentiation	0.069282259	6.906799083	LIF, A2M, ETV4
cellular response to hydrogen peroxide	0.005947051	6.866408445	IL6, ARG1, CDK1, ECT2, EZH2
response to heat	0.005947051	6.866408445	IL6, CCL2, IGF1, CD14, CXCL2
cell cycle	2.52683E-28	6.756814304	ERCC6L, MCM7, CCNF, BUB1B, BRCA1, KIF11, FOXM1, BRCA2, MKI67, CDC20, CHAF1B, CHAF1A, CHEK2, CHEK1, RCC1, NEK2, DLGAP5, KIF23, CDC25C, CIT, CCNA2, CCNE1, INCENP, FANCD2, MCM3, BIRC5, MCM5, KIF2C, MCM6, KIF20B, MCM2, UHRF1, PMF1, AURKB, AURKA, CCNB1, DSN1, RACGAP1, E2F1, ECT2, BUB1, CDT1, SPAG5, PLK1, CDC7, NDC80, ANLN, TPX2, KIF18B, PRC1, CDK1, NCAPD2, MAD2L1
chemotaxis	2.00391E-05	6.633648837	CXCL10, CCL24, CCL8, CCL11, HMGB2, CCL2, CCR7, CCR5, CXCL2, CCR3
positive regulation of tumor necrosis factor production	0.00671867	6.633648837	TWIST1, CCL2, CCR7, CD14, CCR5
cellular response to glucose stimulus	0.002098946	6.614962502	LGALS1, CMA1, SERPINF1, GATA4, IGF1, PPARGC1A
skeletal system development	6.70631E-05	6.584051463	COL1A1, COL3A1, VCAN, EDN1, COL1A2, COL5A2, FAM20C, SOX9, FBN1
branching involved in ureteric bud morphogenesis	0.023086701	6.523088023	BMP2, WT1, SOX9, WNT4
branching morphogenesis of an epithelial tube	0.076545127	6.523088023	MMP14, IGF1, WNT4
cellular response to epidermal growth factor stimulus	0.076545127	6.523088023	COL1A1, MCM7, SOX9
positive regulation of angiogenesis	2.44997E-05	6.469178205	C3, CCL24, CCL11, SFRP2, CMA1, WNT5A, HMOX1, GATA4, BRCA1, CCR3
cell development	0.024367253	6.389963778	LEF1, GATA4, IGF1, INHBA
aging	3.4006E-07	6.334559468	NTRK1, IL10, ARG1, SERPINF1, AURKB, DCN, ALDH3A1, IL6, COL3A1, KRT14, CCL2, TIMP1, APOE, PPARGC1A
embryonic limb morphogenesis	0.008454593	6.212464784	FBN2, ALDH1A2, WNT5A, LEF1, TWIST1
metanephros development	0.084036132	6.179767601	WT1, WNT4, FBN1
mitotic cell cycle	0.084036132	6.179767601	RRM1, KIF18B, AURKA
protein kinase B signaling	0.084036132	6.179767601	CCL2, IGF1, SOX9
canonical Wnt signaling pathway	0.00095726	6.156622404	SFRP4, CDH3, WNT5A, LEF1, GATA4, GLI1, WNT4
neural tube development	0.027041368	6.139376963	STIL, SFRP2, ALDH1A2, WNT5A
cellular response to hypoxia	0.000346113	6.079771361	CCNA2, CCNB1, EDN1, E2F1, TWIST1, HMOX1, PTN, PPARGC1A
cellular response to heat	0.087862142	6.021312021	CXCL10, HMOX1, MKI67
regulation of cell differentiation	0.087862142	6.021312021	HMGB2, LIF, SOX9
embryo development	0.003347921	5.945092882	BMP2, RACGAP1, WNT5A, BIRC5, BUB1, DLK1
cellular response to calcium ion	0.029865766	5.907702361	EDN1, WNT5A, ALOX15, ECT2
positive regulation of smooth muscle cell proliferation	0.00353484	5.870779221	IL6, EDN1, HMOX1, IGF1, SKP2, PPARGC1A
face morphogenesis	0.091739218	5.870779221	COL1A1, MMP2, LEF1
patterning of blood vessels	0.091739218	5.870779221	EDN1, SFRP2, LEF1
positive regulation of axon extension	0.091739218	5.870779221	FN1, APOE, DBN1
positive regulation of cell cycle	0.091739218	5.870779221	IL10, CCNB1, BIRC5
response to cold	0.091739218	5.870779221	CXCL10, IL6, PPARGC1A
regulation of cell proliferation	3.59517E-08	5.8621584	EGLN3, SPARC, TNC, IGF1, BRCA1, FOXM1, BRCA2, CXCL2, ESR2, NR3C2, CXCL10, IL6, SFRP2, CHEK1, SOX9, EZH2
negative regulation of cysteine-type endopeptidase activity involved in apoptotic process	0.010460705	5.841571364	IL6, SFRP2, LEF1, BIRC5, IGF1
positive regulation of canonical Wnt signaling pathway	0.010460705	5.841571364	COL1A1, SFRP4, SFRP2, WNT4
positive regulation of cell migration	3.42547E-07	5.784018937	COL18A1, CCL24, EDN1, CCL11, MMP2, LEF1, FN1, IGF1, COL1A1, CXCL10, MMP14, BMP2, GPNMB, SNAI1, HAS2
double-strand break repair via homologous recombination	0.011006192	5.755665903	MMS22L, BLM, RAD51, BRCA1, BRCA2
vasculogenesis	0.011006192	5.755665903	WT1, HAS2, ZFPM2, FOXM1
negative regulation of protein phosphorylation	0.004357896	5.591218306	CCNB1, CD109, IGFBP3, PBK, BDKRB1, PPARGC1A
regulation of cell adhesion	0.099638985	5.591218306	LAMA1, KIF14, SOX9
DNA recombination	0.004583303	5.525439267	BLM, RAD51, EXO1, HMGB2, BRCA1, BRCA2
blood vessel development	0.012751569	5.512468752	COL1A1, COL3A1, COL1A2, COL5A1, ALDH1A2
ovarian follicle development	0.035962216	5.493126756	MMP14, MMP2, INHBA, ESR2
response to ionizing radiation	0.035962216	5.493126756	RRM1, RAD51, KRT14, BRCA1
negative regulation of epithelial cell proliferation	0.013370332	5.435906686	SFRP2, WNT5A, SOX9, PTN, ESR2
positive regulation of peptidyl-tyrosine phosphorylation	0.001840763	5.425142514	IL6, IL5, ADIPOQ, LIF, TREM2, CSPG4, IGF1
positive regulation of ERK1 and ERK2 cascade	5.35438E-06	5.412775168	NTRK1, CCL24, CCL11, ALOX15, TREM2, GATA4, ESR2, C3, IL6, BMP2, CCL8, GPNMB, CCL2
negative regulation of inflammatory response	0.005058796	5.398417674	IL10, SERPINF1, ADIPOQ, PBK, NR1H4, APOE
positive regulation of GTPase activity	0.000106508	5.361442211	NTRK1, CCL24, CCL8, CCL11, WNT5A, CCL2, CCR7, ECT2, WNT4, EZH2
positive regulation of MAPK cascade	0.002135776	5.268648019	NGFR, IL6, BMP2, CDH2, IGFBP3, LIF, IGF1
inflammatory response	5.65828E-10	5.233640391	IL10, NGFR, CCL24, CCL11, HMGB2, NR1H4, CXCL2, C3, C4B, SELP, CXCL10, IL6, BMP2, CCL8, IL5, SPP1, CCL2, BDKRB1, CCR7, CSPG4, CD14, CCR5, CCR3
cellular response to drug	0.015340133	5.218470418	BLM, EDN1, CHEK2, ADIPOQ, CCL2
response to estrogen	0.015340133	5.218470418	MMP14, MMP2, HMOX1, GATA4, BRCA1
activation of MAPK activity	0.01603515	5.149806334	BMP2, WNT5A, CSPG4, IGF1, IQGAP3
positive regulation of epithelial cell proliferation	0.01603515	5.149806334	IL6, WNT5A, TWIST1, IGF1, SOX9
positive regulation of sequence-specific DNA binding transcription factor activity	0.002465154	5.120928915	IL10, FOXA1, IL6, EDN1, IL5, PPARGC1A, ESR2
response to drug	2.51448E-09	5.079926956	NTRK1, IL10, COL18A1, MCM7, ARG1, MMP2, HMGB2, ATP1A3, GATA4, INHBA, PTN, COL1A1, ALDH3A1, IL6, CCNB1, RAD51, CDH3, LGALS1, SFRP2, MDK, CDK1, PPARGC1A
heart looping	0.044409561	5.050132663	STIL, ALDH1A2, WNT5A, GATA4
regulation of protein phosphorylation	0.044409561	5.050132663	RAD51, FN1, IGF1, EZH2
cell-matrix adhesion	0.017483778	5.017760018	COL3A1, COL5A3, FN1, ADAMTS12, MSLN
cellular response to oxidative stress	0.046207309	4.969971827	NGFR, FANCD2, PYCR1, PPARGC1A
cellular response to growth factor stimulus	0.04804061	4.892316017	NTRK1, BMP2, SPARC, TWIST1
heart morphogenesis	0.04804061	4.892316017	COL5A1, ALDH1A2, COL11A1, GATA4
positive regulation of phosphatidylinositol 3-kinase signaling	0.04804061	4.892316017	SELP, IGF1, SOX9, DCN

response to organic cyclic compound	0.019011525	4.892316017	ALDH3A1, MMP14, LUM, CDK1, MKI67
extracellular matrix organization	0.003381136	4.806485912	COL18A1, CCDC80, LAMA1, ELN, COL5A3, FN1, SOX9
protein complex assembly	0.051812835	4.744064017	CCNB1, CDK1, SOX9, CLGN
response to ethanol	0.003842914	4.683242683	NTRK1, SPARC, ADIPOQ, TNC, CDK1, CCL2, CD14
cell adhesion	2.42974E-11	4.680483777	COL18A1, COL15A1, ITGAM, COL16A1, LAMA1, COL14A1, TROAP, TNC, MSLN, ISLR, CDH3, CDH2, GPNMB, HAS1, SPP1, CLCA2, HAS2, EPHB1, FN1, SELP, MFAP4, FERMT1, VCAN, COL5A1, COL6A2, COL6A1, ADAM12, COL6A6, COL6A5
retina development in camera-type eye	0.022308667	4.659348588	RRM1, LAMA1, SERPINF1, LIF, SOX9
response to estradiol	0.009406264	4.650122155	COL1A1, FOXA1, ALDH1A2, PTN, CXCL2, EZH2
palate development	0.023183993	4.604532722	WNT5A, LEF1, SNAI1, TWIST1, INHBA
cerebral cortex development	0.055724063	4.604532722	COL3A1, CDH2, MDK, KIF14
positive regulation of neuron differentiation	0.010187156	4.559828521	FOXA1, NGFR, IL6, BMP2, ECT2, CCR5
positive regulation of neuron projection development	0.001948011	4.537800364	NTRK1, NGFR, IL6, SERPINF1, WNT5A, LIF, PTN, DBN1
negative regulation of sequence-specific DNA binding transcription factor activity	0.05773108	4.537800364	SFRP4, TWIST1, HMOX1, EZH2
negative regulation of neuron apoptotic process	0.001034732	4.403084416	NTRK1, NGFR, MDK, KIF14, BIRC5, HMOX1, CCL2, APOE, PPARGC1A
single organismal cell-cell adhesion	0.012331375	4.348725349	SELP, CDH3, ITGAM, CCDC80, CDH2, SOX9
cell fate commitment	0.063954044	4.348725349	BMP2, WNT5A, SOX9, WNT4
hippocampus development	0.066094593	4.289153769	MDK, LEF1, KIF14, EZH2
positive regulation of protein phosphorylation	0.000595291	4.254187841	NTRK1, C3, BMP2, GPNMB, CHEK2, WNT5A, ADIPOQ, GATA4, SOX9, IQGAP3
positive regulation of peptidyl-serine phosphorylation	0.068267699	4.231192231	IL6, SFRP2, WNT5A, LIF
kidney development	0.006399124	4.214918415	WT1, ALDH1A2, SERPINF1, HAS2, DCN, WNT4, FBN1
heart development	3.12034E-05	4.198769302	EDN1, SPARC, MMP2, GATA4, PTN, VCAN, COL3A1, BMP2, MMP13, WT1, ALDH1A2, SOX9, ZFPM2, FBN1
regulation of cell cycle	0.01425278	4.193413729	FOXA1, CCNE1, CCNF, E2F1, SKP2, DTL
transforming growth factor beta receptor signaling pathway	0.070473014	4.174776335	COL3A1, BMP2, COL1A2, CCL2
cellular response to lipopolysaccharide	0.000355161	4.119845067	IL10, CXCL10, IL6, ARG1, WNT5A, NR1H4, HMGB2, CCL2, CD14, CXCL2, PPARGC1A
liver development	0.033085614	4.119845067	ARG1, ALDH1A2, PTN, FOXM1, WNT4
sensory perception of pain	0.074978857	4.066340586	NTRK1, NGFR, EDN1, BDKRB1
neural tube closure	0.035319926	4.034899808	STIL, SFRP2, WNT5A, TWIST1, KIF20B
immune response	4.77018E-05	4.028966132	IL10, NGFR, CCL24, CCL11, CMA1, LIF, CXCL2, CXCL10, IL6, CCL8, IL5, CCL2, CCR7, CCR5
cellular response to amino acid stimulus	0.008200145	3.99955762	COL1A1, COL3A1, COL1A2, COL16A1, MMP2, COL5A2, COL6A1
response to lipopolysaccharide	0.000968344	3.973454633	NGFR, CXCL10, EDN1, SPARC, HMGB2, BDKRB1, CCR7, CXCL2, LOXL1, DCN
regulation of cell shape	0.008775524	3.942010028	CCL24, IL6, CCL11, FN1, CCL2, CDC7, PTN
DNA repair	1.46203E-05	3.93846824	BARD1, BLM, UHRF1, BRCA1, FOXM1, BRCA2, MMS22L, BRIP1, CHAF1B, RAD51, CHAF1A, FANCD2, EXO1, CHEK2, CHEK1, POLE
positive regulation of gene expression	8.97318E-07	3.923661969	LEF1, TNC, HMGB2, LIF, FN1, TWIST1, GATA4, INHBA, BRCA1, IQGAP3, ETV4, SFRP4, IL6, BMP2, CDH3, ALDH1A2, E2F1, CDK1, SOX9, CCR5
cellular response to DNA damage stimulus	4.56401E-07	3.913852814	BARD1, TOP2A, BLM, EGLN3, MCM7, UHRF1, BRCA1, FOXM1, BRCA2, MMS22L, BRIP1, CHAF1B, RAD51, CHAF1A, FANCD2, EXO1, CHEK2, CHEK1, DTL, POLE
cell proliferation	0.000531678	3.913852814	MCM7, UHRF1, CDK1, CSPG4, IGF1, MKI67, BRCA2, BUB1, CFB, AURKB
positive regulation of cell proliferation	6.69994E-09	3.899410553	COL18A1, LEF1, TNC, KIF14, PTN, FOXM1, GLI1, CDC20, HAS2, SOX9, TIMP1, EDN1, WNT5A, LIF, FN1, CDC7, IGF1, CXCL10, ALDH3A1, IL6, SFRP2, FABP4, IL5, PRC1, ALDH1A2, KIF20B
response to cytokine	0.084361126	3.865533643	COL3A1, SPARC, ALDH1A2, TIMP1
positive regulation of transcription, DNA-templated	5.48072E-09	3.80513468	BLM, LEF1, HMGB2, GATA4, BRCA1, FOXM1, BRCA2, GLI1, MDK, CHEK2, E2F1, SOX9, PPARGC1A, WNT4, IL10, WNT5A, IGF1, INHBA, ETV4, ESR2, CCNA2, COL1A1, IL6, BMP2, IL5, WT1, SNAI1, ZFPM2
cell-cell signaling	0.042536255	3.799857101	EDN1, WNT5A, GATA4, CCR5, WNT4
nucleosome assembly	0.043813953	3.763320013	CHAF1B, CHAF1A, SOX9, CENPA, MCM2
Notch signaling pathway	0.021857336	3.757298701	CFD, FOXA1, BMP2, NR1H4, SOX9, DLK1
positive regulation of apoptotic process	2.6772E-05	3.738605673	BARD1, TOP2A, NGFR, IGFBP3, MMP2, PTN, ESR2, ALDH1A3, SFRP4, IL6, BMP2, SFRP2, WT1, ALDH1A2, HMOX1, ECT2
negative regulation of cell migration	0.046433572	3.692313975	SFRP2, ADIPOQ, PTN, CCR5, WNT4
cell migration	0.003147632	3.688447678	MMP14, CDH2, COL5A1, MDK, MMP2, WNT5A, SNAI1, BDKRB1, ADAMTS12
response to hypoxia	0.003241139	3.669237013	ALDH3A1, EGLN3, MMP14, BMP2, EDN1, MMP2, ADIPOQ, HMOX1, CCL2
forebrain development	0.096752244	3.640793315	STIL, ALDH1A2, E2F1, PPARGC1A
circadian rhythm	0.04913869	3.623937791	NTRK1, NGFR, ADIPOQ, IGF1, PPARGC1A
negative regulation of gene expression	0.00060587	3.544621416	BRIP1, BMP2, CCNB1, SFRP2, SERPINF1, HMGB2, GATA4, IGF1, SOX9, IQGAP3, WNT4, EZH2
glucose homeostasis	0.027641436	3.531295772	FOXA1, NGFR, IL6, ADIPOQ, NR1H4, FBN1
negative regulation of cell proliferation	0.000125271	3.261544012	NTRK1, IL10, IGFBP3, LIF, IGF1, INHBA, ESR2, SFRP4, IL6, BRIP1, BMP2, SFRP2, WT1, ALDH1A2, HMOX1, SOX9
axon guidance	0.041913756	3.152096226	NTRK1, NGFR, LAMA1, TUBB3, WNT5A, EPHB1
protein phosphorylation	4.67323E-06	3.125646344	NTRK1, PLK4, LAMA1, IGFBP3, PLK1, WNT5A, BUB1B, CDC7, AURKB, CIT, AURKA, BMP2, CCNB1, CCNE1, CHEK2, CHEK1, PBK, CDK1, FAM20C, BIRC5, NEK2, EPHB1, BUB1
rhythmic process	0.080804374	3.057697511	TOP2A, NGFR, TWIST1, PPARGC1A, EZH2
angiogenesis	0.011764565	2.947671575	COL18A1, MMP14, CCDC80, MMP2, FN1, HMOX1, CCL2, CSPG4, EPHB1
peptidyl-serine phosphorylation	0.089985677	2.942746477	CHEK2, PLK1, PBK, CDK1, CDC7
negative regulation of apoptotic process	3.90527E-05	2.904272406	BARD1, IL10, NGFR, STIL, PLK1, LEF1, WNT5A, KIF14, NR1H4, FN1, TWIST1, IGF1, AURKA, IL6, WT1, SPP1, CDK1, BIRC5, TIMP1, SOX9, MAD2L1
spermatogenesis	0.002213774	2.692576874	HMGB2, GATA4, GLI1, CDC25C, BRCA2, CIT, CLGN, BRIP1, ADAMTS2, CCNB1, RACGAP1, WT1, E2F1, SOX9
regulation of gene expression	0.017380574	2.541462866	IL10, FOXA1, NGFR, WT1, CHEK1, GATA4, IGF1, APOE, DLK1, EZH2
negative regulation of transcription, DNA-templated	0.001218295	2.433483615	LEF1, WNT5A, ADIPOQ, HMGB2, TWIST1, BRCA1, FOXM1, BMP2, FABP4, SFRP2, WT1, SNAI1, E2F1, BIRC5, SOX9, ZFPM2, EZH2, WNT4
regulation of transcription from RNA polymerase II promoter	0.013029114	2.366057117	FOXA1, BRIP1, WT1, LEF1, UCP1, SNAI1, NR1H4, HMGB2, GATA4, INHBA, SOX9, EZH2
positive regulation of transcription from RNA polymerase II promoter	2.67129E-05	2.36011225	FOXA1, TOP2A, LEF1, HMGB2, TWIST1, GATA4, BRCA1, FOXM1, GLI1, CREB3L1, E2F1, SOX9, PPARGC1A, IL10, EDN1, LUM, WNT5A, NR1H4, LIF, IGF1, INHBA, ETV4, ESR2, DCN, IL6, BMP2, SFRP2, WT1, ZFPM2
proteolysis	0.00326085	2.286443225	CFD, F10, CMA1, MMP2, ADAMTS12, MMP10, MMP12, ADAMTS4, MMP14, ADAMTS2, MMP13, ESPL1, CTSK, ADAM12, CLCA2, CPXM1, CFB
phosphorylation	0.005275732	2.174362674	NTRK1, PLK4, PLK1, BUB1B, CDC7, AURKB, CIT, AURKA, CHEK2, CHEK1, PBK, CDK1, FAM20C, NEK2, TK1, EPHB1, BUB1
innate immune response	0.032040057	2.152619048	CFD, C3, C4B, C1QA, NR1H4, HMGB2, PTX3, TREM2, CD14, CFP, CFB
negative regulation of transcription from RNA polymerase II promoter	0.012188747	1.932766822	FOXA1, EDN1, UHRF1, PLK1, LEF1, NR1H4, TWIST1, FOXM1, AURKB, ESR2, BMP2, CCNE1, WT1, SNAI1, E2F1, SOX9, ZFPM2, EZH2
cell differentiation	0.010878658	1.906748807	NTRK1, NGFR, WNT5A, TWIST1, GLI1, DLK1, CIT, CLGN, CDC20, SFRP4, BMP2, SFRP2, RACGAP1, MDK, CSPG4, SOX9, ECT2, DBN1, WNT4
apoptotic process	0.060073877	1.785266196	NGFR, EGLN3, BUB1B, TPX2, LGALS1, SFRP2, WT1, CHEK2, E2F1, CDK1, HMOX1, BIRC5, BUB1
multicellular organism development	0.039323608	1.597490944	NTRK1, FOXA1, NGFR, COL18A1, STIL, WNT5A, TWIST1, GLI1, CIT, CLGN, SFRP4, BMP2, SFRP2, RACGAP1, FANCD2, MDK, CREB3L1, SNAI1, CSPG4, DBN1, WNT4
