# Supplementary material for: Hydrostatic Pressure Controls Angiogenesis Through Endothelial YAP1 During Lung Regeneration
Source: Front Bioeng Biotechnol. 2022 Feb 18;10:823642. doi: 10.3389/fbioe.2022.823642 (PMC8896883; doi:10.3389/fbioe.2022.823642)
Supplement: Supplementary file 7 [file DataSheet3.PDF]

ID	FPKM Sham LECs rep 1	FPKM Sham LECs rep 2	FPKM PNX7D LECs rep 1	FPKM PNX7D LECs rep 2
1190002F15Rik	1.25767437	1.052829398	6.814873807	5.959153991
1600029I14Rik	3.738199684	3.111678969	1.974335816	1.451348276
1700001C02Rik	2.969176786	3.033156217	1.048172666	1.693448723
1700003M02Rik	3.94685444	4.160795998	1.901161132	1.967400568
1700007K13Rik	7.766528298	6.961877291	3.553804468	3.53922358
1700026D08Rik	3.116199393	2.946188454	1.36769291	1.37904832
1700071M16Rik	1.289858038	1.390501491	3.005973588	2.667641621
1810010H24Rik	20.5541921	20.5529833	8.963736111	9.325524724
2010107G12Rik	0.607736971	0.377832211	1.244984327	0.90513971
2410004I01Rik	3.303465655	2.818124349	1.05049566	1.036376418
2610318N02Rik	0.154617479	0.052219925	0.522122931	0.742870208
2610524H06Rik	1.532823697	1.562398058	4.670714444	4.210504889
2700094K13Rik	14.61606547	14.4423855	36.49061083	34.48510049
2700099C18Rik	1.351029344	1.209863992	6.605895364	6.865599736
2810408A11Rik	0.619744542	0.684536463	1.604943953	1.642220382
2810408I11Rik	0.137269095	0.154535824	0.986891002	0.811715337
2810417H13Rik	2.082924807	1.929490139	12.4256607	12.32507163
4833403I15Rik	0.224995091	0.205375661	0.450435608	0.546869778
4930427A07Rik	0.618451483	0.545172906	3.171412989	3.097895657
4930558J18Rik	0.019078531	0.021478371	0.311737234	0.293795198
4930579G24Rik	1.361968759	1.61163048	4.342536085	4.482470184
4932418E24Rik	1.542578353	1.484526021	0.724989663	0.59003262
4933413G19Rik	0.394359527	0.423784778	2.772748541	2.76038345
5730559C18Rik	0.168914336	0.175533821	0.424616531	0.448199054
6030419C18Rik	1.500808767	1.421729576	3.130139139	3.043932515
8430408G22Rik	71.4910369	67.90081141	34.95518837	34.89949651
8430426J06Rik	1.901529938	2.374578541	0.696255569	0.688991709
9330159F19Rik	0.749133193	0.804320081	0.328682837	0.384536478
9530026P05Rik	5.547768396	5.492591449	0.728619208	0.824020235
A2m	0.08800386	0.070766886	0.287591113	0.371710363
A530064D06Rik	1.081752712	0.904668978	2.323065868	2.348011219
A730017C20Rik	0.272289267	0.272479857	0.670114598	0.646040655
A830018L16Rik	0.172307225	0.22586865	0.087420247	0.104682208
AA467197	3.058527014	3.202223726	27.8196256	25.84795458
Abat	0.755954379	0.79874516	1.748149771	1.841770717
Abcb1b	1.761891805	1.69435866	3.971032893	3.958054351
Acaa1b	9.159821106	9.506592015	4.471533483	4.377925149
Acer2	204.0371235	202.7953239	86.70825821	85.80494496
Ackr1	0.777573707	1.065683309	2.467408284	2.873773709
Acoxl	7.27974329	7.75640199	3.845710812	3.624370927
Adam12	1.17574959	1.217408049	4.667407041	4.511885771
Adam28	1.059922725	1.089682804	0.544616541	0.527232111
Adam8	7.866955553	8.236779608	23.1985104	23.55481956
Adamts12	0.405056128	0.484286615	2.024881853	2.197169582
Adamts16	0	0	0.347235418	0.310281751
Adamts17	1.081163173	1.059989127	2.532284624	2.483867108
Adamts19	0.037756356	0.061397023	0.658055521	0.692535601
Adamts2	3.75845592	3.889133978	13.00309784	12.9852522
Adamts3	0.263105965	0.261692492	0.731815666	0.689171532
Adamts4	0.500955582	0.425976967	3.163882551	3.433703576
Adamts7	0.933691748	0.981062553	2.389145765	2.187735552
Adamts9	8.840338915	9.192956202	18.42255497	17.8023769
Adamtsl2	19.20626661	19.01201517	47.26932643	47.85750862
Adh1	51.48963738	50.96218442	108.484277	105.4431052
Adipoq	0.015888452	0.01788702	1.436521611	1.820347413
Adra1b	0.219507406	0.234762748	0.621691614	0.507037633
Adra2a	0.164795141	0.127547957	0.488052074	0.387002709
AF357359	4.567400341	5.141921968	37.67032736	36.97588838
Agr3	3.298279681	2.77141791	1.327795076	1.413316326
AI427809	0.085966504	0.029034003	0.449493055	0.508346979
Ak5	0.26494749	0.241460357	0.90706192	1.056912838
Akr1b8	6.139570791	5.374006965	15.46886055	15.21963664
Aldh1a2	8.516322568	9.256627571	21.06853515	21.92052686
Aldh1a3	0.391491458	0.434254754	1.586669171	1.758955304
Aldh1l2	0.175294683	0.160799266	0.4596961	0.467897355
Aldh3a1	1.847513249	1.824703883	0.765498125	0.851764504
Alox15	3.032680307	3.322865895	13.24947813	13.9653539
Alppl2	0	0.02086819	0.121152425	0.171269246
Amigo2	31.09762575	31.82104167	15.69025681	15.48623555
Angptl7	0.674003237	0.865655423	12.81230961	12.54853716
Ankle1	0.376081297	0.309795807	4.946016528	4.780001055
Ankrd55	0.098502184	0.086249739	0.530537206	0.626968972
Anln	2.043757287	1.914653527	16.0639042	15.38697311
Ano9	0.467918077	0.329235186	0.941545805	0.952739199

Aoah	0.564356222	0.524850342	1.891056091	1.81370428
Aoc3	0.979619207	0.967494092	2.211830988	2.260068345
Aox3	11.69984792	12.08991876	5.629030655	5.420278812
Apitd1	0.862965041	0.781951267	3.737226634	3.526452188
Aplp1	3.567940543	3.795637573	8.004974279	7.561050319
Apoe	214.8039461	218.3070879	570.6199807	581.7023166
Apol7a	0.163666997	0.184254251	0.632909993	0.735942231
Apol7c	1.522679594	1.239840005	0.438140277	0.424720917
Aqp4	0.600872367	0.520349668	0.201396239	0.185059757
Arg1	1.444569022	1.283118162	157.300962	157.4395036
Arhgap11a	2.567285023	2.609101895	7.641706718	7.695365839
Arhgap19	1.923182745	1.92500525	4.048848048	4.36316734
Arhgef38	1.332300773	1.22298518	0.558189183	0.441892588
Arhgef39	1.074067193	0.991792241	7.729853368	8.043192362
Arnt2	0.077357113	0.105230931	0.323019935	0.266044057
Arsi	0.363755129	0.307133219	1.721180561	1.93253772
Asf1b	1.970923224	2.315311956	9.574602657	9.561429658
Aspm	0.398792738	0.359611409	3.633050057	3.593008659
Aspn	3.247164367	2.821386363	9.550377129	10.35978601
Atad2	5.387054724	5.223223818	11.19607869	11.1429224
Atad5	1.033640813	0.969716543	2.070120425	2.012875256
Atp1a3	4.569939196	4.734422712	9.428743039	9.456880364
Atp2c2	1.305781313	1.237562	0.535882399	0.583601417
Atp7b	3.236805833	3.428779576	1.557005396	1.76598915
Aurka	1.793922882	1.919154962	11.87600481	11.89252018
Aurkb	1.600373869	1.53142869	13.8375013	12.70427482
B4galt6	1.29081636	1.27106247	3.149963051	3.346497264
Bai2	0.12107249	0.116257494	0.725371769	0.750157427
Bard1	0.499424675	0.570335901	1.565552629	1.659878154
Basp1	3.672210978	4.252925832	9.701598828	10.11387829
Batf3	2.061928324	2.166540155	4.761698683	4.030407961
BC030867	0.138553799	0.112653759	1.693751874	1.621555399
BC048546	0.404157848	0.469067868	0.19516423	0.174521101
Bcat1	0	0.002719922	0.08948113	0.059527795
Bdkrb1	0.512040397	0.428218998	1.067734975	1.189514834
Bean1	0.363667972	0.292437741	0.918057421	1.038262351
Birc5	0.928302344	0.883794718	8.788799809	9.64306019
Blm	0.937964774	0.877729733	2.224540767	2.350038441
Bmp2	1.454644368	1.606721871	3.121288725	3.049841314
Bnc1	0.650666695	0.614365236	1.609615063	1.629022859
Bok	5.221708689	5.001370629	12.73115707	13.33718588
Bora	1.724406334	1.806768419	4.761137081	5.142648539
Bpifa1	79.57489413	76.62980759	18.73473008	17.32152616
Brca1	0.550608473	0.50053521	2.687805184	2.680621323
Brca2	0.548573317	0.526523942	1.187474411	1.104688943
Brinp1	0.332662688	0.492772997	1.945374181	2.034723577
Brip1	0.440449065	0.384603176	1.405528898	1.59651692
Bub1	0.961352093	0.950169052	6.23411215	6.055088538
Bub1b	3.393139372	3.063170428	13.19237309	13.31599948
C1qa	16.7161787	16.15082171	114.7924826	117.5647821
C1qb	16.6807801	16.86998612	133.0166927	131.9042837
C1qc	13.83591727	14.88634498	110.4579074	113.3894704
C1qtnf6	4.184185885	4.383763786	15.43006866	15.86128154
C1s1	38.09336479	38.47028769	81.91251424	80.35005154
C3	94.88309917	97.81543802	349.5896772	354.964958
C330013F16Rik	0.276107561	0.381731401	0.105532427	0.113382699
C330027C09Rik	1.398887443	1.425128276	8.987351511	8.774501861
C3ar1	0.915210316	0.984198004	7.186968143	7.10147072
C4b	22.72508824	21.67722395	74.77420742	72.23681385
Cacna1g	0.023804661	0.02411909	0.134839717	0.175955562
Cacna1i	0.689527021	0.738010329	0.313506949	0.268378943
Cacna2d4	0.937744488	0.953168864	0.378468234	0.403089239
Cadm3	0.296513126	0.23313767	0.666498554	0.626205497
Cadm4	1.771249055	1.77930611	4.066673698	4.218675453
Calca	0.50456599	0.553469082	8.216264395	7.841663734
Calcb	0.488346933	0.343609279	3.989718114	3.860358952
Camk1g	0.047879102	0.044918077	0.269469071	0.245767528
Capn11	0.296469259	0.462131128	1.175860741	1.451565057
Capn6	0.49583776	0.44656634	2.196497217	2.002206416
Car13	0.936582435	0.824343421	2.309431873	2.517412073
Car3	0.268846325	0.172950753	7.823483218	7.743834218
Car6	0	0.016097011	1.183736185	1.655794538
Casc5	0.548988021	0.486329565	3.035847679	3.680958004
Cav3	0.473714815	0.590441713	1.363776304	1.375601424
Cbr2	872.4201168	871.2417328	434.4343225	436.3557922

Cbx7	22.83587401	22.81377161	11.45374105	11.0195805
Ccdc11	1.996457861	1.839885334	0.930597754	0.903729637
Ccdc147	0.971413782	1.03431358	0.433468527	0.569525284
Ccdc151	1.299039837	1.45242601	0.523378476	0.833053612
Ccdc153	17.35756325	18.07992294	8.246397108	9.192900427
Ccdc170	2.102150784	2.366574964	1.0606892	1.17714762
Ccdc18	0.172333581	0.231866784	1.03935617	1.092592607
Ccdc34	2.513906186	2.79832488	5.794803823	6.3447553
Ccdc40	1.875290474	1.973974003	0.980704426	0.900850778
Ccdc80	8.805338298	9.399472018	40.53558221	40.66713125
Ccl11	0.346614059	0.287525926	1.569900576	1.595661134
Ccl12	0.766914236	1.068949663	6.802618254	5.398815631
Ccl2	17.12167976	15.88509392	55.50284199	54.12346161
Ccl24	0.865593991	0.46159335	32.20755919	32.90750548
Ccl7	4.703777215	3.916429033	34.5595253	33.83302842
Ccl8	0.689515288	1.207496353	29.5849006	27.51247715
Ccl9	9.949974051	9.252822158	47.03356246	45.62394068
Ccna1	0.099997818	0.154792396	0.43571549	0.461967621
Ccna2	5.19481728	5.103508463	32.32938024	32.73629764
Ccnb1	1.242427554	1.008593132	11.85829056	12.20312604
Ccnb2	2.794552911	2.731363018	26.48403018	25.41355532
Ccne1	0.800596028	0.878768434	4.164273947	4.04377547
Ccne2	3.264455168	3.065990739	7.006071666	7.35423759
Ccnf	0.934829554	0.810647358	5.346187941	5.55595444
Ccr3	0.358837267	0.363577041	1.309467824	1.289359675
Ccr5	2.528772574	2.530542595	12.19175983	11.60407089
Ccr7	26.1357715	25.61848717	10.45088423	10.15870307
Ccr9	0.900287733	0.908165375	0.368974234	0.444738501
Ccsap	1.164368533	0.997550999	3.246685564	3.076362158
Cd109	0.303611067	0.285460617	1.58094932	1.615319747
Cd14	36.79218336	36.85626359	85.4581927	80.57594189
Cd163	0.784377364	0.87323061	4.670895276	4.665111939
Cd248	2.124016886	2.262170421	8.896995261	8.160598521
Cd276	0.934171139	0.934056244	3.514751672	3.861382106
Cd28	2.570948072	2.460445139	1.111335657	1.133955546
Cd4	2.264199384	2.549007244	1.109200361	1.293389929
Cd5l	0.672665735	0.573368113	29.91685764	29.01578486
Cd6	1.606482745	1.656029111	0.787131379	0.755074961
Cd8a	1.876157146	1.999505941	0.87880021	0.955343136
Cdc20	2.995436322	3.569864146	31.69738604	30.77869633
Cdc25c	0.282374055	0.238419875	3.416485903	3.714733764
Cdc45	1.909046073	1.552756917	7.223692143	7.674396684
Cdc6	0.466358682	0.426579359	2.549123307	2.882890218
Cdc7	1.932694639	1.903827942	5.204059243	5.342289925
Cdca2	0.562544948	0.57165678	4.522695208	4.293056173
Cdca3	2.091637829	2.339929928	18.55716446	18.91254459
Cdca5	0.801058072	0.77464122	5.168492123	5.174667531
Cdca7	3.125630656	2.76276802	6.466388816	6.26104071
Cdca8	1.793733924	1.788965998	16.06424529	16.87733073
Cdh2	1.040662829	1.015040251	2.221319022	2.32011952
Cdh3	0.344264225	0.474907687	2.038793943	1.995123378
Cdhr1	0.04093196	0.015360229	0.118900521	0.240922984
Cdhr3	5.660916101	5.805837102	2.909955707	2.711169543
Cdk1	1.779506626	1.864653021	20.18958566	18.56664022
Cdkn2c	7.949726672	7.668378967	27.85311997	27.20798595
Cdkn3	1.063560235	0.855244829	12.74404397	10.48194372
Cdt1	3.63617123	4.103613802	10.082359	10.53298827
Ceacam19	0.029120162	0.045896358	0.25376765	0.208070986
Cela1	0.796433797	0.677442574	2.332786658	1.744282808
Cemip	0.099056034	0.086734697	0.797283094	0.789812677
Cenpa	7.01287989	7.037933584	25.03877194	25.73798871
Cenpe	0.776669345	0.713594334	5.179921321	5.209987223
Cenpf	0.351997355	0.366553638	6.031434377	5.999406767
Cenph	0.568752723	0.409077154	3.717296683	3.912071568
Cenpi	0.665336499	0.539019774	3.833773816	3.914427165
Cenpk	0.875140576	1.055595295	2.529167413	3.014338727
Cenpl	1.239526917	1.2324723	3.001063445	2.920293031
Cenpm	0.427456787	0.497819468	3.981973681	3.540940071
Cenpn	1.073944327	0.810176781	4.727685582	4.964793197
Cenpp	0.910013351	0.961758243	3.904464492	3.889469325
Cenpq	3.230754749	3.255090289	10.39524774	10.11747534
Cenpw	0.453115113	0.247768349	1.903823822	1.419450512
Cep55	0.866692752	1.166094615	11.21405686	11.54519944
Cercam	1.586153236	1.420420433	4.241004227	3.854800759
Ces2b	2.840637227	2.942117432	1.519040432	1.081662446

Cfap221	0.647028893	0.649432029	0.288720076	0.307316095
Cfap44	2.35204119	2.323187764	1.00735214	1.087964274
Cfb	12.65542099	12.69101964	54.31227491	53.9442635
Cfd	0.190044397	0.166405242	9.499813886	10.48352541
Cfi	0.20773787	0.414585397	0.781734123	0.791027625
Cfp	17.0057313	16.15165028	44.87027925	46.69455949
Chaf1a	2.023731538	1.991839698	6.67788651	6.874296057
Chaf1b	1.708810074	1.797042402	7.67978446	7.42475906
Chd3os	1.034294464	0.904264764	2.241353914	1.748621454
Chek1	0.535894989	0.415176844	2.347582345	2.61236774
Chek2	3.040287467	2.696983676	6.063767838	6.535762474
Chga	0	0.0234185	0.861070583	0.922560631
Chil3	934.0085676	929.9991364	2683.475676	2609.528117
Chil4	0.233339279	0.394035647	2.005192985	1.549090631
Chl1	0.030305106	0.025587831	1.020612662	1.011131556
Chrd	0.66791419	0.692566563	1.474281051	1.732274875
Chrm1	0.535607149	0.501314605	0.23744796	0.195649093
Chtf18	1.020953254	1.284179757	3.411926891	3.315190306
Cilp	0.259234652	0.291843117	1.052535395	0.998731059
Cit	1.362499273	1.434821199	3.225015714	3.126239933
Ckap2	0.714949798	0.804881508	15.46130387	15.62217841
Ckap2l	1.622374546	1.685952829	15.11018298	14.94389413
Cks1b	4.990611053	5.70569941	24.42125607	24.65625548
Cks2	7.587262402	7.080991759	26.26947683	25.08763759
Clca2	0.02678513	0.012061745	0.9220051	0.752347268
Clca3	1.900767725	1.539197863	5.114572427	4.790076941
Clec10a	2.464753015	2.466478226	5.696504697	5.430227793
Clec11a	2.471840237	2.546175208	8.666486356	7.853307849
Clec4a2	3.187602466	3.445357282	7.254325635	6.544894484
Clec4d	10.95941656	10.9029419	23.31785177	25.08940617
Clec4e	8.066060603	8.494537908	16.62486378	16.81041304
Clgn	0.034371506	0.03869501	0.336971802	0.201132347
Clhc1	0.215782693	0.260277265	1.662173113	1.689928483
Clip3	1.233791261	1.328886327	2.843043575	2.776845373
Clspn	0.550068287	0.575027117	3.890495698	4.240161742
Cma1	0.563951449	0.614409157	6.361177886	5.580448647
Cntrob	2.755306969	2.934521832	6.072957003	5.848580254
Col11a1	0.002690663	0	0.058619455	0.043091578
Col14a1	3.81594884	3.759381091	10.88628193	10.78579763
Col15a1	3.400433418	3.413861656	14.16711223	14.75716873
Col16a1	7.997525266	7.843681527	19.20528907	19.60767704
Col18a1	14.03447188	13.58131053	34.36469394	33.35916773
Col1a1	41.45326217	42.26770558	211.0360745	212.7155576
Col1a2	57.75317305	60.21451855	248.8754905	251.2327954
Col24a1	0.054853784	0.021613796	1.819476184	1.882008318
Col28a1	0.180474785	0.182337658	0.38814602	0.467471916
Col3a1	70.69221201	70.9542457	343.3649598	344.0333484
Col5a1	7.879219889	7.954967862	27.23088927	28.2046243
Col5a2	16.69535	16.86657008	56.36446706	56.64313849
Col5a3	1.177176089	1.028336115	8.370067901	8.213899679
Col6a1	61.03761435	61.76513913	144.712062	149.6252241
Col6a2	47.8157105	48.6712669	143.4444713	145.9622192
Col6a3	12.8360519	13.30217456	31.43843407	31.72437888
Col6a4	0.105253786	0.082945374	0.240773955	0.21719123
Col6a5	0.155804926	0.234661077	2.360028911	2.404467678
Col6a6	0.070741998	0.076690807	0.519442441	0.629416198
Colq	12.69758009	12.91116007	4.82114003	4.516723426
Coro6	8.500156326	7.819049867	3.498276273	3.469629472
Cpxm1	4.252881181	4.296312921	18.36352495	18.24789276
Cpz	0.459412433	0.385365265	2.816222409	2.752162004
Crb2	0.301052753	0.283587303	1.003899567	1.040732141
Creb3l1	4.585501002	4.356903121	9.933751988	9.588543797
Crmp1	0.279148449	0.339915846	1.222525286	1.221175643
Cspg4	1.818854723	1.97646903	3.795728052	3.99016068
Cst9	0.456549805	0.385483529	1.864970275	1.37095289
Ctsk	15.71653957	15.49397839	63.69908274	63.21923294
Cxcl1	6.951139161	6.641229939	54.80123771	55.49686643
Cxcl10	3.790349114	3.826391283	15.44935724	13.52615996
Cxcl13	1.987779347	1.896455115	16.03799538	15.29478583
Cxcl3	1.403677624	1.385418212	4.084431461	3.576936328
Cxcl5	0.035461183	0.066536257	1.480751862	1.441640261
Cxcr5	5.876114615	5.443892256	2.70714283	2.969383006
Cygb	5.491965109	4.963245143	12.44060251	12.22805105
Cyp1a1	9.89615113	9.607004753	2.708389196	2.820737496
Cyp1b1	0.648923048	0.575844838	4.1456432	4.067711387

Cyp26a1	0.054953882	0.074239669	0.526785165	0.379119525
Cyp26b1	268.0533695	273.34459	1.688588471	1.631186805
Cyp2a5	9.142409845	8.416390393	2.946653034	3.149676384
Cyp2b10	74.34793845	73.74938286	35.06117635	34.64402933
Cyp2f2	226.3014221	221.0945725	95.29663366	93.54387434
Cyp4a32	2.17836156	2.129994546	0.712988984	0.768546133
Cyp4b1	465.7199958	451.3246726	217.6728979	217.0581657
D030025P21Rik	1.297050606	1.19268513	3.009139443	2.951825885
D130043K22Rik	0.334305545	0.385212444	0.111391098	0.10174983
D430020J02Rik	0.744537696	0.617213514	1.835886541	1.500915549
D430036J16Rik	1.255727317	1.104810342	0.436801843	0.571993117
D430041D05Rik	0.081222294	0.089261919	0.185379249	0.195357262
Dapl1	2.391309079	1.961391327	0.930315306	0.715445143
Dbf4	3.091565112	3.480445195	8.837980179	9.205820073
Dbn1	4.541917634	4.773315906	11.55365509	12.20376847
Dcdc2a	0.367631904	0.39194821	0.145865109	0.125972365
Dck	6.191891391	6.415688422	13.19685585	13.00001367
Dclk1	0.360009459	0.423947834	0.913952638	0.966815222
Dcn	29.77514206	29.73488739	147.8785105	146.6091043
Dcstamp	0.343079174	0.321861856	1.204210277	0.962709934
Dctd	0.295306057	0.316620811	1.171836387	1.178017434
Ddias	0.516464521	0.479199951	1.768143945	1.803883773
Depdc1a	0.27096911	0.27189562	2.759198173	2.699570283
Depdc1b	0.564799929	0.609714067	2.814950259	3.28836952
Dfna5	0.321195523	0.340935152	0.819724237	0.75747096
Dhfr	1.258101093	1.074763058	2.942469484	2.931145257
Dhrs3	85.0192217	85.10598249	42.44393517	42.64693812
Diap3	0.957572963	0.821052874	5.294319626	5.274235452
Dixdc1	2.45495343	2.551752175	1.204700553	1.164187495
Dkk2	0.29586399	0.29144497	0.656086979	0.63134328
Dlgap5	0.917970861	0.8996133	6.7623	6.728362017
Dlk1	0.004068712	0.013741514	0.221604935	0.215533677
Dmkn	2.831257539	2.729142634	7.85318529	7.544204504
Dnah5	0.989007301	1.021686047	0.447866752	0.44319427
Dnah6	2.293061183	2.168668603	1.110721909	0.97594905
Dnah7a	0.137175136	0.149042951	0.071238258	0.047160449
Dnah9	0.709141271	0.793542688	0.371531758	0.357151213
Dnph1	0.513257336	0.458269976	2.602695858	2.921673703
Doc2b	0.184448272	0.134972207	0.386774479	0.420374211
Dock3	0.034557339	0.038904219	0.138811297	0.133039335
Dok2	5.301209315	5.236469331	12.25721029	12.13460063
Dpf1	0.128893149	0.096737526	0.468016392	0.518709737
Dscc1	0.254215232	0.606054319	2.296805638	2.155401871
Dsn1	2.203532734	2.065649148	5.800033048	5.555995341
Dtl	0.600932866	0.57163544	4.252386689	4.298409722
Dut	3.919590033	3.737133914	8.760014836	8.563025938
E030019B06Rik	1.326890664	1.678869029	0.690721444	0.650966972
E2f1	2.785539414	2.581271849	7.245219952	7.073336955
E2f7	0.59696013	0.54327413	3.239699033	3.293974241
E2f8	0.715948886	0.752272518	4.488712119	4.253231127
Ear1	65.06555083	64.16327392	31.93938153	28.95118037
Ear6	1.127187484	1.207072372	2.695301142	2.404697187
Ear7	1.547562386	1.789313505	3.827174043	3.194683779
Ebf2	0.038922687	0.015934062	0.165741423	0.130773914
Ebf3	0.109115676	0.118453878	0.44572901	0.422475505
Ecel1	0.006557646	0	0.19286979	0.274673412
Ect2	1.374071305	1.188285517	8.167621539	8.381867804
Edn1	133.5313227	134.4113695	56.40906529	57.33416289
Efcab11	0.378435435	0.34083029	0.886304063	0.920764657
Efcab6	0.501715721	0.431164321	0.212769354	0.217037107
Efna4	0.576444189	0.635146108	1.162334544	1.34474259
Efna5	0.677423895	0.678829239	1.844815782	1.788312764
Efs	1.145641482	0.999709596	2.263049947	2.214960745
Egln3	0.731486281	0.889377919	3.577708983	3.658659262
Egr4	0.046606126	0.010493718	0.436610263	0.539710172
Elmod1	1.142163178	1.260454686	0.376525924	0.610967696
Eln	70.26344968	73.48322685	245.9879351	254.961555
Emid1	9.638761427	9.61731985	19.87229437	19.9883309
Emilin2	8.891161496	9.338872525	30.32095065	30.18964311
Emr1	14.38082643	14.09812459	33.22286975	33.10663551
Eno2	0.595197959	0.485798133	1.531742405	1.402337743
Eno4	2.280014528	2.42945726	0.963555867	0.986382754
Enpp3	1.189077074	1.064655754	3.643438613	3.70072989
Entpd2	0.909505194	0.796374079	1.618201105	1.842528164
Ephb1	0.461367692	0.434408908	1.151350408	1.064416161

Ephb2	0.141056218	0.145187949	0.544375237	0.417054895
Ercc6l	0.470946132	0.440946143	2.219646335	2.274755857
Errfi1	79.82897386	81.06449544	40.16576223	38.59312406
Esco2	0.567183209	0.699339902	5.207506513	5.090790758
Esm1	14.06427943	13.45471328	3.22427909	3.050172521
Espl1	0.909346674	0.890778983	5.165076901	5.215760766
Esr2	1.34495132	1.383035865	0.602518902	0.710100021
Etv4	0.100554313	0.056601392	1.104478615	1.280061651
Exo1	0.199087556	0.160093051	1.037870974	1.011714124
Ezh2	5.365209142	5.527793342	12.64019092	13.16498594
F10	5.349753303	5.654450289	15.44776647	15.08848349
F13a1	7.427072474	7.558844146	21.10020499	20.89695888
F2rl3	0.333278767	0.43773456	1.367468462	1.135936955
F5	1.239599606	1.303284107	3.918494098	3.884532651
Fabp12	0.444168254	0.49126643	0.135814183	0.163196683
Fabp4	35.69840204	35.65081177	81.09992474	82.125447
Fabp5	45.08519149	43.84238068	93.01048378	93.67539952
Fabp7	0.179818911	0.144598481	2.966167509	2.436788262
Faim2	4.776229332	4.900215757	2.00986371	2.11804435
Fam110c	0.608497959	0.478702206	1.461452175	1.219283081
Fam132b	0.173841678	0.289648992	0.719598063	0.770985394
Fam167b	0.829431055	0.957107146	1.919957922	2.605614832
Fam179a	3.40864672	3.321562904	1.680017185	1.55578773
Fam180a	1.601323044	1.42777751	4.005007391	3.913905782
Fam183b	10.13558845	9.724427921	4.666739675	5.191957096
Fam20c	2.302507241	2.452613801	9.023655464	8.975718382
Fam216b	2.475247599	2.451816488	1.210008492	1.152937422
Fam47e	2.068011355	1.625306137	0.835911483	0.705014772
Fam64a	0.867903982	1.17249049	26.79171008	26.4505139
Fam71f2	6.707953124	7.120737419	3.064249771	3.321924311
Fam83d	0.909438121	0.692306814	6.679894416	6.508837605
Fancd2	0.434080487	0.465631308	1.949388141	1.821215532
Fbln2	9.680166266	9.529430674	26.91429401	26.67861463
Fbn1	9.006280758	9.150840351	24.12925543	23.67626055
Fbn2	0.059769688	0.033643982	0.201427467	0.200188908
Fbp1	0.383815155	0.297996057	3.67634945	3.766394839
Fbxo48	0.36060857	0.582929274	1.11801312	0.899887612
Fbxo5	3.444778184	3.304911503	9.215898029	9.421685186
Fcgr2b	26.563683	26.19899994	60.65833914	60.91069976
Fcna	2.080820201	1.874049009	24.13996566	24.04146848
Fcrlb	0.198184928	0.205951481	1.12924628	1.314664865
Fcrls	1.818832739	1.925868743	7.368209364	8.005938317
Fen1	5.100665033	4.94886022	12.27358033	11.81859541
Fermt1	0.312895171	0.247416118	0.11361354	0.110133784
Ffar2	0.886604858	0.764192225	1.901398569	1.749297775
Fgf23	0.028977902	0.00815574	0.252527927	0.249893366
Fgfbp1	3.215374553	3.549197512	1.298505479	1.082070312
Fhad1	3.063279442	3.150211905	1.434143512	1.541628508
Fhl2	0.595291125	0.420499624	1.246056665	1.107234738
Fhl5	0.070858182	0.019942813	0.289450138	0.48011187
Fignl1	0.842483298	0.926227658	5.004483226	5.043493445
Fjx1	0.774272352	0.91706537	1.915276574	1.858034914
Fkbp10	13.50592889	14.30741692	29.50627052	30.3124589
Flnc	0.768802775	0.776553472	2.424000167	2.317811406
Flrt2	2.559560891	2.400232904	5.423094602	5.416210715
Fmn1	0.295410752	0.285270912	0.624996667	0.574198268
Fmo1	368.5170021	371.1487405	174.5244956	172.5767238
Fmo3	3.992823325	4.51699755	1.877681764	1.619644689
Fn1	69.21383102	69.93188253	322.9326302	326.033739
Fndc1	8.059527359	8.417412459	22.56295016	22.29558785
Fndc5	0.06520028	0.130491834	0.347225899	0.294517181
Folr1	0.292157804	0.250596258	1.21238532	1.456823265
Folr2	1.901322991	1.651231714	11.8745067	11.39927541
Foxa1	4.317830166	4.003549446	2.060693648	2.058111412
Foxm1	1.870010743	1.696304154	11.83624498	11.45241545
Fras1	0.303845591	0.303131262	1.085785035	0.98601287
Frzb	0.100554313	0.120749635	0.372419235	0.313821566
Fsbp	0.128442079	0.096398987	0.652929955	0.569638815
Fscn1	12.33043961	12.23716284	33.61923012	33.59964817
Fsip1	0.842420351	0.974730319	0.369612658	0.46124968
Fstl1	41.95868547	42.46772033	112.699618	110.7646286
G530011O06Rik	0.328984418	0.314811549	1.137814708	1.19560326
Gabrb3	0	0	0.037421598	0.033857086
Gas1	5.607918199	6.13736728	13.17098277	13.49376289
Gas2l3	0.60626641	0.578025355	2.578456828	2.380023227

Gata4	0.311531664	0.253296649	0.942653705	0.810220135
Gatm	1.843680603	1.682912821	7.861491938	8.021184767
Gbp10	1.12631471	0.880722786	0.42307196	0.526313111
Gdf3	0.046407238	0.083591497	0.3134223	0.30872317
Gdf6	0.099757166	0.087348618	0.869333822	0.689576923
Gen1	0.363706654	0.328907589	1.539296361	1.300122948
Gfpt2	3.303946663	3.328399398	14.77459098	14.76278953
Ggct	3.535495544	3.62426218	7.123264463	7.701834633
Gins1	1.199132593	1.292927501	4.378630527	4.098876474
Gins2	2.451536104	2.590068043	8.915324504	10.57051346
Gjb3	0.061106887	0.149052294	1.031749684	0.740253839
Gjb4	0.081278503	0.091502319	0.737814076	0.734288742
Gjb5	0.287185635	0.168121191	2.427598827	2.731302227
Glb1l3	3.613156456	3.2272973	1.479190014	1.35981528
Gli1	0.919395559	0.848495355	2.79490903	2.680143882
Gm11346	1.913568112	1.403240716	0.822315294	0.7137089
Gm12695	0.717017322	0.64576728	0.208281581	0.250274766
Gm14005	1.10733664	1.14355821	2.331738967	2.449066298
Gm14085	1.172380836	1.337528212	0.376285179	0.432001486
Gm14461	0	0	0.227603208	0.121928308
Gm16548	3.627841873	3.995392343	8.659713708	9.599247904
Gm1976	1.095438964	0.956618776	1.951618563	2.459397706
Gm20554	0.403084949	0.341421429	0.15054359	0.222277184
Gm4759	0.721935073	0.634511792	0.26903599	0.171634945
Gm5577	0.261361128	0.216806267	0.794174154	0.864265777
Gm609	0.24906384	0.176543706	0.030145372	0.034092427
Gm867	5.068985487	5.56303796	2.760854611	2.45467323
Gm9733	3.357451013	3.530168341	7.142111892	7.296845851
Gmnn	6.210969903	5.593785559	18.85662473	18.95608714
Gpbar1	0	0	0.172305671	0.170508049
Gpnmb	10.72014524	11.08222857	22.18741747	22.74960644
Gpr133	1.505153096	1.489608008	9.606216378	9.126504548
Gpr173	0.103536944	0.061708549	0.305180471	0.217587612
Gpr176	0.210210192	0.152133426	0.866870228	0.86938739
Gpr39	0.531403429	0.696589333	2.434402317	2.376496133
Gpr64	0.605100072	0.671882366	1.629798958	1.69002095
Gpr84	0.088344301	0.142081292	0.618650681	0.777392322
Gpr88	0.15319231	0.146912056	0.889996249	0.929639538
Greb1l	0.072059347	0.070655959	0.232953339	0.177545661
Gria2	0.103569169	0.133659827	0.423759882	0.320533632
Grin2c	0.151957869	0.157566557	0.431248183	0.389185307
Gsg2	0.905263065	1.003454813	2.783876713	2.607923082
Gtse1	0.746368607	0.959156051	5.092934173	4.501990789
Gxylt2	1.050552105	1.291313528	2.674111358	2.680879569
H19	1.534526746	1.398894914	4.541802881	4.518626314
H1fx	0.976885464	1.037514521	6.404879239	6.176272194
H2afx	8.843846765	9.956291669	26.09261294	26.15532176
H2-Eb2	1.575231901	1.473467073	0.637161393	0.627838599
H2-M11	0	0	0.401918362	0.406696495
H2-M2	0.896740905	1.153759604	2.093209438	2.114771672
H2-M9	0.076091634	0.042831503	0.911763245	0.913968594
H2-Q1	0.473890352	0.446397799	0.073744954	0.023828765
Hapln1	0.573103222	0.488253718	1.784284024	1.803236461
Has1	2.53571188	2.601860269	12.38395534	12.40316815
Has2	0.05534752	0.046732157	0.864167345	0.687530605
Has2os	0.080719888	0.090873437	1.099114784	0.84525787
Hcn1	0.03958958	0.055711815	0.156329662	0.173750279
Hells	1.536659084	1.518269677	6.547287331	6.078600255
Hes2	0.520228633	0.454547417	0.143787408	0.105220868
Hhip	1.615042421	1.471062559	3.729967941	3.647223681
Hist1h1b	0.073959343	0.194279168	0.751938477	0.941506303
Hmgb2	17.26954332	16.4293739	50.9783482	51.11386858
Hmgb3	3.625324122	3.493042494	8.680928378	8.798247853
Hmgcs2	4.296422647	4.49566948	1.793085315	1.676462826
Hmmr	0.865202123	0.855798386	9.555502867	9.623703815
Hmox1	26.87777648	26.66509751	79.51412002	77.91377055
Hpgds	0.409534727	0.434321573	1.060322497	1.169907086
Hspb6	3.191024712	3.342942261	6.453172491	6.715719187
Hspb7	0.848608651	0.764282373	1.517556677	1.777240174
Ifi204	4.563135728	5.050702064	12.27340053	11.6738312
Ifi205	2.584863004	2.882682318	5.089453392	6.039890121
Igdcc4	0.219939021	0.208695297	0.941216272	0.925106845
Igf1	1.765392591	1.750653452	7.528591786	7.730774312
Igfbp3	53.02448798	53.23306018	16.43307128	16.04684826
Igj	0.717051433	0.471768021	6.948685235	7.319307737

Il10	0.17985825	0.35434378	2.220447508	2.252675802
Il22ra2	0.515537779	0.589314974	0.129595787	0.068396665
Il31ra	0.149424221	0.114149226	0.488309011	0.447055689
Il5	0.599742455	0.459698742	0.959110202	1.289052285
Il6	7.527296658	7.237479863	16.94841064	15.3074055
Ildr2	0.879196918	0.941766753	4.576921541	4.595224365
Incenp	5.537103304	5.404737909	15.53643334	15.2768973
Inhba	0.563297062	0.360314069	1.64558114	2.144931678
Inmt	1178.550018	1169.523366	580.0309134	564.2323501
Inpp5j	0.197267507	0.13324877	0.497307507	0.479100195
Insc	0.084336807	0.066461723	0.284794326	0.322083587
Insl6	0.207609106	0.367280141	1.259985221	0.876898539
Iqca	1.59767542	1.585826401	0.750616645	0.646063877
Iqgap3	0.731115125	0.683311889	9.658384025	10.2899625
Islr	3.274896696	3.303181901	7.026120426	7.075696171
Itgad	1.061688489	0.9784254	0.328126245	0.365005699
Itgam	9.770563187	10.29357113	43.2877977	43.62485949
Itgb8	0.177031021	0.128120979	0.523428565	0.615330128
Itm2a	9.391477066	10.1407127	20.55196481	20.28768173
Itpka	1.515730202	1.779521035	0.707615934	0.733578009
Iyd	0.699091889	0.758409641	0.29076582	0.281860245
Kcnd1	0.190141557	0.16899393	0.501457072	0.480814766
Kcng1	0.160585874	0.123695384	0.395890135	0.416488943
Kcnk13	1.24542591	1.267541359	2.85035217	2.79736833
Kcnmb2	1.166829627	1.109767331	0.496729093	0.578290403
Kcnt1	0.028871051	0.010834222	0.31799037	0.312202897
Kcp	0.264304789	0.256975876	0.946614856	1.021224413
Kdelr3	5.938820204	5.922197457	14.89883514	15.12711995
Kif11	1.710084081	1.623379746	12.61051181	12.32006963
Kif14	0.218329969	0.198220273	1.616218103	1.58200363
Kif15	0.864479375	0.817870352	4.686335562	4.759942721
Kif18a	1.540160872	1.498345942	3.545363127	3.236297161
Kif18b	0.428340474	0.594518213	7.13956761	6.823826759
Kif20a	4.255777819	4.249351281	16.50676196	16.63637087
Kif20b	1.284328439	1.259698918	5.768623827	5.95173406
Kif22	1.526892556	1.612188989	16.2193983	16.32182636
Kif23	3.554856741	3.455114198	16.56643566	16.55290008
Kif27	1.482789251	1.590285145	0.68336267	0.589086367
Kif2c	0.609918532	0.586503827	5.155957797	5.580586855
Kif4	1.159020763	1.318841252	5.579593687	5.496434283
Kifc1	1.39938807	1.205286249	11.74500236	11.67309812
Kifc5b	0.782386811	0.754972501	4.220734656	4.02064823
Klf15	9.676358176	10.43242108	3.745902007	4.005102197
Kndc1	1.072285559	1.07135949	0.487662569	0.392995134
Knstrn	1.362309916	1.533671402	10.47677058	11.26848371
Kntc1	0.374000603	0.449536963	3.488967064	3.270260892
Kpna2	11.47773042	11.77531128	32.71662386	32.44481566
Krt14	0.212254405	0.371705202	3.391100167	3.384775534
Krt15	0.7283562	0.807162169	0.247939847	0.238343062
Krt5	0.241330351	0.221374331	0.038945802	0.044045132
Lacc1	2.322406953	2.951602287	5.588537494	5.512788147
Lama1	0.777389005	0.812662299	1.796691227	1.920461236
Lctl	0.225409637	0.192860138	1.188421024	1.033011521
Ldlrad1	4.051337155	3.932651135	1.441035814	1.757037991
Lef1	2.866902457	2.725463696	1.266530803	1.484686501
Lgals1	91.56006468	93.10551563	279.5955665	276.9875856
Lgals7	0.819705732	1.181202462	9.536339628	9.8559283
Lgi2	1.07675388	0.926300718	3.69580911	4.16720206
Lgmn	64.29108651	66.22801766	140.134437	141.3425681
Lgr5	0.116342187	0.051455082	0.239887459	0.29177212
Lhfpl2	4.330896761	4.455003361	11.09787421	10.82056091
Lhx2	0.027765352	0.015628942	0.166348294	0.136821049
Lif	2.589491781	2.534309152	7.341853711	7.119369273
Lig1	4.878346241	5.254015992	12.37923705	12.36915351
Lin7a	0.008591619	0.01289645	0.096709236	0.081146792
Lingo1	0.60000967	0.60191586	1.300724792	1.36857382
Lipn	0.026064678	0	0.681421895	0.674312791
Lmnb1	10.14187333	10.45643847	34.07302223	36.12541298
Lmnb2	6.36420406	6.765973518	17.16705783	17.61926143
Loxl1	22.46560477	23.08649464	52.55329424	52.91076534
Loxl2	7.535076899	7.861780306	19.36343191	19.63160836
Lrat	35.39085706	34.00070667	4.125841418	4.070963351
Lrg1	29.91885937	31.33689536	64.53582837	64.76929451
Lrr1	0.091287149	0.234902688	1.448986232	1.349524067
Lrrc15	0.014750997	0.033212974	0.21692401	0.204438949

Lrrc25	3.874935089	4.12000032	8.626281074	9.850470624
Lrrc26	1.362273507	1.697947957	0.688972863	0.519455224
Lrrc43	0.908887793	1.077067861	0.385603417	0.506809639
Lrrc6	1.19288424	1.353508387	0.583205916	0.555425178
Lrriq1	0.491860607	0.466299366	0.213510263	0.264245828
Lrrn1	0.031269286	0.005867095	0.24411117	0.186189187
Lrrn2	0.672766253	0.625671489	2.823075994	2.825154288
Lrrn4	5.576640909	6.056158865	11.62784368	12.22737229
Ltbp2	12.16333069	12.59532259	25.03647982	25.80927673
Lum	0.754682328	0.456666392	12.00243938	11.57506071
Lypd1	0.421370454	0.603748176	2.253292853	2.135402386
Lyve1	372.1646643	369.1735986	167.5154645	168.8122187
Mad2l1	5.072367583	5.07165968	15.64910072	13.79780788
Mafb	7.491360589	7.20471636	17.17654682	16.7927752
Map3k19	0.741008984	0.884607656	0.395469095	0.379855693
Map3k7cl	0.580601738	0.666973621	1.316542575	1.284560813
Mapk4	0.097575894	0.109849717	0.277279932	0.235188977
March4	0.509584057	0.610525383	0.290279706	0.270692945
Marco	14.87075865	14.58759007	115.7985254	118.6419426
Mastl	0.496517541	0.450020809	3.740203499	3.514568628
Matk	1.059903818	1.0606457	2.217500682	2.456031898
Mchr1	0.706437304	0.583889853	0.224040665	0.187277326
Mcm10	0.504210521	0.53609866	2.849959966	2.622664491
Mcm2	6.689382369	6.537172481	20.07040328	19.22881637
Mcm3	13.03615007	12.89680161	28.54126125	28.24245294
Mcm5	5.010907613	4.784730762	17.55924195	17.10298092
Mcm6	16.65289414	17.06884128	36.91252542	36.77472466
Mcm7	13.23489486	12.67392405	27.77485078	26.5402712
Mcm8	0.761108105	0.971092077	1.830127416	1.868334372
Mcoln2	0.998552856	0.843118702	2.255452798	1.88141772
Mcpt4	0.395445917	0.467447452	2.003053428	1.997380005
Mdga1	0.191451017	0.19223227	0.428372941	0.455776302
Mdh1b	0.983876837	0.965961796	0.398790449	0.408723895
Mdk	3.320561901	3.722539985	10.27386381	10.55340829
Mefv	1.103717155	1.034339683	2.463075697	2.373986977
Meg3	0.512178158	0.496661629	2.794547694	2.972459986
Melk	0.503806783	0.77892631	6.146593864	6.22315092
Mest	9.952933712	9.292438846	28.89369726	27.91818223
Mettl21e	0.19076132	0.193973793	0.811085207	0.818732682
Mex3a	0.437174498	0.412045587	1.011505288	1.031221143
Mfap4	228.2578913	227.3738377	706.415528	724.2063349
Mfap5	12.0501626	13.237714	36.44539901	36.65093839
Mgp	1167.415763	1194.917426	2516.876706	2523.469517
Mir147	11.89341138	13.38945323	114.71141	108.3635694
Mir1906-1	2.32448053	1.790490685	11.86082241	11.6052041
Mir1906-2	2.32448053	1.790490685	11.86082241	11.6052041
Mir5134	2.007648501	0.847569555	12.02826128	12.36651785
Mir675	4.194551333	5.771545066	14.72290423	16.93771285
Mir6950	18.50199355	21.13118617	44.98240176	50.54178907
Mir6978	13.39312882	14.69120562	49.00509319	57.53685149
Mir7678	84.91504859	82.25005965	174.7871155	183.4076521
Mirg	0.075344776	0.016964441	0.541688441	0.594049818
Mis18bp1	0.687254676	0.504827285	4.268812937	4.455456844
Mki67	2.556837511	2.666289775	19.6466304	19.61010596
Mlxipl	0.407691844	0.52540486	1.706293622	1.526582068
Mmp10	0.022577362	0.012708655	0.418094643	0.458930464
Mmp12	1.883109729	1.936697608	30.63937104	29.6570149
Mmp13	0.607360579	0.757901449	2.343518161	2.127503371
Mmp14	26.68232115	26.70382601	82.12203071	82.7347187
Mmp19	15.78777454	15.80804358	44.05976521	45.79909267
Mmp2	25.56087765	27.609418	86.85493446	87.72013153
Mmp23	3.030373209	2.535616033	8.624295362	8.475462864
Mmp8	4.125582627	4.186364749	9.031560153	9.929008118
Mms22l	0.903104595	0.922930163	2.951289355	2.770626234
Mnda	1.363462073	1.294712549	2.750915875	3.286377776
Mpp6	4.862810186	4.899272442	11.22759107	11.23254935
Mrap	0.217056756	0.088858099	0.816801833	0.753584682
Mrgprb1	0	0.005265665	0.101901203	0.046097414
Ms4a4d	32.11620497	31.56501864	15.71538943	15.17052441
Ms4a6d	8.794994091	9.065470428	28.50819952	28.16021183
Ms4a7	0.981010063	1.078724888	6.896368811	6.141100344
Msi1	0.063103072	0.014208129	0.302451444	0.248765543
Msln	22.67503715	22.94328708	90.33216464	88.86527107
Msr1	1.483515654	1.460668893	13.00276432	12.50370021
Msx3	0.008559061	0	0.661967951	0.62211101

Mtfr2	0.296970113	0.191042986	2.061114165	2.090568688
Mthfd2	3.640681431	3.811196821	7.61235218	8.434323627
Mxd3	7.172822706	7.684891625	20.50208675	20.23482194
Mybl1	0.683381959	0.636697515	1.638571964	1.596680225
Mybl2	0.475881026	0.482166797	2.488239109	2.514391677
Mybpc2	0.345687821	0.340524634	0.835497077	0.858387849
Mycbpap	2.446821611	2.33313204	1.172464179	1.136553946
Myrf	5.693658149	5.638147164	13.80250161	13.95094343
Naip1	0.10953874	0.106875027	0.401717125	0.445319208
Ncapd2	4.631290174	4.876998588	18.87594504	18.20623196
Ncapg	1.434480598	1.340800946	7.051870521	7.649149436
Ncapg2	1.562053288	1.521620934	6.731994836	6.883135676
Ncaph	6.982614902	6.323107988	14.93547668	13.99977957
Ndc80	1.414065011	1.451766117	9.116210826	9.170382606
Ndrg4	0.791958445	0.817278802	1.833216735	1.94316104
Necab3	0.259552348	0.206975549	0.612589499	0.599536984
Neil3	0.773252103	0.821612032	6.54922155	6.764534667
Nek11	0.745698015	0.83110249	0.276181376	0.367462245
Nek2	1.781733097	1.716348233	9.530910075	9.255160758
Nfasc	0.007543915	0.016985689	0.071904705	0.074349235
Nfe2l3	0.646330237	0.692981397	1.734994163	1.924247678
Nfil3	8.539309157	9.082439621	18.22930424	17.85602258
Ngfr	0.247124672	0.200569895	0.494569383	0.502684791
Nhs	0.200037441	0.229294179	0.495234736	0.586961536
Nid2	4.352577018	4.597769819	9.390950136	8.838110273
Nkain4	11.93018706	12.57257913	30.99665666	29.5468655
Nme4	0.748506263	0.944799435	2.643734031	1.956001955
Nnmt	5.457899778	5.0587642	14.5057805	14.10483027
Npl	1.688184832	1.38220894	6.094407377	5.60327541
Npr3	77.50537718	78.66467364	24.44621961	24.85801647
Nr1h4	0.058556415	0.122426714	0.400942043	0.391606399
Nr3c2	3.673525179	3.472401427	1.846900096	1.734701416
Nrcam	1.414967147	1.404007186	4.604335508	4.866838942
Nrep	26.01472648	25.88989745	53.49304705	54.35347981
Nrn1	4.441086821	3.722617454	1.906171324	1.917877661
Nsl1	0.612322664	0.73381905	3.28484853	3.009269203
Ntrk1	0.060508726	0.093664951	0.321325443	0.316808398
Nuf2	0.976693866	1.13620135	12.90895461	13.00489756
Nusap1	1.924392132	1.928220738	15.32217994	15.44644926
Nxnl2	0.319790959	0.284223669	1.063391191	0.829396726
Nxpe5	0.169330215	0.142972373	2.812916166	3.04562944
Nyx	0.524367183	0.456437623	0.206102993	0.25306793
Ocstamp	0.080059603	0.135195144	1.537074939	1.898601898
Oip5	0.603455041	0.551981714	3.62912595	3.422832928
Olfm2	5.76326769	5.30328439	2.502810491	2.783074707
Olfml2b	2.637349562	2.371003945	8.30866854	8.142123347
Olfr558	0.053573925	0.015078213	0.240729827	0.214499643
Orc1	0.253455625	0.168275762	1.189321017	1.088844799
Osr1	2.131691428	2.279291971	5.492403922	5.839812869
Oxtr	0	0.00964834	0.112028862	0.042232416
P2ry10	11.11326123	10.82742374	5.242081582	5.578712494
P4ha3	0.290177912	0.196007191	1.246315163	1.225652341
Padi4	2.053847231	2.253764499	11.61447915	11.18045637
Pappa	0.264497267	0.235816033	0.539500198	0.572962181
Pappa2	0.050161981	0.033369658	0.675574711	0.603921096
Parpbp	0.437028777	0.26726008	2.774083311	2.898019151
Pask	0.52387507	0.473539544	2.061886945	2.119870955
Pbk	1.260573953	1.231704967	19.59990095	19.41190189
Pcdh10	0	0	0.019882266	0.010220696
Pck1	0.007482635	0.050543139	0.839545551	0.838852673
Pcnxl2	0.154194397	0.182726438	0.562833046	0.453233905
Pdcd1lg2	0.687832722	0.516235629	1.94184455	1.963071546
Pde4c	0.606915977	0.648750389	0.3138656	0.302094705
Pdgfrl	0.94489132	0.718747829	2.072473056	2.658438329
Pdlim4	3.386318517	3.421749158	7.179584723	6.654438911
Pf4	16.51188461	14.76616075	41.01380463	38.67376081
Phex	1.371626097	1.202967037	2.74661153	2.698230101
Phf19	0.72169144	0.629959525	4.847909595	4.625797929
Pidd1	1.437718629	1.466587596	3.396946892	3.509106049
Pif1	0.122341081	0.161683105	2.636382119	2.62116411
Pkhd1l1	2.504635671	2.452955048	6.476019854	6.540256097
Pla1a	0.565223229	0.379560024	1.361031498	1.392642816
Pla2g1b	6.099323436	5.748732635	2.163185328	3.189083001
Pla2g7	9.10754279	8.826230845	25.15314139	24.61841133
Pld5	0.165886211	0.260321825	0.66804953	0.805062218

Plek2	0.681513176	0.468868265	1.635987357	1.616976029
Plekhb1	14.22260021	13.95695385	6.725511522	6.481904361
Plekhg4	0.103345477	0.148075507	0.593580012	0.619216211
Plin1	0.071514619	0.08051026	0.500797081	0.427922826
Plk1	1.315041665	1.280395588	11.10180769	10.57413059
Plk4	2.549606054	2.360864732	7.346065509	7.342511024
Pmch	0.209353721	0.294609738	2.537074313	2.417918765
Pmf1	6.689294566	6.0423507	14.78841214	15.38771302
Pnoc	0	0	0.306447673	0.380111328
Podn	6.148459728	6.902790297	15.01884715	14.94736412
Pole	0.645255243	0.581136202	3.370855073	3.32173447
Pole2	2.679233575	2.977244325	5.937683926	6.003780261
Polq	0.247199938	0.265410579	0.9025799	0.916426648
Pon1	8.590559417	7.544657481	3.283687142	2.406689479
Ppargc1a	0.48522036	0.549647878	0.203543535	0.263609607
Prc1	2.996042065	2.389445675	35.53334544	34.14324038
Prg4	2.548629523	2.560143476	10.72620928	10.74959131
Prim1	5.222439864	6.023106806	13.11638988	11.24723909
Prn	2.176231927	1.795351746	4.465250791	4.170426286
Prnd	0.292521637	0.39106416	2.509348859	2.177230961
Prok2	0.012937589	0	0.549630037	0.637533637
Prr11	0.982053304	0.811912723	6.986889161	6.786397301
Prr15	1.700904234	2.026556605	6.376775867	6.845015386
Prrx2	0.294745562	0.253745358	1.624236586	1.537873434
Prss35	0.069826063	0.069874938	2.400191365	2.551972858
Prtn3	0.948458686	0.681550776	3.187432874	3.132426224
Psat1	3.316125957	3.060913576	11.41896867	11.28773651
Psrc1	0.62930099	0.596597183	4.606115436	5.222813632
Ptger3	0.475299342	0.556926279	2.229492793	2.35411191
Ptn	0.61019203	0.545195656	1.656449188	1.873334706
Ptprq	0.038827433	0.062444909	0.154075399	0.133249317
Ptpru	22.87138049	23.10581785	6.36120669	6.720923848
Ptx3	3.259033988	2.992511447	13.97854411	14.37848983
Pycard	3.869072487	4.035780842	9.188290709	8.708403404
Pycr1	0.181370442	0.317620437	0.819545849	0.777893865
Racgap1	4.132055027	3.688379356	25.54565091	26.48290807
Rad51	1.043733073	1.102871142	6.243259861	5.786176864
Rad51ap1	1.013397506	1.080824392	7.088242154	6.820454711
Rad51b	0.24149103	0.357311663	0.616310116	0.77350673
Rad51c	0.54931662	0.545268042	1.479858228	1.375280674
Rad54b	0.235972018	0.221899503	1.345718044	1.128613564
Rad54l	1.056365117	1.103273244	2.807459317	2.806588464
Raet1a	0.532124312	0.727428628	1.38011824	1.373523925
Raet1b	0.614660501	0.745206082	1.613804928	1.572698466
Raet1d	0.323419379	0.225396152	1.157572817	1.005570118
Rag1	0.155563407	0.12556586	0.028775745	0.018079704
Rai2	8.437046767	8.742673202	4.233958433	3.826815992
Rarres1	1.539884898	1.468079371	4.201095573	3.366830499
Rbm44	0.025414922	0.02288944	0.177182669	0.269263184
Rcc1	4.407853972	4.44145387	11.05085051	10.69461991
Reep2	1.290742864	1.464543863	2.856328825	2.929824557
Reg3g	11.3046952	11.73018744	5.895458575	5.296512492
Retnla	23.04431323	26.49576131	123.0519654	117.3878265
Rfc4	4.120962713	4.258758209	9.118314096	9.419808522
Rfc5	4.401450204	4.636646399	9.391904063	9.994362392
Rgs16	4.099343102	4.614989004	10.87601145	11.76302119
Rgs22	1.074452604	1.02266626	0.601167522	0.445231668
Rgs5	2.705164855	2.813987147	12.23498852	11.34417803
Rhou	1.988269655	1.956936656	6.29488858	6.560461595
Rian	1.372790666	1.059047513	7.196975352	7.209482957
Rmi2	0.136116313	0.106087872	0.598795618	0.677198323
Rnase2a	0.056737892	0.031937404	22.89886183	20.48002892
Rnase2b	0.309479413	0.116136013	1.741785589	1.429725878
Rnf128	1.259808577	0.949977777	3.32076811	3.565228804
Ror2	0.665634091	0.754913337	1.960411029	2.229243955
Rph3a	0	0.010674162	0.087790762	0.105125884
Rpl39l	0.048632479	0.054749835	0.529759672	0.389430096
Rpp25	0.319941013	0.458418006	3.104958436	3.063607265
Rrm1	10.90142588	10.9935942	24.41162259	24.11470982
Rrm2	4.301018538	4.195080505	25.76345221	25.87353498
Rsph4a	2.895964208	2.80742902	1.402048886	1.360994581
S100a4	46.48470716	45.26598927	103.281107	101.8230332
Saa3	4.054996267	3.984055762	556.722544	541.3320923
Sapcd2	0.189598152	0.232851513	2.797564891	2.61885607
Sbsn	2.38045987	2.52565346	5.279405063	4.68369692

Scamp5	1.496770851	1.602174728	3.615057757	3.76343638
Scara3	0.764586841	0.718380539	5.279043465	5.771949633
Scgb1a1	6698.226443	6764.075922	3341.259624	3202.979651
Scgb3a1	57.606084	56.87150235	27.27095631	29.93685835
Scml2	0.156404086	0.126414799	0.576646822	0.573101073
Scn4a	0.347108977	0.347351937	0.161585532	0.146194058
Scrg1	0.335564107	0.472217324	1.370753152	1.13683632
Sdf2l1	17.61358228	17.54193596	35.65992606	36.36794233
Sdk1	0.528856075	0.56431625	1.3575675	1.376688473
Sdsl	0.12952571	0.145818418	0.70546987	0.538541907
Sel1l3	0.542292024	0.498172531	1.157821934	1.052880726
Selp	5.32920309	5.531636323	12.13751369	11.89602904
Serf1	3.290123457	4.15294692	6.588717282	8.474948951
Serpina3i	0.357037353	0.33160724	1.001482518	0.791727819
Serpinb2	1.128382805	1.098072526	17.66659222	17.411598
Serpinf1	2.109441924	2.256781587	14.64338709	14.86589541
Sez6l	0.035414523	0.018122375	0.70491582	0.690124919
Sfrp1	2.932604127	3.064588181	6.779215294	6.889129131
Sfrp2	2.357557254	2.488914896	9.345386862	10.36414315
Sfrp4	0.189287679	0.125351584	0.533677121	0.466382329
Sgol1	0.406786635	0.396894582	3.432685613	3.354276622
Sgol2	0.455390728	0.484191323	3.657478971	3.953815051
Sh2d5	0.15584851	0.126325653	0.312372622	0.422391414
Shc2	0.395745724	0.445525569	0.883736062	1.078671629
Shc4	0.101527868	0.096010991	0.309667609	0.330201421
Shcbp1	0.697780272	0.847569555	7.641012984	7.419910713
Shisa4	1.809414301	1.944424274	4.92754401	4.498710571
Siglech	2.523380485	2.785629101	1.227597038	0.995976751
Ska1	0.205353331	0.189901309	2.173026936	2.285882946
Ska2	7.414830798	6.207551671	13.98865954	14.1041557
Ska3	0.502356295	0.682556013	4.745744197	4.577526775
Skp2	1.153470874	1.09828714	2.525482859	2.361276188
Slc10a5	0.209567184	0.280866791	0.065224043	0.104499125
Slc11a1	5.284455007	6.216981546	13.40080435	11.92129101
Slc15a2	5.666078084	5.890143599	2.730175965	2.96115297
Slc16a4	0.402768989	0.282135636	0.77998452	0.871084625
Slc16a5	0.447233619	0.503489994	0.141210774	0.135745055
Slc26a4	0.609522866	0.543236277	1.486995707	1.454860858
Slc6a19	0.329051861	0.343336909	0.104909357	0.118645559
Slc7a8	1.543062513	1.311064458	4.048905636	4.052992874
Slc9a5	2.00918634	1.789277437	4.454845662	4.225384811
Slfn9	1.00512589	1.02868919	7.586856427	7.592131151
Slpi	35.10462786	33.86021022	77.79424028	77.07898115
Slurp1	4.436903188	3.232065237	21.60713117	20.34822181
Smc2	4.289410259	4.095922485	12.2783577	12.29075231
Smpd3	0.981010063	0.988831148	3.520668762	3.494478151
Snai1	4.603603838	5.085417331	9.733749439	10.05032988
Snhg11	2.059602911	2.160839845	1.050869193	0.949948494
Sntb1	15.92877459	16.06957234	7.269370589	7.302736089
Sntg1	0.010781918	0.009103611	0.058724392	0.073054753
Sntn	4.388492954	3.287749645	1.650799495	1.73081385
Soat2	0.322253081	0.176211544	0.651920857	0.70325118
Sorcs1	0.014343849	0.034987605	0.119791162	0.117805129
Sox12	0.631153083	0.819858713	1.620246366	1.647521325
Sox9	0.250229706	0.228553488	0.483440839	0.552556062
Sp6	1.078776461	1.292826095	2.394474477	2.393608234
Spag16	1.332447419	1.355903381	0.627654755	0.576741833
Spag5	0.773354397	0.818740659	7.643190143	7.817405929
Spag6	2.817912966	2.671940682	1.357536013	1.349484978
Sparc	440.5872438	447.4364932	950.2262233	975.2532851
Spata18	6.106382923	6.02648481	2.713217572	2.375589679
Spc24	0.870625925	1.127160698	6.84416902	6.649868089
Spc25	3.10570025	2.911310343	14.39492986	14.78588402
Spdl1	0.748668923	0.763825631	3.729370904	4.111990359
Speg	0.520347137	0.689285497	1.253442482	1.271962472
Spon2	4.620194227	4.551187079	13.9451478	13.81987393
Spp1	67.97972901	69.27373073	225.6773009	225.2209177
Spsb1	5.351921533	4.964415259	11.56906484	11.31512088
Spsb4	0.122779579	0.167843075	0.640066934	0.66985305
Srrm4	0.010474688	0.01474034	0.15118526	0.122589829
Stac2	0.918059666	0.917899203	1.881220207	1.882355178
Steap1	0.25756017	0.362447507	0.824155313	0.892402913
Steap4	2.082136097	2.042264731	13.36411566	12.76390055
Stil	0.363716726	0.376379423	3.177613455	3.113911478
Stk33	1.978716223	2.209203853	0.881771033	1.138246537

Ston2	7.326618632	7.50667757	3.225573134	3.223904718
Stra6l	0.276845041	0.238334867	0.597228372	0.461429556
Sult1d1	1.087476272	0.994141734	0.365177903	0.372700193
Sv2a	0.129930788	0.15190039	0.40283104	0.510983129
Syt12	0.322559334	0.275712281	0.644174505	0.61077843
Tacc3	2.609943052	2.871081327	13.89842159	13.89925941
Tarm1	0.674985272	0.621728138	3.542664015	2.872598662
Tbx18	0.175706788	0.164840414	0.519513199	0.577227506
Tcf19	4.184299563	3.666586852	17.48640168	17.38217662
Tcte1	1.204295012	1.18361764	0.458107607	0.573038352
Tdo2	0.371593239	0.418335004	1.07071145	1.461945053
Tenm3	0.142645822	0.118434296	0.464215304	0.524996871
Tesc	1.038990068	0.959139221	2.987912037	1.996759412
Tex11	0.63178922	0.637681713	0.278846093	0.355614808
Tgfbi	42.73806112	42.63357151	97.97512468	99.01618713
Tgm1	0.866073917	1.019000789	2.128026628	2.182036284
Thbs2	0.277653516	0.3832812	2.747267284	2.569466716
Thbs4	0.042792633	0.048175409	0.539396931	0.391619272
Themis	1.086656772	0.918892288	0.444560318	0.321044868
Ticrr	0.22849696	0.165367934	0.785234728	0.784163716
Timd4	1.097976042	0.921999801	2.87245437	3.237469088
Timp1	5.586451139	4.55592444	51.98936424	50.01770148
Tk1	4.375324546	3.863282706	22.9582518	21.33156581
Tm4sf19	0.077862263	0.052593815	0.508898015	0.575530067
Tmc7	0.758062694	0.701371824	1.755941669	1.749698509
Tmem108	0.673339858	0.57300477	1.62866662	1.685155778
Tmem151a	1.166039196	1.240094163	3.491646671	3.45980374
Tmem198b	13.97465225	13.54296364	26.99369424	28.49356933
Tmem212	10.34694996	10.48054627	4.669015075	5.347613474
Tmem26	0.526948122	0.553973609	1.544765363	1.503589389
Tnc	4.287107161	4.621522219	26.7916814	26.60429887
Tnfaip6	2.237443372	2.133482391	7.431691043	7.651638093
Tnfaip8l1	2.375434427	2.581055364	5.048956877	5.404142158
Tnfrsf9	0.317282586	0.337348711	0.72964198	0.716601012
Tnfsf14	1.15206154	1.07295322	2.555544786	2.29663903
Tnip3	0.488195769	0.491136086	1.403040873	1.254041689
Tnni1	0.123000009	0.059345086	0.478519453	0.476233048
Tonsl	1.302800192	1.225722171	2.823107804	2.848825952
Top2a	4.086009177	4.08886919	34.44676717	33.65058222
Tpbg	0.394436584	0.461813825	0.956720064	1.004239662
Tph1	0.008231528	0.004633475	0.143467296	0.167322419
Tpsab1	0	0.037005556	0.250646159	0.283464263
Tpsb2	0.071965342	0.040508839	2.99852252	3.701430643
Tpx2	1.632335517	1.731643824	15.71978389	15.8499662
Trabd2b	2.319248171	2.287938249	5.043104724	5.032856923
Traip	0.4717639	0.506593297	1.676099104	1.832968303
Trem2	3.399075048	3.384361505	18.90400701	19.46431639
Treml4	6.293028877	6.238500496	3.018188093	2.943514723
Trim59	2.922778692	2.588570306	10.83666952	11.17369945
Trip13	0.690765695	0.729051889	3.555372093	3.595400052
Tro	0.243162396	0.267665858	0.676915137	0.595794763
Troap	0.423280702	0.505699046	6.577518067	6.587381013
Tsnaxip1	1.603166377	1.425911134	0.675383655	0.785637605
Ttc18	1.303254458	1.067045461	0.667741155	0.507678101
Ttc29	2.045343126	1.987810067	0.913835435	0.885846483
Ttc34	0.434310836	0.488941687	0.139931051	0.14318109
Ttk	0.389356278	0.424192655	4.699641327	4.928158535
Tuba1c	7.128827759	7.345602811	21.64639019	20.60179306
Tubb2b	5.0413182	5.033381323	11.33815244	11.69350133
Tubb3	0.329461761	0.179057062	0.990032585	1.105665743
Tubb6	13.6371021	14.75859647	39.9313947	40.63826385
Tube1	0.48117762	0.45141977	1.172063884	1.185564429
Twist1	0.084062583	0.027039029	0.640992953	0.798892227
Tyms	2.030450707	2.152755446	5.66107613	5.718377879
Ube2c	3.195848635	3.550506192	41.29436814	41.52081615
Ube2s	19.51513593	20.40858271	40.68170904	40.3701721
Ube2t	1.313034015	1.380947395	7.490278085	6.151060138
Uchl1	4.624855495	4.188338482	9.406582707	9.544967966
Ucp1	0.011906675	0.026808769	0.596624108	0.674742489
Ugt1a1	6.371284022	6.381051683	12.9825573	12.92739631
Ugt1a10	6.333660382	6.341869916	12.9173448	12.86812091
Ugt1a2	6.410886317	6.413082429	13.06239297	13.01261634
Ugt1a5	6.400787706	6.409084248	13.05424933	13.00450374
Ugt1a6b	9.057401585	9.101115389	18.31921885	18.37261145
Ugt1a7c	8.242238189	8.108261027	17.61366477	17.80418163

Ugt1a9	6.384865348	6.393141253	13.02177607	12.97215422
Uhrf1	1.876499729	1.913357511	12.87779598	12.58244925
Ulbp1	1.969294579	2.120088772	7.760149174	7.662618072
Ung	0.634750098	0.725102423	2.338698219	2.403421245
Upk3b	22.74726889	23.07587209	47.8402126	49.17695243
Usp26	0.061570154	0.074646823	0	0
Vash2	0.140152073	0.184078352	0.544520876	0.587040669
Vat1l	0.042844482	0.054263003	0.28002618	0.323288859
Vcan	0.519714305	0.474071173	3.322755161	3.335903761
Vit	0.151127217	0.226849499	1.716801129	1.711080513
Vmn2r26	0.030489989	0.05148787	0.274008288	0.33805668
Vpreb3	6.633243969	6.515866946	3.046118116	3.324785573
Vps37d	0.507113287	0.542356684	1.325771615	0.968336793
Vsig4	0.136693945	0.184665992	7.474901577	6.432834602
Vsig8	0.052087741	0.070367669	0.374482855	0.282343596
Vwa1	1.327829307	1.417654555	4.298518908	4.054957574
Wasf1	0.345433639	0.25925657	0.877998751	1.115896376
Wdhd1	1.993098581	1.79504429	4.710025471	4.621889759
Wdr86	0.361680238	0.283788686	0.967048696	0.850630861
Wee1	3.057274619	3.003554469	7.090978088	6.390151241
Wfdc12	0.431990574	0.212769185	1.20584262	1.929176785
Wfdc17	12.31236461	13.29726394	114.4340194	105.6625345
Wfdc21	12.15589915	12.57808701	32.81183851	31.7124951
Wisp1	0.771757354	0.759133385	4.538849434	5.061111937
Wnt2b	2.865239748	3.157807422	1.223391966	1.106860456
Wnt4	2.944122263	3.239520361	7.245187556	7.928901449
Wnt5a	2.103830153	2.000597518	5.466016202	5.580628021
Wt1	1.012914509	1.140326439	6.847854793	6.987962482
Wt1os	0.029958024	0.013490547	0.234962279	0.295252033
Xkr5	0.043878246	0.082329297	0.626673366	0.540553894
Zbtb7c	1.126008895	1.079668655	2.835581518	2.885115107
Zbtbd6	0.059078188	0.05911954	0.507686353	0.4528584
Zfp367	10.46033766	9.660835789	20.79128356	20.40422295
Zfpm2	1.219967349	1.249372734	2.35777136	2.627698577
Zgrf1	0.608233749	0.65034849	1.397722189	1.389127722
Zranb3	0.814091205	0.962574346	1.927179282	1.92178101
Zwilch	0.907011459	0.823214108	5.162207352	4.521508091
