# Supplementary material for: Hydrostatic Pressure Controls Angiogenesis Through Endothelial YAP1 During Lung Regeneration
Source: Front Bioeng Biotechnol. 2022 Feb 18;10:823642. doi: 10.3389/fbioe.2022.823642 (PMC8896883; doi:10.3389/fbioe.2022.823642)
Supplement: Supplementary file 8 [file DataSheet1.PDF]

Supplemental Dataset

name	log2FoldChange	pvalue	padj
Rnase2a	8.904920322	24.20233178	6.2758E-25
Adamts16	8.735784635	7.553337961	2.7968E-08
Pnoc	7.590404671	5.333027398	4.6449E-06
Car6	7.539251521	5.875157996	1.333E-06
Msx3	7.139960059	5.214227573	6.1062E-06
Saa3	7.08649255	1.7976931348623157e+308	0
Arg1	6.838887731	1.7976931348623157e+308	0
H2-M11	6.730399601	3.84034907	0.00014443
Adipoq	6.587821009	8.219171818	6.0371E-09
Prok2	6.429910543	4.086299025	8.1979E-05
Gm14461	6.344980172	3.191326983	0.00064368
Chga	6.326647318	7.491198414	3.227E-08
Ecel1	6.065025539	3.538285254	0.00028954
Tpsb2	5.86656093	18.01193506	9.7289E-19
Bcat1	5.854957396	3.25008702	0.00056223
Cfd	5.796403189	46.52258732	3.002E-47
Gabrb3	5.726247441	2.341053277	0.00455981
Lipn	5.618547908	5.508001955	3.1045E-06
Ccl24	5.601701478	49.80136939	1.5799E-50
Col24a1	5.55712044	69.60480289	2.4843E-70
Cd5l	5.551097295	1.7976931348623157e+308	0
Pcdh10	5.53628181	2.076418457	0.00838652
Gpbar1	5.466534624	2.016365173	0.00963019
Vsig4	5.44135843	53.44513931	3.5881E-54
Clca2	5.395840797	16.76350586	1.7238E-17
Chl1	5.170972294	44.99912943	1.002E-45
Col11a1	5.156235154	2.348469412	0.00448261
Prss35	5.140555197	35.83928598	1.4478E-36
Car3	5.116247687	68.22722919	5.9261E-69
Ucp1	5.058804897	6.219281161	6.0356E-07
Ccl8	4.925561518	84.39722302	4.0066E-85
Pck1	4.902969211	12.93539811	1.1604E-13
Mrgprb1	4.891219857	2.02716837	0.00939359
Cxcl5	4.856637132	14.48691502	3.259E-15
Fam64a	4.709799732	230.218135	6.052E-231
Sez6l	4.674089426	25.55172989	2.8072E-26
Dlk1	4.650452861	6.504298938	3.1311E-07
Mmp10	4.608137113	4.79135932	1.6167E-05
Tph1	4.566737275	4.666102713	2.1572E-05
Ckap2	4.351528887	244.4357748	3.666E-245
Lum	4.269184802	44.33434083	4.6308E-45
Rph3a	4.237641997	2.595228915	0.00253963
Nxpe5	4.216519451	41.24323859	5.7116E-42
Pif1	4.213176351	58.92274745	1.1947E-59
Oxtr	4.064617682	2.218471338	0.00604684
Cenpf	4.06023512	1.7976931348623157e+308	0

Egr4	4.046337535	6.271667683	5.3497E-07
Fabp7	4.045466371	12.55885736	2.7615E-13
Angptl7	4.043650318	155.4343226	3.679E-156
Ocstamp	4.005131654	19.90800115	1.2359E-20
Mmp12	3.974979591	1.7976931348623157e+308	0
Serpinb2	3.970319688	215.6743899	2.116E-216
Pbk	3.961035475	193.0975169	7.989E-194
Kcnt1	3.952466992	11.58795909	2.5825E-12
Calca	3.920571628	73.04422351	9.0318E-74
Pappa2	3.918306443	32.61755013	2.4124E-33
Has2	3.915673077	19.53559768	2.9134E-20
H2-M9	3.915423043	5.840531206	1.4437E-06
Tpsab1	3.910367064	2.078473093	0.00834693
4930558J18Rik	3.897903459	3.948099603	0.00011269
Etv4	3.897658385	16.5972351	2.5279E-17
Alppl2	3.865373128	2.004110255	0.0099058
Troap	3.823976051	88.65436814	2.2163E-89
Ankle1	3.813231626	61.6974187	2.0072E-62
Iqgap3	3.808790022	1.7976931348623157e+308	0
Adamts19	3.776072315	18.69851771	2.0021E-19
Kif18b	3.774775669	131.7671669	1.709E-132
Cdc25c	3.762686847	40.83354151	1.4671E-41
Fgf23	3.716414863	4.157917555	6.9516E-05
BC030867	3.709343195	24.97390464	1.0619E-25
Prc1	3.684948619	121.1538499	7.017E-122
Sapcd2	3.680804939	53.761623	1.7313E-54
Twist1	3.65622052	6.849796252	1.4132E-07
Nuf2	3.61439595	178.0058388	9.866E-179
Ube2c	3.61428118	220.8456621	1.427E-221
Col6a5	3.608358311	47.1892999	6.467E-48
Fcna	3.596928057	182.0362351	9.2E-183
Cdkn3	3.585700906	49.1009285	7.9263E-50
Wt1os	3.577512014	5.129416968	7.4231E-06
Mirg	3.575284841	4.367710101	4.2883E-05
Igj	3.565938125	81.45981953	3.4688E-82
Ttk	3.560527622	86.03391893	9.2487E-87
Krt14	3.546467482	31.4527104	3.5261E-32
Has2os	3.50135332	2.899343611	0.00126083
Lrrn1	3.489524595	4.612240664	2.4421E-05
Cep55	3.486644481	166.4276451	3.736E-167
Ska1	3.486385704	34.03396858	9.2477E-35
Gjb5	3.479067449	24.27545688	5.3033E-25
Hmmr	3.471188007	211.6184001	2.408E-212
Srrm4	3.445967145	5.94756488	1.1283E-06
Fbp1	3.433951459	31.03322069	9.2636E-32
Cdk1	3.406031837	286.9102753	1.229E-287
Ccnb1	3.40512742	150.2567373	5.537E-151
Kif22	3.368774807	185.418602	3.814E-186
Thbs4	3.354691728	8.278504926	5.2662E-09

Cma1	3.338053777	35.10883099	7.7834E-36
Birc5	3.337748732	164.4096438	3.894E-165
Depdc1a	3.323542586	50.25821293	5.5181E-51
Timp1	3.318133078	231.0181867	9.59E-232
Pmch	3.301295448	10.32880205	4.6903E-11
Shcbp1	3.284043857	86.23725661	5.7909E-87
Lgals7	3.280410474	31.14724581	7.1245E-32
Spag5	3.274437191	159.4659646	3.42E-160
Melk	3.268108485	31.68397708	2.0703E-32
Cdc20	3.248784415	278.9861831	1.032E-279
Aspm	3.242633275	189.2509562	5.611E-190
Tpx2	3.225276823	1.7976931348623157e+308	0
Ccnb2	3.224401739	210.9861442	1.032E-211
Calcb	3.221693815	20.2741431	5.3193E-21
Xkr5	3.221207744	12.38579061	4.1135E-13
Rbm44	3.195346115	4.681764118	2.0808E-05
Cdca8	3.193676835	140.8339408	1.466E-141
Vit	3.185827245	24.52990821	2.9518E-25
Ckap2l	3.177926002	247.2483016	5.645E-248
Kifc1	3.157643425	140.6046797	2.485E-141
Kif2c	3.157350851	85.84083728	1.4427E-86
Rpl39l	3.151683069	2.159294855	0.00692955
Lrrc15	3.15094533	5.994900864	1.0118E-06
Mir147	3.138499267	46.29752836	5.0405E-47
Lrr1	3.11912839	10.54357444	2.8604E-11
Msr1	3.108149182	258.9645267	1.085E-259
Wfdc17	3.099354882	247.170826	6.748E-248
AA467197	3.094871475	87.93941758	1.1497E-88
Gjb3	3.094288124	8.388883824	4.0843E-09
Gjb4	3.087641369	5.749343558	1.781E-06
Cemip	3.084305221	29.47608161	3.3413E-30
Stil	3.082114422	86.96492655	1.0841E-87
Il10	3.077159068	14.64607957	2.259E-15
Cdca3	3.076170167	141.6895153	2.044E-142
Aurkb	3.075998472	129.2595352	5.501E-130
Sgol1	3.070918032	63.51645686	3.0447E-64
Mtfr2	3.069956474	24.17601001	6.6679E-25
Mir5134	3.065469061	5.013103272	9.7028E-06
Neil3	3.056206146	77.00807797	9.8157E-78
Lin7a	3.055292809	3.335065538	0.00046231
Plk1	3.055046062	121.3505696	4.461E-122
Top2a	3.051869161	1.7976931348623157e+308	0
Gdf6	3.050124443	13.97178575	1.0671E-14
Fhl5	3.042167264	2.294971036	0.00507025
Tm4sf19	3.036732198	3.690928189	0.00020374
Kntc1	3.035764358	114.3581424	4.384E-115
Fam83d	3.027797975	72.87921641	1.3206E-73
AI427809	3.023780382	5.520386149	3.0173E-06
Esco2	3.022746534	74.29284394	5.0951E-75

Cenpm	3.021873382	25.28367224	5.2039E-26
Zbtbd6	3.016979212	7.490418703	3.2328E-08
Cxcl1	3.012541532	260.016949	9.617E-261
Sgol2	3.012470733	101.6547268	2.214E-102
Thbs2	3.01025052	73.86491036	1.3649E-74
Cxcl13	3.004409017	91.53976547	2.8856E-92
Psrc1	2.99409191	43.00337748	9.9225E-44
Nusap1	2.990894416	226.8113917	1.544E-227
Parpbp	2.990327234	46.77133428	1.693E-47
Rpp25	2.989723591	20.76157166	1.7315E-21
Marco	2.985193172	1.7976931348623157e+308	0
Anln	2.982065713	1.7976931348623157e+308	0
Ska3	2.977916182	50.96974634	1.0721E-51
Ccl7	2.976669874	131.2938522	5.083E-132
C1qb	2.974862182	1.7976931348623157e+308	0
C1qc	2.957331121	1.7976931348623157e+308	0
Col6a6	2.956089976	20.95407672	1.1115E-21
Cdca2	2.95262836	85.80187899	1.5781E-86
Cenph	2.947980197	23.22491525	5.9578E-24
AF357359	2.939421329	5.784164696	1.6437E-06
Kif14	2.931595832	65.86300296	1.3709E-66
Mastl	2.929395839	82.03190572	9.2917E-83
Prr11	2.929216834	124.0467201	8.98E-125
Arhgef39	2.923497339	62.90880297	1.2337E-63
C3ar1	2.906644361	148.90498	1.245E-149
Vmn2r26	2.90601796	4.019180131	9.568E-05
Knstrn	2.904470376	155.4427723	3.608E-156
Mki67	2.9043846	1.7976931348623157e+308	0
Col5a3	2.900589425	232.3980351	3.999E-233
Kif11	2.894980198	284.127988	7.448E-285
Slfn9	2.893791971	142.3646101	4.319E-143
Scara3	2.88861879	88.61801068	2.4098E-89
Dlgap5	2.884714085	97.44503729	3.5889E-98
Clgn	2.881196911	3.115121201	0.00076715
Rnase2b	2.868844571	5.729123963	1.8658E-06
Dtl	2.858302419	86.64143697	2.2833E-87
Mis18bp1	2.856868528	77.42358384	3.7706E-78
Clspn	2.847166217	94.06698986	8.5706E-95
Adamts4	2.819865721	56.02371793	9.4685E-57
Clhc1	2.814101381	10.32190987	4.7653E-11
C1qa	2.813949759	1.7976931348623157e+308	0
Cyp26a1	2.813836586	3.982564493	0.0001041
Phf19	2.79938604	81.94123403	1.1449E-82
Msi1	2.79610332	2.15078562	0.00706666
Cenpe	2.792643358	186.3981026	3.999E-187
Lhx2	2.784705109	2.204595152	0.00624317
Prnd	2.77914371	33.41837163	3.8162E-34
Spc24	2.756104943	41.2554612	5.5531E-42
4933413G19Rik	2.752974725	14.27840205	5.2674E-15

Serpinf1	2.751712132	101.5990833	2.517E-102
Uhrf1	2.742081075	210.906204	1.241E-211
Ccl12	2.736066668	14.524583	2.9883E-15
Cyp1b1	2.735881164	95.44301112	3.6057E-96
Vcan	2.735205998	217.0193601	9.564E-218
Rad51ap1	2.726869167	58.57471693	2.6625E-59
Racgap1	2.724370155	1.7976931348623157e+308	0
Sntg1	2.715350701	2.394927795	0.00402784
Cpz	2.709597061	27.62288058	2.383E-28
Cdca5	2.707077244	45.22959748	5.8939E-46
Folr2	2.701839015	56.49622232	3.1899E-57
Foxm1	2.698300158	218.6110103	2.449E-219
Olfr558	2.695371353	3.091798008	0.00080947
Cacna1g	2.688616252	5.896372299	1.2695E-06
Casc5	2.6871126	82.33430374	4.6312E-83
Ect2	2.680791802	145.3741709	4.225E-146
Wt1	2.680046325	94.63457951	2.3196E-95
Bub1	2.677895995	119.1933512	6.407E-120
Cenpi	2.673467427	53.37672499	4.2002E-54
Aurka	2.673448201	104.8278732	1.486E-105
Hist1h1b	2.672902051	3.129687737	0.00074184
Ndc80	2.66774597	90.28544175	5.1827E-91
Ms4a7	2.658821214	48.46017688	3.466E-49
Steap4	2.656582288	177.8943909	1.275E-178
Ccna2	2.652302791	1.7976931348623157e+308	0
C330027C09Rik	2.646992189	155.087694	8.172E-156
Cdhr1	2.643313719	3.266046219	0.00054194
Wisp1	2.640776662	99.97350688	1.063E-100
Gpr133	2.638554411	205.6697219	2.139E-206
H1fx	2.638064277	29.83761338	1.4534E-30
Ankrd55	2.635897877	9.261319784	5.4787E-10
Vat1l	2.633450223	5.064660922	8.6167E-06
Ccnf	2.632375442	72.8692271	1.3514E-73
Ceacam19	2.62865756	3.603901583	0.00024894
Bai2	2.628280939	18.14485202	7.1639E-19
2810408I11Rik	2.62190431	3.037011596	0.00091831
2810417H13Rik	2.616419921	127.6925725	2.03E-128
Plin1	2.608697589	4.105414155	7.8449E-05
Gpr84	2.602240125	4.825609974	1.4941E-05
Oip5	2.60117028	24.08452093	8.2315E-25
Rrm2	2.596229255	239.49231	3.219E-240
Cdc6	2.595043145	53.00538833	9.8767E-54
Gpr88	2.59201557	13.8755251	1.3319E-14
Fcrlb	2.589298217	6.984496825	1.0363E-07
Nfasc	2.588443673	3.426265291	0.00037474
2610318N02Rik	2.579397471	3.430275998	0.0003713
Tnc	2.578537497	1.7976931348623157e+308	0
E2f8	2.568939681	68.75605326	1.7537E-69
Diap3	2.560537997	77.47964242	3.314E-78

Rian	2.554025837	97.52052198	3.0163E-98
Espl1	2.520513938	143.3078018	4.923E-144
Prrx2	2.517424044	7.71091255	1.9458E-08
Chil4	2.509571873	10.52193406	3.0065E-11
E2f7	2.509449492	74.07863237	8.3439E-75
Meg3	2.507247736	148.8880449	1.294E-149
Fignl1	2.50177385	61.97159217	1.0676E-62
Exo1	2.500767079	23.36235965	4.3415E-24
Mir1906-2	2.498701573	7.988830341	1.0261E-08
Mir1906-1	2.498701573	7.988830341	1.0261E-08
Dnph1	2.497027172	12.80364328	1.5717E-13
Trem2	2.492824582	90.75238565	1.7685E-91
Gtse1	2.492091415	50.18050257	6.5993E-51
Cd163	2.489700965	85.54532343	2.8489E-86
Rad51	2.481793006	52.38612928	4.1103E-53
Kif15	2.481126119	93.31688253	4.8208E-94
Zwilch	2.476716221	52.46459661	3.4309E-53
Camk1g	2.465660976	2.81483851	0.00153166
1190002F15Rik	2.457115113	15.70209804	1.9856E-16
Pole	2.439105287	94.39645409	4.0137E-95
Slurp1	2.438365583	40.95915051	1.0986E-41
Arsi	2.433461642	12.72825433	1.8696E-13
Cd109	2.431528237	37.94429362	1.1369E-38
Fsbp	2.430582703	11.1953192	6.3779E-12
Rad54b	2.427346725	33.22238136	5.9926E-34
Vsig8	2.42638093	2.668907357	0.00214335
4930427A07Rik	2.420183893	41.87336137	1.3386E-42
Orc1	2.418011061	13.34692129	4.4986E-14
Nek2	2.417732643	118.4914592	3.225E-119
Tk1	2.417578305	112.2024412	6.274E-113
Kifc5b	2.415342246	43.2835606	5.2052E-44
Ebf2	2.410022729	3.32238145	0.00047601
Lctl	2.399791428	9.664196224	2.1667E-10
Padi4	2.399624458	115.3083554	4.916E-116
Ccdc18	2.399003338	19.26851711	5.3887E-20
Ncapg	2.396351052	101.4597971	3.469E-102
Mcm10	2.390767035	37.39441975	4.0326E-38
2700099C18Rik	2.385550691	33.72722642	1.874E-34
Rmi2	2.383661885	9.432800431	3.6915E-10
Mybl2	2.378098663	36.61098037	2.4492E-37
Dscc1	2.373551713	4.229603987	5.8938E-05
Depdc1b	2.371395425	29.01248223	9.7167E-30
Chek1	2.369360081	31.0429939	9.0575E-32
Spdl1	2.367171323	37.74924953	1.7814E-38
Tnni1	2.36518014	2.24447332	0.00569543
Cenpn	2.34949036	31.47728333	3.3321E-32
Brca1	2.343657054	67.96809934	1.0762E-68
Tacc3	2.338023024	135.1359015	7.313E-136
Ube2t	2.336254191	27.91854782	1.2063E-28

Mrap	2.334531564	3.075773422	0.0008399
Col1a1	2.333202559	1.7976931348623157e+308	0
P4ha3	2.330524179	11.02260981	9.4927E-12
Ildr2	2.327489594	141.0482023	8.949E-142
Trip13	2.326942967	31.18010346	6.6054E-32
Ccl11	2.308347147	6.621590116	2.3901E-07
Cdh3	2.300699496	28.84455637	1.4304E-29
Dcn	2.300396431	1.7976931348623157e+308	0
Tarm1	2.299878968	11.41940816	3.8071E-12
Ercc6l	2.292772532	35.45838054	3.4803E-36
Folr1	2.286189758	7.105222639	7.8483E-08
Ccne1	2.284943335	30.04094471	9.1003E-31
Sorcs1	2.280609181	3.635386193	0.00023153
Retnla	2.275514186	220.5536221	2.795E-221
Col3a1	2.272270998	1.7976931348623157e+308	0
Plekhg4	2.271450176	9.339714209	4.5739E-10
Brinp1	2.271170816	22.81878528	1.5178E-23
Spc25	2.269523138	84.08551568	8.2127E-85
Gdf3	2.265739239	2.601158367	0.0025052
Has1	2.264377612	95.1320618	7.378E-96
Ccl9	2.262674939	1.7976931348623157e+308	0
Wfdc12	2.256648409	3.97105042	0.00010689
Gpr176	2.246511651	12.27446079	5.3154E-13
Adamts12	2.243817909	45.42294738	3.7762E-46
Kif23	2.232908736	199.8205106	1.512E-200
Cenpw	2.228533996	8.209285518	6.1761E-09
Ccr5	2.227490368	122.1091068	7.778E-123
Nsl1	2.223357403	32.54306089	2.8638E-33
Fn1	2.215009864	1.7976931348623157e+308	0
Mcpt4	2.210184058	7.286207871	5.1736E-08
Kif20b	2.196474349	113.4063836	3.923E-114
Cks1b	2.194244131	63.86232075	1.373E-64
Sdsl	2.174183342	2.821734779	0.00150753
Ptx3	2.172625853	92.39500458	4.0271E-93
Spsb4	2.172350468	5.272792277	5.3359E-06
Gatm	2.16222711	63.9415369	1.1441E-64
Kif4	2.156605976	86.2715914	5.3507E-87
Egln3	2.156029345	32.59336372	2.5506E-33
Asf1b	2.155460615	51.1581045	6.9486E-52
Ccdc80	2.151875266	1.7976931348623157e+308	0
Gfpt2	2.148480917	149.4192033	3.809E-150
Capn6	2.147343334	25.34909929	4.4761E-26
Ptger3	2.147196384	15.94718074	1.1293E-16
Tcf19	2.141546781	99.77785871	1.668E-100
Nr1h4	2.139984577	3.265932923	0.00054208
Ncapg2	2.135069493	149.4470345	3.572E-150
Apitd1	2.134321597	11.7179574	1.9144E-12
Igdcc4	2.114699238	19.83325467	1.4681E-20
Dpf1	2.114657539	3.973176157	0.00010637

Lrrn2	2.112728932	31.93764175	1.1544E-32
Igf1	2.110617183	192.7523726	1.769E-193
Itgam	2.109277096	1.7976931348623157e+308	0
Chaf1b	2.101938949	48.16398483	6.8551E-49
Lypd1	2.099548723	14.36905241	4.2751E-15
Cdc45	2.094071333	48.907754	1.2366E-49
Alox15	2.093185279	102.7299147	1.862E-103
Cpxm1	2.092071944	144.780471	1.658E-145
Rgs5	2.08986819	68.42792624	3.7331E-69
Cfb	2.088072861	1.7976931348623157e+308	0
Fbn2	2.085546109	6.740799967	1.8164E-07
Col15a1	2.07875434	235.177801	6.64E-236
Col1a2	2.077655025	1.7976931348623157e+308	0
Mettl21e	2.076419979	8.771001059	1.6943E-09
Prg4	2.065100458	147.3099844	4.898E-148
Fancd2	2.062788014	29.23844506	5.775E-30
Gas2l3	2.058891778	52.87808169	1.3241E-53
Pask	2.058714908	34.57943999	2.6337E-35
Cenpp	2.052580683	13.54692781	2.8384E-14
Ntrk1	2.052405074	2.860706812	0.00137814
Hells	2.040923272	59.6777313	2.1002E-60
A2m	2.040346376	5.00398465	9.9087E-06
Fcrls	2.031451073	47.36207745	4.3443E-48
Bub1b	2.028825313	143.4801169	3.31E-144
Gen1	2.028137134	14.54001494	2.8839E-15
Tubb3	2.023096801	5.547478908	2.8348E-06
Inhba	2.019374985	8.426202655	3.748E-09
Sfrp2	2.01751637	58.535597	2.9134E-59
Ctsk	2.016918862	299.8389917	1.449E-300
Aldh1a3	2.013196585	17.78678083	1.6339E-18
Scml2	2.012118282	8.964848158	1.0843E-09
Insc	1.995758397	2.397522243	0.00400385
Trim59	1.988304752	92.76175351	1.7308E-93
Ticrr	1.981119186	16.58251089	2.6151E-17
Crmp1	1.978548409	12.96071738	1.0947E-13
Cd276	1.973711492	35.23207136	5.8604E-36
Apol7a	1.970684525	5.179770971	6.6104E-06
Gpr39	1.969245833	19.03129395	9.3048E-20
Msln	1.967584566	1.7976931348623157e+308	0
Raet1d	1.965689102	4.312940989	4.8647E-05
Lgi2	1.962033965	22.18906862	6.4704E-23
Naip1	1.960490101	7.089570008	8.1364E-08
Ncapd2	1.958161541	231.1443499	7.172E-232
Kif20a	1.955562372	189.5931008	2.552E-190
Cd248	1.954786172	62.79262232	1.612E-63
Ak5	1.945378071	9.192551962	6.4187E-10
Gins2	1.943533349	28.21329885	6.1193E-29
Prtn3	1.941602358	9.089478221	8.1381E-10
Dctd	1.935965352	10.00040229	9.9907E-11

Cst9	1.934751763	3.440760496	0.00036244
Adam12	1.933864767	102.396384	4.014E-103
Ebf3	1.927371891	6.690036685	2.0416E-07
Cxcl10	1.922258634	43.98902844	1.0256E-44
Npl	1.919690853	23.2236651	5.975E-24
Mir6978	1.917447566	8.967051369	1.0788E-09
Fam20c	1.915136467	78.05440102	8.8226E-79
Tenm3	1.910191728	15.45780112	3.485E-16
Ulbp1	1.910157009	40.92648821	1.1844E-41
Kcp	1.908525086	14.21322158	6.1204E-15
Insl6	1.902297809	2.171680724	0.00673472
Cilp	1.892566403	12.45921039	3.4737E-13
Itgb8	1.885051283	5.124021531	7.5159E-06
Dock3	1.884062196	3.764615417	0.00017194
C3	1.863973557	1.7976931348623157e+308	0
C1qtnf6	1.862815584	103.9651778	1.083E-104
Brip1	1.852943713	27.42951619	3.7195E-28
G530011O06Rik	1.849717553	7.992326348	1.0178E-08
Cenpa	1.846695265	92.97750435	1.0532E-93
Ccr3	1.840745227	12.07291905	8.4544E-13
Traip	1.836671105	13.18268223	6.5663E-14
Ddias	1.834599953	17.20727444	6.2048E-18
Smpd3	1.825987967	49.64943948	2.2416E-50
Psat1	1.823986551	75.2309071	5.8762E-76
Prr15	1.822930045	24.09647655	8.008E-25
Polq	1.821923012	21.44839399	3.5613E-22
Ccna1	1.818746545	2.105374789	0.00784558
Il31ra	1.815938741	4.819987302	1.5136E-05
Mcm5	1.815819333	152.888387	1.293E-153
Cdkn2c	1.810657333	160.1484772	7.104E-161
Vash2	1.801488425	6.461048348	3.459E-07
Col5a1	1.801055251	1.7976931348623157e+308	0
Cks2	1.800233015	47.03874348	9.1465E-48
Crb2	1.79798762	17.65430611	2.2166E-18
Ung	1.797949873	13.45250764	3.5277E-14
Eln	1.794698562	1.7976931348623157e+308	0
Capn11	1.792196965	8.219881985	6.0272E-09
Mlxipl	1.792063179	15.00081521	9.9812E-16
Hcn1	1.79162586	3.640203291	0.00022898
Pld5	1.78962688	7.150965652	7.0637E-08
Gm5577	1.782901681	3.337865692	0.00045934
Tnfaip6	1.779042105	31.93133324	1.1713E-32
Fras1	1.76534751	41.34613351	4.5068E-42
Lmnb1	1.762381115	220.3884533	4.088E-221
Gins1	1.76184105	12.95301297	1.1143E-13
Adamts2	1.758814508	280.9402591	1.147E-281
Aoah	1.758359443	15.52063845	3.0155E-16
Chaf1a	1.747610141	57.2107327	6.1556E-58
Hapln1	1.747121447	22.87163275	1.3439E-23

Col5a2	1.745025937	1.7976931348623157e+308	0
Ephb2	1.744277546	5.972321498	1.0658E-06
Nrcam	1.741083427	87.79962357	1.5863E-88
Emilin2	1.725182609	267.824515	1.498E-268
Ccl2	1.723887205	105.0497749	8.917E-106
Fndc5	1.721430427	2.275425258	0.00530365
C4b	1.720123859	1.7976931348623157e+308	0
Clec11a	1.713836722	38.83678449	1.4562E-39
Mmp13	1.709984702	14.38717962	4.1003E-15
Spp1	1.709649115	1.7976931348623157e+308	0
Mmp2	1.7095893	1.7976931348623157e+308	0
Olfml2b	1.707213855	60.43143168	3.7031E-61
Wasf1	1.707046259	6.12848154	7.4391E-07
Aspn	1.704181307	51.91431645	1.2181E-52
F2rl3	1.699494643	5.461722589	3.4536E-06
Dcstamp	1.69850957	5.559801559	2.7555E-06
Rhou	1.696848446	52.61101892	2.449E-53
Enpp3	1.695280518	25.22344301	5.978E-26
Sv2a	1.691324115	4.470633253	3.3835E-05
Arnt2	1.691026323	4.444978288	3.5894E-05
Fam132b	1.687637264	4.985284012	1.0345E-05
Shc4	1.687230033	3.994534544	0.00010127
Pdcd1lg2	1.685204551	15.32196723	4.7647E-16
Pycr1	1.682768898	5.359621114	4.369E-06
Tbx18	1.678346841	6.399778179	3.9831E-07
Tdo2	1.673717592	4.931443076	1.171E-05
Gmnn	1.670760722	43.27827053	5.269E-44
Mir675	1.665940013	3.340619874	0.00045644
Ms4a6d	1.659845526	90.78820204	1.6285E-91
Cenpq	1.654921272	35.21576111	6.0847E-36
Sfrp4	1.654895447	2.252339619	0.0055932
Gria2	1.650143472	6.490155979	3.2348E-07
Gpr173	1.648234979	2.087431402	0.00817652
Mfap4	1.643951359	1.7976931348623157e+308	0
Mms22l	1.642172974	29.77933984	1.6621E-30
Lgr5	1.641500338	2.815591516	0.001529
Nxnl2	1.641241028	2.75474519	0.00175896
Scrg1	1.636132169	2.222318283	0.00599352
Frzb	1.631347297	2.524059876	0.00299185
Rnf128	1.627611435	22.86766217	1.3562E-23
Gata4	1.624603044	6.855033092	1.3963E-07
Gli1	1.623020455	23.03960439	9.1284E-24
Mmp14	1.619993814	1.7976931348623157e+308	0
H19	1.61858299	27.02671693	9.4034E-28
F5	1.611986057	64.80393046	1.5706E-65
Flnc	1.611479219	48.93624088	1.1581E-49
Mmp23	1.609423854	26.54025179	2.8824E-27
Ptn	1.601182253	8.117739185	7.6254E-09
Vwa1	1.60050439	29.24201677	5.7277E-30

Hmgb2	1.591572542	266.4825027	3.292E-267
Spon2	1.59120858	68.78212766	1.6515E-69
Pcnxl2	1.591028018	7.968059952	1.0763E-08
Bard1	1.587569735	19.22107302	6.0107E-20
Timd4	1.585801112	14.19289742	6.4136E-15
Lgals1	1.585407223	1.7976931348623157e+308	0
Col6a2	1.578207976	1.7976931348623157e+308	0
Plk4	1.572656794	55.62414268	2.3761E-56
Adra2a	1.572371105	3.738350521	0.00018266
4930579G24Rik	1.565650582	18.47706253	3.3338E-19
Mcm2	1.564381142	134.1796654	6.612E-135
Bean1	1.56419505	7.189532133	6.4635E-08
Arhgap11a	1.560608244	82.1693033	6.7717E-83
Mdk	1.559474066	30.88113572	1.3148E-31
Ror2	1.554918424	17.1471567	7.126E-18
Mest	1.554129897	149.7411071	1.815E-150
Hmox1	1.549319509	242.9011091	1.256E-243
Inpp5j	1.549114135	3.730410007	0.00018603
Smc2	1.543404226	131.646292	2.258E-132
Chtf18	1.542907096	20.68443211	2.0681E-21
Tuba1c	1.539752735	84.5265307	2.9749E-85
Ccsap	1.539256152	17.54675833	2.8395E-18
Ackr1	1.532666437	6.341268761	4.5575E-07
Mad2l1	1.531908714	47.87823815	1.3236E-48
Adam8	1.531736125	140.7368895	1.833E-141
Pla1a	1.529199054	5.586457278	2.5914E-06
Mfap5	1.526339277	98.20669755	6.213E-99
Tmem151a	1.525231857	28.72995497	1.8623E-29
Greb1l	1.519797597	3.711216706	0.00019444
Cenpk	1.516831306	11.81418095	1.534E-12
Clca3	1.515960284	26.98374582	1.0381E-27
2610524H06Rik	1.515457982	8.851623317	1.4073E-09
Col14a1	1.509625857	138.1048273	7.855E-139
Ptprq	1.507999736	2.224231314	0.00596717
Kcng1	1.502681227	2.124341144	0.00751033
Mmp19	1.501008793	266.1027314	7.893E-267
Tubb6	1.499149772	127.5813481	2.622E-128
Chil3	1.499077034	1.7976931348623157e+308	0
Slc7a8	1.495622942	31.12315577	7.5309E-32
Pdgfrl	1.494174791	6.839215705	1.4481E-07
Gsg2	1.49410844	15.32250474	4.7588E-16
Tmem26	1.490356687	15.76797396	1.7062E-16
Lif	1.490152288	62.2402691	5.7508E-63
Incenp	1.486767702	96.74039756	1.818E-97
Plek2	1.486305988	5.038435737	9.153E-06
Wdr86	1.483713386	3.420130271	0.00038008
Bora	1.481324159	22.4136949	3.8575E-23
Kpna2	1.480506906	127.1891174	6.47E-128
F13a1	1.480454103	156.3105762	4.891E-157

Fbln2	1.473376315	218.9832452	1.039E-219
H2afx	1.470052108	64.03903162	9.1405E-65
Steap1	1.468169925	2.238199619	0.0057783
F10	1.467063755	77.77677651	1.672E-78
Pla2g7	1.465626587	90.61075679	2.4504E-91
Dmkn	1.462484017	32.50462817	3.1288E-33
Cela1	1.461021816	4.380797242	4.161E-05
Fbxo5	1.457680134	32.77494392	1.679E-33
Cfp	1.457591115	122.3745552	4.221E-123
Aldh1l2	1.456157149	5.677207997	2.1028E-06
Cxcl3	1.452217726	7.674847388	2.1142E-08
Dbf4	1.452207744	40.20556648	6.2292E-41
Cdc7	1.451686886	33.14665542	7.1342E-34
Mxd3	1.44988499	49.94207298	1.1427E-50
Fscn1	1.445338316	163.3133012	4.861E-164
Car13	1.44489084	10.64396196	2.2701E-11
Nfe2l3	1.443701281	9.03527808	9.2198E-10
Kcnd1	1.44312323	2.061897131	0.00867167
Nme4	1.442022199	3.795304854	0.00016021
Fndc1	1.43912964	220.2741319	5.319E-221
Tyms	1.438100658	40.89593952	1.2708E-41
Nnmt	1.436089333	25.83274931	1.4698E-26
Tnip3	1.434550355	9.62299104	2.3824E-10
Eno2	1.431024369	7.316621098	4.8237E-08
Adamts3	1.430915825	10.53395614	2.9244E-11
Soat2	1.425259673	2.712396082	0.00193912
Cercam	1.42132118	19.83015555	1.4786E-20
Wnt5a	1.42058217	44.52921516	2.9565E-45
Efna5	1.415295301	18.02900443	9.354E-19
Cdt1	1.408441927	38.94608944	1.1322E-39
E2f1	1.407949737	39.96661783	1.0799E-40
Akr1b8	1.405518796	34.72334412	1.8908E-35
Apoe	1.405200882	1.7976931348623157e+308	0
Dsn1	1.4040131	23.70660271	1.9652E-24
Grin2c	1.401409872	3.903205738	0.00012497
Tmem108	1.400862002	11.14979422	7.0828E-12
Lmnb2	1.399824866	98.9866634	1.031E-99
Fstl1	1.397935993	1.7976931348623157e+308	0
Hpgds	1.395592116	6.864146688	1.3673E-07
Fbn1	1.390406002	1.7976931348623157e+308	0
Pkhd1l1	1.385507254	140.6132885	2.436E-141
Fam180a	1.377882185	10.91364886	1.22E-11
Wfdc21	1.377679841	25.27716374	5.2825E-26
Rad51c	1.377137372	8.640454971	2.2885E-09
Serpina3i	1.375016871	3.622427941	0.00023855
Gpr64	1.373290388	13.94840673	1.1261E-14
Rad54l	1.372422251	15.55080164	2.8132E-16
Rgs16	1.372013809	41.69544868	2.0163E-42
Zbtb7c	1.367375508	23.17366085	6.7041E-24

Sh2d5	1.367333079	2.171082488	0.006744
Necab3	1.366889038	2.135999814	0.00731139
Cav3	1.361480617	2.976976395	0.00105444
Osr1	1.355228635	19.61700521	2.4154E-20
Bnc1	1.348388281	13.23050035	5.8817E-14
Dbn1	1.344429485	58.34111568	4.5592E-59
Slc26a4	1.343099323	7.945805616	1.1329E-08
Bok	1.342590543	31.46549572	3.4238E-32
Cfi	1.341911162	2.672614912	0.00212513
Pf4	1.341189393	37.14285385	7.1969E-38
B4galt6	1.334938822	31.39447431	4.032E-32
5730559C18Rik	1.334903876	2.455316513	0.00350496
Loxl2	1.334604837	154.3841342	4.129E-155
Fam167b	1.333925121	3.618382817	0.00024078
Kdelr3	1.333136059	35.94609699	1.1321E-36
Tube1	1.330069493	6.063095886	8.6478E-07
Nhs	1.327935516	4.972882554	1.0644E-05
Rarres1	1.325698439	8.541208584	2.876E-09
Blm	1.324730735	18.82641245	1.4914E-19
Doc2b	1.324342026	3.009170651	0.00097911
Shisa4	1.323897922	9.485959465	3.2662E-10
Dhfr	1.322835336	24.41386548	3.856E-25
Efcab11	1.320091016	3.342827394	0.00045412
Basp1	1.317595894	28.29965749	5.0158E-29
Sdk1	1.317025895	19.4009819	3.9721E-20
Tesc	1.314437435	3.716419309	0.00019212
Tro	1.313342067	7.660645175	2.1845E-08
Lhfpl2	1.312985395	75.97835438	1.0511E-76
Ifi204	1.312227779	42.15015091	7.077E-43
2810408A11Rik	1.31087434	4.703681182	1.9784E-05
Adra1b	1.30967959	3.468535935	0.00033999
Adamtsl2	1.308678136	224.872936	1.34E-225
Mapk4	1.302069109	2.127894998	0.00744912
Nkain4	1.299770116	45.02200323	9.506E-46
Ephb1	1.299658045	8.560468008	2.7513E-09
Mybpc2	1.296409363	5.213249946	6.12E-06
Fam110c	1.292975387	5.613467675	2.4352E-06
Wdhd1	1.292398842	30.20985449	6.168E-31
Rcc1	1.290901462	40.3030949	4.9763E-41
Wnt4	1.289208067	41.02379951	9.4667E-42
Hmgb3	1.288569171	25.25859997	5.5132E-26
Col18a1	1.28727949	251.1660319	6.823E-252
Col16a1	1.285705364	147.5431293	2.863E-148
Mybl1	1.285595707	13.0323457	9.2823E-14
Myrf	1.285378524	117.7073274	1.962E-118
D430020J02Rik	1.28506432	7.300379848	5.0075E-08
Lig1	1.283000384	65.16633318	6.8182E-66
2700094K13Rik	1.28207305	37.43368834	3.6839E-38
Cadm3	1.277012088	4.356049519	4.405E-05

Col6a4	1.273137966	2.85090031	0.00140961
Aldh1a2	1.268656134	68.12182301	7.554E-69
Col6a3	1.266700186	1.7976931348623157e+308	0
A730017C20Rik	1.266364672	2.214202938	0.00610657
Mir6950	1.263704721	5.52276326	3.0008E-06
Mex3a	1.258374713	9.320023134	4.786E-10
Dclk1	1.258070082	17.7492901	1.7812E-18
Tmc7	1.256268327	12.27521695	5.3062E-13
Slc16a4	1.255575351	2.872247851	0.001342
Fen1	1.254641504	40.33327184	4.6422E-41
Col6a1	1.254463373	1.7976931348623157e+308	0
Gm16548	1.254153817	16.6471488	2.2535E-17
Cenpl	1.253785364	19.83905463	1.4486E-20
Bdkrb1	1.252483351	2.504758255	0.00312782
Adamts7	1.252302795	18.35748396	4.3905E-19
D030025P21Rik	1.251618602	17.96588494	1.0817E-18
Hhip	1.249289518	47.57464888	2.6629E-48
Dfna5	1.247432989	2.783757355	0.00164529
Scamp5	1.245706732	17.82006164	1.5133E-18
Pidd1	1.24304705	15.34031826	4.5675E-16
Ezh2	1.237735049	49.8567222	1.3908E-50
Pmf1	1.236198428	21.79106043	1.6179E-22
Ano9	1.236051803	4.238109102	5.7795E-05
Cygb	1.23028475	40.06244118	8.6608E-41
Col28a1	1.22937906	2.86842097	0.00135388
Chrd	1.229302508	7.854195418	1.399E-08
Raet1b	1.22556148	3.124498741	0.00075076
Ndrg4	1.223942064	8.458628661	3.4783E-09
A530064D06Rik	1.223706496	6.613544932	2.4348E-07
Adamts17	1.221399007	22.37722409	4.1954E-23
Lama1	1.218589546	25.22190626	5.9992E-26
Cadm4	1.215575389	13.2279862	5.9158E-14
Shc2	1.21541968	5.580529012	2.6271E-06
Emr1	1.212821993	144.1221231	7.549E-145
Rad51b	1.212415569	2.851260315	0.00140844
Loxl1	1.204887991	216.6533833	2.221E-217
Dok2	1.204084727	30.12340052	7.5266E-31
4833403I15Rik	1.20221755	2.473740071	0.00335939
Mafb	1.201724768	77.85856047	1.385E-78
Lrrc25	1.201672448	15.8542043	1.3989E-16
Abat	1.201107209	12.0134972	9.694E-13
Fhl2	1.200735977	2.904449416	0.00124609
Tgfbi	1.199548513	1.7976931348623157e+308	0
Fcgr2b	1.197277827	125.0660397	8.589E-126
Mdga1	1.197153456	4.983853172	1.0379E-05
Mpp6	1.195591036	35.36619942	4.3033E-36
Podn	1.194320428	62.68379091	2.0711E-63
Aoc3	1.192462412	14.12435709	7.5101E-15
Tpbg	1.19107711	5.291103557	5.1156E-06

Abcb1b	1.190652732	24.31438406	4.8486E-25
Gxylt2	1.190101213	6.866938159	1.3585E-07
Tgm1	1.188997032	9.05673858	8.7753E-10
Fabp4	1.187107261	71.21587304	6.0831E-72
Ccdc34	1.186604158	16.8526312	1.404E-17
Tubb2b	1.185896026	30.80733727	1.5583E-31
Slc9a5	1.184282073	29.29260341	5.098E-30
Mmp8	1.182445952	30.2693236	5.3787E-31
Sfrp1	1.182434433	40.8029802	1.5741E-41
Cit	1.177563377	29.86811868	1.3548E-30
Gas1	1.177445794	52.65104331	2.2333E-53
Mnda	1.174192687	6.754221187	1.7611E-07
Ccne2	1.173527705	30.66421226	2.1666E-31
Pycard	1.173509099	25.84622805	1.4249E-26
Dut	1.170660276	29.50043045	3.1591E-30
Mefv	1.170435218	11.03240199	9.2811E-12
Sox12	1.168673741	9.22599386	5.943E-10
Clec10a	1.167880848	11.6074007	2.4694E-12
Cd14	1.166607384	149.8464158	1.424E-150
Kcnk13	1.162141931	12.15192725	7.0481E-13
Slpi	1.160008164	86.26001784	5.4952E-87
Mcoln2	1.159846324	7.078802013	8.3406E-08
Tonsl	1.157748854	16.14153399	7.2188E-17
Fjx1	1.154249895	6.221715247	6.0018E-07
D430041D05Rik	1.153620693	2.800363078	0.00158357
S100a4	1.153619028	66.06442853	8.6213E-67
Kif18a	1.152088698	15.03313052	9.2655E-16
Wee1	1.147413024	28.36463694	4.3188E-29
Ngfr	1.145014116	2.436715591	0.00365834
Lingo1	1.143988352	6.032718866	9.2743E-07
Pappa	1.143620415	8.150186111	7.0764E-09
Rrm1	1.141780664	119.0587921	8.734E-120
Spsb1	1.141758626	43.41660602	3.8317E-44
Zgrf1	1.14142218	13.05878787	8.734E-14
Selp	1.140102652	52.48821244	3.2493E-53
Clec4d	1.139347221	39.64521	2.2635E-40
Rfc4	1.139110316	14.91735125	1.2096E-15
Tnfrsf9	1.138185691	2.325114412	0.00473027
Ffar2	1.136693523	6.757966597	1.746E-07
Cdh2	1.13598789	13.89634624	1.2696E-14
Slc11a1	1.135054325	29.22919296	5.8994E-30
Matk	1.132383731	7.173739253	6.7029E-08
Clip3	1.127777073	12.07291905	8.4544E-13
Raet1a	1.127097368	2.861740567	0.00137486
Vps37d	1.125864803	2.38060932	0.00416285
Dkk2	1.125806335	3.304885956	0.00049558
Chek2	1.125611969	17.17726507	6.6487E-18
Mcm3	1.123977652	99.23094747	5.876E-100
Ear6	1.123288193	2.534605286	0.00292008

Trabd2b	1.122107254	42.14334476	7.1888E-43
Mcm6	1.121527281	125.2048992	6.239E-126
Il6	1.121109578	21.21812265	6.0517E-22
Arhgap19	1.120903056	26.54075183	2.879E-27
Flrt2	1.120232651	46.69340614	2.0258E-47
Creb3l1	1.11924293	27.70141063	1.9888E-28
Tnfsf14	1.118067825	5.894440928	1.2751E-06
2010107G12Rik	1.114523789	2.692531329	0.00202987
Ncaph	1.113213296	42.6745248	2.1158E-43
Prim1	1.112058562	19.36589873	4.3063E-20
Prn	1.111626033	16.37990663	4.1696E-17
Zranb3	1.111474414	10.18825565	6.4825E-11
Skp2	1.11104543	10.57238978	2.6768E-11
Sparc	1.109966016	1.7976931348623157e+308	0
Ugt1a7c	1.108122081	70.21084432	6.154E-71
Sox9	1.103969587	2.861318338	0.0013762
Cdca7	1.10364626	17.91320371	1.2212E-18
Lgmn	1.102463444	254.3135039	4.858E-255
Mthfd2	1.099701799	19.40666305	3.9205E-20
Fbxo48	1.099379163	2.545163302	0.00284995
Fkbp10	1.098824685	84.97568158	1.0576E-85
Uchl1	1.096111806	13.28543286	5.1828E-14
Rfc5	1.094541098	20.09192201	8.0924E-21
Islr	1.093574326	20.49647163	3.1881E-21
Gm1976	1.093005778	4.961744892	1.0921E-05
Mir7678	1.091782381	15.5454903	2.8478E-16
Mcm8	1.091397393	7.481053239	3.3033E-08
Mgp	1.086849825	1.7976931348623157e+308	0
Brca2	1.085656538	15.28316854	5.2099E-16
Sel1l3	1.080190698	6.104860137	7.8549E-07
Gm14005	1.080144819	12.97242183	1.0656E-13
1700071M16Rik	1.0776696	8.413587682	3.8584E-09
C1s1	1.077261914	234.8556246	1.394E-235
Upk3b	1.075491451	106.4542999	3.513E-107
Aplp1	1.07488704	21.57862427	2.6386E-22
Pole2	1.073021258	11.96873891	1.0746E-12
Phex	1.071970306	19.09117331	8.1064E-20
Lrg1	1.071912246	93.89465386	1.2745E-94
6030419C18Rik	1.071728388	8.02712406	9.3945E-09
Il5	1.071616915	2.113064931	0.00770788
Ear7	1.071060248	2.120765967	0.00757241
Lacc1	1.070786135	13.71971864	1.9067E-14
Tnfaip8l1	1.070582471	14.29819604	5.0327E-15
Atad2	1.067008675	70.85216142	1.4055E-71
Reep2	1.06562928	6.654009358	2.2181E-07
Fabp5	1.062760731	95.99760287	1.0055E-96
Cntrob	1.061896393	26.68326309	2.0737E-27
Gm9733	1.061877999	5.434419696	3.6777E-06
Mcm7	1.061028214	68.21058412	6.1577E-69

Syt12	1.06001735	2.589653659	0.00257245
Speg	1.059021334	13.85508369	1.3961E-14
Itm2a	1.058846274	36.69851689	2.0021E-37
Map3k7cl	1.055987559	2.691389899	0.00203521
Adh1	1.05563975	143.1916024	6.433E-144
Efs	1.052966731	9.132876577	7.3642E-10
Clec4a2	1.052443053	19.26943378	5.3773E-20
Batf3	1.051997914	3.732827484	0.000185
Dck	1.049201157	44.45146955	3.5361E-45
Nrep	1.048185972	135.5088751	3.098E-136
Ggct	1.043014649	11.41046655	3.8863E-12
Emid1	1.042911375	42.42603559	3.7494E-43
Efna4	1.042552566	2.446304576	0.00357845
Fmn1	1.039909893	10.08746065	8.176E-11
Gpnmb	1.037057995	86.75326681	1.765E-87
Chd3os	1.035050869	3.893102379	0.00012791
Ska2	1.034852256	18.68028968	2.0879E-19
H2-M2	1.034536259	3.673352221	0.00021215
Stra6l	1.032627382	2.24290919	0.00571598
Ltbp2	1.031794006	149.1888172	6.474E-150
Cspg4	1.030817398	32.04637299	8.9873E-33
Lrrn4	1.030254951	40.98024713	1.0465E-41
Stac2	1.028918038	6.593222113	2.5514E-07
Nfil3	1.02861808	37.95097687	1.1195E-38
Sdf2l1	1.027892579	43.05337486	8.8435E-44
Zfp367	1.026140512	67.84748657	1.4207E-68
Snai1	1.02457078	16.73746011	1.8304E-17
Nid2	1.021003872	44.07978628	8.3217E-45
Hspb7	1.020580116	4.973230144	1.0636E-05
Atad5	1.019802255	16.08967554	8.1344E-17
Ifi205	1.018817323	8.992951926	1.0164E-09
Ugt1a9	1.017932139	44.56665217	2.7124E-45
Ugt1a5	1.017932139	44.56665217	2.7124E-45
Ugt1a10	1.017932139	44.56665217	2.7124E-45
Sbsn	1.017524385	11.88187077	1.3126E-12
Pdlim4	1.017243003	9.006836584	9.8438E-10
Ugt1a2	1.017229389	44.51504713	3.0546E-45
Ugt1a1	1.016173549	44.47199207	3.3729E-45
Ube2s	1.015765072	41.8748773	1.3339E-42
Atp1a3	1.015218871	36.01263575	9.7132E-37
Serf1	1.011633646	4.37679363	4.1996E-05
Entpd2	1.010369452	3.71689442	0.00019191
Sp6	1.009852106	8.412124752	3.8715E-09
Ugt1a6b	1.00822023	44.10297864	7.889E-45
Clec4e	1.007694559	42.95932756	1.0982E-43
Bmp2	1.006599457	11.66824819	2.1466E-12
Zfpm2	1.006245709	12.76655274	1.7118E-13
Hspb6	1.004812157	9.426210052	3.7479E-10
Tmem198b	1.004191947	60.98178166	1.0428E-61

Adamts9	1.000560635	99.25504086	5.559E-100
March4	-1.001207489	2.622145217	0.00238701
Nr3c2	-1.002664646	15.15959507	6.9248E-16
1600029I14Rik	-1.00310756	3.085279112	0.00082171
8430408G22Rik	-1.00379711	83.78501075	1.6405E-84
Cxcr5	-1.004743391	14.96850109	1.0752E-15
Ccdc170	-1.004993488	5.152561231	7.0378E-06
Rgs22	-1.005190463	4.338322881	4.5886E-05
Dhrs3	-1.006232292	144.1406295	7.234E-145
Cbr2	-1.008404097	1.7976931348623157e+308	0
Scgb3a1	-1.009068568	34.4742522	3.3554E-35
Adam28	-1.009752353	5.839247829	1.4479E-06
Ccdc147	-1.010440645	3.620376045	0.00023968
Cd4	-1.010451602	7.63917952	2.2952E-08
Tex11	-1.01140374	2.599909506	0.00251241
Atp7b	-1.012419565	15.84558885	1.427E-16
Gm20554	-1.012495451	2.103618476	0.00787738
Acoxl	-1.014256077	22.18728922	6.497E-23
Lrriq1	-1.014909036	2.889696982	0.00128915
Ttc18	-1.016066786	4.512891519	3.0698E-05
Amigo2	-1.019247789	100.0215704	9.515E-101
Fam183b	-1.019355169	6.190408596	6.4505E-07
Foxa1	-1.021996102	14.26533434	5.4283E-15
Spag6	-1.027165464	7.624370627	2.3748E-08
P2ry10	-1.027766125	32.66900674	2.1429E-33
Cbx7	-1.028402612	68.440355	3.6278E-69
Slc15a2	-1.029470179	25.4669712	3.4122E-26
Ccdc153	-1.031006145	20.14321713	7.1909E-21
Gm867	-1.031206934	7.189132167	6.4695E-08
Pde4c	-1.03280474	2.43708507	0.00365523
Lef1	-1.033205054	10.95629405	1.1059E-11
Ccdc151	-1.033218577	3.139614477	0.00072508
Cdhr3	-1.03368596	18.55732738	2.7712E-19
Errfi1	-1.036565796	206.1570286	6.966E-207
Ccdc40	-1.037293968	10.69382867	2.0238E-11
Inmt	-1.043507378	1.7976931348623157e+308	0
Reg3g	-1.045751794	10.07925334	8.332E-11
Scgb1a1	-1.047110008	1.7976931348623157e+308	0
Dnah9	-1.049029537	11.46376556	3.4374E-12
Ms4a4d	-1.050136016	51.06085263	8.6926E-52
Rsph4a	-1.052006953	12.06299164	8.6498E-13
Vpreb3	-1.053806539	4.746141213	1.7942E-05
Mycbpap	-1.056308398	8.241170706	5.7389E-09
A830018L16Rik	-1.058600888	2.028578115	0.00936315
Stk33	-1.061710015	5.575269771	2.6591E-06
1700007K13Rik	-1.06182412	6.950489712	1.1208E-07
Fam179a	-1.062084417	13.68544034	2.0633E-14
Tsnaxip1	-1.062403189	4.008704337	9.8016E-05
Esr2	-1.064655598	5.4477338	3.5667E-06

Tmem212	-1.064999235	8.702009365	1.9861E-09
Fam216b	-1.066021683	6.666264738	2.1564E-07
Fhad1	-1.069783669	16.32927646	4.6852E-17
Ccdc11	-1.071455127	4.958263532	1.1009E-05
Map3k19	-1.072262874	3.882083307	0.00013119
1700003M02Rik	-1.074206378	8.40718931	3.9157E-09
Olfm2	-1.075323118	9.853461168	1.4013E-10
Treml4	-1.078056931	13.22296919	5.9845E-14
Snhg11	-1.081207298	14.52177505	3.0076E-15
Acaa1b	-1.08267474	18.68798405	2.0512E-19
Cyp4b1	-1.083616839	1.7976931348623157e+308	0
Dixdc1	-1.085294059	17.24192525	5.7289E-18
Cd6	-1.086567323	6.193607163	6.4031E-07
Cd8a	-1.086831126	7.438333902	3.6447E-08
Ear1	-1.090818855	50.07465579	8.4206E-51
Kcnmb2	-1.092413539	4.209826572	6.1684E-05
Gbp10	-1.092552755	4.126781628	7.4682E-05
Cyp2b10	-1.093733397	152.1519836	7.047E-153
Rai2	-1.096284116	25.10883638	7.7833E-26
Fmo1	-1.097992745	1.7976931348623157e+308	0
Plekhb1	-1.099489841	40.30471321	4.9578E-41
Dnah6	-1.101471958	30.77645836	1.6732E-31
Nrn1	-1.102578277	7.88817098	1.2937E-08
Nyx	-1.106673143	2.374986721	0.00421709
Aox3	-1.112070637	61.31162085	4.8795E-62
Gm11346	-1.117783517	2.414381012	0.0038514
Fam71f2	-1.122140872	10.37958338	4.1727E-11
Efcab6	-1.126563651	3.2345962	0.00058264
Cfap221	-1.128875879	2.310725004	0.00488962
9330159F19Rik	-1.131817044	5.741467324	1.8136E-06
Fsip1	-1.138177382	2.15036777	0.00707347
Sntb1	-1.1414338	44.48631346	3.2635E-45
1700001C02Rik	-1.144994661	2.790381476	0.00162039
Lyve1	-1.147011557	1.7976931348623157e+308	0
1700026D08Rik	-1.149550998	5.412954096	3.8641E-06
Ces2b	-1.153599275	6.717224453	1.9177E-07
Agr3	-1.156398646	3.471865103	0.00033739
Ppargc1a	-1.1574623	4.474745874	3.3516E-05
Pla2g1b	-1.160178913	4.228241847	5.9123E-05
Ccr9	-1.162119742	5.454189471	3.5141E-06
Spag16	-1.163327735	9.03980421	9.1242E-10
Lrrc43	-1.163899683	2.84738404	0.00142107
Lrrc6	-1.165620277	3.775104633	0.00016784
Cfap44	-1.166041858	20.6318708	2.3342E-21
E030019B06Rik	-1.166960681	3.476534844	0.00033378
Ttc29	-1.170535202	6.968006189	1.0764E-07
Cd28	-1.171526519	14.93910767	1.1505E-15
1810010H24Rik	-1.175770161	30.79840537	1.5907E-31
Scn4a	-1.178775866	3.383185349	0.00041382

Dnah5	-1.180226514	21.74464156	1.8004E-22
Aldh3a1	-1.191872634	4.663369805	2.1709E-05
Iqca	-1.192586585	7.279489656	5.2542E-08
Atp2c2	-1.192681247	5.952638809	1.1152E-06
Sntn	-1.192691685	6.149714888	7.0841E-07
Itpka	-1.19883368	4.351970949	4.4466E-05
4932418E24Rik	-1.206152526	6.804379827	1.569E-07
Ston2	-1.207996408	99.36750948	4.29E-100
Tcte1	-1.222427959	5.432504001	3.694E-06
Coro6	-1.235094595	18.32922837	4.6857E-19
Faim2	-1.236433718	33.10002827	7.9428E-34
D430036J16Rik	-1.239323701	3.38473961	0.00041234
Edn1	-1.242979649	1.7976931348623157e+308	0
BC048546	-1.243122077	3.260725902	0.00054862
Acer2	-1.244280253	1.7976931348623157e+308	0
Cyp2f2	-1.250930414	1.7976931348623157e+308	0
Siglech	-1.257544055	9.353757169	4.4284E-10
Spata18	-1.257863629	17.69104328	2.0368E-18
Chrm1	-1.263243739	5.140404791	7.2376E-06
Fam47e	-1.266969333	4.275689953	5.3004E-05
Dnah7a	-1.271884766	2.822051303	0.00150643
Glb1l3	-1.274898811	12.87389615	1.3369E-13
Kif27	-1.275298206	10.60642851	2.475E-11
H2-Eb2	-1.276101859	8.269582817	5.3755E-09
Eno4	-1.278598788	9.679276256	2.0928E-10
Mdh1b	-1.27911222	2.857203733	0.0013893
Cacna2d4	-1.282356999	8.885570159	1.3015E-09
Fmo3	-1.285724047	12.87595409	1.3306E-13
Kndc1	-1.286169695	11.93586185	1.1591E-12
Elmod1	-1.296725354	4.644635947	2.2665E-05
Cacna1i	-1.297826724	11.09145641	8.1011E-12
Nek11	-1.303605856	3.47368142	0.00033598
Ldlrad1	-1.330626573	6.421386528	3.7898E-07
Fermt1	-1.332710357	2.638775939	0.00229733
Ccr7	-1.334676056	82.85344151	1.4014E-83
Lrrc26	-1.339822019	3.204507687	0.00062444
Hmgcs2	-1.346386824	24.44608832	3.5802E-25
Iyd	-1.353054748	2.146870912	0.00713065
Arhgef38	-1.356394725	4.341872037	4.5512E-05
Wnt2b	-1.373862909	18.31297716	4.8643E-19
Klf15	-1.382572085	38.74442351	1.8013E-39
Themis	-1.391623203	6.739876959	1.8202E-07
Dapl1	-1.406643927	2.440601674	0.00362575
Colq	-1.460710373	67.84970786	1.4135E-68
Dcdc2a	-1.485543937	6.244428423	5.696E-07
Sult1d1	-1.504200437	5.268393192	5.3902E-06
Pon1	-1.50611313	20.03703961	9.1825E-21
Fgfbp1	-1.508789866	8.581006952	2.6242E-09
Cyp2a5	-1.534913949	30.42992916	3.716E-31

Aqp4	-1.54300468	6.135108876	7.3264E-07
Slc10a5	-1.544059579	2.142467431	0.00720332
Cyp4a32	-1.548471048	8.969707631	1.0722E-09
2410004I01Rik	-1.560451124	6.377239296	4.1953E-07
Itgad	-1.567076998	8.865006193	1.3646E-09
Gm12695	-1.582878657	2.655150172	0.00221233
C330013F16Rik	-1.592491733	3.050887305	0.00088943
Slc6a19	-1.597503649	2.039021117	0.00914069
Gm4759	-1.621052817	4.700552675	1.9927E-05
8430426J06Rik	-1.630547148	6.022115374	9.5035E-07
Gm14085	-1.642983901	10.76239908	1.7282E-11
Mchr1	-1.654107205	3.475283719	0.00033475
Fabp12	-1.655033919	2.963770729	0.001087
Krt15	-1.663685451	3.342606672	0.00045435
Npr3	-1.670134	1.7976931348623157e+308	0
Apol7c	-1.686672895	6.819962639	1.5137E-07
Ttc34	-1.711379269	3.853130517	0.00014024
Igfbp3	-1.716093248	294.4207658	3.795E-295
D130043K22Rik	-1.758583153	4.771276515	1.6933E-05
Slc16a5	-1.784041865	3.970387967	0.00010706
Ptpru	-1.820984469	300.6765438	2.106E-301
Cyp1a1	-1.826398052	72.30260815	4.9819E-73
Hes2	-1.969678383	3.86545607	0.00013632
Bpifa1	-2.120367584	253.9298684	1.175E-254
Esm1	-2.138608348	94.53788345	2.8981E-95
Il22ra2	-2.470165733	5.495674314	3.1939E-06
Krt5	-2.489761234	2.270447957	0.00536478
Rag1	-2.581320857	4.105721775	7.8393E-05
Gm609	-2.741020578	2.227737578	0.00591919
9530026P05Rik	-2.840255181	25.8369802	1.4555E-26
Lrat	-3.088198363	1.7976931348623157e+308	0
H2-Q1	-3.21404529	4.928345268	1.1794E-05
Usp26	-6.081743825	2.908312395	0.00123506
Cyp26b1	-7.354658521	1.7976931348623157e+308	0
