# Supplementary material for: Hydrostatic Pressure Controls Angiogenesis Through Endothelial YAP1 During Lung Regeneration
Source: Front Bioeng Biotechnol. 2022 Feb 18;10:823642. doi: 10.3389/fbioe.2022.823642 (PMC8896883; doi:10.3389/fbioe.2022.823642)
Supplement: Supplementary file 9 [file DataSheet5.PDF]

Biological Processes GO Terms	PValue	Fold Enrichment	Genes
cell adhesion	6.4308E-24	4.60548211	COL18A1, PTPRU, SPON2, ITGAM, TENM3, COL16A1, TNFAIP6, COL14A1, TNC, MSLN, WISP1, HAPLN1, ISLR, CDH3, CDH2, CHL1, CYP1B1, EMILIN2, NRCAM, EPHB1, MYBPC2, APLP1, VCAN, ITGAD, COL6A2, CDHR1, COL6A1, ADAM12, CDHR3, COL6A4, CERCAM, COL6A6, COL6A5, COL15A1, LAMA1, PCDH10, TROAP, AMIGO2, THBS2, NID2, THBS4, EFS, FLRT2, GPNMB, HAS1, SPP1, CLCA2, HAS2, COL28A1, AOC3, CADM3, CADM4, FN1, LYVE1, SELP, MFAP4, FERMT1, SDK1, NFASC, CD4, COL5A1, CD6, TGFBI
cell cycle	1.3838E-22	3.98434566	ERCC6L, MCM7, CCNF, DIXDC1, BUB1B, BRCA1, KIF11, FOXM1, BRCA2, MKI67, CDC20, CHAF1B, CHAF1A, CHEK2, NUF2, CHEK1, RCC1, NEK2, DLGAP5, HELLS, LIG1, KIF23, CDC25C, CIT, CCNA2, PSRC1, CCNE1, INCENP, FANCD2, MCM3, BIRC5, GAS1, MCM5, KIF2C, MCM6, KIF20B, MCM2, CDCA3, UHRF1, PMF1, AURKB, AURKA, CCNB1, DSN1, RACGAP1, E2F1, CLSPN, ECT2, BUB1, MAPK4, E2F7, CDT1, CDKN2C, SPAG5, PLK1, CDC7, NDC80, ANLN, TPX2, CENPE, WEE1, KIF18B, PRC1, UBE2S, CDK1, NCAPD2, SPC24, CDKN3, MAD2L1
cell division	8.76111E-19	4.645160952	ERCC6L, CCNF, KIF14, BUB1B, KIF11, CDC20, CHEK2, NUF2, RCC1, NEK2, HELLS, LIG1, KIFC5B, KIF23, CDC25C, CIT, CCNA2, PSRC1, KIFC1, CCNE1, INCENP, BIRC5, MCM5, KIF2C, KIF20B, CDCA3, PMF1, AURKB, AURKA, CCNB1, DSN1, RACGAP1, ECT2, BUB1, SPAG5, PLK1, CDC7, NDC80, ANLN, TPX2, CENPE, WEE1, KIF18B, PRC1, UBE2S, CDK1, NCAPD2, SPC24, MAD2L1
mitotic nuclear division	7.57426E-17	5.119841438	ERCC6L, CDCA3, CCNF, BUB1B, KIF11, PMF1, AURKB, AURKA, CDC20, CCNB1, DSN1, TUBB3, CHEK2, NUF2, RCC1, NEK2, BUB1, HELLS, SPAG5, PLK1, KIF23, CDC25C, NDC80, CIT, CCNA2, ANLN, CENPE, TPX2, WEE1, KIF18B, PSRC1, ESPL1, INCENP, CDK1, BIRC5, NCAPD2, KIF2C, KIF20B, SPC24, MAD2L1
inflammatory response	6.1505E-13	4.019596443	TPSB2, CALCA, CCL11, HMGB2, CXCL1, MEFV, CXCL13, PLA2G7, CXCL5, PYCARD, C3, C4B, CYP26B1, CCL8, CCL7, SPP1, CCL2, BDKRB1, CCR7, CD14, CCR5, CCR3, IL10, NGFR, CCL24, CD163, TNFRSF9, SLC11A1, NR1H4, CELA1, SELP, CXCL10, IL6, BMP2, IL5, ADAM8, CSPG4, ACKR1, PF4
DNA replication	3.15043E-11	6.34152718	CDT1, PIF1, POLQ, RMI2, RRM1, FEN1, BLM, RRM2, MCM7, LIG1, PRIM1, MCM10, BRCA1, ADRA2A, CHAF1B, CHAF1A, MCM3, MCM5, MCM6, DTL, POLE, MCM2
microtubule-based movement	4.31295E-11	8.181900452	DNAH5, KIFC5B, KIF14, DNAH7A, KIF23, DNAH9, KIF11, KIF22, KIF27, KIF15, KIF4, CENPE, KIF18A, KIF18B, KIFC1, KIF2C, KIF20A, KIF20B
cellular response to tumor necrosis factor	2.24005E-10	6.446345811	CCL24, EDN1, CALCA, CCL11, BRCA1, ADAMTS12, PYCARD, COL1A1, DCSTAMP, IL6, SFRP1, CCL8, FABP4, CCL7, CCL2, HAS2, PCK1, PPARGC1A, DBN1, ADAMTS7
proteolysis	9.10231E-10	2.802277475	CFD, TPSB2, MYRF, MCPT4, CFI, ADAMTS12, CPZ, PHEX, MMP23, ADAMTS4, ADAMTS16, UCHL1, ADAM28, ADAMTS2, C1S1, CAPN6, ADAMTS19, CTSK, CLCA2, CPXM1, TPSAB1, ADAMTS9, ADAMTS7, USP26, F10, CMA1, MMP2, ECEL1, CELA1, PRSS35, MMP8, MMP10, MMP12, CAPN11, MMP14, MMP13, ESPL1, PAPPA, ADAM12, MMP19, PRTN3, ADAM8, TRABD2B, CFB, LGMN, PAPPA2
immune response	3.60952E-09	3.780118224	CCL11, MCPT4, CXCR5, CXCL1, CXCL13, CXCL5, CCL8, CCL7, CCR9, CCL2, CCR7, ENPP3, CCR5, IL10, NGFR, CCL24, TNFSF14, CMA1, TNFRSF9, LIF, PRG4, CXCL10, IL6, CD4, IL5, SLPI, CD28, FCGR2B, PF4
mitotic sister chromatid segregation	5.94907E-09	15.41517477	DSN1, KIF18B, ESPL1, SPAG5, PLK1, NEK2, CENPA, NDC80, CIT, MAD2L1
collagen fibril organization	6.48616E-09	10.9092006	COL1A1, ADAMTS2, COL3A1, COL1A2, SFRP2, COL5A1, COL14A1, LUM, COL11A1, COL5A2, CYP1B1, LOXL2
response to drug	9.07685E-09	3.346775406	COL18A1, OXTR, NNMT, MCM7, HMGB2, ATP1A3, ABAT, GATA4, PTN, TYMS, RAD54B, CCNB1, CDH3, LGALS1, MDK, RAD54L, PPARGC1A, IL10, NTRK1, UGT1A1, ARG1, MMP2, INHBA, COL1A1, ALDH3A1, IL6, SFRP1, SFRP2, RAD51, SCGB1A1, CYP1A1, CDK1
response to lipopolysaccharide	4.04875E-08	4.139404797	NGFR, SPON2, EDN1, SPARC, UGT1A1, TNFRSF9, SLC11A1, PTGER3, HMGB2, CXCL1, CXCL13, LOXL1, CXCL5, DCN, CXCL10, SLPI, CD6, SCGB1A1, CYP1A1, BDKRB1, CCR7, PCK1, PF4
cellular response to interleukin-1	4.51056E-08	6.647794118	CCL24, EDN1, CCL11, FN1, ADAMTS12, PYCARD, IL6, SFRP1, CCL8, CCL7, CCL2, HAS2, SOX9, PCK1, ADAMTS7
osteoblast differentiation	1.10213E-07	5.333923304	IGFBP3, LEF1, TNC, FHL2, CHRD, TWIST1, IGF1, GLI1, COL1A1, VCAN, SFRP1, BMP2, GPNMB, CREB3L1, COL6A1, SNAI1, SPP1
cellular response to DNA damage stimulus	1.19453E-07	2.87015873	TOP2A, PIF1, FEN1, BLM, MCM7, UHRF1, MCM10, BRCA1, FOXM1, BRCA2, UNG, RAD54B, BRIP1, CHAF1B, CHAF1A, EXO1, CHEK2, CHEK1, RAD54L, CLSPN, POLE, E2F7, BARD1, POLQ, EGLN3, LIG1, ATAD5, RAD51B, MMS22L, RAD51C, RAD51, FANCD2, DTL, CDKN3
chromosome segregation	1.81043E-07	5.975545274	TOP2A, SPAG5, KIF11, BRCA1, PMF1, NDC80, CENPE, DSN1, ESPL1, INCENP, BIRC5, RCC1, KIF2C, NEK2, BUB1
collagen catabolic process	1.84502E-07	13.29558824	ADAMTS2, MMP14, MMP13, CTSK, MMP2, MMP19, PRTN3, MMP8, MMP10
chemokine-mediated signaling pathway	3.08138E-07	7.735614973	CXCL10, CCL24, CCL8, CCL11, CCL7, CCR9, CCL2, CXCL1, ACKR1, CXCL13, CXCL5, PF4
wound healing	3.6346E-07	5.657697121	SERPINB2, SPARC, BNC1, SLC11A1, ALOX15, WNT5A, TNC, FN1, DCN, MMP12, COL1A1, IL6, COL3A1, CDH3, TIMP1
regulation of cell proliferation	4.9204E-07	3.592346895	EGLN3, SPARC, TNFRSF9, TNC, CDCA7, PRG4, CELA1, IGF1, BRCA1, CXCL13, FOXM1, BRCA2, CXCL5, ESR2, NR3C2, CXCL10, IL6, SFRP2, CHEK1, SOX9, LGR5, PF4, EZH2
chemotaxis	1.13419E-06	4.807444334	CCL24, CCL11, HMGB2, CXCR5, CXCL13, CXCL5, CXCL10, CCL8, CCL7, CCR9, PROK2, CCL2, CCR7, CCR5, CCR3, PF4
positive regulation of cell proliferation	1.8378E-06	2.420353086	COL18A1, LEF1, TNC, KIF14, PTN, FOXM1, GLI1, CDC20, ESM1, PROK2, HAS2, SOX9, TIMP1, ARNT2, FGFBP1, EDN1, CAV3, WNT5A, LIF, FN1, CDC7, IGF1, RAD51B, CXCL10, ALDH3A1, MLXIPL, IL6, SFRP1, SFRP2, FABP4, IL5, PRC1, ALDH1A2, SP6, GAS1, KIF20B, FOLR2
aging	1.95488E-06	3.893890967	NTRK1, IL10, CALCA, ARG1, SERPINF1, ABAT, TYMS, AURKB, DCN, ALDH3A1, IL6, COL3A1, KRT14, CYP1A1, CCL2, TIMP1, APOE, PCK1, PPARGC1A
positive regulation of transcription, DNA-templated	2.89806E-06	2.339038671	BLM, MYRF, LEF1, HMGB2, GATA4, BRCA1, FOXM1, BRCA2, GLI1, MDK, CHEK2, E2F1, SOX9, PPARGC1A, FGF23, WNT4, IL10, ARNT2, WNT5A, EBF3, IGF1, INHBA, GDF6, KLF15, ETV4, ESR2, CCNA2, COL1A1, MLXIPL, IL6, SFRP1, BMP2, PSRC1, IL5, WT1, SNAI1, ROR2, ZFPM2
DNA replication initiation	3.06834E-06	11.81830065	MCM7, CCNE1, MCM3, CDC7, MCM5, MCM10, MCM6, MCM2
negative regulation of neuron death	3.18323E-06	6.964355742	GABRB3, NTRK1, CHGA, IL6, GPNMB, MDK, SERPINF1, IGF1, APOE, PPARGC1A, ESR2
mitotic metaphase plate congression	3.55421E-06	9.385121107	CENPE, KIF18A, CCNB1, PSRC1, KIFC1, KIFC5B, KIF14, KIF2C, KIF22
extracellular matrix organization	3.8991E-06	4.665118679	VIT, COL18A1, OLFML2B, LAMA1, ELN, APLP1, FN1, NID2, CCDC80, COL5A3, ADAMTSL2, COL6A4, VWA1, SOX9, TGFBI
DNA repair	4.05957E-06	2.898828462	PIF1, FEN1, BLM, UHRF1, BRCA1, FOXM1, BRCA2, UNG, BRIP1, CHAF1B, CHAF1A, EXO1, CHEK2, CHEK1, RAD54L, CLSPN, POLE, BARD1, POLQ, LIG1, KIF22, RAD51B, MMS22L, RAD51C, RAD51, FANCD2
negative regulation of angiogenesis	5.0551E-06	5.909150327	NGFR, CXCL10, SPARC, SERPINF1, HHIP, LIF, CCL2, PTN, THBS2, THBS4, DCN, PF4
cytokinesis	7.01687E-06	8.624165342	PRC1, INCENP, PLK1, BIRC5, KIF23, KIF20A, ECT2, BRCA2, CIT
skeletal system development	9.69794E-06	4.638959135	EDN1, NPR3, CHRD, GDF3, HAPLN1, COL1A1, VCAN, COL3A1, COL1A2, COL5A2, FAM20C, ROR2, SOX9, FBN1
heterophilic cell-cell adhesion via plasma membrane cell adhesion molecules	1.29515E-05	6.818250377	SELP, CADM3, LGALS1, CADM4, TENM3, CDH2, CD6, GM609, AMIGO2, LGALS7
male gonad development	1.60084E-05	4.431862745	RRM1, WNT2B, WNT5A, HMGB2, GATA4, INHBA, INSL6, MMP14, BRIP1, SFRP1, SFRP2, WT1, SOX9, WNT4
positive regulation of endothelial cell proliferation	1.68071E-05	5.820954053	IL10, CCL24, BMP2, CCL11, LRG1, ARG1, WNT5A, HMGB2, CCL2, CCR3, THBS4
cartilage development	1.81809E-05	5.188522238	BMP2, EDN1, MMP13, SFRP2, LUM, COL11A1, MGP, WNT5A, ITGB8, TIMP1, SOX9, TYMS
positive regulation of cytokinesis	3.04033E-05	8.595127748	RACGAP1, CXCR5, KIF14, KIF23, KIF20B, ECT2, AURKB, CIT
G2/M transition of mitotic cell cycle	3.04033E-05	8.595127748	CHEK2, PLK1, CHEK1, BIRC5, CDC25C, SKP2, FOXM1, CIT
mitotic spindle assembly	4.55815E-05	8.103977591	CDC20, TPX2, KIFC1, CHEK2, KIFC5B, BIRC5, NEK2, KIF11
response to activity	6.09426E-05	6.51212485	NTRK1, IL10, EDN1, ADIPOQ, CDK1, CCL2, PTN, PCK1, PPARGC1A
positive regulation of cell migration	6.42873E-05	3.14378441	COL18A1, CCL24, EDN1, CCL11, TNFAIP6, MMP2, LEF1, FN1, IGF1, ADRA2A, COL1A1, CXCL10, MMP14, BMP2, GPNMB, SNAI1, HAS2, ROR2
positive regulation of gene expression	6.89988E-05	2.399203892	LEF1, PTGER3, HMGB2, TNC, TWIST1, GATA4, BRCA1, IQGAP3, CYP26B1, CDH3, E2F1, ITGB8, SOX9, CCR5, OSR1, SLC11A1, LIF, FN1, INHBA, ETV4, SFRP4, IL6, BMP2, ALDH1A2, CD28, CDK1, PF4
cellular response to retinoic acid	8.13919E-05	5.454600302	COL1A1, CYP26A1, CYP26B1, ALDH1A2, SERPINF1, OSR1, TNC, CCL2, SOX9, PCK1
endodermal cell differentiation	8.64059E-05	9.192011619	MMP14, COL11A1, MMP2, COL6A1, FN1, INHBA, MMP8
positive regulation of ERK1 and ERK2 cascade	8.66316E-05	3.206028369	NTRK1, CCL24, NDRG4, CCL11, ALOX15, TREM2, GATA4, ESR2, PYCARD, C3, IL6, BMP2, CCL8, CCL7, GPNMB, CCL2, FGF23
DNA unwinding involved in DNA replication	0.000113857	17.72745098	TOP2A, RAD51, MCM7, MCM6, MCM2
eosinophil chemotaxis	0.000113857	17.72745098	CCL24, CCL11, CCL7, CCL2, CCR3
double-strand break repair via homologous recombination	0.000116455	5.213956171	RAD51B, MMS22L, BLM, RAD51, RAD51C, TONSL, RAD54L, BRCA1, BRCA2, RAD54B
DNA recombination	0.000134988	4.58828143	RAD51B, PIF1, BLM, RAD51, RAD51C, LIG1, EXO1, HMGB2, BRCA1, BRCA2, RAG1
negative regulation of canonical Wnt signaling pathway	0.000139591	4.171164937	SFRP4, SFRP1, BMP2, SFRP2, CDH2, FRZB, WNT5A, LEF1, ROR2, SOX9, GLI1, WNT4
protein heterotrimerization	0.000145285	11.19628483	COL1A1, COL1A2, COL6A2, COL6A1, ADIPOQ, COL6A4
negative regulation of apoptotic process	0.000156153	2.067158595	HHIP, LEF1, AMIGO2, KIF14, FHL2, TWIST1, AURKA, UNG, INSL6, SPP1, PROK2, SOX9, TIMP1, IL10, BARD1, NGFR, ARNT2, HELLS, STIL, SERPINB2, OSR1, PLK1, WNT5A, NR1H4, FN1, IGF1, IL6, SFRP1, WT1, CDK1, BIRC5, GAS1, MAD2L1
positive regulation of angiogenesis	0.000157683	3.809204343	CCL24, CCL11, CMA1, WNT5A, CELA1, GATA4, BRCA1, C3, LRG1, SFRP2, HMOX1, CYP1B1, CCR3
mitotic cytokinesis	0.000161342	8.272810458	ANLN, RACGAP1, PLK1, KIF23, KIF20A, CENPA, KIF4
organ regeneration	0.000161572	5.698109244	CCNA2, PTPRU, NNMT, UGT1A1, CDK1, LIF, CCL2, MKI67, CXCL5
extracellular fibril organization	0.000174942	16.11586453	MFAP5, MFAP4, COL3A1, COL5A1, LTBP2
canonical Wnt signaling pathway	0.000198462	4.382066534	PTPRU, SFRP4, SFRP1, CDH3, WNT2B, WNT5A, LEF1, DIXDC1, GATA4, GLI1, WNT4
lung development	0.00019886	3.717046173	FOXA1, SPARC, ARG1, WNT5A, LIF, IGF1, PTN, GLI1, MMP14, ADAMTS2, ALDH1A2, MGP, ZFPM2
response to wounding	0.000207564	5.501622718	NGFR, IL6, FABP5, MDK, CYP1A1, CCL2, GLI1, GRIN2C, AURKA
response to glucocorticoid	0.000249445	4.727320261	IL10, ALDH3A1, PTPRU, IL6, SPARC, MDK, PAPPA, ADIPOQ, SCGB1A1, TYMS
prostate gland epithelium morphogenesis	0.000256587	14.77287582	FOXA1, MMP2, TNC, IGF1, ESR2
positive regulation of epithelial cell proliferation	0.000276021	4.665118679	IL6, SFRP1, BNC1, OSR1, WNT5A, TWIST1, GAS1, IGF1, SOX9, TBX18
cellular response to estradiol stimulus	0.000280178	7.52073678	IL10, CCNA2, IL6, SFRP1, UGT1A1, MMP2, PPARGC1A
activation of protein kinase activity	0.000280178	7.52073678	TPX2, CALCA, SLC11A1, KIF14, CLSPN, ECT2, ADRA2A
cellular response to transforming growth factor beta stimulus	0.000296237	5.231051109	COL1A1, SFRP1, EDN1, ARG1, WNT5A, SOX9, PPARGC1A, DBN1, WNT4
axon guidance	0.000306884	3.33133307	NTRK1, NGFR, LAMA1, WNT5A, EFNA5, EFNA4, NFASC, FLRT2, TUBB3, CHL1, GAS1, NRCAM, EPHB2, EPHB1
positive regulation of epithelial to mesenchymal transition	0.00033221	7.299538639	COL1A1, CRB2, BMP2, LEF1, SNAI1, TWIST1, EZH2
ossification	0.000369572	4.06254085	MMP14, BMP2, SPARC, COL11A1, COL5A2, MGP, ALOX15, SPP1, TWIST1, SOX9, PTN
response to mechanical stimulus	0.000370939	5.064985994	COL3A1, MMP14, CCNB1, MMP2, TNC, CCL2, BDKRB1, GATA4, DCN
positive regulation of inflammatory response	0.000370939	5.064985994	CCL24, CCL8, CCL11, FABP4, CCL7, WNT5A, CCL2, ADAM8, CCR5
bone mineralization	0.000391512	7.090980392	BMP2, MMP13, GPNMB, ALOX15, PTN, PHEX, ASPN
mitotic spindle midzone assembly	0.000416247	23.63660131	RACGAP1, KIF23, AURKB, KIF4
collagen biosynthetic process	0.000416247	23.63660131	COL1A1, ADAMTS3, COL5A1, ARG1
negative regulation of hormone secretion	0.000496121	12.66246499	IL6, EDN1, ADIPOQ, LIF, FGF23
tissue remodeling	0.000496121	12.66246499	MMP14, MMP2, CSPG4, CELA1, THBS4
positive regulation of canonical Wnt signaling pathway	0.000566361	4.762598771	COL1A1, SFRP4, SFRP1, WNT2B, SFRP2, DIXDC1, ROR2, LGR5, WNT4
negative regulation of cell proliferation	0.000596205	2.215931373	NTRK1, IL10, PTPRU, CDKN2C, IGFBP3, TNFRSF9, LIF, IGF1, INHBA, ESR2, SFRP4, IL6, BRIP1, SFRP1, BMP2, SFRP2, FRZB, WT1, ALDH1A2, HMOX1, CYP1B1, ROR2, SOX9, E2F7
negative regulation of smooth muscle cell proliferation	0.000620044	6.531166151	NDRG4, IGFBP3, NPR3, ADIPOQ, HMOX1, PPARGC1A, ESR2
palate development	0.000638336	4.171164937	GABRB3, OSR1, WNT5A, LEF1, SNAI1, TWIST1, GAS1, INHBA, EPHB2, DHRS3
cellular response to hypoxia	0.000649065	3.78644584	CCNA2, SFRP1, CCNB1, EDN1, E2F1, TWIST1, HMOX1, ADAM8, PTN, PCK1, PPARGC1A
cell-cell signaling	0.000649065	3.78644584	FGFBP1, EDN1, WNT2B, WNT5A, GATA4, CXCL13, CCR5, ADRA1B, WISP1, ADRA2A, WNT4
retinoic acid metabolic process	0.00066155	11.81830065	CYP26A1, ALDH1A3, CYP26B1, ALDH1A2, LRAT
ERK1 and ERK2 cascade	0.00069848	8.181900452	OXTR, CCL11, IGF1, SOX9, IQGAP3, CCR3
regulation of attachment of spindle microtubules to kinetochore	0.000713205	20.25994398	RACGAP1, SPAG5, NEK2, ECT2
positive regulation of synapse assembly	0.000761699	4.558487395	NTRK1, OXTR, FLRT2, TPBG, AMIGO2, THBS2, EPHB2, EFNA5, EPHB1
axon regeneration	0.000862557	11.07965686	DHFR, CHL1, APOE, NREP, FOLR1
mitotic spindle organization	0.00099633	7.597478992	STIL, CCNB1, RCC1, AURKB, NDC80, AURKA
response to ionizing radiation	0.001051517	4.976126591	RAD51B, RRM1, RAD51, RAD51C, KRT14, RAD54L, BRCA1, RAD54B
positive regulation of cell-substrate adhesion	0.001071504	5.909150327	VIT, CCDC80, ALOX15, SPP1, PTN, FBLN2, DBN1
cellular response to interleukin-6	0.001103034	10.42791234	SELP, ENTPD2, CCL2, PPARGC1A, FGF23
response to hypoxia	0.001110108	2.769914216	ARNT2, EDN1, EGLN3, MMP2, ADIPOQ, ABAT, CYGB, LOXL2, ALDH3A1, MMP14, BMP2, CYP1A1, TACC3, HMOX1, CCL2
maternal process involved in parturition	0.001117308	17.72745098	EDN1, OXTR, CYP1A1, CCL2
liver regeneration	0.001176358	7.335496957	IL10, HMOX1, GLI1, TYMS, EZH2, AURKA
angiogenesis	0.001257811	2.521896792	COL18A1, MMP2, FN1, ESM1, MMP14, CCDC80, PROK2, HMOX1, CCL2, MMP19, CYP1B1, ADAM8, NRCAM, CSPG4, TGFBI, EPHB2, EPHB1
positive regulation of tumor necrosis factor production	0.001292958	4.807444334	PYCARD, SPON2, TWIST1, CCL2, CCR7, CD14, CCR5, PF4
glucose homeostasis	0.001357093	3.198938523	FOXA1, MLXIPL, NGFR, IL6, CAV3, PMCH, ADIPOQ, NR1H4, PCK1, ADRA1B, ADRA2A, FBN1
mitotic spindle assembly checkpoint	0.00138688	9.848583878	CENPE, PLK1, BUB1B, BUB1, MAD2L1
trophoblast giant cell differentiation	0.00138688	9.848583878	PLK4, GJB5, SNAI1, LIF, E2F7
cell proliferation	0.001471897	2.578538324	MCM7, UHRF1, MCM10, IGF1, MKI67, BRCA2, AURKB, UCHL1, CYP1A1, CDK1, TACC3, PROK2, CSPG4, TGFBI, BUB1, CFB
cell chemotaxis	0.001555992	4.090950226	CXCL10, CCL8, LEF1, HMGB2, CCL2, CXCL1, CXCL13, EPHB1, CXCL5
epithelial to mesenchymal transition	0.001607056	6.862239089	BMP2, LEF1, SNAI1, SOX9, LOXL2, WNT4
bone trabecula formation	0.001641027	15.7577342	FBN2, COL1A1, SFRP1, MMP2
estrous cycle	0.001717972	9.330237358	OXTR, HAS1, HAS2, PTN, ESR2
response to vitamin A	0.001717972	9.330237358	ARG1, ALDH1A2, CYP1A1, GATA4, TYMS
G1/S transition of mitotic cell cycle	0.001734017	4.574826059	CDKN2C, CCNE1, RCC1, INHBA, SKP2, IQGAP3, POLE, CDKN3
bone morphogenesis	0.001861185	6.647794118	CYP26B1, MMP13, SFRP2, HAS2, DHRS3, PAPPA2
negative regulation of neuron apoptotic process	0.001939023	2.880710784	GABRB3, NTRK1, NGFR, KIF14, CHL1, MDK, HMOX1, CCL2, BIRC5, ADAM8, APOE, PPARGC1A, LGMN
lymphocyte chemotaxis	0.002143507	6.446345811	CCL24, CCL8, CCL11, CCL7, CCL2, ADAM8
positive regulation of peptidyl-tyrosine phosphorylation	0.00218186	3.510386333	IL6, CD4, IL5, ADIPOQ, LIF, TREM2, CSPG4, IGF1, EFNA5, THBS4
complement activation, alternative pathway	0.002295529	14.18196078	CFD, C3, CFP, CFB
protein localization to kinetochore	0.002295529	14.18196078	CDK1, BUB1B, TTK, AURKB
cellular response to vitamin D	0.002295529	14.18196078	SFRP1, TNC, PTN, FGF23
development of primary male sexual characteristics	0.002328295	35.45490196	SFRP1, SFRP2, WNT5A
response to gamma radiation	0.0024558	6.256747405	CXCL10, CCL7, FANCD2, CHEK2, CCL2, BRCA2
cellular response to fatty acid	0.002537203	8.441643324	CCNB1, EDN1, E2F1, UCP1, NR1H4
G2 DNA damage checkpoint	0.002537203	8.441643324	PLK1, CHEK1, CLSPN, BRCA1, DTL
Wnt signaling pathway	0.002926165	2.496824082	WNT2B, LEF1, WNT5A, DIXDC1, CELA1, CPZ, WISP1, SFRP4, SFRP1, SFRP2, FRZB, CCNE1, ROR2, TRABD2B, WNT4
immune system process	0.002936625	2.036574003	C1QB, CFD, C1QA, SPON2, CFI, HMGB2, NR1H4, HMGB3, CFP, MEFV, PYCARD, C3, DCSTAMP, CD4, BPIFA1, C1S1, SLPI, CD8A, EXO1, PADI4, CD14, CFB
cellular response to interferon-gamma	0.002959251	4.171164937	CCL24, EDN1, CCL8, CCL11, CCL7, WNT5A, CCL2, AQP4

heart development	0.003076096	2.309323116	EDN1, OXTR, SPARC, DNAH5, MMP2, OSR1, GATA4, PTN, VCAN, COL3A1, BMP2, MMP13, WT1, ALDH1A2, SOX9, ZFPM2, FBN1
negative regulation of inflammatory response	0.003114712	3.667748479	IL10, TNFAIP6, SERPINF1, ADIPOQ, PBK, NR1H4, APOE, MEFV, CD276
positive regulation of apoptotic process	0.003155262	2.116710565	TOP2A, BARD1, NGFR, IGFBP3, MMP2, PTN, ESR2, PYCARD, ALDH1A3, SFRP4, IL6, SFRP1, BMP2, SFRP2, FRZB, WT1, ALDH1A2, HMOX1, CYP1B1, ECT2
neutrophil chemotaxis	0.003215303	4.110713271	CCL24, CCL8, CCL11, ITGAM, CCL7, SPP1, CCL2, CXCL1
positive regulation of bone mineralization	0.003590369	5.749443561	FBN2, BMP2, OSR1, FAM20C, CD276, WNT4
cellular response to platelet-derived growth factor stimulus	0.003590688	7.707587383	CCNA2, HAS1, CCL2, HAS2, PTN
cellular response to glucose stimulus	0.003777796	3.994918531	MLXIPL, LGALS1, CMA1, SERPINF1, AQP4, GATA4, IGF1, PPARGC1A
metaphase plate congression	0.004035469	11.81830065	CENPE, KIF2C, KIF22, NDC80
inner ear morphogenesis	0.004085599	3.939433551	ALDH1A3, MAFB, PTPRQ, FRZB, COL11A1, WNT5A, ROR2, EPHB2
retinol metabolic process	0.004214274	7.386437908	ALDH1A3, ALDH1A2, CYP1B1, LRAT, DHRS3
regulation of mitotic centrosome separation	0.00456974	26.59117647	CHEK1, NEK2, KIF11
cell differentiation	0.004655273	1.63638009	PTPRU, TENM3, MYRF, PTPRQ, TWIST1, EFNA5, GLI1, CLGN, CDC20, RACGAP1, MDK, FRZB, CHL1, NHS, SOX9, ECT2, FGF23, WNT4, NTRK1, NGFR, OSR1, WNT5A, DLK1, TBX18, CIT, SFRP4, DCSTAMP, SFRP1, BMP2, SFRP2, MGP, MMP19, CSPG4, ROR2, TRIP13, DBN1
somitogenesis	0.004765575	4.431862745	CRB2, SFRP1, SFRP2, WNT5A, LEF1, ROR2, TBX18
monocyte chemotaxis	0.005058973	5.318235294	CCL24, CALCA, CCL8, CCL11, CCL7, CCL2
regulation of cell-cell adhesion	0.005137389	10.9092006	LEF1, ADAM8, EFNA5, WNT4
leukocyte migration involved in inflammatory response	0.005137389	10.9092006	AOC3, ITGAM, CCL2, ADAM8
skin development	0.005668119	4.279039892	COL1A1, NGFR, ADAMTS2, COL3A1, COL5A1, GJB3, COL5A2
positive regulation of JAK-STAT cascade	0.005672201	6.818250377	IL10, IL6, IL5, CYP1B1, CXCL5
positive regulation of neuron projection development	0.005708321	2.826115374	NTRK1, NGFR, IL6, NDRG4, TENM3, SERPINF1, WNT5A, LIF, PTN, DBN1, CXCL5
cell-matrix adhesion	0.006347977	3.636400201	COL3A1, COL5A3, FN1, ITGB8, ADAM8, ADAMTS12, MSLN, NID2
microtubule depolymerization	0.006403189	10.12997199	KIF18A, KIF18B, KIF14, KIF2C
positive regulation of protein import into nucleus, translocation	0.006403189	10.12997199	IL6, CDK1, IGF1, BRCA1
cellular response to follicle-stimulating hormone stimulus	0.006403189	10.12997199	GATA4, INHBA, EFNA5, PPARGC1A
positive regulation of urine volume	0.006403189	10.12997199	EDN1, NPR3, PTGER3, HAS2
microtubule bundle formation	0.006513003	6.565722585	PSRC1, CAPN6, PRC1, PLK1, KIF20A
homophilic cell adhesion via plasma membrane adhesion molecules	0.006519242	2.610176831	SDK1, CADM3, CDH3, CADM4, TENM3, CDH2, GM609, CHL1, PCDH10, CDHR1, AMIGO2, CDHR3
protein phosphorylation	0.007026157	1.723502179	LAMA1, BUB1B, TTK, AURKB, AURKA, CCNB1, CHEK2, CHEK1, PBK, NEK2, EPHB2, BUB1, EPHB1, MAPK4, PLK4, NTRK1, IGFBP3, PLK1, WNT5A, CDC7, CIT, WEE1, BMP2, CCNE1, CDK1, BIRC5, FAM20C, ROR2
metaphase/anaphase transition of mitotic cell cycle	0.007474518	21.27294118	TACC3, BUB1B, CIT
mast cell chemotaxis	0.007474518	21.27294118	CHGA, CCL11, CCR3
eye morphogenesis	0.007474518	21.27294118	COL5A1, COL5A2, GAS1
regulation of vascular endothelial growth factor production	0.007474518	21.27294118	IL6, CCL2, AQP4
osteoclast fusion	0.007474518	21.27294118	DCSTAMP, CD109, ADAM8
complement activation	0.007838599	9.454640523	C3, C4B, C1S1, CFB
positive regulation of leukocyte chemotaxis	0.007838599	9.454640523	CXCL10, CXCL13, CXCL5, PF4
positive regulation of T cell chemotaxis	0.007838599	9.454640523	TNFSF14, WNT5A, CCR7, CXCL13
hematopoietic stem cell proliferation	0.007838599	9.454640523	WNT2B, SFRP2, WNT5A, PRG4
positive regulation of GTPase activity	0.008389593	2.671259737	NTRK1, CCL24, CCL8, CCL11, CCL7, WNT5A, CCL2, CCR7, ECT2, WNT4, EZH2
double-strand break repair	0.008462655	3.939433551	POLQ, LIG1, CHEK2, RAD54L, TRIP13, BRCA1, BRCA2
negative regulation of cell growth	0.008873317	2.836392157	DCSTAMP, SFRP1, CDKN2C, PSRC1, SFRP2, FRZB, WT1, GAS1, BDKRB1, INHBA
positive regulation of T cell proliferation	0.009122157	3.877879902	IL6, CD4, CD6, CD28, CCR7, PDCD1LG2, CD276
positive regulation of cytosolic calcium ion concentration	0.009190679	2.635161632	EDN1, OXTR, CAV3, PTGER3, PMCH, CXCL1, CXCL13, CCR5, ADRA1B, MCHR1, CACNA1G
spindle organization	0.009448455	8.86372549	SPAG5, KIF11, AURKB, AURKA
cell proliferation in forebrain	0.009448455	8.86372549	RRM1, FABP7, KIF14, DIXDC1
negative regulation of smooth muscle cell migration	0.009448455	8.86372549	NDRG4, IGFBP3, ADIPOQ, PPARGC1A
innate immune response	0.009811849	1.861382353	C1QB, CFD, C1QA, SPON2, CFI, HMGB2, NR1H4, HMGB3, TREM2, CFP, MEFV, PYCARD, C3, C4B, BPIFA1, C1S1, SLPI, PTX3, PADI4, CD14, CFB
positive regulation of fibroblast proliferation	0.009817577	3.818220211	CCNA2, NGFR, CCNB1, WNT5A, E2F1, FN1, IGF1
negative regulation of cell migration	0.010148713	3.010321865	PTPRU, SFRP1, SFRP2, ADIPOQ, CHRD, CYP1B1, PTN, CCR5, WNT4
positive regulation of phagocytosis	0.010963783	4.431862745	PYCARD, C3, SLC11A1, CCR7, PTX3, FCGR2B
response to hormone	0.010963783	4.431862745	MMP14, FHL2, MMP19, MSI1, TIMP1, ADRA1B
intramembranous ossification	0.011003654	17.72745098	COL1A1, CTSK, MMP2
regulation of branching involved in mammary gland duct morphogenesis	0.011003654	17.72745098	CAV3, WNT5A, ETV4
negative regulation of centrosome duplication	0.011003654	17.72745098	KIFC1, CCNF, KIFC5B
non-canonical Wnt signaling pathway	0.011236759	8.342329873	SFRP4, FRZB, WNT5A, WNT4
neural crest cell development	0.011236759	8.342329873	CYP26A1, EDN1, ALDH1A2, SOX9
single organismal cell-cell adhesion	0.01128895	2.954575163	SELP, PTPRU, CDH3, ITGAM, CCDC80, CDH2, NRCAM, ADAM8, SOX9
positive regulation of fat cell differentiation	0.011934617	4.341416567	TPH1, SFRP1, BMP2, SFRP2, FRZB, IGF1
positive regulation of mitotic cell cycle	0.01195212	5.539828431	FOXA1, CCNB1, CDK1, BIRC5, BRCA2
response to axon injury	0.01195212	5.539828431	NTRK1, LGALS1, ARG1, CDK1, FOLR1
positive regulation of osteoblast differentiation	0.01212928	3.649769319	FBN2, BMP2, SFRP2, FAM20C, IGF1, CD276, WNT4
central nervous system development	0.012741956	3.18695748	NGFR, ARNT2, CYP26A1, VCAN, CHRD, NRCAM, SOX9, HAPLN1
brown fat cell differentiation	0.014751597	5.213956171	MRAP, LRG1, FABP4, ADIPOQ, UCP1
T cell activation	0.014751597	5.213956171	IL6, CD4, CD8A, CD28, CD276
stem cell differentiation	0.014751597	5.213956171	OSR1, LIF, A2M, ETV4, PHF19
positive regulation of ubiquitin protein ligase activity	0.015119833	15.19495798	CDC20, UBE2S, PLK1
strand invasion	0.015119833	15.19495798	RAD51B, RAD51, RAD51C
positive regulation of male gonad development	0.015119833	15.19495798	WT1, SOX9, ZFPM2
copper ion import	0.015119833	15.19495798	ATP7B, STEAP4, STEAP1
positive regulation of transcription from RNA polymerase II promoter	0.015272794	1.460955759	FOXA1, TOP2A, LEF1, HMGB2, TWIST1, GATA4, FHL5, BRCA1, FOXM1, GLI1, CREB3L1, E2F1, SOX9, PPARGC1A, E2F7, IL10, ARNT2, EDN1, LUM, OSR1, SLC11A1, WNT5A, NR1H4, LIF, CELA1, EBF3, IGF1, INHBA, KLF15, ETV4, ESR2, DCN, MLXIPL, IL6, BMP2, MAFB, SFRP2, WT1, CD28, ZFPM2, PF4
positive regulation of protein phosphorylation	0.015303156	2.312276215	NTRK1, C3, BMP2, GPNMB, CD6, CHEK2, PTGER3, WNT5A, ADIPOQ, GATA4, SOX9, IQGAP3
seminiferous tubule development	0.015360879	7.464189886	BRIP1, KIF18A, WT1, GATA4
negative regulation of cAMP biosynthetic process	0.015360879	7.464189886	EDN1, PTGER3, APLP1, ADRA2A
negative regulation of epithelial cell proliferation	0.015773322	3.447004357	SFRP1, SFRP2, WNT5A, GAS1, SOX9, PTN, ESR2
cell fate commitment	0.015773322	3.447004357	BMP2, WNT2B, WNT5A, GAS1, ROR2, SOX9, WNT4
integrin-mediated signaling pathway	0.015934202	3.04988404	ADAM28, COL3A1, ADAMTS3, ITGAM, ITGAD, ADAMTS19, ITGB8, ADAM8
negative regulation of blood pressure	0.016293758	5.064985994	CALCA, PMCH, ADIPOQ, BDKRB1, ABAT
response to ethanol	0.017614533	2.727300151	NTRK1, SPARC, ADIPOQ, TNC, CDK1, CCL2, ABAT, CD14, TYMS
positive regulation of cell-cell adhesion	0.017701007	7.090980392	WNT5A, CCL2, CCR5, TBX18
DNA metabolic process	0.017701007	7.090980392	TOP2A, RAD51, TK1, MKI67
retinal ganglion cell axon guidance	0.017701007	7.090980392	NRCAM, EPHB2, EFNA5, EPHB1
regulation of mitotic nuclear division	0.017701007	7.090980392	CENPE, RCC1, MKI67, KIF20B
negative regulation of gene expression	0.018372608	2.006881243	SERPINF1, HMGB2, GATA4, IGF1, IQGAP3, BRIP1, SFRP1, BMP2, CCNB1, SFRP2, CD28, ITGB8, SOX9, EZH2, WNT4
complement activation, classical pathway	0.01899806	3.867807487	C1QB, C3, C4B, C1QA, C1S1, CFI
positive regulation of mesenchymal cell proliferation	0.019671834	4.791202968	WNT5A, CHRD, GAS1, SOX9, TBX18
pyrimidine nucleotide metabolic process	0.019787342	13.29558824	DCTD, NME4, DCK
cerebellar cortex development	0.019787342	13.29558824	KIF14, DIXDC1, EZH2
positive regulation of acute inflammatory response	0.019787342	13.29558824	AOC3, IL6, ADAM8
negative regulation of retinoic acid receptor signaling pathway	0.019787342	13.29558824	CYP26B1, DHRS3, EZH2
attachment of mitotic spindle microtubules to kinetochore	0.019787342	13.29558824	CENPE, KIF2C, NDC80
regulation of mitotic spindle organization	0.019787342	13.29558824	TPX2, PSRC1, TACC3
tube morphogenesis	0.019787342	13.29558824	FOXA1, GATA4, WNT4
negative regulation of ossification	0.020228307	6.753314659	CALCA, TPH1, SFRP1, SOX9
cellular response to hydrogen peroxide	0.021854063	3.732094943	IL6, ARG1, CDK1, CYP1B1, ECT2, EZH2
response to heat	0.021854063	3.732094943	IL6, CALCA, HSPB7, CCL2, IGF1, CD14
positive regulation of interleukin-1 beta secretion	0.022943381	6.446345811	PYCARD, WNT5A, CCR7, CCR5
extracellular matrix disassembly	0.022943381	6.446345811	MMP12, MMP13, LAMA1, MMP19
positive regulation of leukocyte migration	0.022943381	6.446345811	SELP, AOC3, CCL2, BDKRB1
embryo development	0.023853795	3.141573591	BMP2, RACGAP1, WNT5A, BIRC5, LRAT, BUB1, DLK1
response to estradiol	0.023946273	2.808309066	COL1A1, FOXA1, ARNT2, CD4, OXTR, ALDH1A2, PTN, EZH2
protein K6-linked ubiquitination	0.024972003	11.81830065	BARD1, UBE2S, BRCA1
xenobiotic glucuronidation	0.024972003	11.81830065	UGT1A10, UGT1A1, UGT1A9
attachment of spindle microtubules to kinetochore	0.024972003	11.81830065	CENPE, NUF2, NDC80
positive regulation of cAMP metabolic process	0.024972003	11.81830065	CHGA, CXCL10, PF4
regulation of chromosome segregation	0.024972003	11.81830065	KIF2C, MKI67, BUB1
positive regulation of smooth muscle cell proliferation	0.025197833	3.102303922	IL6, EDN1, HMOX1, IGF1, SKP2, PPARGC1A, WISP1
cochlea morphogenesis	0.025846285	6.166069906	FRZB, WNT5A, SOX9, TBX18
positive regulation of cardiac muscle cell proliferation	0.025846285	6.166069906	CCNB1, CDK1, GATA4, ZFPM2
response to cytokine	0.026591415	3.064003873	COL3A1, OXTR, SPARC, ALDH1A2, SCGB1A1, TIMP1, TYMS
positive regulation of MAPK cascade	0.027558386	2.727300151	NGFR, IL6, BMP2, CDH2, IGFBP3, LIF, IGF1, ADRA1B
epithelial tube branching involved in lung morphogenesis	0.028936572	5.909150327	FOXA1, LAMA1, HHIP, SOX9
positive regulation of vascular endothelial growth factor production	0.028936572	5.909150327	C3, CYP1B1, GATA4, BRCA1
replication fork processing	0.028936572	5.909150327	MMS22L, BLM, RAD51, TONSL
positive regulation of synaptic transmission, glutamatergic	0.028936572	5.909150327	NTRK1, NGFR, OXTR, ROR2
regulation of blood pressure	0.030169829	3.431119545	AOC3, CALCA, EDN1, COL1A2, NPR3, HMOX1
nucleotide biosynthetic process	0.030641122	10.63647059	DHFR, DCTD, TYMS
positive regulation of keratinocyte proliferation	0.030641122	10.63647059	TGM1, CDH3, HAS2
positive regulation of sequence-specific DNA binding transcription factor activity	0.031523715	2.650833791	PYCARD, IL10, FOXA1, IL6, EDN1, IL5, PPARGC1A, ESR2
embryonic limb morphogenesis	0.032041137	3.37665733	FBN2, CYP26B1, ALDH1A2, WNT5A, LEF1, TWIST1
negative regulation of extrinsic apoptotic signaling pathway via death domain receptors	0.032213331	5.672784314	SFRP2, HMGB2, HMOX1, BRCA1
peptidyl-threonine phosphorylation	0.032252334	4.122663019	OSR1, CHEK1, PBK, CDK1, TTK
negative regulation of tumor necrosis factor production	0.032252334	4.122663019	IL10, GPNMB, ADIPOQ, NR1H4, TWIST1
positive regulation of DNA replication	0.032252334	4.122663019	IL6, PLA2G1B, CDK1, IGF1, GLI1
circadian rhythm	0.032926125	2.626289034	NTRK1, NGFR, TPH1, ADIPOQ, PROK2, IGF1, TYMS, PPARGC1A
mitophagy in response to mitochondrial depolarization	0.033235974	2.417379679	SFRP4, FGFBP1, CALCA, CHAF1B, CALCB, SNTG1, KRT15, HAPLN1, ADAMTS7
response to toxic substance	0.034323676	2.885864113	ARNT2, RAD51, PON1, CYP1A1, CDK1, CYP1B1, TYMS
cellular response to lipopolysaccharide	0.034873163	2.035688151	PYCARD, IL10, SPON2, CXCL10, IL6, ARG1, WNT5A, NR1H4, HMGB2, CCL2, CD14, PPARGC1A
response to steroid hormone	0.035675217	5.454600302	OXTR, HMGB2, SPP1, ADRA1B
ventricular cardiac muscle cell development	0.036763427	9.669518717	CCNB1, CDK1, FHL2

telomere maintenance via recombination	0.036763427	9.669518717	RAD51, RAD51C, BRCA2
glial cell migration	0.036763427	9.669518717	FN1, CCL2, CSPG4
positive regulation of insulin-like growth factor receptor signaling pathway	0.036763427	9.669518717	CDH3, IGFBP3, IGF1
multicellular organism development	0.036992159	1.378227481	FOXA1, COL18A1, WNT2B, CHRD, D130043K22RIK, DIXDC1, TWIST1, EFNA5, GLI1, CLGN, FLRT2, RACGAP1, MDK, FRZB, CHL1, CREB3L1, EPHB2, WNT4, NTRK1, NGFR, HELLS, STIL, TNFRSF9, WNT5A, EBF3, GDF6, TBX18, CIT, CENPE, SFRP4, SFRP1, BMP2, SFRP2, FANCD2, MGP, SNAI1, MMP19, CSPG4, ROR2, DBN1
response to nicotine	0.037285318	3.939433551	DHFR, NTRK1, EDN1, HMOX1, ABAT
protein complex assembly	0.038083091	3.223172906	CCNB1, MGP, CDK1, SOX9, PF4, CLGN
embryonic digit morphogenesis	0.038083091	3.223172906	SFRP2, OSR1, WNT5A, TWIST1, GAS1, ROR2
inner ear development	0.038083091	3.223172906	C1QB, BMP2, SPARC, LIN7A, IGF1, LGR5
negative regulation of interferon-gamma production	0.039320494	5.252578068	IL10, SCGB1A1, NR1H4, PDCD1LG2
odontogenesis	0.039320494	5.252578068	OSR1, TWIST1, GAS1, INHBA
cellular response to interleukin-4	0.039320494	5.252578068	DCSTAMP, ARG1, LEF1, MCM2
chondrocyte differentiation	0.039961387	3.853793691	BMP2, WNT2B, OSR1, TGFBI, SOX9
negative regulation of BMP signaling pathway	0.039961387	3.853793691	SFRP1, SFRP2, WNT5A, CHRD, GDF3
negative regulation of cysteine-type endopeptidase activity involved in apoptotic process	0.040241392	3.175065847	IL6, SFRP2, LEF1, BIRC5, IGF1, RAG1
spermatogenesis	0.04025556	1.655142843	HMGB2, GATA4, GLI1, CDC25C, BRCA2, CIT, CLGN, INSL6, CYP26B1, BRIP1, ADAMTS2, CCNB1, RAD51C, RACGAP1, WT1, E2F1, PROK2, SOX9, TRIP13
reciprocal meiotic recombination	0.043147059	5.064985994	RAD51B, RAD51, RAD51C, TRIP13
positive regulation of neutrophil chemotaxis	0.043147059	5.064985994	EDN1, CCR7, CXCL1, THBS4
negative regulation of cytokine production	0.043309019	8.86372549	IL10, GPNMB, SLC11A1
positive regulation of cell growth involved in cardiac muscle cell development	0.043309019	8.86372549	EDN1, IGF1, WISP1
mesonephros development	0.043309019	8.86372549	WT1, OSR1, WNT4
DNA damage response, signal transduction by p53 class mediator resulting in transcription of p21 class mediator	0.043309019	8.86372549	CHEK2, FOXM1, BRCA2
branching involved in ureteric bud morphogenesis	0.045633114	3.693218954	ADAMTS16, BMP2, WT1, SOX9, WNT4
negative regulation of fat cell differentiation	0.045633114	3.693218954	WNT5A, ADIPOQ, E2F1, ZFPM2, DLK1
sprouting angiogenesis	0.047152479	4.890331305	ESM1, LEF1, E2F7, LOXL2
negative regulation of DNA binding	0.047152479	4.890331305	LEF1, E2F1, HMOX1, NEK2
biomineral tissue development	0.047152479	4.890331305	SPP1, FAM20C, PHEX, ASPN
positive regulation of actin filament polymerization	0.048628536	3.617847139	PYCARD, CCL24, CCL11, ALOX15, FMN1
dorsal/ventral pattern formation	0.048628536	3.617847139	EDN1, HHIP, CHRD, GAS1, GLI1
cell development	0.048628536	3.617847139	LEF1, GATA4, IGF1, GDF3, INHBA
somatic hypermutation of immunoglobulin genes	0.050249315	8.181900452	POLQ, EXO1, UNG
retinal metabolic process	0.050249315	8.181900452	ALDH1A3, ALDH1A2, CYP1B1
negative regulation of fibroblast growth factor receptor signaling pathway	0.050249315	8.181900452	NGFR, WNT5A, WNT4
positive regulation of macrophage differentiation	0.050249315	8.181900452	LIF, ROR2, PF4
fatty acid homeostasis	0.050249315	8.181900452	MLXIPL, NR1H4, APOE
notochord development	0.050249315	8.181900452	STIL, GDF3, SOX9
planar cell polarity pathway involved in neural tube closure	0.050249315	8.181900452	SFRP1, SFRP2, WNT5A
response to molecule of bacterial origin	0.050249315	8.181900452	IL10, CXCL1, CD14
positive regulation of smoothened signaling pathway	0.051334014	4.727320261	FOXA1, SFRP1, GAS1, GLI1
DNA damage checkpoint	0.051334014	4.727320261	CHEK2, CHEK1, E2F1, CLSPN
response to progesterone	0.051334014	4.727320261	OXTR, CCL2, PTN, TYMS
negative regulation of protein kinase activity	0.051602521	2.61246646	IL6, FABP4, FLRT2, CAV3, NYX, ASPN, DCN
response to nutrient	0.054728773	2.914101531	ALDH3A1, SLC6A19, GATM, SFRP2, UGT1A1, ADIPOQ
neural tube development	0.054936938	3.47597078	SFRP1, STIL, SFRP2, ALDH1A2, WNT5A
dUMP biosynthetic process	0.055508198	35.45490196	DCTD, DUT
regulation of midbrain dopaminergic neuron differentiation	0.055508198	35.45490196	SFRP1, SFRP2
negative regulation of interleukin-13 production	0.055508198	35.45490196	LEF1, SCGB1A1
sequestering of TGFbeta in extracellular matrix	0.055508198	35.45490196	FBN2, FBN1
actomyosin contractile ring assembly	0.055508198	35.45490196	RACGAP1, KIF23
posterior mesonephric tubule development	0.055508198	35.45490196	WT1, OSR1
positive regulation of antigen processing and presentation of peptide antigen via MHC class II	0.055508198	35.45490196	PYCARD, TREM2
mitotic recombination-dependent replication fork processing	0.055508198	35.45490196	RAD51, BRCA2
flavone metabolic process	0.055508198	35.45490196	UGT1A1, PPARGC1A
positive regulation of eosinophil migration	0.055508198	35.45490196	CCL24, ADAM8
minus-end-directed vesicle transport along microtubule	0.055508198	35.45490196	KIFC1, KIFC5B
ureter urothelium development	0.055508198	35.45490196	OSR1, SOX9
negative regulation of endodermal cell differentiation	0.055508198	35.45490196	COL5A1, COL5A2
lymphocyte chemotaxis across high endothelial venule	0.055508198	35.45490196	CCR7, CXCL13
response to antibiotic	0.055688649	4.574826059	AOC3, IL6, CYP1A1, CCL2
lipopolysaccharide-mediated signaling pathway	0.055688649	4.574826059	CD6, CCL2, TREM2, CD14
cell activation	0.057557002	7.597478992	FN1, IGF1, TIMP1
negative regulation of androgen receptor signaling pathway	0.057557002	7.597478992	SFRP1, IGF1, ESR2
vasculogenesis involved in coronary vascular morphogenesis	0.057557002	7.597478992	PROK2, GATA4, ZFPM2
cell recognition	0.057557002	7.597478992	CADM3, CADM4, GM609
negative regulation of phagocytosis	0.057557002	7.597478992	ADIPOQ, PRTN3, FCGR2B
somite development	0.057557002	7.597478992	RAD51B, FRZB, WNT5A
positive regulation of monocyte chemotaxis	0.057557002	7.597478992	CXCL10, CCL2, PLA2G7
response to dietary excess	0.057557002	7.597478992	GDF3, APOE, PPARGC1A
positive regulation of interleukin-6 production	0.058248924	3.409125189	PYCARD, SPON2, IL6, WNT5A, CCR5
cellular response to organic cyclic compound	0.060146798	2.836392157	MSR1, BMP2, CCNB1, LGALS1, CYP1A1, CYP1B1
neuropeptide signaling pathway	0.062966139	2.799071207	CALCA, PMCH, PNOC, ECEL1, PROK2, MCHR1
activation of MAPK activity	0.062966139	2.799071207	BMP2, WNT5A, PROK2, CSPG4, IGF1, IQGAP3
phosphorylation	0.064454637	1.448321159	BUB1B, TTK, AURKB, AURKA, CHEK2, CHEK1, PBK, NEK2, TK1, EPHB2, BUB1, EPHB1, MAPK4, PLK4, NTRK1, PLK1, CDC7, NME4, DCK, CIT, WEE1, ITPKA, CDK1, FAM20C, ROR2
regulation of neuron apoptotic process	0.064903926	4.297563874	GABRB3, EGLN3, KIF14, ESR2
skeletal system morphogenesis	0.0651851	3.282861293	COL1A1, HHIP, COL11A1, MMP2, FMN1
intrinsic apoptotic signaling pathway in response to DNA damage	0.0651851	3.282861293	CHEK2, E2F1, HMOX1, BRCA1, BRCA2
positive regulation of cholesterol efflux	0.065205988	7.090980392	PON1, ADIPOQ, APOE
Wnt signaling pathway, planar cell polarity pathway	0.065205988	7.090980392	SFRP1, WNT5A, ROR2
positive regulation of membrane protein ectodomain proteolysis	0.065205988	7.090980392	ADAM8, APOE, ADRA2A
atrioventricular valve morphogenesis	0.065205988	7.090980392	BMP2, GATA4, ZFPM2
detection of temperature stimulus involved in sensory perception of pain	0.065205988	7.090980392	NTRK1, CALCA, EPHB1
SMAD protein signal transduction	0.065858811	2.762719633	BMP2, GATA4, GDF3, INHBA, ROR2, GDF6
positive regulation of neuron differentiation	0.070732236	2.409556444	FOXA1, NGFR, IL6, BMP2, ECT2, CCR5, GDF6
positive regulation of cell adhesion	0.072531783	3.165616246	SELP, CALCA, CHRD, CCL2, ADAM8
defense response to Gram-negative bacterium	0.072531783	3.165616246	PYCARD, CHGA, CD4, SLC11A1, HMGB2
urogenital system development	0.073171349	6.647794118	HELLS, OSR1, EPHB2
positive regulation of T cell migration	0.073171349	6.647794118	PYCARD, CXCL10, ADAM8
response to vitamin D	0.073171349	6.647794118	CXCL10, CD4, SPP1
positive regulation of G2/M transition of mitotic cell cycle	0.073171349	6.647794118	RAD51B, RAD51C, CDC7
positive regulation of cellular protein metabolic process	0.073171349	6.647794118	UHRF1, ADIPOQ, INHBA
positive regulation of cartilage development	0.073171349	6.647794118	BMP2, WNT5A, SOX9
Sertoli cell development	0.073171349	6.647794118	NTRK1, WT1, SOX9
chromosome condensation	0.073171349	6.647794118	TOP2A, CDK1, NCAPD2
kidney development	0.074686271	2.181840121	WT1, ALDH1A2, SERPINF1, HAS2, FMN1, DCN, WNT4, FBN1
acute-phase response	0.074769591	4.051988796	IL6, CD163, UGT1A1, FN1
cellular response to BMP stimulus	0.074769591	4.051988796	SFRP1, BMP2, ADAMTS12, ADAMTS7
response to bacterium	0.074769591	4.051988796	PYCARD, SLC11A1, CD14, FCGR2B
mesoderm formation	0.074769591	4.051988796	CRB2, SNAI1, CHRD, INHBA
response to organic cyclic compound	0.074974163	2.659117647	ALDH3A1, SFRP1, MMP14, LUM, CDK1, MKI67
ovarian follicle development	0.076356516	3.110079119	MMP14, MMP2, MMP19, INHBA, ESR2
growth	0.079936527	3.939433551	BMP2, GDF3, INHBA, GDF6
branching morphogenesis of an epithelial tube	0.079936527	3.939433551	MMP14, MGP, IGF1, WNT4
response to amino acid	0.079936527	3.939433551	IL6, EDN1, ARG1, CCL2
regulation of heart rate	0.079936527	3.939433551	CALCA, CAV3, PMCH, CACNA1G
negative regulation of peptidyl-tyrosine phosphorylation	0.081429295	6.256747405	SFRP1, SFRP2, IGF1
fatty acid oxidation	0.081429295	6.256747405	ADIPOQ, PPARGC1A, CYGB
endocardial cushion morphogenesis	0.081429295	6.256747405	BMP2, TWIST1, SOX9
acute inflammatory response	0.081429295	6.256747405	IL6, CXCL1, ADRA2A
positive regulation of T cell activation	0.081429295	6.256747405	PYCARD, CD4, CCL2
negative regulation of female gonad development	0.08209815	23.63660131	WT1, ZFPM2
negative regulation of transcription involved in G1/S transition of mitotic cell cycle	0.08209815	23.63660131	E2F1, E2F7
extracellular polysaccharide biosynthetic process	0.08209815	23.63660131	HAS1, HAS2
negative regulation of planar cell polarity pathway involved in axis elongation	0.08209815	23.63660131	SFRP1, SFRP2
tendon development	0.08209815	23.63660131	COL5A1, COL11A1
retinal rod cell differentiation	0.08209815	23.63660131	SOX9, PTN
adaptive thermogenesis	0.08209815	23.63660131	UCP1, PPARGC1A
odontoblast differentiation	0.08209815	23.63660131	LEF1, FAM20C
renal vesicle induction	0.08209815	23.63660131	SOX9, WNT4
negative regulation of hair follicle development	0.08209815	23.63660131	NGFR, INHBA
mesenchymal-epithelial cell signaling	0.08209815	23.63660131	WNT2B, WNT5A
deoxyribonucleotide biosynthetic process	0.08209815	23.63660131	RRM1, RRM2
prostate gland stromal morphogenesis	0.08209815	23.63660131	FOXA1, IGF1
endothelial cell-cell adhesion	0.08209815	23.63660131	CYP1B1, THBS4
regulation of endothelial tube morphogenesis	0.08209815	23.63660131	CXCL10, ADAMTS12
response to methionine	0.08209815	23.63660131	PCK1, PPARGC1A
spindle checkpoint	0.08209815	23.63660131	BIRC5, AURKB
negative regulation of epidermal cell differentiation	0.08209815	23.63660131	GDF3, EZH2
dTMP biosynthetic process	0.08209815	23.63660131	DCTD, TYMS

embryonic heart tube anterior/posterior pattern specification	0.08209815	23.63660131	BMP2, GATA4
cellular response to dexamethasone stimulus	0.085254	3.832962374	IL6, ARG1, SERPINF1, CCL2
regulation of calcium ion transport	0.085254	3.832962374	CAV3, IGF1, CACNA2D4, CACNA1G
base-excision repair	0.085254	3.832962374	POLQ, FEN1, LIG1, UNG
organ morphogenesis	0.087425662	2.276920309	TGM1, BMP2, WNT2B, HHIP, WNT5A, SLC26A4, EPHB2
negative regulation of protein phosphorylation	0.088134033	2.532492997	CCNB1, CD109, IGFBP3, PBK, BDKRB1, PPARGC1A
blood coagulation	0.088134033	2.532492997	C3, WEE1, F10, F13A1, F2RL3, F5
response to copper ion	0.089957115	5.909150327	ATP7B, CDK1, LOXL2
positive regulation of nitric-oxide synthase activity	0.089957115	5.909150327	DHFR, NPR3, APOE
digestive tract morphogenesis	0.089957115	5.909150327	SFRP1, SFRP2, WNT5A
drug metabolic process	0.089957115	5.909150327	CYP1A1, FMO3, DCK
chondrocyte development	0.089957115	5.909150327	SFRP2, COL11A1, SOX9
cardiac muscle hypertrophy in response to stress	0.089957115	5.909150327	GATA4, KLF15, EZH2
retinoic acid receptor signaling pathway	0.089957115	5.909150327	CYP26A1, CYP26B1, ALDH1A2
negative regulation of chondrocyte differentiation	0.089957115	5.909150327	SOX9, ADAMTS12, ADAMTS7
mammary gland development	0.090717701	3.732094943	LEF1, IGF1, SOX9, BRCA2
mitotic cell cycle	0.090717701	3.732094943	CENPE, RRM1, KIF18B, AURKA
metanephros development	0.090717701	3.732094943	WT1, OSR1, WNT4, FBN1
protein kinase B signaling	0.090717701	3.732094943	LINGO1, CCL2, IGF1, SOX9
chromosome organization	0.090717701	3.732094943	BLM, BNC1, RAD54L, BRCA2
cell migration	0.091342922	1.85627759	MMP14, CDH2, COL5A1, MDK, MMP2, WNT5A, SNAI1, FSCN1, BDKRB1, ADAMTS12
negative regulation of transcription, DNA-templated	0.092375549	1.408398523	CALCA, LEF1, WNT5A, ADIPOQ, HMGB2, FHL2, TWIST1, BRCA1, FOXM1, LOXL2, MLXIPL, SFRP1, BMP2, FABP4, SFRP2, WT1, SNAI1, E2F1, BIRC5, SOX9, ZFPM2, EZH2, WNT4
regulation of inflammatory response	0.092636017	2.906139505	PYCARD, FABP4, FANCD2, SCGB1A1, ADAMTS12
forebrain development	0.095135915	2.473597811	STIL, ALDH1A2, APLP1, E2F1, CHRD, PPARGC1A
platelet activation	0.096323212	3.636400201	ENTPD2, ADRA2A, F5, PF4
regulation of cell differentiation	0.096323212	3.636400201	HMGB2, LIF, CELA1, SOX9
regulation of cell cycle	0.096514451	2.215931373	FOXA1, CCNE1, CCNF, E2F1, TACC3, SKP2, DTL
regulation of transcription from RNA polymerase II promoter	0.096802912	1.518219983	FOXA1, BNC1, LEF1, HMGB2, UCP1, FHL2, NR1H4, GATA4, INHBA, MLXIPL, BRIP1, MAFB, WT1, SP6, SNAI1, SOX9, EZH2
heart looping	0.09694403	2.859266287	STIL, ALDH1A2, WNT5A, GATA4, FOLR1
regulation of cell shape	0.098000881	2.040569897	CCL24, IL6, CCL11, CCL7, FN1, CCL2, CDC7, PTN
retinoid metabolic process	0.098733142	5.598142415	ALDH1A2, APOE, DHRS3
DNA biosynthetic process	0.098733142	5.598142415	LIG1, TK1, POLE
muscle fiber development	0.098733142	5.598142415	UCHL1, LEF1, FLNC
male meiosis I	0.098733142	5.598142415	RAD51C, TRIP13, BRCA2
regulation of cyclin-dependent protein serine/threonine kinase activity	0.098733142	5.598142415	CCNA2, BLM, CDKN2C
negative regulation of endopeptidase activity	0.098733142	5.598142415	CRB2, SLPI, TIMP1
regulation of ossification	0.098733142	5.598142415	SFRP1, CREB3L1, DHRS3
regulation of transforming growth factor beta receptor signaling pathway	0.098733142	5.598142415	CAV3, NREP, FOLR1
oocyte maturation	0.098733142	5.598142415	CCNB1, TRIP13, BRCA2
behavioral response to cocaine	0.098733142	5.598142415	SDK1, ABAT, ADRA1B
BMP signaling pathway	0.098740129	2.445165652	BMP2, LEF1, GATA4, GDF3, ROR2, GDF6
